# Ecogeography and utility to plant breeding of the crop wild relatives of sunflower (*Helianthus annuus* L.)

**DOI:** 10.3389/fpls.2015.00841

**Published:** 2015-10-08

**Authors:** Michael B. Kantar, Chrystian C. Sosa, Colin K. Khoury, Nora P. Castañeda-Álvarez, Harold A. Achicanoy, Vivian Bernau, Nolan C. Kane, Laura Marek, Gerald Seiler, Loren H. Rieseberg

**Affiliations:** ^1^Biodiversity Research Centre and Department of Botany, University of British ColumbiaVancouver, BC, Canada; ^2^Department of Agronomy and Plant Genetics, University of MinnesotaSt. Paul, MN, USA; ^3^International Center for Tropical AgricultureCali, Colombia; ^4^Centre for Crop Systems Analysis, Wageningen UniversityWageningen, Netherlands; ^5^School of Biosciences, University of BirminghamBirmingham, UK; ^6^Department of Horticulture and Crop Science, The Ohio State UniversityColumbus, OH, USA; ^7^Department of Ecology and Evolutionary Biology, University of Colorado at BoulderBoulder, CO, USA; ^8^Agronomy Department, North Central Regional Plant Introduction Station, Iowa State University and United States Department of Agriculture Agricultural Research ServiceAmes, IA, USA; ^9^Northern Crop Science Laboratory, United States Department of Agriculture Agricultural Research ServiceFargo, ND, USA; ^10^Department of Biology, Indiana UniversityBloomington, IN, USA

**Keywords:** conservation, climate change, crop wild relatives, ecological niche modeling, plant breeding, plant genetic resources, publicly available data sources

## Abstract

Crop wild relatives (CWR) are a rich source of genetic diversity for crop improvement. Combining ecogeographic and phylogenetic techniques can inform both conservation and breeding. Geographic occurrence, bioclimatic, and biophysical data were used to predict species distributions, range overlap and niche occupancy in 36 taxa closely related to sunflower (*Helianthus annuus* L.). Taxa lacking comprehensive *ex situ* conservation were identified. The predicted distributions for 36 *Helianthus* taxa identified substantial range overlap, range asymmetry and niche conservatism. Specific taxa (e.g., *Helianthus deblis* Nutt., *Helianthus anomalus* Blake, and *Helianthus divaricatus* L.) were identified as targets for traits of interest, particularly for abiotic stress tolerance, and adaptation to extreme soil properties. The combination of techniques demonstrates the potential for publicly available ecogeographic and phylogenetic data to facilitate the identification of possible sources of abiotic stress traits for plant breeding programs. Much of the primary genepool (wild *H. annuus*) occurs in extreme environments indicating that introgression of targeted traits may be relatively straightforward. Sister taxa in *Helianthus* have greater range overlap than more distantly related taxa within the genus. This adds to a growing body of literature suggesting that in plants (unlike some animal groups), geographic isolation may not be necessary for speciation.

## Introduction

Plant genetic resources represent the biological foundation for maintaining and improving crop productivity having played a central role in crop development from antiquity (Porter et al., 2014). Crop wild relatives (CWR) are an important source of useful traits for plant breeding (Hoisington et al., 1999; Hajjar and Hodgkin, 2007). With the world's population projected to increase the need to produce more food while using fewer natural resource inputs under increasingly stochastic climatic conditions is a major challenge (Butler and Huybers, 2013; Challinor et al., 2014). CWR conservation and utilization focusing on the use of improving technologies (high throughput phenotyping, genotyping, and geographical information systems), has been proposed as a way to acquire a greater knowledge of conservation needs and lead to more targeted use of CWR germplasm (Khoury et al., 2010; Cabrera-Bosquet et al., 2012; McCouch et al., 2013). Targeted collecting for *ex situ* conservation has become a priority as rapid changes in both climate and land use patterns increasingly threaten CWR in their natural habitats (Jarvis et al., 2008; McCouch et al., 2013).

CWR have traditionally been categorized based on crossing relationships with domesticates; the primary germplasm contains no crossing barriers, the secondary contains some meiotic abnormalities, and the tertiary requires special techniques such as embryo rescue (Harlan and de Wet, 1971; Harlan, 1976). Such classifications may be supplemented by molecular, bioclimatic, and biophysical data to aid in the identification of candidate taxa for breeding, although such efforts have been constrained by challenges in comprehensively generating and integrating these data (Ricklefs and Jenkins, 2011).

The genus *Helianthus* L. contains 52 species comprising 67 taxa (Schilling, 2006; Stebbins et al., 2013). Native to North America, the taxa occupy a variety of habitats ranging from open plains to salt marshes (Seiler and Marek, 2011; Kane et al., 2013). Sunflower (*Helianthus annuus* L.) is the most economically important species from the genus, with ~26 million hectares in production worldwide and a substantial private sector breeding effort, particularly for oil production (FAOSTAT, 2013). Domesticated approximately 4000 years ago in east central North America, sunflower has a typical domestication syndrome; i.e., it does not branch, does not have seed dormancy, has a predictable flowering time, and does not shatter (Harlan et al., 1973; Harter et al., 2004; Blackman et al., 2011). The crop has undergone both selection and genetic drift during domestication and improvement, which has reduced genetic diversity (Tang and Knapp, 2003; Liu and Burke, 2006), with modern cultivars retaining 50–67% of the diversity present in wild *H. annuus* populations (Kolkman et al., 2007; Mandel et al., 2011).

Sunflower has often utilized CWR in breeding efforts, with many of the taxa hybridizing well with the crop (Table S1; Table 1) (Long, 1960; Chandler et al., 1986). Despite the historical use, CWR of sunflower are considered to be relatively untapped, particularly in regard to adaptation to abiotic stresses. To contribute to an enhanced understanding of the CWR of sunflower, this studies' objectives were to (1) create geographical distribution models for 36 CWR taxa, and (2) explore niche habitation through comparisons of ecogeographic and phylogenetic data, to identify taxa occurring in extreme environments of potential interest to sunflower breeding.

**Table 1:** **Taxa examined in this study, recommendation, position in germplasm, environmental cluster, life history, and potential extreme characteristics**.

**Taxa**	**Recommendation for Collection**	**Position in Germplasm**	**Range overlap with *H. annuus***	**Environmental Cluster Assignment**	**Life History**	**Potential Extreme Characteristics Based on Different Ecological Niche Relative to *H. annuus***
*H. annuus* (wild)	Assessed to be well represented	Primary	NA	Cluster 1	Annual	NA
*H. anomalus*	High priority	Secondary	Utah New Mexico	Cluster 3	Annual	Low precipitation tolerance Tolerance to high pH
*H. argophyllus*	Medium priority	Secondary	Texas	Cluster 1	Annual	High temperature tolerance
						Tolerance to high clay content
*H. arizonensis*	Medium priority	Tertiary	Arizona New Mexico	Cluster 3	Perennial	Response to stochastic climate Low precipitation tolerance Tolerance to low bulk density
*H. atrorubens*	Medium priority	Tertiary	No overlap	Cluster 2	Perennial	Tolerance to low
						Cation-exchange capacity
						Tolerance of high precipitation
						Tolerance to low pH
*H. bolanderi*	High priority	Secondary	California	Cluster 1	Annual	Tolerance to erratic precipitation
						Low precipitation tolerance
*H. debilis* subsp. *cucmerifolius*	High priority	Secondary	East Texas	Cluster 2	Annual	High temperature tolerance
*H. debilis* subsp. *debilis*	Medium priority	Secondary	No overlap	Cluster 2	Annual	High temperature tolerance
						Tolerance of high precipitation
						Tolerance to low clay content
*H. debilis* subsp. *silvestris*	Medium priority	Secondary	No overlap	Cluster 2	Annual	Tolerance to high clay content
*H. debilis* subsp. *tardiflorus*	Assessed to be well represented	Secondary	No overlap	Cluster 2	Annual	Tolerance of high precipitation Tolerance to low clay content
*H. debilis* subsp. *vestitus*	Low priority	Secondary	No overlap	Cluster 2	Annual	High temperature tolerance Tolerance of high precipitation
						Tolerance to low clay content
*H. deserticola*	High priority	Secondary	Nevada	Cluster 3	Annual	Response to stochastic climate
			Utah			Low precipitation tolerance
			New Mexico			
*H. divaricatus*	High priority	Tertiary	Central US	Cluster 2	Perennial	Perennial habit
						Tolerance to low pH
*H. exilis*	Medium priority	Secondary	California	Cluster 1	Annual	Tolerance to erratic precipitation
						Low precipitation tolerance
						Low bulk density
*H. giganteus*	High priority	Tertiary	No overlap	Cluster 2	Perennial	Tolerance of high precipitation
*H. grosseserratus*	Medium priority	Tertiary	Central US	Cluster 3	Perennial	Tolerance to erratic temperature
*H. hirsutus*	High priority	Tertiary	Central US	Cluster 2	Perennial	Tolerance to low pH
*H. maximilliani*	High priority	Tertiary	Central US	Cluster 3	Perennial	Low temperature tolerance
						Tolerance to erratic temperature
*H. neglectus*	Assessed to be well represented	Secondary	New Mexico	Cluster 1	Annual	Low organic carbon content
*H. niveus* subsp. *canescens*	High priority	Secondary	California Arizona New Mexico	Cluster 1	Annual Rarely Perennial	High temperature tolerance Low precipitation tolerance
*H. niveus* subsp. *niveus*	High priority	Secondary	Baja California	Cluster 1	Perennial	Low precipitation tolerance
*H. niveus* subsp. *tephrodes*	High priority	Secondary	California, Mexico (Sonora)	Cluster 1	Perennial Sometime Annual	High temperature tolerance low Precipitation tolerance
*H. paradoxus*	Assessed to be well represented	Secondary	Texas, New Mexico	Cluster 1	Annual	Low organic carbon content
*H. pauciflorus* subsp. *pauciflorus*	High priority	Tertiary	Central US	Cluster 3	Perennial	Tolerance to erratic temperature
*H. pauciflorus* subsp. *subrhomboideus*	High priority	Tertiary	Central US	Cluster 3	Perennial	Low temperature tolerance Tolerance to erratic temperature
*H. petiolaris* subsp. *fallax*	High priority	Secondary	Western US	Cluster 3	Annual	Tolerance to erratic temperature
*H. petiolaris* subsp. *petiolaris*	High priority	Secondary	Central US	Cluster 3	Annual	Tolerance to erratic temperature Low temperature tolerance
*H. praecox* subsp. *hirtus*	Assessed to be well represented	Secondary	West Texas	Cluster 1	Annual	High temperature tolerance
*H. praecox* subsp. *praecox*	Assessed to be well represented	Secondary	East Texas	Cluster 2	Annual	Tolerance to erratic temperature
*H. praecox* subsp. *runyonii*	Low priority	Secondary	Texas	Cluster 1	Annual	Tolerance of high bulk density
*H. resinosus*	Medium priority	Tertiary	No overlap	Cluster 2	Perennial	Tolerance of high precipitation
						Tolerance to low
						Cation exchange capacity Tolerance to low pH
*H. salicifolius*	Medium priority	Tertiary	Oklahoma Kansas Arkansas Missouri	Cluster 3	Perennial	Tolerance to high clay content
*H. silphioides*	Assessed to be well represented	Tertiary	Oklahoma Arkansas Missouri	Cluster 2	Perennial	Tolerance to low cation-exchange capacity Tolerance to low pH
*H. strumosus*	High priority	Tertiary	Central US	Cluster 2	Perennial	Tolerance of high precipitation
*H. tuberosus*	Medium priority	Secondary	Central US	Cluster 2	Perennial	Low temperature tolerance
*H. winteri*	High priority	Primary	California	Cluster 1	Perennial	High temperature tolerance

## Materials and methods

### Species distribution modeling

A modified gap analysis (Ramírez-Villegas et al., 2010) was used to determine the conservation status of 36 taxa within *Helianthus* selected based upon their potential to provide useful traits for sunflower breeding. Briefly, (1) target taxa were identified, and geographic occurrence data were gathered and verified, (2) the overall representation of CWR in germplasm collections was estimated, (3) potential distribution models were produced for taxa with sufficient samples with coordinates, (4) the geographic and ecological representation of germplasm collections were assessed for each taxon by comparing potential distribution models to existing germplasm collection locations, (5) taxa were prioritized for further collecting based upon the average of their overall, geographic, and ecological coverage results, and (6) gap analysis results were correlated with the subjective assessments of collection priorities from crop experts.

The selection of taxa for analysis was based on membership within the primary or secondary genepools of sunflower (Vincent et al., 2013) with the addition of all taxa from the tertiary genepool indicated in publications to be confirmed or potential trait donors (Table S1). A total of 12,737 occurrence records for the 36 taxa, sourced from 31 herbaria and five genebanks, were used for distribution models and conservation analysis (Table S2), including 4705 records with geographic coordinates. The overall representation of taxa in genebank collections was estimated using the “Sampling Representativeness Score” (SRS), calculated as the number of germplasm samples (GS) divided by the total number of samples (GS plus reference records). After eliminating duplicate records, potential distributions were calculated using Maxent (Phillips et al., 2006), with a k-5 cross-validation option and 10,000 background points for model training over North America (Phillips, 2008; VanDerWal et al., 2009). We included 19 bioclimatic variables derived from the WorldClim database (Nix, 1986; Hijmans et al., 2005a,b), seven biophysical variables from the ISRIC—World Soil Information database (http://soilgrids1km.isric.org) at a resolution of 2.5 arc-min, and the occurrence information (coordinates) for each taxon as inputs (Table S3). For edaphic data we calculated a weighted mean from five depths (0–5, 5–15, 15–30, 30–60, 60–100 cm) to generate a single value for the first meter of soil for each layer, and then resampled the data from 1 to 2.5 arc min resolution to match the WorldClim dataset, using the raster package in R and ArcGIS Desktop 10.1 (Hengl et al., 2014). Distributions were further restricted by applying a taxon independent threshold, based on the Receiver Operating Characteristic (ROC) curve (Liu et al., 2005). GRIN distribution data was used to ensure that taxa distributions were not overinflated beyond known native boundaries (USDA, 2007). Soil cover data from GlobCover 2009 (Global Land Cover Map) (http://due.esrin.esa.int/page_globcover.php) further refined the maxent outputs and collecting maps by excluding urban areas, water bodies, bare areas, and permanent snow and ice regions.

Potential distribution models were considered accurate if they complied with the following conditions: (i) five-fold average area under the test ROC curve (ATAUC) is greater than 0.7, (ii) the standard deviation of ATAUC (STAUC) is less than 0.15, and (iii) At least 10% of grids for each model has standard deviation less than 0.15 (ASD15). For taxa whose Maxent model did not comply, potential distributions were estimated by forming a circular buffer of 50 km around each occurrence point for each species.

Geographic representativeness of taxa in genebank collections was calculated using the “Geographic Representativeness Score” (GRS), comparing the spatial overlap of a circular buffer surrounding each accession record (50 Km radius as described in Hijmans et al., 2001) against the potential distribution of the taxon. Ecological gaps in genebank collections were calculated using the “Ecological Representativeness Score” (ERS), calculated by comparing records to the full environmental range of the modeled taxon across ecosystem types (Olson et al., 2001). The overall priority for further collecting for *ex situ* conservation for each taxon was determined by averaging the SRS, GRS, and ERS with equal weight to obtain a final prioritization score (FPS), classified according to the following ranges: 1., high priority (FPS between 0 and 3); 2., medium priority (FPS between 3.01 and 5); 3., low priority (FPS between 5.01 and 7.5); and 4., and well conserved taxa (FPS between 7.51 and 10).

### Expert evaluation of conservation assessment results

**Figure 1:**
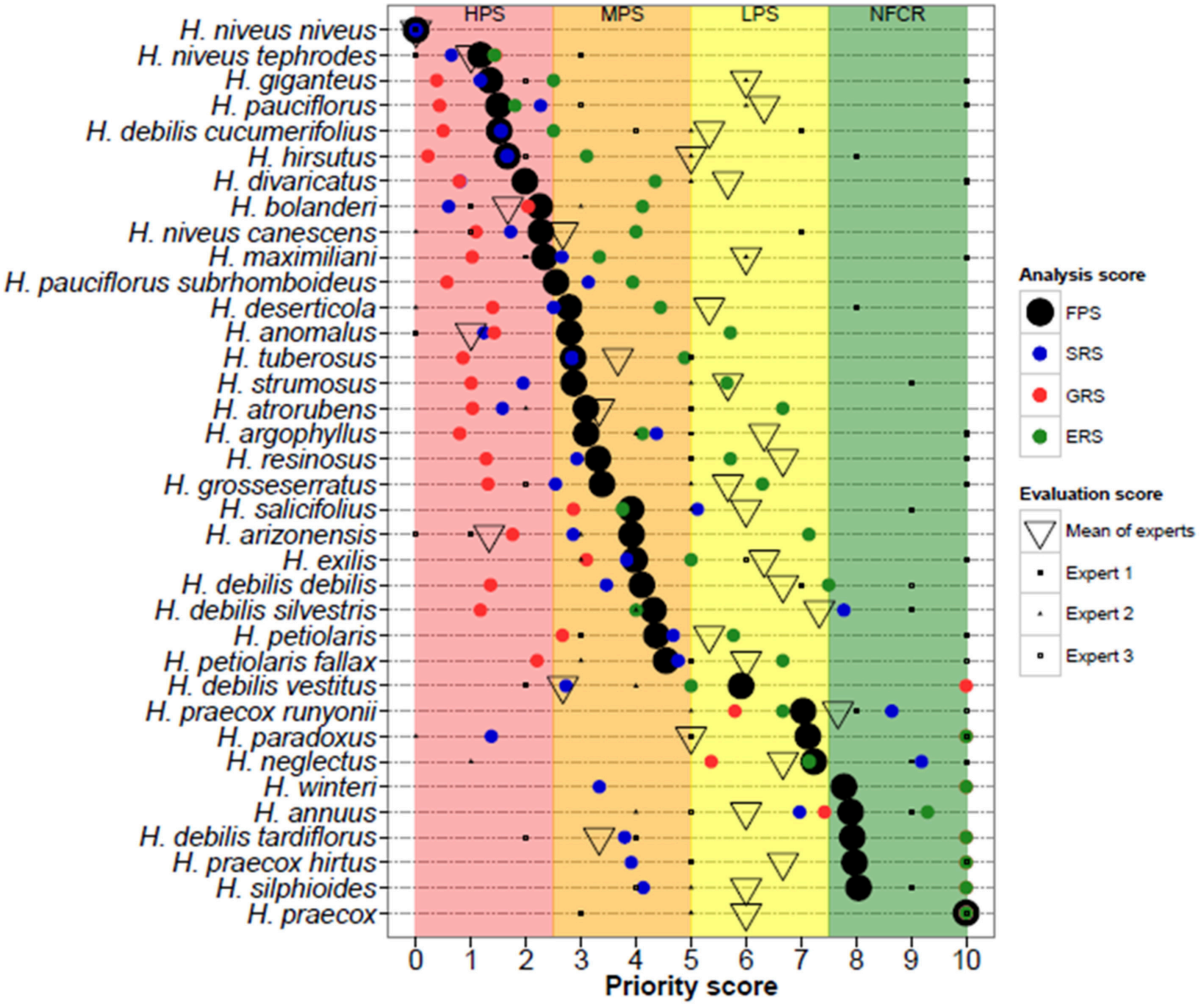
**Synthesis of gap analysis results and expert assessments for each of the 36 ***Helianthus*** CWR taxa surveyed**. Taxa are listed by descending priority for further collecting by category: HPS, high priority taxa; MPS, medium priority taxa; LPS, low priority taxa; NFCR, no further collecting recommended. The final priority scores (FPS, black circle) is the mean of the sampling representativeness score (SRS, blue circle), geographic representativeness score (GRS, red circle), and ecological representativeness score (ERS, green circle).

Predicted taxon distributions based on genebank and herbarium records were compared to the knowledge of four crop experts with experience with *Helianthus* distributions, systematics, conservation, and diversity. *Helianthus* experts were asked to evaluate of the adequacy of germplasm collections per species based on their knowledge of total accessions conserved, geographic, and environmental gaps. This assessment was given an expert priority score (EPS), analogous to the FPS score. A second score was generated, the contextual EPS, which based on additional knowledge such as *in situ* threats and utility to crop breeding. After initial evaluation the experts were asked to review the quantitative results, occurrence data, potential distribution models, and maps of collecting priorities. Following expert input, occurrence data were refined through elimination of incorrect points and adjustment native areas. Potential distribution modeling and gap analyses were then conducted using refined datasets to create more accurate species distribution maps. Potential zones for collecting were identified for each high priority taxon, and then combined to create maps depicting areas where multiple taxa of high priority for conservation could be collected (Figure 1).

### Ecogeographic niche overlap and phylogenetic analyses

Potential distribution probability outputs were used when Maxent models performed well and CA50 sample buffers when Maxent models did not pass the validation criteria, to calculate niche overlap based on Schoener's D and Hellinger's I as outlined in Warren et al. (2008), and implemented in the R package Phyloclim (Heibl, 2011). Both indices utilize probability distributions in geographic space, with statistics ranging from 0 (no niche overlap) to 1 (complete niche overlap). First pairwise niche overlap was examined, then niche overlap between allopatric/sympatric taxa separately, annual/perennial taxa separately, and lastly allopatric/sympatric sister taxa. Geographic range overlap for all pairwise combinations (630 comparisons) was calculated in two ways, with respect to the larger range [(2^*^number of shared grid cells)/(number of grid cells in taxa A + number of grid cells in taxa B)] and with respect to the smaller range [(2^*^number of shared grid cells)/(Total number of grid cells in taxa A + Total number of grid cells in taxa B)]/(Total potential number of shared grid cells) [(2^*^total number of grid cells in species with the smaller range)/(Total number of species A + Total number of species B)].

Principal component analyses (PCA) were used to assess the importance of ecogeographic variables (Table S3) to variation in occurrence data of distribution models per taxon. A hierarchical cluster of principal components (HCPC) identified climatic clusters using R package FactoMineR (Husson et al., 2014). Boxplots for each bioclimatic and biophysical layer were created based on occurrence data points (Figure S1). Ecogeographic variables for cultivated sunflower were extracted from the area of species distribution maps (Monfreda et al., 2008) at a resolution of 5 arc-min, with a random sample of 1000 points weighted by harvested area taken from major production regions.

We downloaded the publically available 18S-26S Ribosomal DNA sequence from the external transcribed spacer (ETS) from GenBank (NCBI-http://www.ncbi.nlm.nih.gov/) for 28 of the 36 *Helianthus* taxa, aligned the sequences using ClustalW, and constructed a maximum likelihood phylogeny with 1000 bootstrap replications, using MEGA6 with a Jukes-Cantor nucleotide substitution model (Tamura et al., 2013). We performed a Mantel test in R utilizing the ade4 package to explore the relationship between geography and genetics (Dray and Dufour, 2007). We estimated phylogenetic signal of individual ecogeographic traits utilizing Blomberg's K (Blomberg et al., 2003), using the multiphylosignal command with 1000 permutations in Picante (Kembel et al., 2010).

## Results

### Geographic distributions of sunflower crop wild relatives

**Figure 2:**
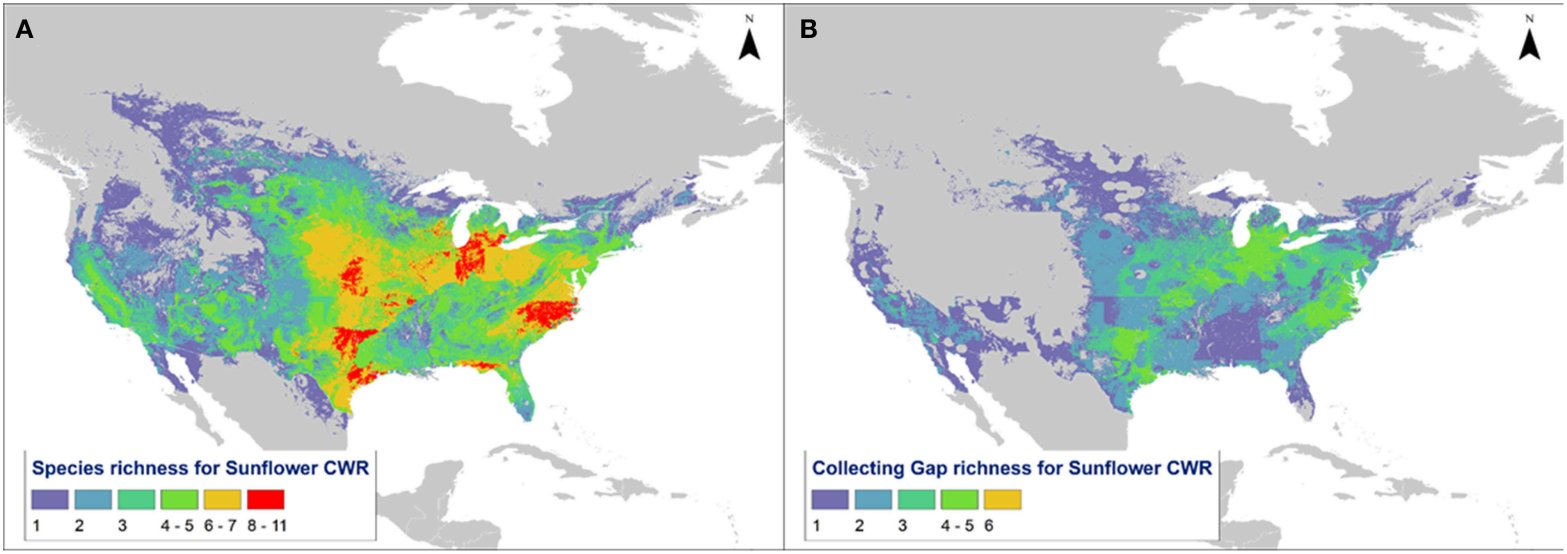
**Map of North America showing (A) taxon richness of sunflower and (B) hotspots for further collecting of high priority taxa**.

**Figure 3:**
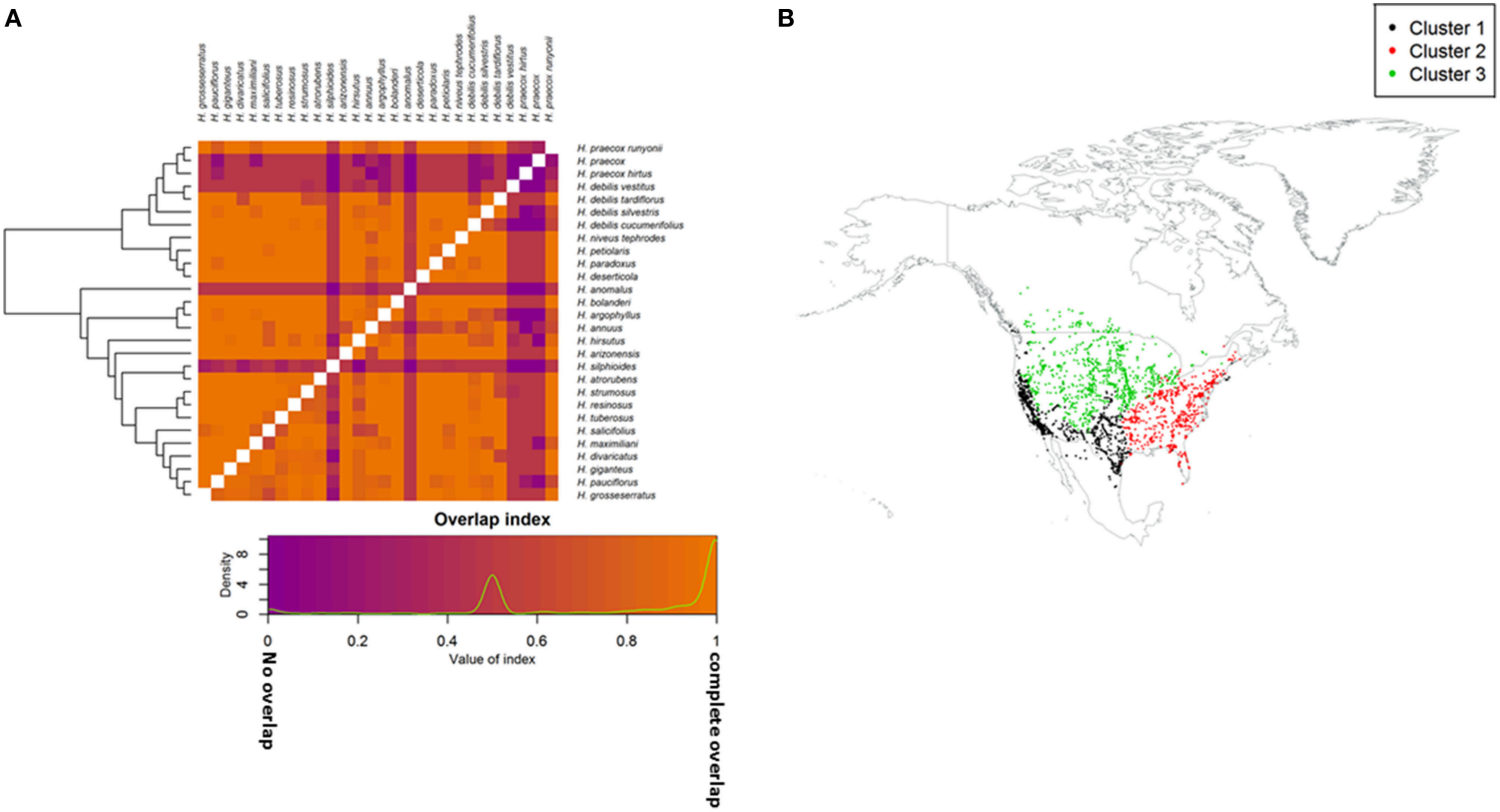
**(A)** Geographic niche overlap based on ecogeographic variables, Schoener's D (above diagonal) and modified Hellinger's I (below diagonal). Taxa are grouped by phylogenetic relationship. Values range from 0 (no overlap; purple) to 1 (complete overlap; orange); **(B)** Occurrence points for assessed taxa grouped based on the first three principle components of biophysical and bioclimatic variables. Clusters share homogeneous bioclimatic and biophysical conditions.

Predicted distribution maps were produced for 36 *Helianthus* taxa, along with taxon richness and collecting hotspot maps (Figure 2; Figure S2). Thirty of the 36 taxa (83%) produced valid maxent models with utilization of soil pH and percent sand greatly improving the accuracy of distribution models, as assessed by expert opinion (Figure 3). Five hotspots (areas of high taxon-level diversity) were identified in the USA, including the southeastern gulf coast, the south-central, the midwest, the north central, and the central east coast (Figure 2A). Our results suggest that half of the 36 taxa are in urgent need of further collecting (high priority species—HPS), along with 28% in moderate need (medium priority species—MPS), 6% of low priority (LPS), and 17% that are well represented in existing germplasm collections and thus do not require urgent additional collecting (Table 1). While the primary genepool taxa has been well collected, only 10% of the taxa in the secondary genepool are well represented across their geographic, climatic, and edaphic ranges. Likewise, only 7% of taxa in the tertiary genepool were assessed as well-conserved (Figure 1; Table 1). These results contrasted with those of expert reviewers, who classified more species as LPS. The discrepancy between the results and expert opinion was due in part to overly optimistic distribution models regarding likelihood of occurrence, in comparison to expert realities of existence of populations in these regions. Additionally, experts assessed some taxa, such as *Helianthus debilis* ssp. *cucumerifolius*, at lower priority because distributions have expanded recently as weedy populations invade new areas, and such regions were not considered by the experts as of particular priority.

### Ecological niches of sunflower crop wild relatives

Three ecogeographic clusters differentiate the taxa, with the first three PCs accounted for 74.3% of the variation (Figure 3B; Table S4). Clusters broadly corresponded to plain, desert, and woodland ecosystems (Table 1). Cluster one was mostly composed of the secondary germplasm and differentiated by temperature, while cluster two was mostly the tertiary germplasm and differentiated by precipitation. Cluster three was differentiated by soil and was evenly split between the secondary and tertiary germplasm (Table S3). It is important to note that PCA can increase type one error, so ecological niches must be carefully examined and validated (Revell, 2009; Uyeda et al., 2015). Schoener's D and Hellinger's I identified substantial niche overlap with few taxa showing niche divergence (Figure 3; Table 1).

Potential geographic distributions of crop wild relative taxa were examined for overlap with wild *H. annuus* (Figure S1); most (81%) taxa exhibited some geographic range overlap with *H. annuus* (Table 1). Among CWR taxa, 39% of pairwise comparisons had overlapping geographic distributions (sympatry), while 61% were allopatric (Table S5; Figure S3). Eight of the 12 sister taxa pairs among the CWR showed some level of sympatry (Table S6). There was considerable range asymmetry between taxa (Figure S1), with the amount of overlap depending on the direction of the comparison, where the smaller range showed 26% more overlap on average than the larger range (Table S5).

**Figure 4:**
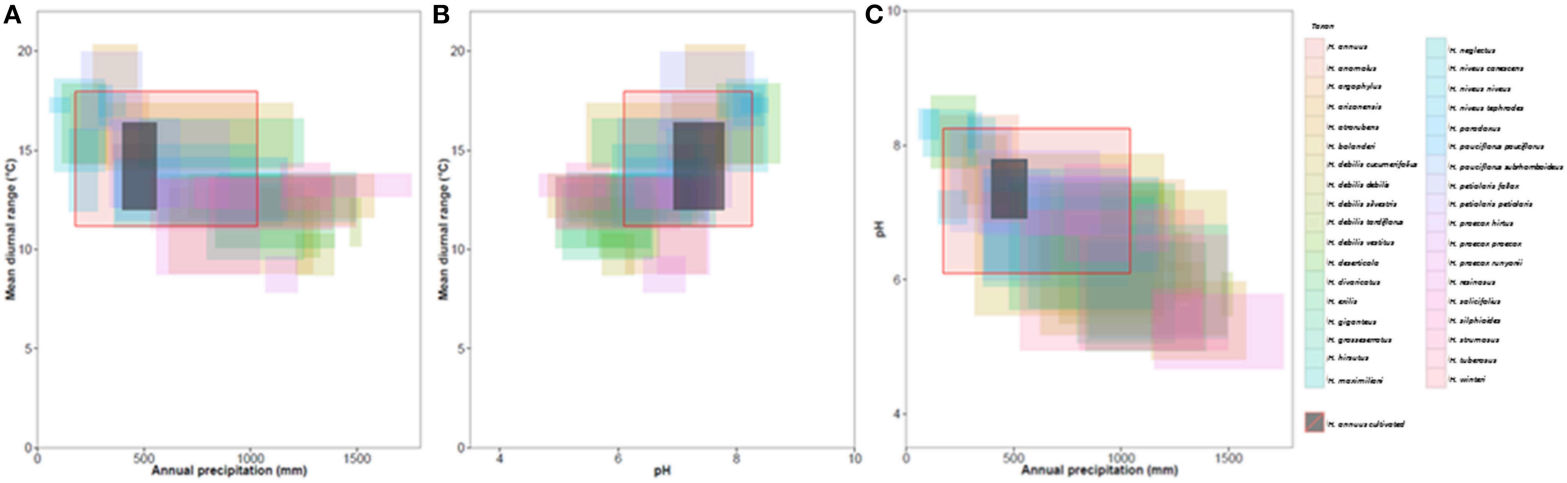
**Climatic niches for (A) mean diurnal range and annual precipitation, (B) Soil pH and mean annual precipitation, (C) mean diurnal range and annual precipitation**. Niches per taxa represent the middle 90% of occurrence points, i.e., 10% outliers are not included. Red boxes show the niche of wild *H. annuus* and black boxes show the niche of cultivated *H. annuus* in North America.

**Figure 5:**
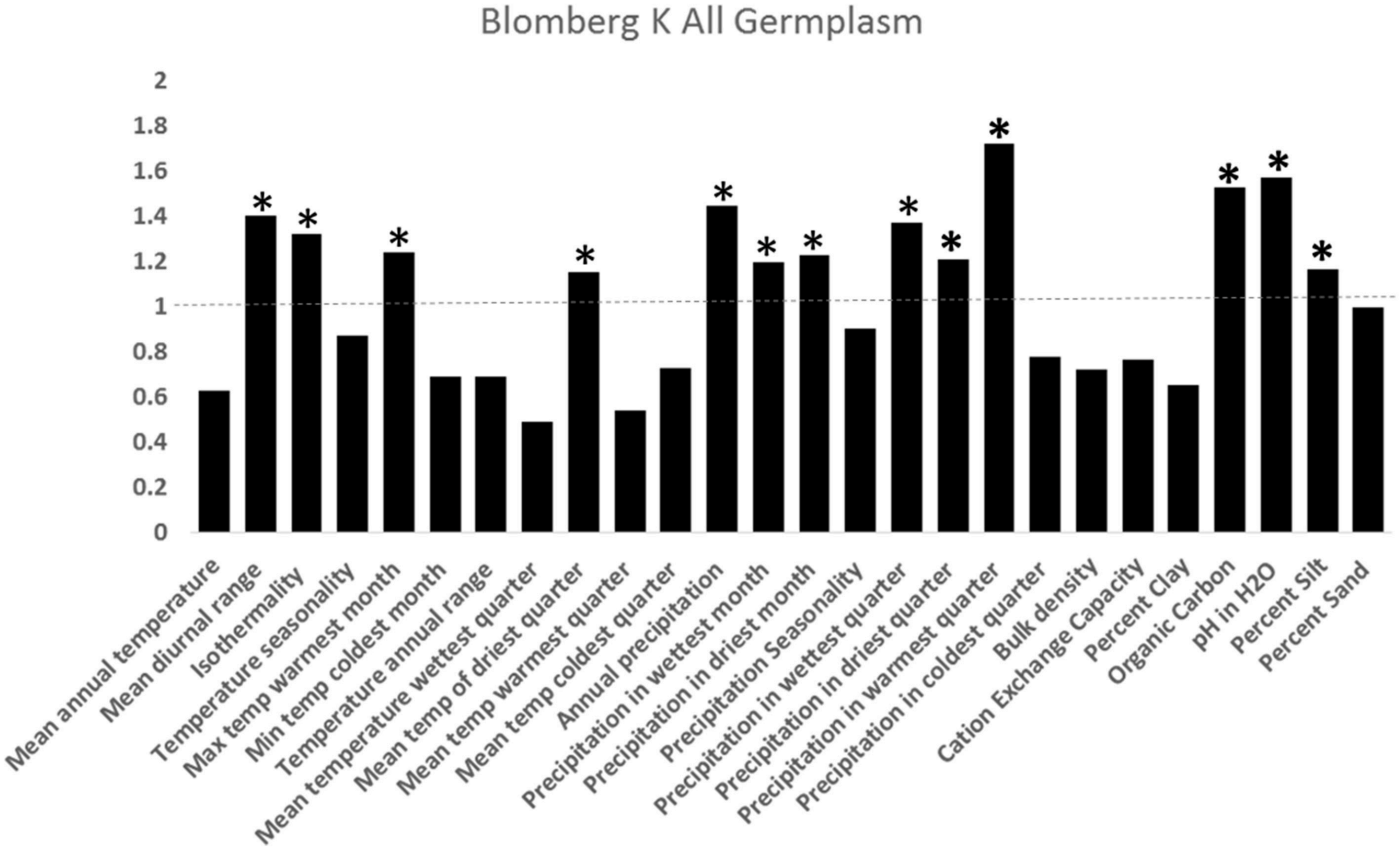
**Test of phylogenetic signal utilizing the ***K*** for 25 of 36 taxa analyzed with complete genetic and environmental information (Blomberg et al., 2003)**. *K* measures phylogenetic signal in traits, where *K*-values below 1 indicates low dependence of traits on evolutionary history (not conserved between taxa) and *K*-values above 1 indicates trait conservation over evolutionary history (traits conserved over evolutionary time). ^*^indicates *K* significantly greater than 1 (*p* < 0.05).

There was general niche conservatism even for sister-taxa (Figure 3; Table 2). While ecogeographic niches were fairly similar for many variables, occasionally there was substantial divergence (Figure 4; Figures S1, S4). Phylogenetic niche conservatism was found in ~54% of variables (Figure 5). Divergence was found in several soil variables suggesting an important role of soil in *Helianthus* diversification. A Mantel's test using Mahalanobis distance (*r* = 0.1423, *p* = 0.01), indicated that taxa that are geographically close are generally more closely related genetically. Notable exceptions to this were *H. maximilliani, H. grosseserratus*, and *H. giganteus*, which are sympatric with *H. annuus*, but are distantly related.

**Table 2:** **Environmental Niche occupancy based on Schoener (1968) D and a modified Hellinger's I (Warren et al., 2008)**.

	**Perfect Overlap (%)**	**D or I Greater than 0.5 (%)**	**D or I Less than 0.2 (%, Divergent Niche)**
All taxa	36.9	69.4	4.7
Annual taxa	32.2	36.6	6.6
Perennial taxa	19.8	85.7	2.2
Allopatric taxa	54.2	62.5	4.3
Sympatric taxa	3.3	83.3	2.6
Sister taxa	33.3	57.7	2.6

## Discussion

There has been increased effort to digitize data related to plant species in general and CWR in particular. The public databases (GBIF, ISRIC, WorldClim, National Germplasm repositories, DivSeek) that archive these data are an increasingly important tool to conservationists, evolutionary biologists and plant breeders. Utilizing public data can reduce the research costs in terms of people hours and consumables to achieve desired environmental and food production goals. Exploring public databases can provide a targeted way to identify accessions for introgression that can then be used to validate predicted extreme variation. This may be a way to more quickly utilize germplasm collections and provide a link to international initiatives aimed at facilitating more use of plant genetic resources (www.DivSeek.org). Here we have used geographic occurrence, bioclimatic, and biophysical data to predict species distributions, range overlap, and niche occupancy in 36 *Helianthus* taxa that are cross-compatible with cultivated sunflower and thus likely to be useful in crop breeding. As discussed briefly below, our results not only have implications for conservation genetics and breeding in *Helianthus*, but they also impact our understanding of the role of geography in the origin of species in this group.

### Implications for conservation and plant breeding

Our approach is both new and complementary to previous work on *Helianthus* species distributions and CWR in the literature (Thompson et al., 1981; Rogers et al., 1982). The method of constraining ranges to known native distributions may have limited our identification of some the extreme variation. Despite this, many taxa that diverge ecologically from cultivated sunflower were identified (Figure 4; Table 1). It was also possible to identify extreme populations within taxa that showed potential adaptation to different ecological niches.

Taxa with larger ranges tend to have greater resilience to changes in environmental conditions than taxa with more limited distributions (Sexton et al., 2014; Sheth and Angert, 2014). Thus, the latter may be considered a primary priority for conservation. Several taxa have expanded far beyond their historical ranges, including *H. annuus, H. petiolaris* Nutt., *H. argophyllus* Torrey and Gray, *H. giganteus* L. and *H. tuberosus* L. While taxa from the non-native parts of their ranges have not been prioritized, existing accessions from such ranges are acknowledged, and may be worthwhile for exploration for traits useful in crop breeding.

Clustering of CWR by environmental variables has great utility by allowing genetic resources to be exploited in a more targeted manner. For example, with respect to soil pH the taxa *H. atrorubens, H. resinosus*, and *H. deserticola* occupy different ecological space from cultivated *H. annuus* (Figure 4). These taxa represent potential candidates for tolerance to acid or alkaline soils, particularly to improve the ability of the crop to accumulate heavy metals for phytoremediation (Fassler et al., 2010). Surprisingly, when examining the properties of the primary, secondary, and tertiary germplasm, often extreme profiles are found in the primary germplasm. This is fortuitous since introgression from primary germplasm is more likely to be successful (Figure 4; Figure S1; Table S7). Approximately 650 wild *H. annuus* accessions are conserved in genebanks which occur outside the ecological parameters of the cultivar (Table S7). The general reduction of environmental diversity occupied by the cultivated sunflower relative to wild *H. annuus* may indicate the reduction in genetic diversity occurring through domestication.

Recent advances in plant and animal breeding (e.g., marker assisted selection, genomic selection) have been facilitated by low cost molecular marker technologies resulting in new tools that can be used to broaden the genetic base in crops (Tester and Langridge, 2010). These methods can shorten breeding cycles, increasing genetic gain per unit time, and allow for wider crosses to be utilized by minimizing linkage drag (Bernardo, 2008). The recent development of genome wide marker sets (Bowers et al., 2012; Renaut et al., 2013) and release of the *H. annuus* genome (Kane et al., 2011; http://www.sunflowergenome.org) facilitate the use of marker assisted selection (Iftekharuddaula et al., 2011) by decreasing costs and increasing data resolution. Further, if germplasm collections are genotyped, these data can be used to associate particular allelic variants with environmental adaptation (Fang et al., 2014).

### Range overlap of wild relatives of sunflower

Sister species in *Helianthus* often have overlapping ranges, an observation that is consistent with sympatric and “budding” speciation (parapatric or peripheral range speciation). Substantial range asymmetry among some (but not all) sister species is also consistent with a budding speciation scenario (Table S6). The amount of range overlap between sister taxa in *Helianthu*s is similar to recent reports from other plant genera, but different from many animal groups, where allopatry tends to be the rule in speciation (Mayr, 1954; Soltis et al., 2004; Quenouille et al., 2011; Anacker and Strauss, 2014). This may suggest that geographic isolation is less critical to plant than animal speciation, perhaps because of the low vagility of many plant species.

Unlike sympatric congeners in other plant groups (Anacker and Strauss, 2014; Grossenbacher et al., 2014), *Helianthus* sister taxa typically lack strong ecological divergence. This observation is inconsistent with most models of speciation involving gene flow, which assume divergent ecological selection (Via, 2009). Possibly, our analyses lacked sufficient resolution or focus on key ecological attributes to detect real differences between the ecological niches of these species. For example, it is possible that there has been pollinator and phenological divergence between sister species that was not included in our analyses. Alternatively, local niche differences between sympatric populations may have been masked by substantial ecological heterogeneity among populations of the more widely ranging species. Additionally, the approach used was designed to analyze potential habitat in the historical, native range, rather than recent range expansions, which in many cases may be recent introductions facilitated by humans, perhaps accounting for observations of limited ecological divergence.

Our analyses imply that many *Helianthus* taxa have similar ecological niches and exhibit niche conservatism. Under niche conservatism, greater allopatric and parapatric speciation is predicted, as habitat fragmentation is expected to contribute to reproductive isolation (Loera et al., 2012). While such a speciation strategy would be surprising given the overlap in geographic range of sister species within *Helianthus*, this trend has been observed in North American *Ephedra* (Loera et al., 2012). That larger amount of niche conservatism observed here than in other systems may be due to properties of the K-statistic, which can have inflated values in polyphyletic phylogenies and in the presence of incomplete lineage sorting, both of which occur in *Helianthus* (Rosenthal et al., 2002; Gross and Rieseberg, 2005; Horandl and Stuessy, 2010; Davies et al., 2012).

## Conclusions

Using a combination of gap analysis, environmental niche modeling, and phylogenetic approaches 36 CWR of sunflower were examined. Taxa that are under-represented in germplasm collections as well as species and populations inhabiting environmental niches with extreme phenotypes that may possess traits of value to crop improvement were identified. In *Helianthus*, sister taxa appear to occur more frequently in sympatry than allopatry, possibly suggesting that speciation may occur in the presence of gene flow. Finally, much of the primary genepool occurs in extreme environments indicating that utilization of wild *H. annuus* for the breeding of abiotic stress tolerance may produce quick gains with minimal effort.

### Conflict of interest statement

The authors declare that the research was conducted in the absence of any commercial or financial relationships that could be construed as a potential conflict of interest.
