# Supplementary material for: Ecogeography and utility to plant breeding of the crop wild relatives of sunflower (Helianthus annuus L.)
Source: Front Plant Sci. 2015 Oct 8;6:841. doi: 10.3389/fpls.2015.00841 (PMC4597133; doi:10.3389/fpls.2015.00841)
Supplement: Supplementary file 8 [file Image1.PDF]

Figure S1. Climatic niches of *Helianthus* species per bioclimatic variable

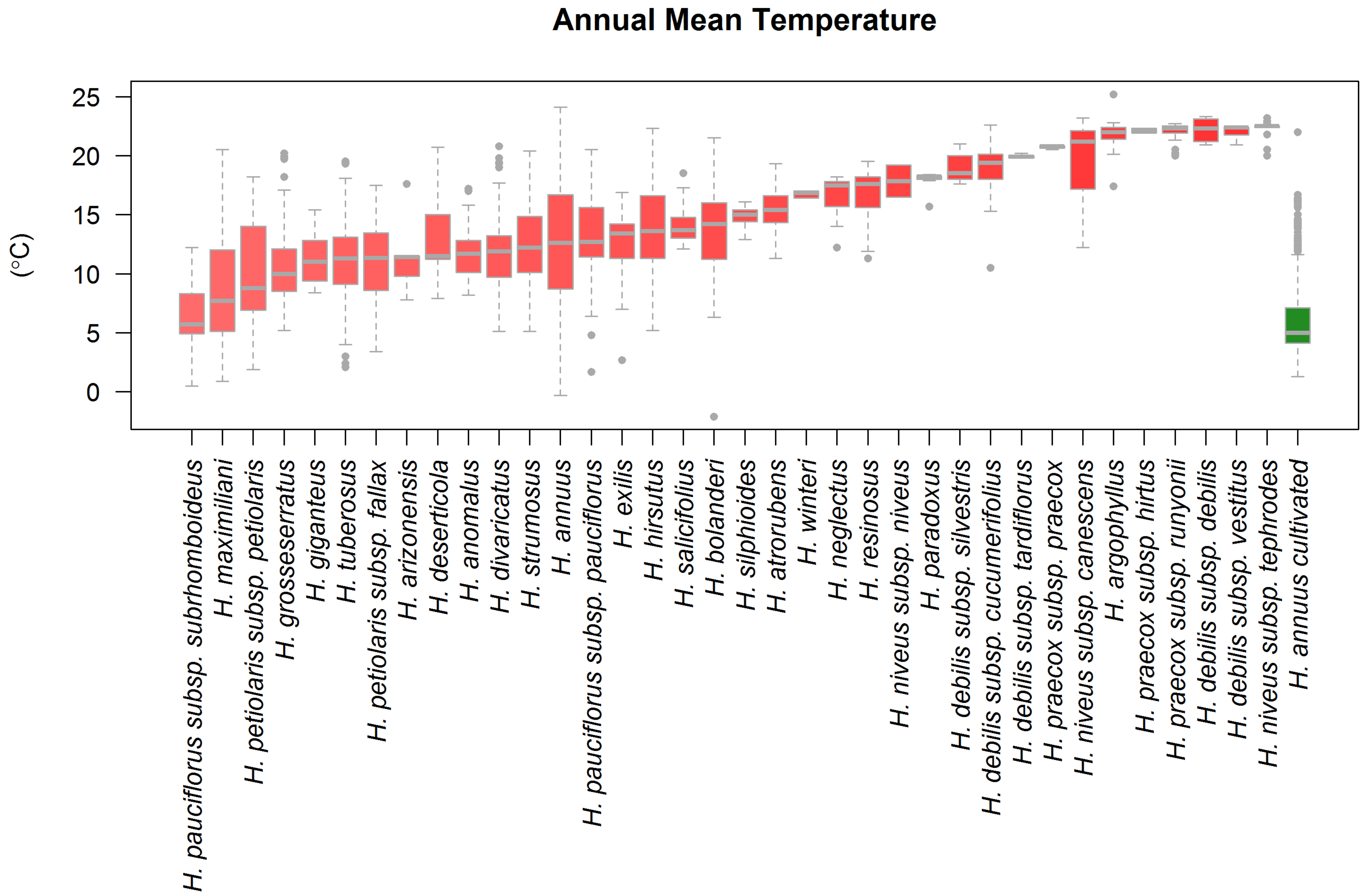

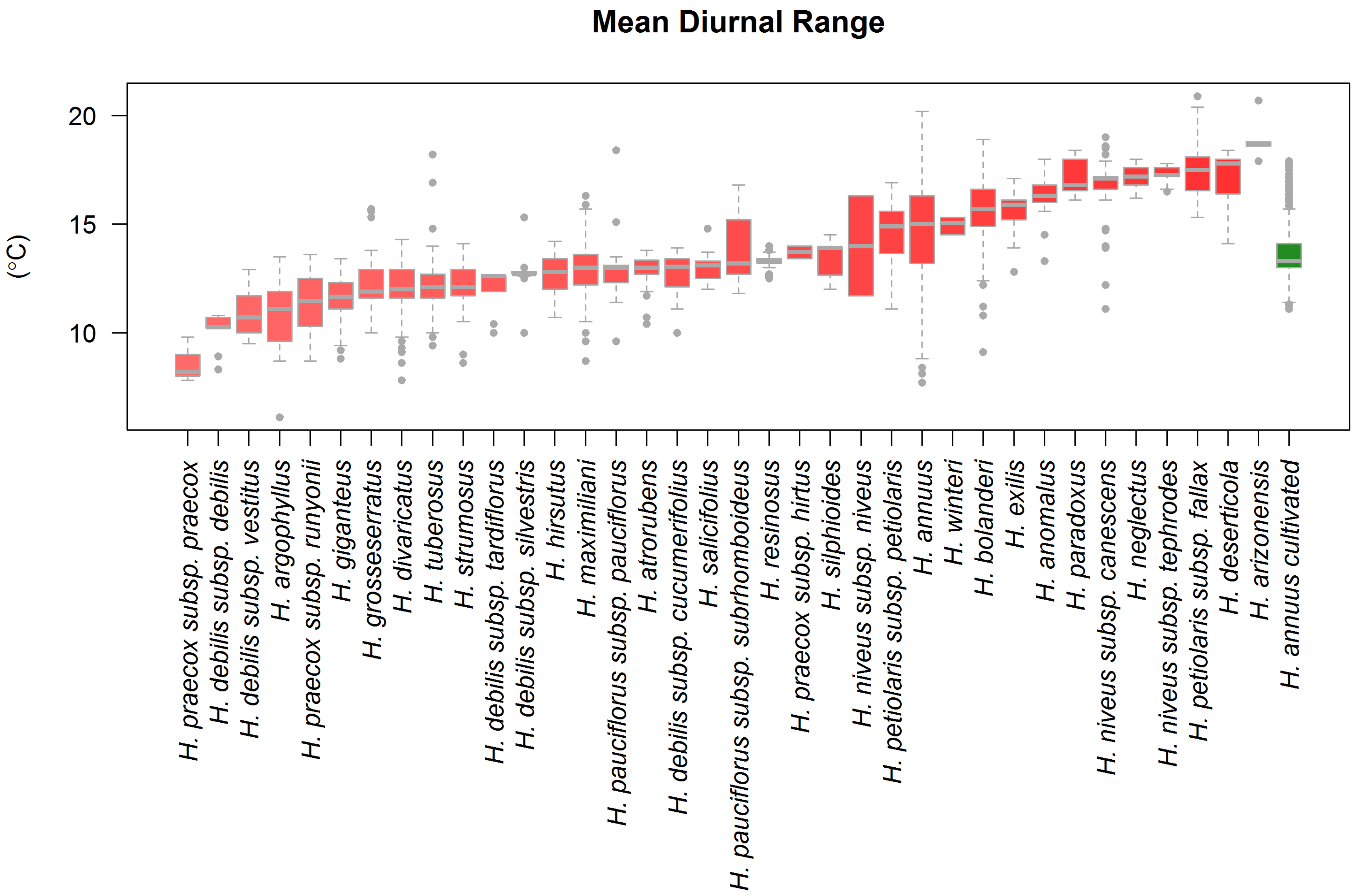

Mean Diurnal Range
(°C)
20
15
10
H. praecox subsp. praecox
H. debilis subsp. debilis
H. debilis subsp. vestitus
H. argophyllus
H. praecox subsp. runyonii
H. giganteus
H. grosseserratus
H. divaricatus
H. tuberosus
H. strumosus
H. debilis subsp. tardiflorus
H. debilis subsp. silvestris
H. hirsutus
H. maximiliani
H. pauciflorus subsp. pauciflorus
H. atrorubens
H. debilis subsp. cucumerifolius
H. salicifolius
H. pauciflorus subsp. subrhomboideus
H. resinosus
H. praecox subsp. hirtus
H. silphioides
H. niveus subsp. niveus
H. petiolaris subsp. petiolaris
H. annuus
H. winteri
H. bolanderi
H. exilis
H. anomalus
H. paradoxus
H. niveus subsp. canescens
H. neglectus
H. niveus subsp. tephrodes
H. petiolaris subsp. fallax
H. deserticola
H. arizonensis
H. annuus cultivated

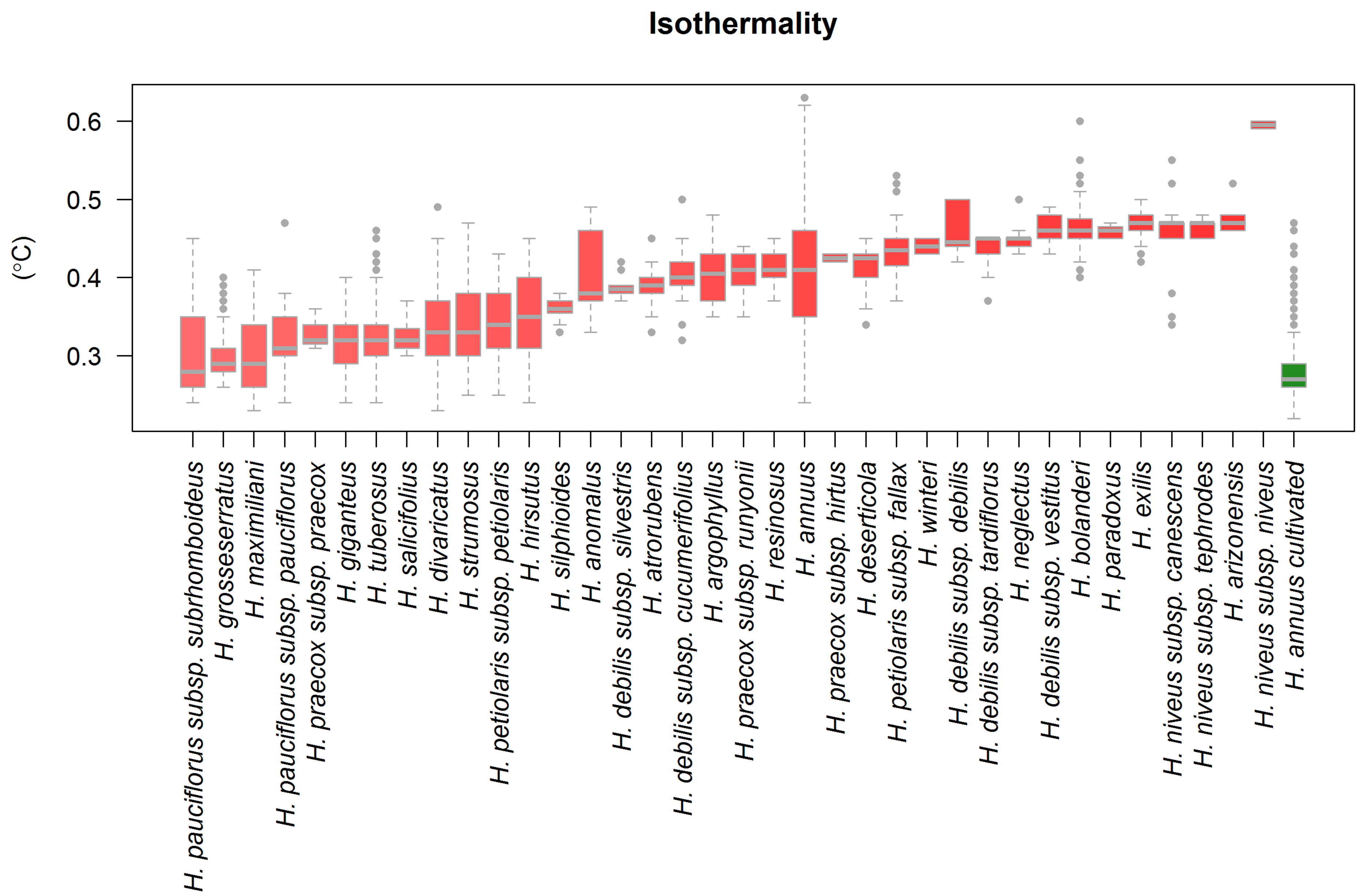
Isothermality
(°C)
0.6
0.5
0.4
0.3
H. pauciflorus subsp. subrhomboideus
H. grosseserratus
H. maximiliani
H. pauciflorus subsp. pauciflorus
H. praecox subsp. praecox
H. giganteus
H. tuberosus
H. salicifolius
H. divaricatus
H. strumosus
H. petiolaris subsp. petiolaris
H. hirsutus
H. silphioides
H. anomalus
H. debilis subsp. silvestris
H. atrorubens
H. debilis subsp. cucumerifolius
H. argophyllus
H. praecox subsp. runyonii
H. resinosus
H. annuus
H. praecox subsp. hirtus
H. deserticola
H. petiolaris subsp. fallax
H. winteri
H. debilis subsp. debilis
H. debilis subsp. tardiflorus
H. neglectus
H. debilis subsp. vestitus
H. bolanderi
H. paradoxus
H. exilis
H. niveus subsp. canescens
H. niveus subsp. tephrodes
H. arizonensis
H. niveus subsp. niveus
H. annuus cultivated

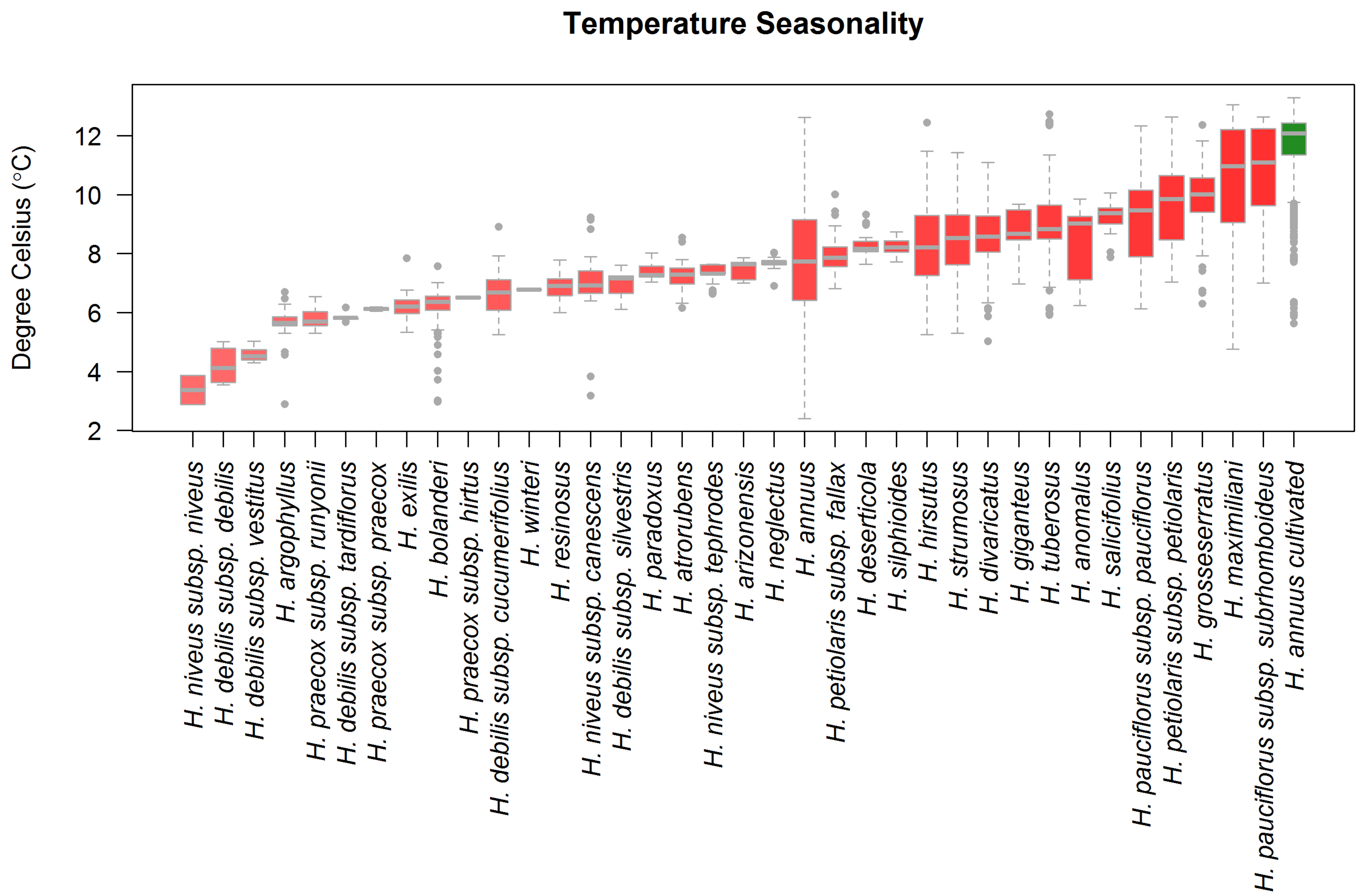
Temperature Seasonality
Degree Celsius (°C)
2
4
6
8
10
12
H. niveus subsp. niveus
H. debilis subsp. debilis
H. debilis subsp. vestitus
H. argophyllus
H. praecox subsp. runyonii
H. debilis subsp. tardiflorus
H. praecox subsp. praecox
H. exilis
H. bolanderi
H. praecox subsp. hirtus
H. debilis subsp. cucumerifolius
H. winteri
H. resinosus
H. niveus subsp. canescens
H. debilis subsp. silvestris
H. paradoxus
H. atrorubens
H. niveus subsp. tephrodes
H. arizonensis
H. neglectus
H. annuus
H. petiolaris subsp. fallax
H. deserticola
H. silphioides
H. hirsutus
H. strumosus
H. divaricatus
H. giganteus
H. tuberosus
H. anomalus
H. salicifolius
H. pauciflorus subsp. pauciflorus
H. petiolaris subsp. petiolaris
H. grosseserratus
H. maximiliani
H. pauciflorus subsp. subrhomboideus
H. annuus cultivated

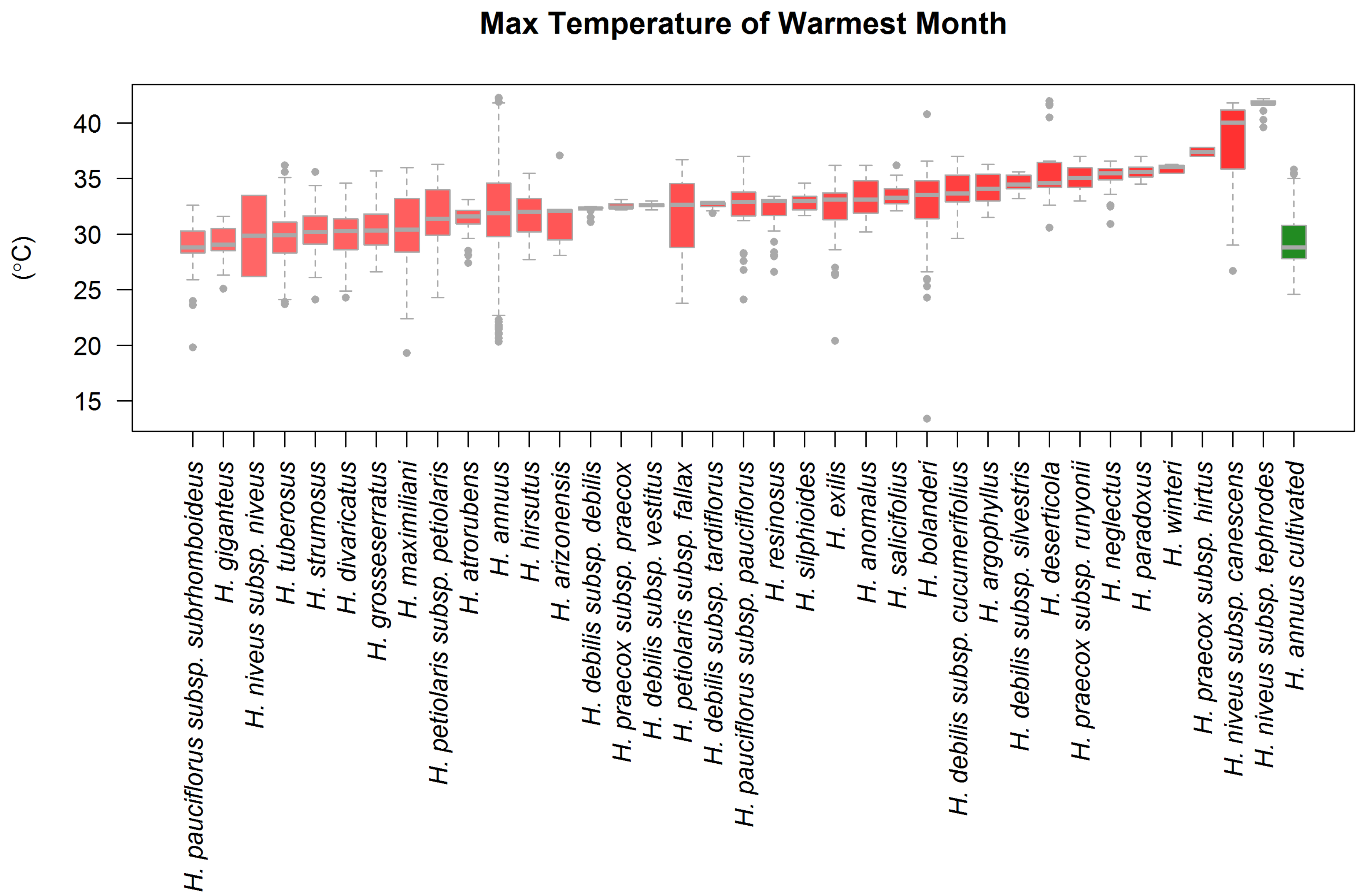

Max Temperature of Warmest Month
(°C)
40
35
30
25
20
15
H. pauciflorus subsp. subrhomboideus
H. giganteus
H. niveus subsp. niveus
H. tuberosus
H. strumosus
H. divaricatus
H. grosseserratus
H. maximiliani
H. petiolaris subsp. petiolaris
H. atrorubens
H. annuus
H. hirsutus
H. arizonensis
H. debilis subsp. debilis
H. praecox subsp. praecox
H. debilis subsp. vestitus
H. petiolaris subsp. fallax
H. debilis subsp. tardiflorus
H. pauciflorus subsp. pauciflorus
H. resinosus
H. silphioides
H. exilis
H. anomalus
H. salicifolius
H. bolanderi
H. debilis subsp. cucumerifolius
H. argophyllus
H. debilis subsp. silvestris
H. deserticola
H. praecox subsp. runyonii
H. neglectus
H. paradoxus
H. winteri
H. praecox subsp. hirtus
H. niveus subsp. canescens
H. niveus subsp. tephrodes
H. annuus cultivated

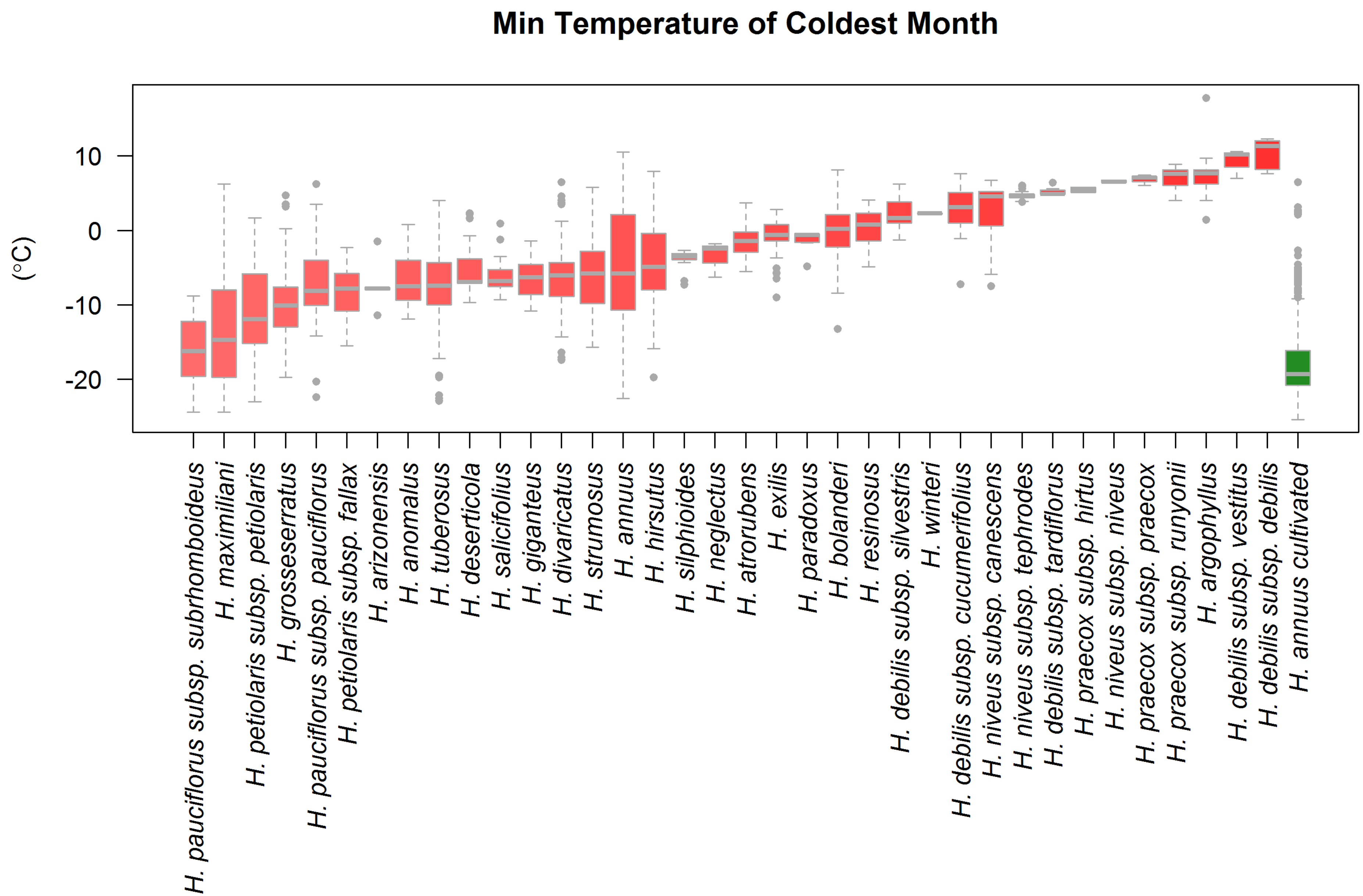

Min Temperature of Coldest Month
(°C)
10
0
-10
-20
H. pauciflorus subsp. subrhomboideus
H. maximiliani
H. petiolaris subsp. petiolaris
H. grosseserratus
H. pauciflorus subsp. pauciflorus
H. petiolaris subsp. fallax
H. arizonensis
H. anomalus
H. tuberosus
H. deserticola
H. salicifolius
H. giganteus
H. divaricatus
H. strumosus
H. annuus
H. hirsutus
H. silphioides
H. neglectus
H. atrorubens
H. exilis
H. paradoxus
H. bolanderi
H. resinosus
H. debilis subsp. silvestris
H. winteri
H. debilis subsp. cucumerifolius
H. niveus subsp. canescens
H. niveus subsp. tephrodes
H. debilis subsp. tardiflorus
H. praecox subsp. hirtus
H. niveus subsp. niveus
H. praecox subsp. praecox
H. praecox subsp. runyonii
H. argophyllus
H. debilis subsp. vestitus
H. debilis subsp. debilis
H. annuus cultivated

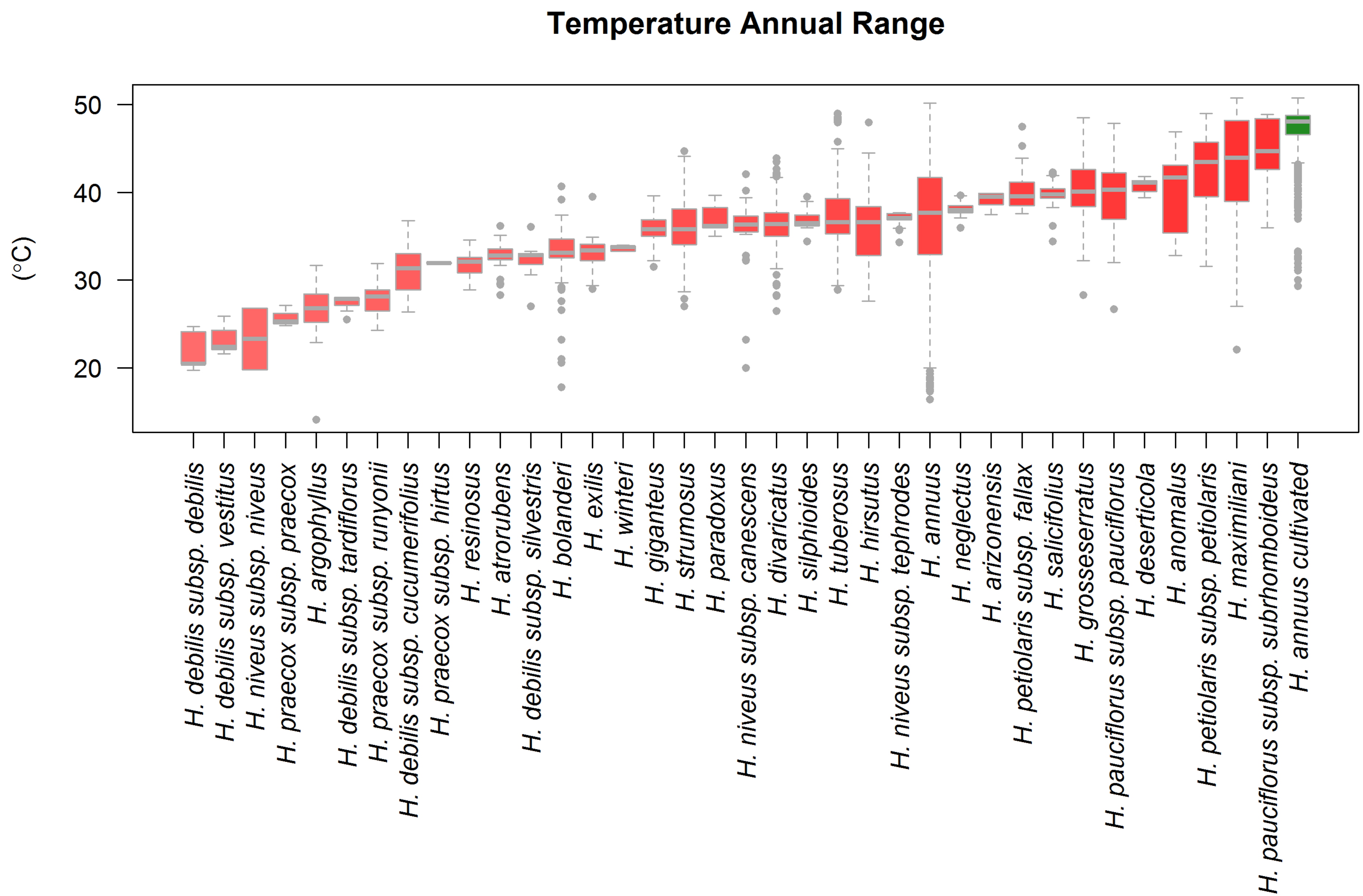

Temperature Annual Range
(°C)
50
40
30
20
H. debilis subsp. debilis
H. debilis subsp. vestitus
H. niveus subsp. niveus
H. praecox subsp. praecox
H. argophyllus
H. debilis subsp. tardiflorus
H. praecox subsp. runyonii
H. debilis subsp. cucumerifolius
H. praecox subsp. hirtus
H. resinosus
H. atrorubens
H. debilis subsp. silvestris
H. bolanderi
H. exilis
H. winteri
H. giganteus
H. strumosus
H. paradoxus
H. niveus subsp. canescens
H. divaricatus
H. silphioides
H. tuberosus
H. hirsutus
H. niveus subsp. tephrodes
H. annuus
H. neglectus
H. arizonensis
H. petiolaris subsp. fallax
H. salicifolius
H. grosseserratus
H. pauciflorus subsp. pauciflorus
H. deserticola
H. anomalus
H. petiolaris subsp. petiolaris
H. maximiliani
H. pauciflorus subsp. subrhomboideus
H. annuus cultivated

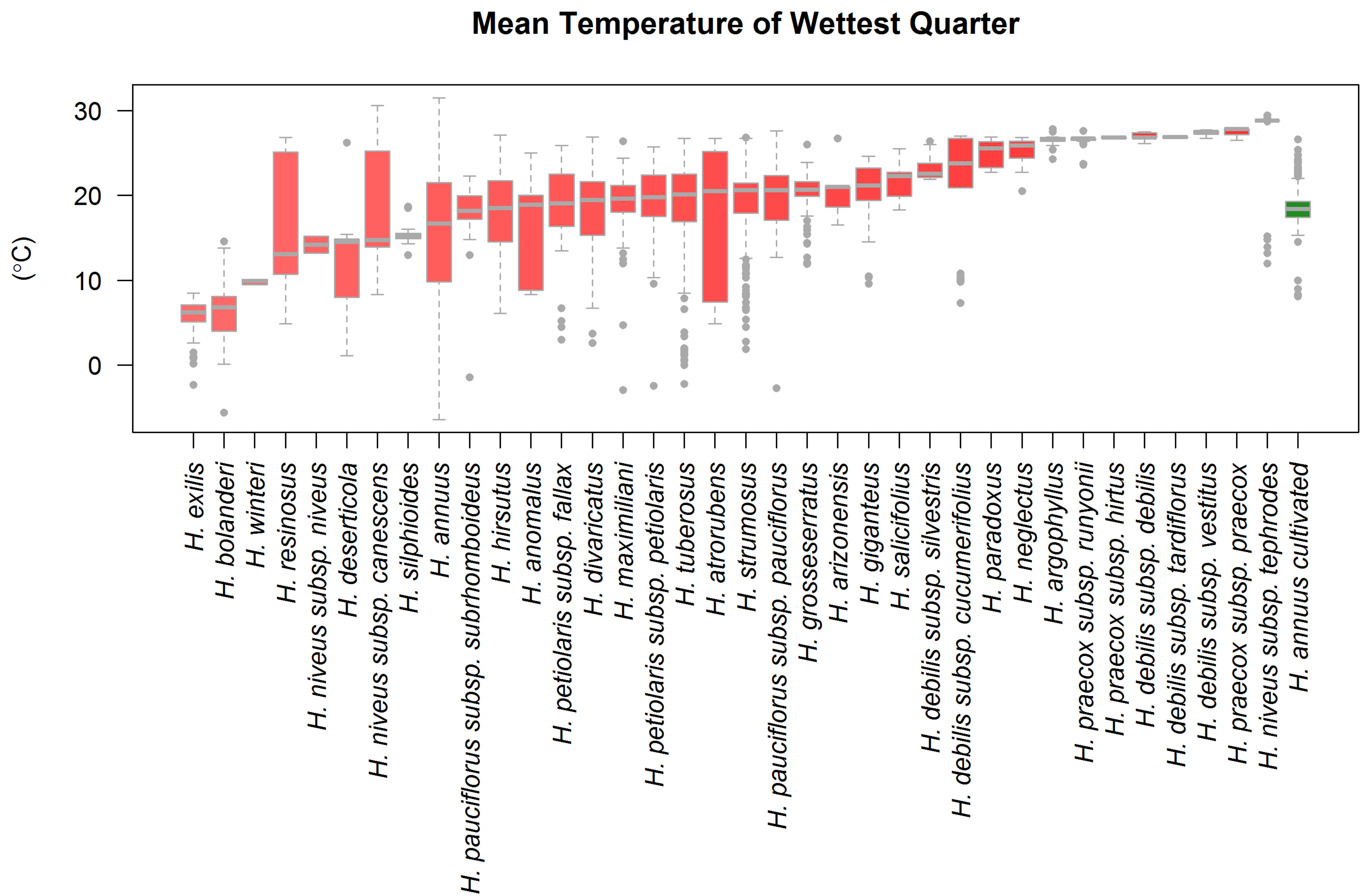
Mean Temperature of Wettest Quarter
(°C)
30
20
10
0
H. exilis
H. bolanderi
H. winteri
H. resinosus
H. niveus subsp. niveus
H. deserticola
H. niveus subsp. canescens
H. silphioides
H. annuus
H. pauciflorus subsp. subrhomboideus
H. hirsutus
H. anomalus
H. petiolaris subsp. fallax
H. divaricatus
H. maximiliani
H. petiolaris subsp. petiolaris
H. tuberosus
H. atrorubens
H. strumosus
H. pauciflorus subsp. pauciflorus
H. grosseserratus
H. arizonensis
H. giganteus
H. salicifolius
H. debilis subsp. silvestris
H. debilis subsp. cucumerifolius
H. paradoxus
H. neglectus
H. argophyllus
H. praecox subsp. runyonii
H. praecox subsp. hirtus
H. debilis subsp. debilis
H. debilis subsp. tardiflorus
H. debilis subsp. vestitus
H. praecox subsp. praecox
H. niveus subsp. tephrodes
H. annuus cultivated

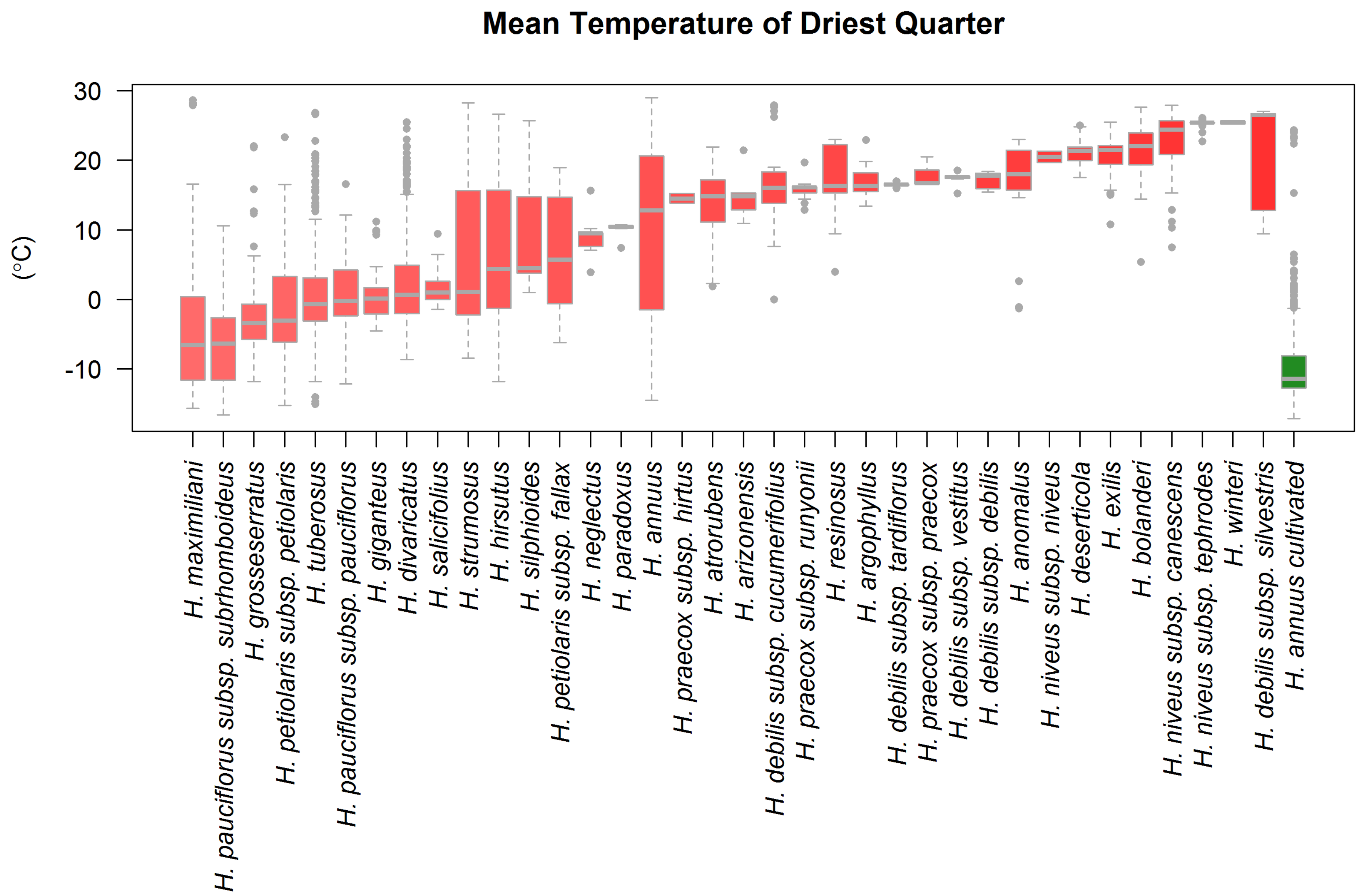
Mean Temperature of Driest Quarter
(°C)
30
20
10
0
-10
H. maximiliani
H. pauciflorus subsp. subrhomboideus
H. grosseserratus
H. petiolaris subsp. petiolaris
H. tuberosus
H. pauciflorus subsp. pauciflorus
H. giganteus
H. divaricatus
H. salicifolius
H. strumosus
H. hirsutus
H. silphioides
H. petiolaris subsp. fallax
H. neglectus
H. paradoxus
H. annuus
H. praecox subsp. hirtus
H. atrorubens
H. arizonensis
H. debilis subsp. cucumerifolius
H. praecox subsp. runyonii
H. resinosus
H. argophyllus
H. debilis subsp. tardiflorus
H. praecox subsp. praecox
H. debilis subsp. vestitus
H. debilis subsp. debilis
H. anomalus
H. niveus subsp. niveus
H. deserticola
H. exilis
H. bolanderi
H. niveus subsp. canescens
H. niveus subsp. tephrodes
H. winteri
H. debilis subsp. silvestris
H. annuus cultivated

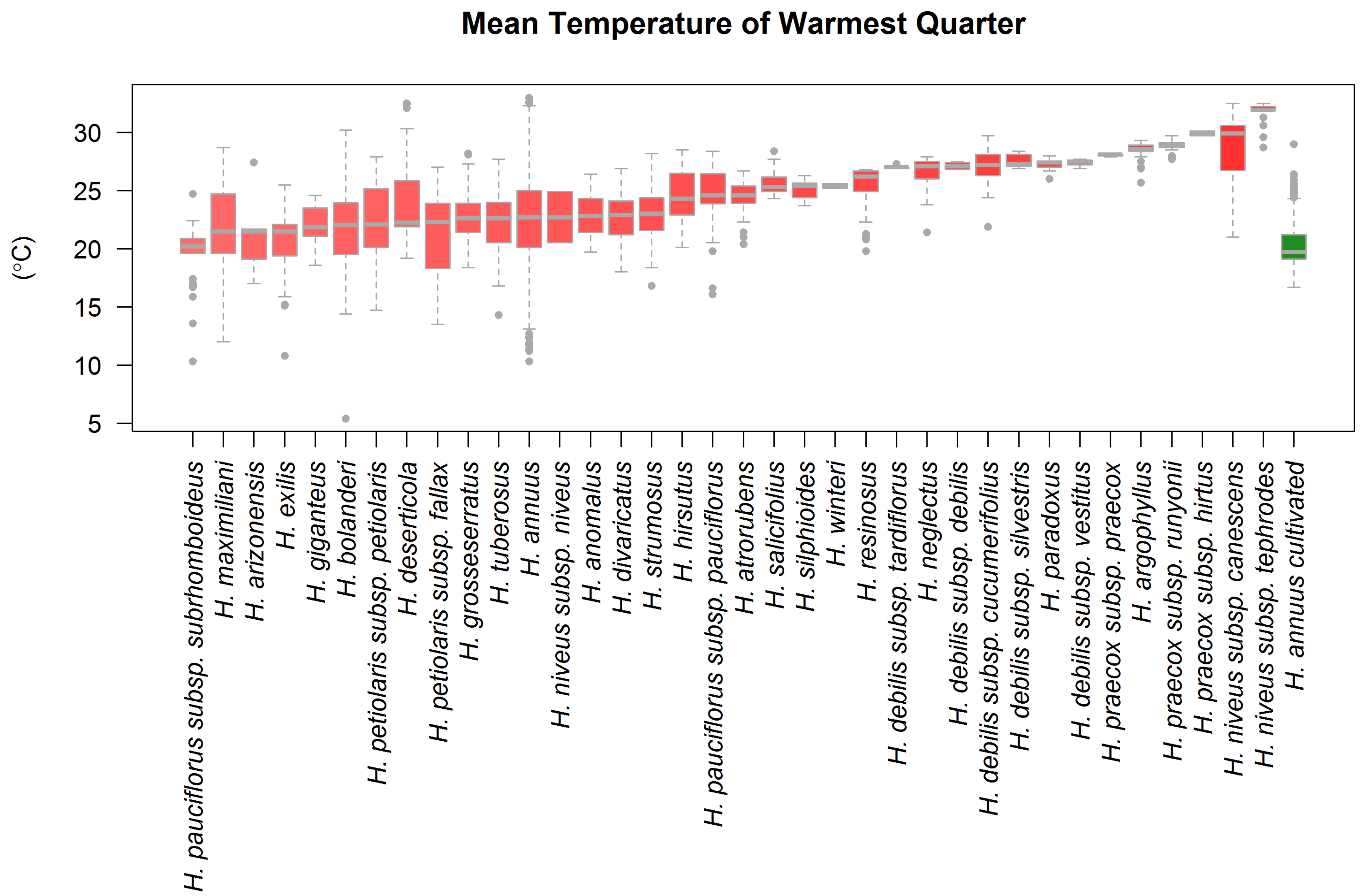

Mean Temperature of Warmest Quarter
(°C)
5
10
15
20
25
30
H. pauciflorus subsp. subrhomboideus
H. maximiliani
H. arizonensis
H. exilis
H. giganteus
H. bolanderi
H. petiolaris subsp. petiolaris
H. deserticola
H. petiolaris subsp. fallax
H. grosseserratus
H. tuberosus
H. annuus
H. niveus subsp. niveus
H. anomalus
H. divaricatus
H. strumosus
H. hirsutus
H. pauciflorus subsp. pauciflorus
H. atrorubens
H. salicifolius
H. silphioides
H. winteri
H. resinosus
H. debilis subsp. tardiflorus
H. neglectus
H. debilis subsp. debilis
H. debilis subsp. cucumerifolius
H. debilis subsp. silvestris
H. paradoxus
H. debilis subsp. vestitus
H. praecox subsp. praecox
H. argophyllus
H. praecox subsp. runyonii
H. praecox subsp. hirtus
H. niveus subsp. canescens
H. niveus subsp. tephrodes
H. annuus cultivated

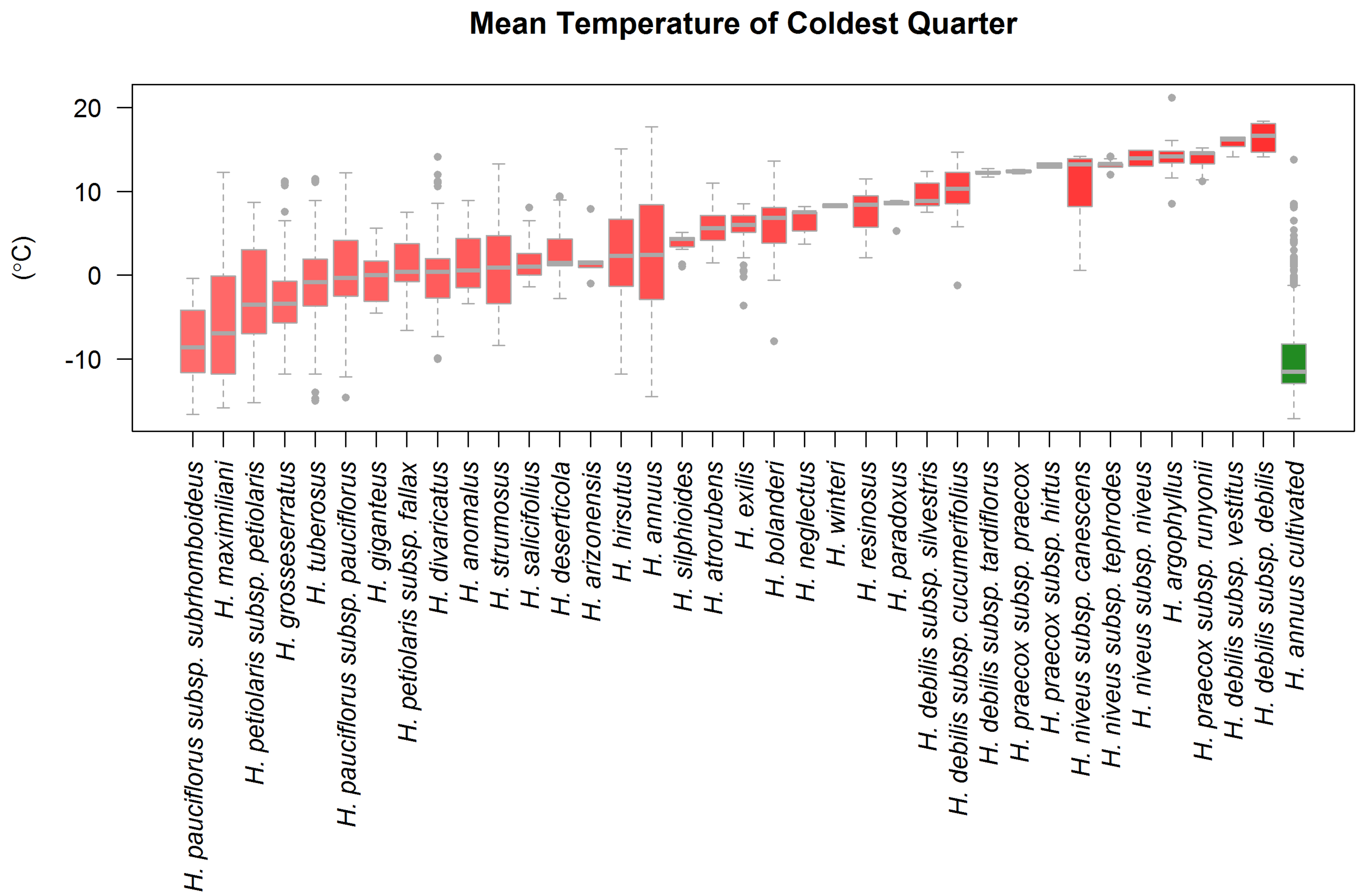

Mean Temperature of Coldest Quarter
(°C)
20
10
0
-10
H. pauciflorus subsp. subrhomboideus
H. maximiliani
H. petiolaris subsp. petiolaris
H. grosseserratus
H. tuberosus
H. pauciflorus subsp. pauciflorus
H. giganteus
H. petiolaris subsp. fallax
H. divaricatus
H. anomalus
H. strumosus
H. salicifolius
H. deserticola
H. arizonensis
H. hirsutus
H. annuus
H. silphioides
H. atrorubens
H. exilis
H. bolanderi
H. neglectus
H. winteri
H. resinosus
H. paradoxus
H. debilis subsp. silvestris
H. debilis subsp. cucumerifolius
H. debilis subsp. tardiflorus
H. praecox subsp. praecox
H. praecox subsp. hirtus
H. niveus subsp. canescens
H. niveus subsp. tephrodes
H. niveus subsp. niveus
H. argophyllus
H. praecox subsp. runyonii
H. debilis subsp. vestitus
H. debilis subsp. debilis
H. annuus cultivated

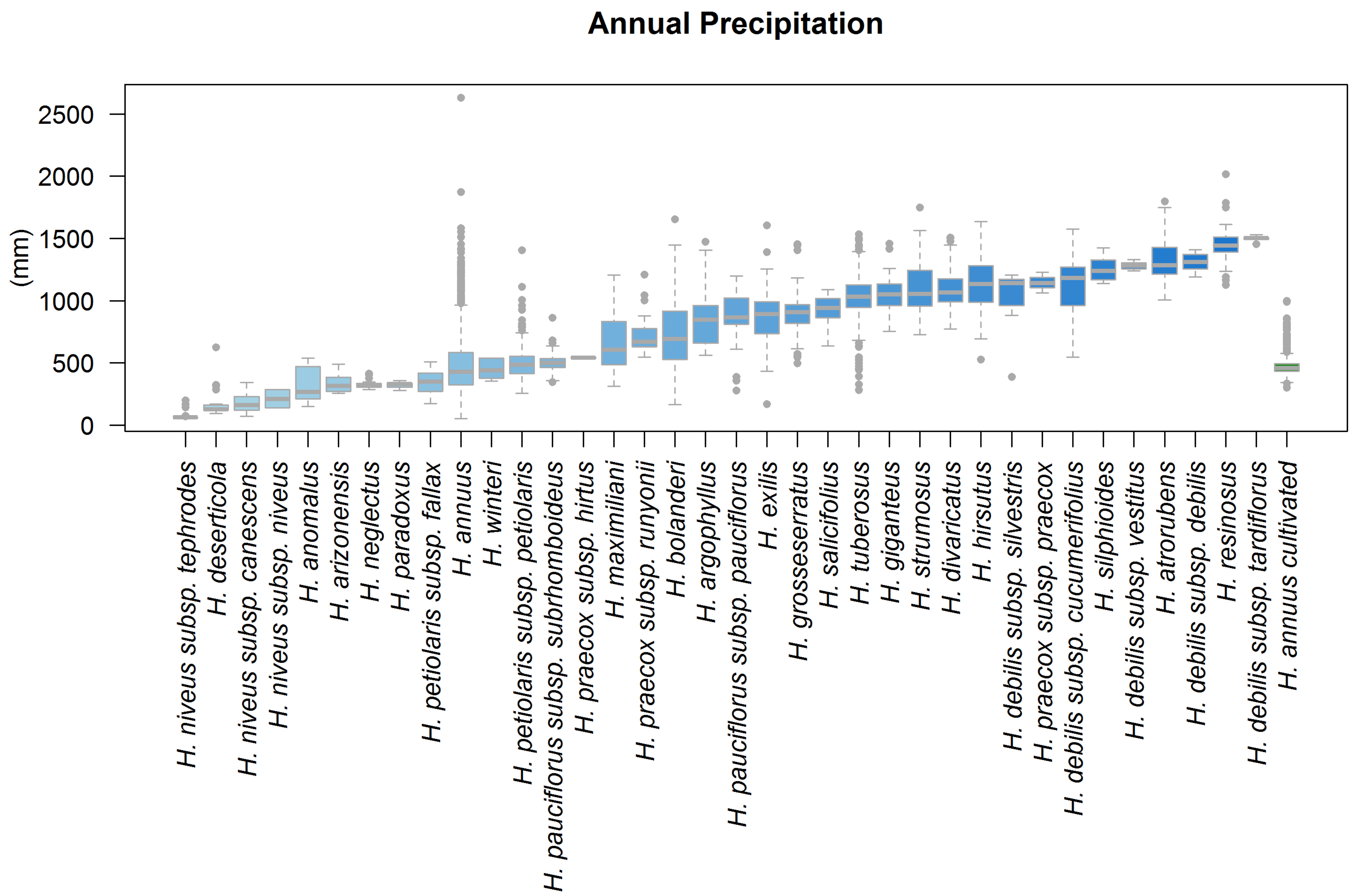

Annual Precipitation
(mm)
0
500
1000
1500
2000
2500
H. niveus subsp. tephrodes
H. deserticola
H. niveus subsp. canescens
H. niveus subsp. niveus
H. anomalus
H. arizonensis
H. neglectus
H. paradoxus
H. petiolaris subsp. fallax
H. annuus
H. winteri
H. petiolaris subsp. petiolaris
H. pauciflorus subsp. subrhomboideus
H. praecox subsp. hirtus
H. maximiliani
H. praecox subsp. runyonii
H. bolanderi
H. argophyllus
H. pauciflorus subsp. pauciflorus
H. exilis
H. grosseserratus
H. salicifolius
H. tuberosus
H. giganteus
H. strumosus
H. divaricatus
H. hirsutus
H. debilis subsp. silvestris
H. praecox subsp. praecox
H. debilis subsp. cucumerifolius
H. silphioides
H. debilis subsp. vestitus
H. atrorubens
H. debilis subsp. debilis
H. resinosus
H. debilis subsp. tardiflorus
H. annuus cultivated

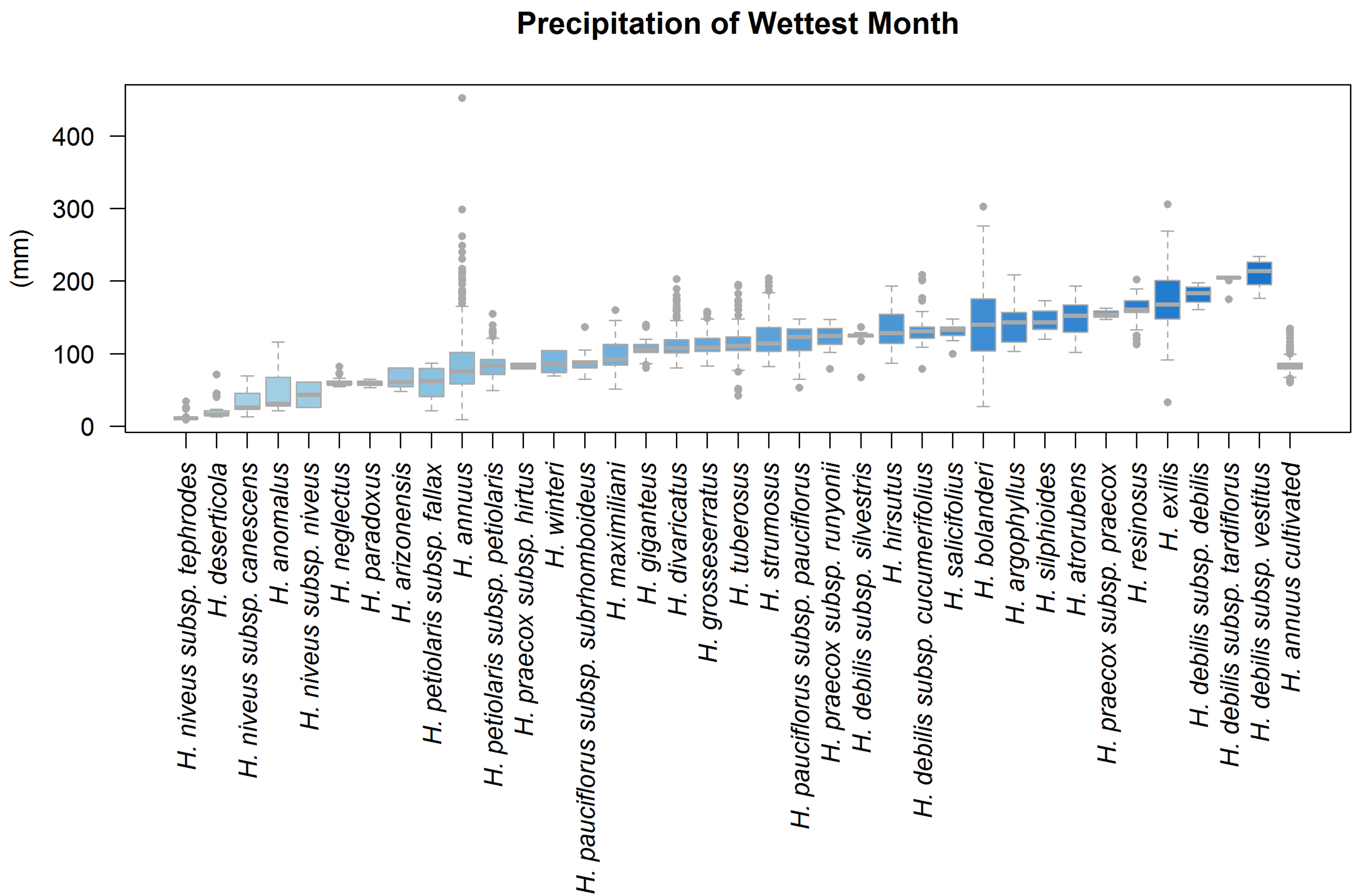
Precipitation of Wettest Month
(mm)
0
100
200
300
400
H. niveus subsp. tephrodes
H. deserticola
H. niveus subsp. canescens
H. anomalus
H. niveus subsp. niveus
H. neglectus
H. paradoxus
H. arizonensis
H. petiolaris subsp. fallax
H. annuus
H. petiolaris subsp. petiolaris
H. praecox subsp. hirtus
H. winteri
H. pauciflorus subsp. subrhomboideus
H. maximiliani
H. giganteus
H. divaricatus
H. grosseserratus
H. tuberosus
H. strumosus
H. pauciflorus subsp. pauciflorus
H. praecox subsp. runyonii
H. debilis subsp. silvestris
H. hirsutus
H. debilis subsp. cucumerifolius
H. salicifolius
H. bolanderi
H. argophyllus
H. silphioides
H. atrorubens
H. praecox subsp. praecox
H. resinosus
H. exilis
H. debilis subsp. debilis
H. debilis subsp. tardiflorus
H. debilis subsp. vestitus
H. annuus cultivated

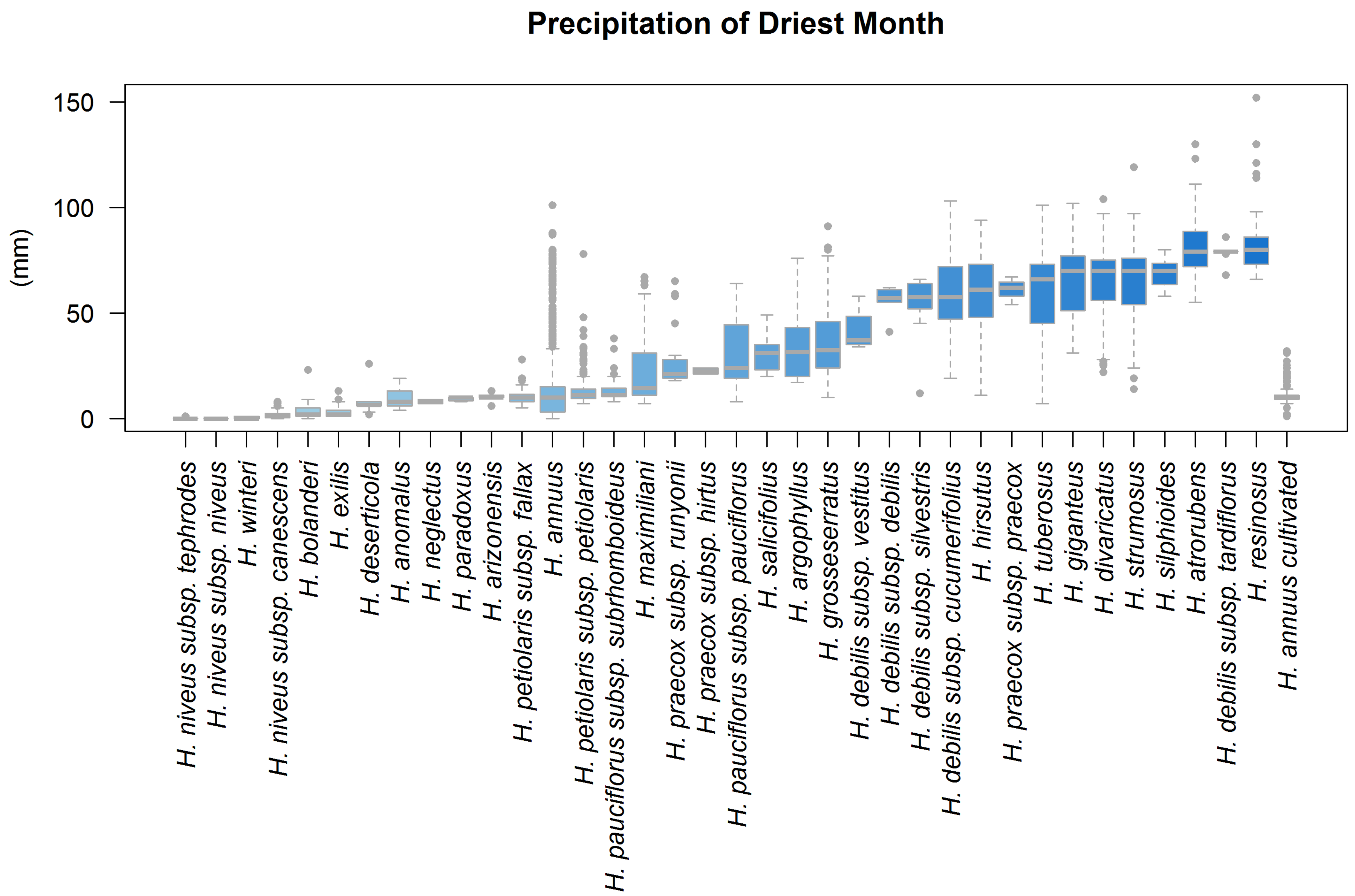
Precipitation of Driest Month
(mm)
150
100
50
0
H. niveus subsp. tephrodes
H. niveus subsp. niveus
H. winteri
H. niveus subsp. canescens
H. bolanderi
H. exilis
H. deserticola
H. anomalus
H. neglectus
H. paradoxus
H. arizonensis
H. petiolaris subsp. fallax
H. annuus
H. petiolaris subsp. petiolaris
H. pauciflorus subsp. subrhomboideus
H. maximiliani
H. praecox subsp. runyonii
H. praecox subsp. hirtus
H. pauciflorus subsp. pauciflorus
H. salicifolius
H. argophyllus
H. grosseserratus
H. debilis subsp. vestitus
H. debilis subsp. debilis
H. debilis subsp. silvestris
H. debilis subsp. cucumerifolius
H. hirsutus
H. praecox subsp. praecox
H. tuberosus
H. giganteus
H. divaricatus
H. strumosus
H. silphioides
H. atrorubens
H. debilis subsp. tardiflorus
H. resinosus
H. annuus cultivated

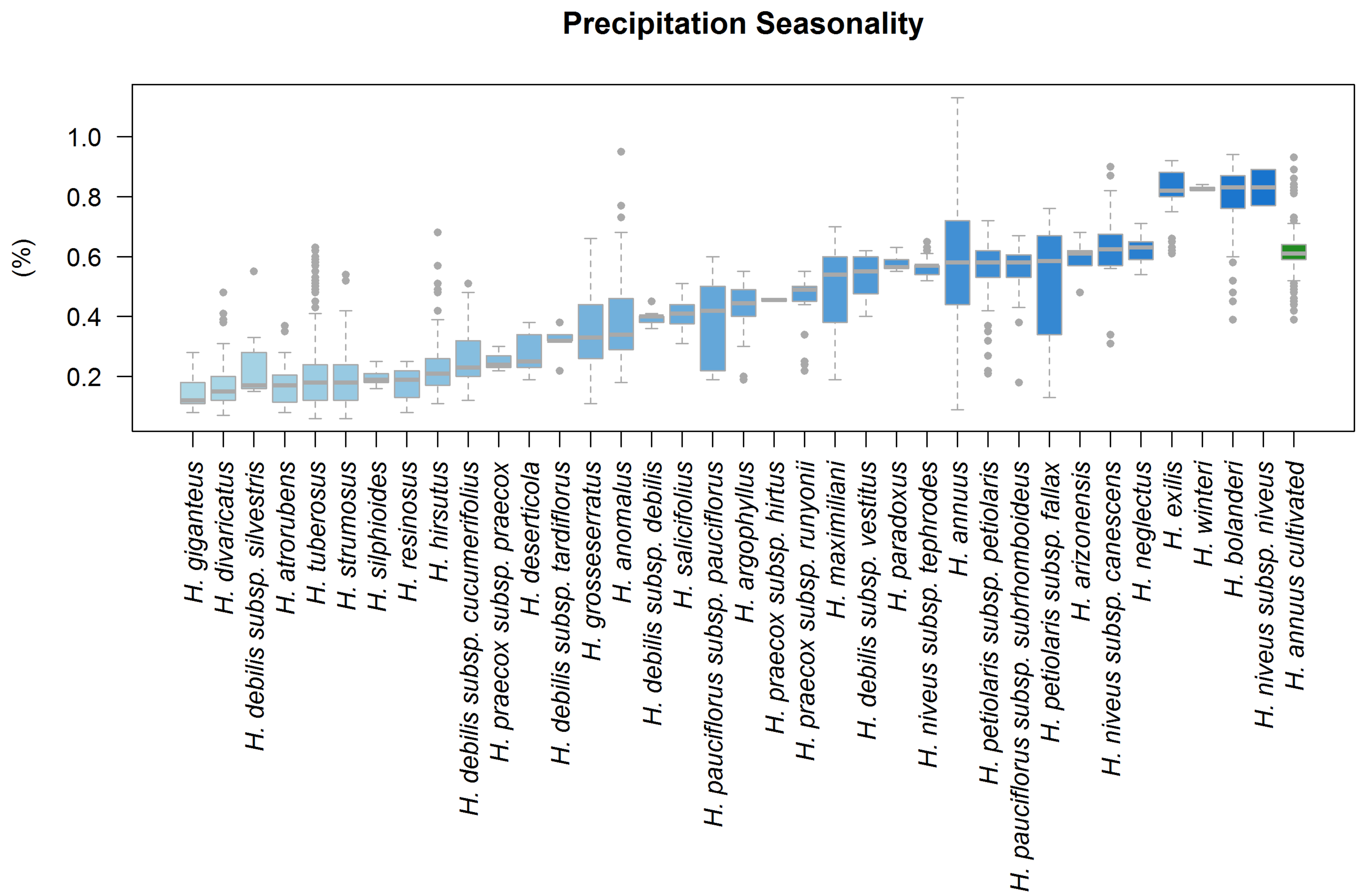
Precipitation Seasonality
(%)
1.0
0.8
0.6
0.4
0.2
H. giganteus
H. divaricatus
H. debilis subsp. silvestris
H. atrorubens
H. tuberosus
H. strumosus
H. silphioides
H. resinosus
H. hirsutus
H. debilis subsp. cucumerifolius
H. praecox subsp. praecox
H. deserticola
H. debilis subsp. tardiflorus
H. grosseserratus
H. anomalus
H. debilis subsp. debilis
H. salicifolius
H. pauciflorus subsp. pauciflorus
H. argophyllus
H. praecox subsp. hirtus
H. praecox subsp. runyonii
H. maximiliani
H. debilis subsp. vestitus
H. paradoxus
H. niveus subsp. tephrodes
H. annuus
H. petiolaris subsp. petiolaris
H. pauciflorus subsp. subrhomboideus
H. petiolaris subsp. fallax
H. arizonensis
H. niveus subsp. canescens
H. neglectus
H. exilis
H. winteri
H. bolanderi
H. niveus subsp. niveus
H. annuus cultivated

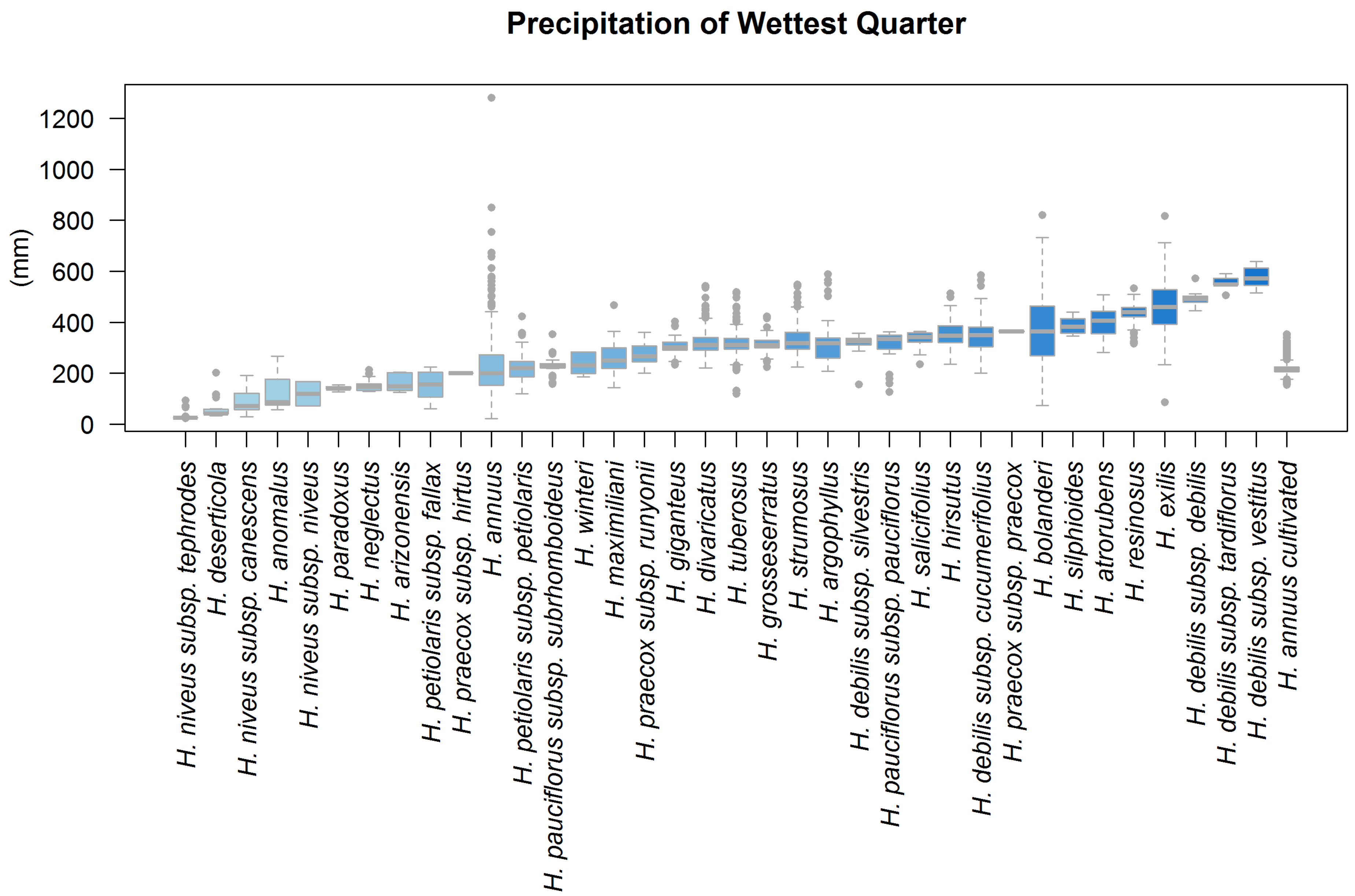

Precipitation of Wettest Quarter
(mm)
0
200
400
600
800
1000
1200
H. niveus subsp. tephrodes
H. deserticola
H. niveus subsp. canescens
H. anomalus
H. niveus subsp. niveus
H. paradoxus
H. neglectus
H. arizonensis
H. petiolaris subsp. fallax
H. praecox subsp. hirtus
H. annuus
H. petiolaris subsp. petiolaris
H. pauciflorus subsp. subrhomboideus
H. winteri
H. maximiliani
H. praecox subsp. runyonii
H. giganteus
H. divaricatus
H. tuberosus
H. grosseserratus
H. strumosus
H. argophyllus
H. debilis subsp. silvestris
H. pauciflorus subsp. pauciflorus
H. salicifolius
H. hirsutus
H. debilis subsp. cucumerifolius
H. praecox subsp. praecox
H. bolanderi
H. silphioides
H. atrorubens
H. resinosus
H. exilis
H. debilis subsp. debilis
H. debilis subsp. tardiflorus
H. debilis subsp. vestitus
H. annuus cultivated

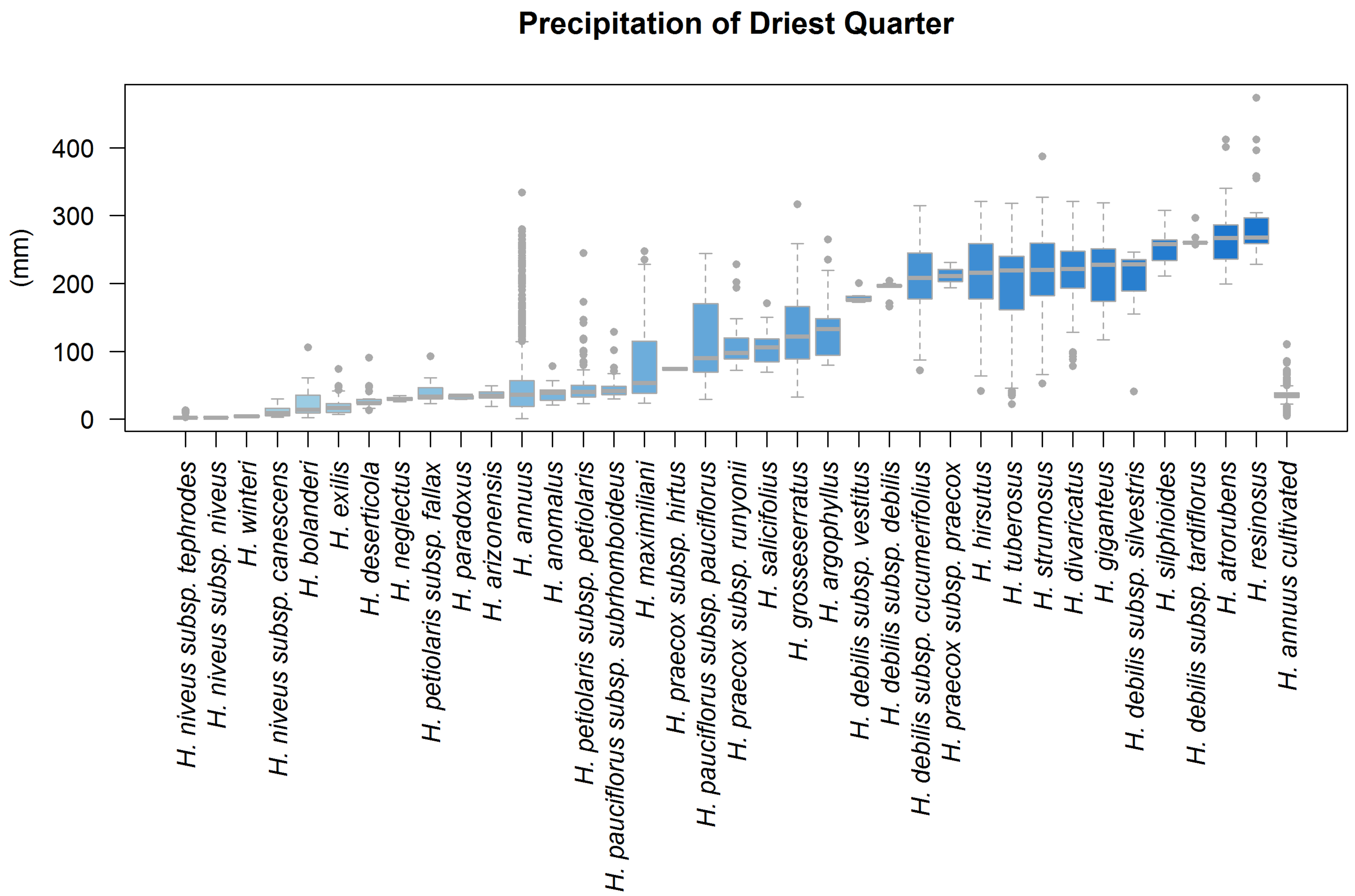
Precipitation of Driest Quarter
(mm)
0
100
200
300
400
H. niveus subsp. tephrodes
H. niveus subsp. niveus
H. winteri
H. niveus subsp. canescens
H. bolanderi
H. exilis
H. deserticola
H. neglectus
H. petiolaris subsp. fallax
H. paradoxus
H. arizonensis
H. annuus
H. anomalus
H. petiolaris subsp. petiolaris
H. pauciflorus subsp. subrhomboideus
H. maximiliani
H. praecox subsp. hirtus
H. pauciflorus subsp. pauciflorus
H. praecox subsp. runyonii
H. salicifolius
H. grosseserratus
H. argophyllus
H. debilis subsp. vestitus
H. debilis subsp. debilis
H. debilis subsp. cucumerifolius
H. praecox subsp. praecox
H. hirsutus
H. tuberosus
H. strumosus
H. divaricatus
H. giganteus
H. debilis subsp. silvestris
H. silphioides
H. debilis subsp. tardiflorus
H. atrorubens
H. resinosus
H. annuus cultivated

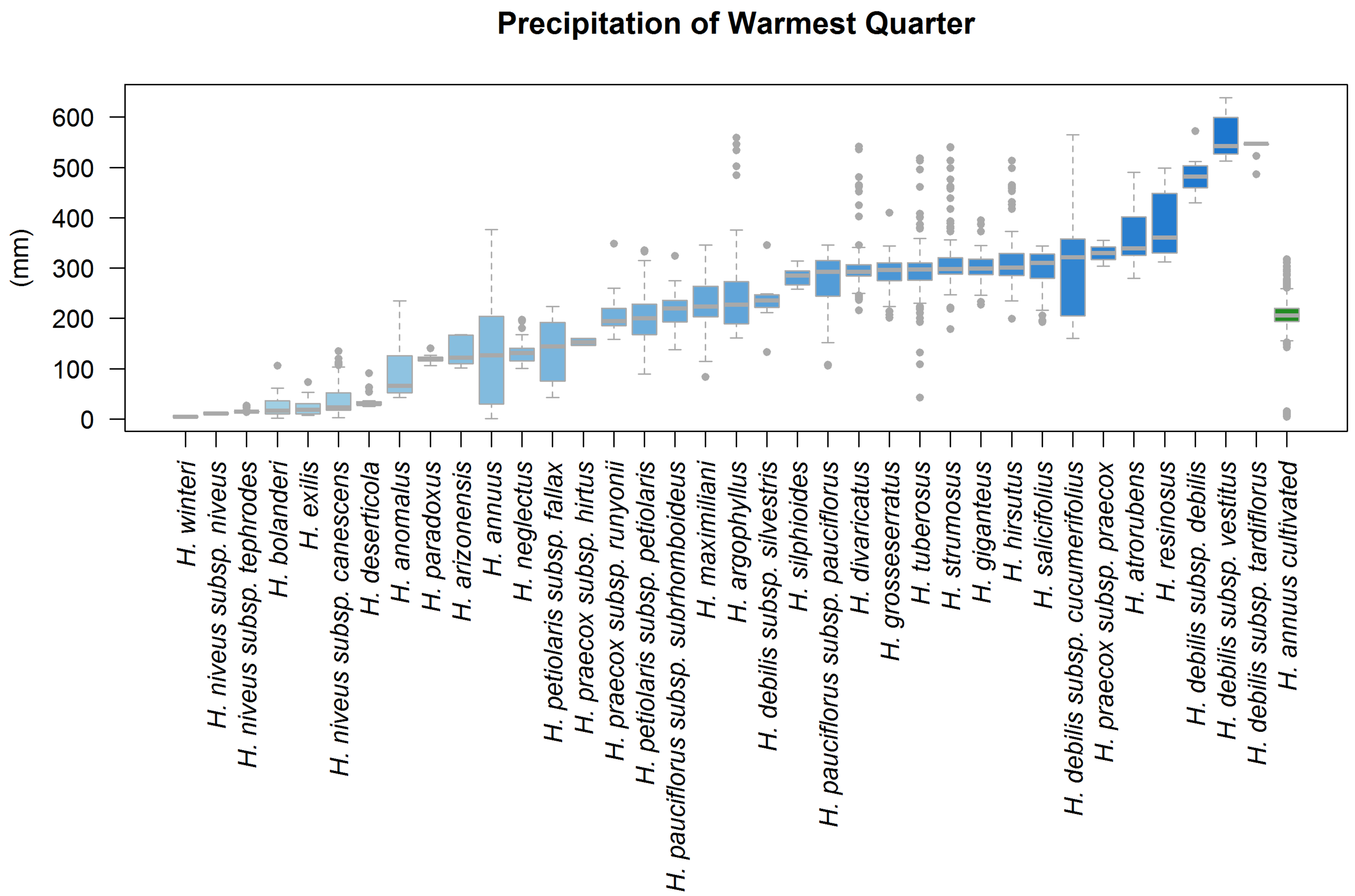

Precipitation of Warmest Quarter
(mm)
0
100
200
300
400
500
600
H. winteri
H. niveus subsp. niveus
H. niveus subsp. tephrodes
H. bolanderi
H. exilis
H. niveus subsp. canescens
H. deserticola
H. anomalus
H. paradoxus
H. arizonensis
H. annuus
H. neglectus
H. petiolaris subsp. fallax
H. praecox subsp. hirtus
H. praecox subsp. runyonii
H. petiolaris subsp. petiolaris
H. pauciflorus subsp. subrhomboideus
H. maximiliani
H. argophyllus
H. debilis subsp. silvestris
H. silphioides
H. pauciflorus subsp. pauciflorus
H. divaricatus
H. grosseserratus
H. tuberosus
H. strumosus
H. giganteus
H. hirsutus
H. salicifolius
H. debilis subsp. cucumerifolius
H. praecox subsp. praecox
H. atrorubens
H. resinosus
H. debilis subsp. debilis
H. debilis subsp. vestitus
H. debilis subsp. tardiflorus
H. annuus cultivated

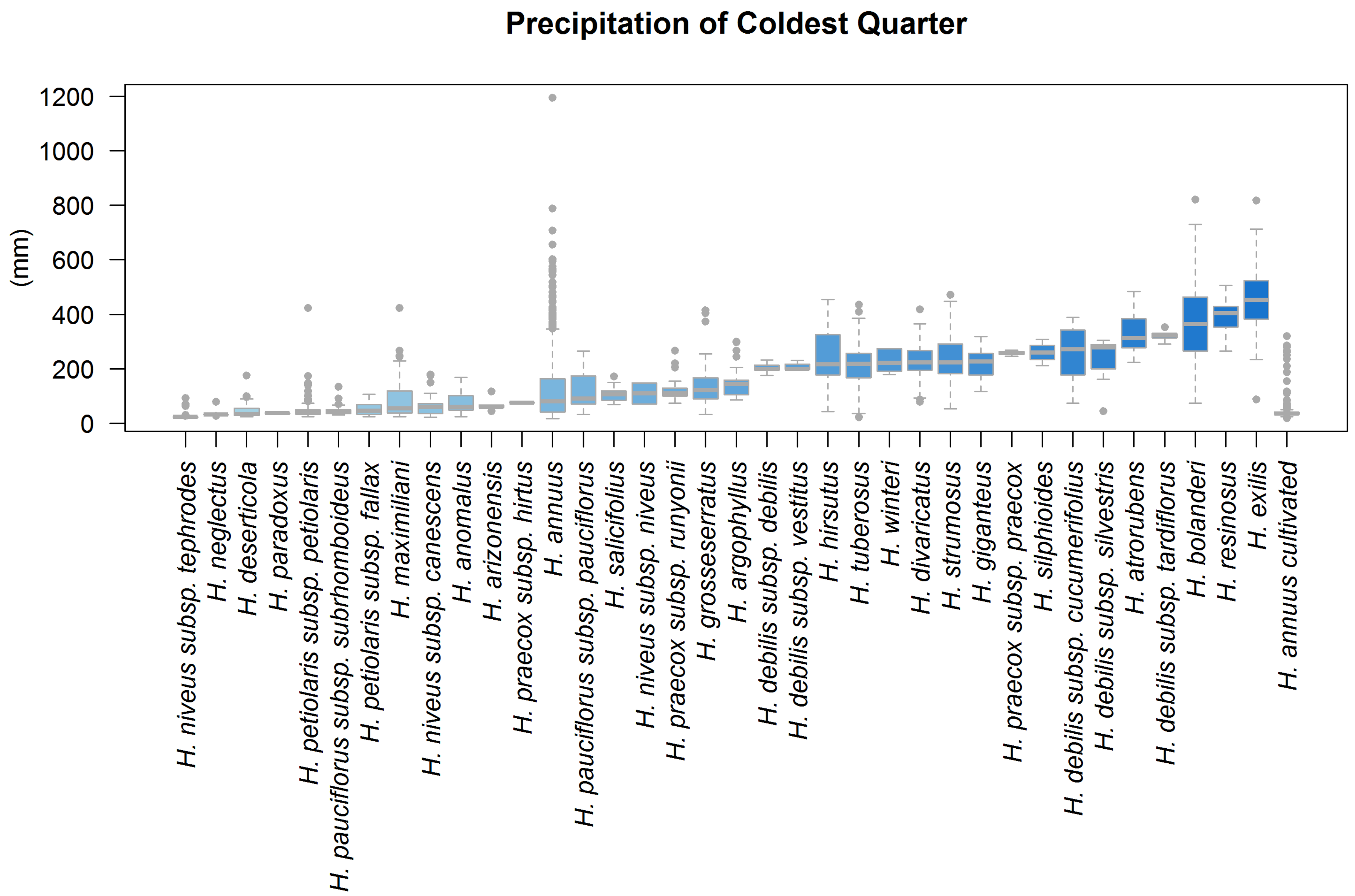
Precipitation of Coldest Quarter
(mm)
1200
1000
800
600
400
200
0
H. niveus subsp. tephrodes
H. neglectus
H. deserticola
H. paradoxus
H. petiolaris subsp. petiolaris
H. pauciflorus subsp. subrhomboideus
H. petiolaris subsp. fallax
H. maximiliani
H. niveus subsp. canescens
H. anomalus
H. arizonensis
H. praecox subsp. hirtus
H. annuus
H. pauciflorus subsp. pauciflorus
H. salicifolius
H. niveus subsp. niveus
H. praecox subsp. runyonii
H. grosseserratus
H. argophyllus
H. debilis subsp. debilis
H. debilis subsp. vestitus
H. hirsutus
H. tuberosus
H. winteri
H. divaricatus
H. strumosus
H. giganteus
H. praecox subsp. praecox
H. silphioides
H. debilis subsp. cucumerifolius
H. debilis subsp. silvestris
H. atrorubens
H. debilis subsp. tardiflorus
H. bolanderi
H. resinosus
H. exilis
H. annuus cultivated

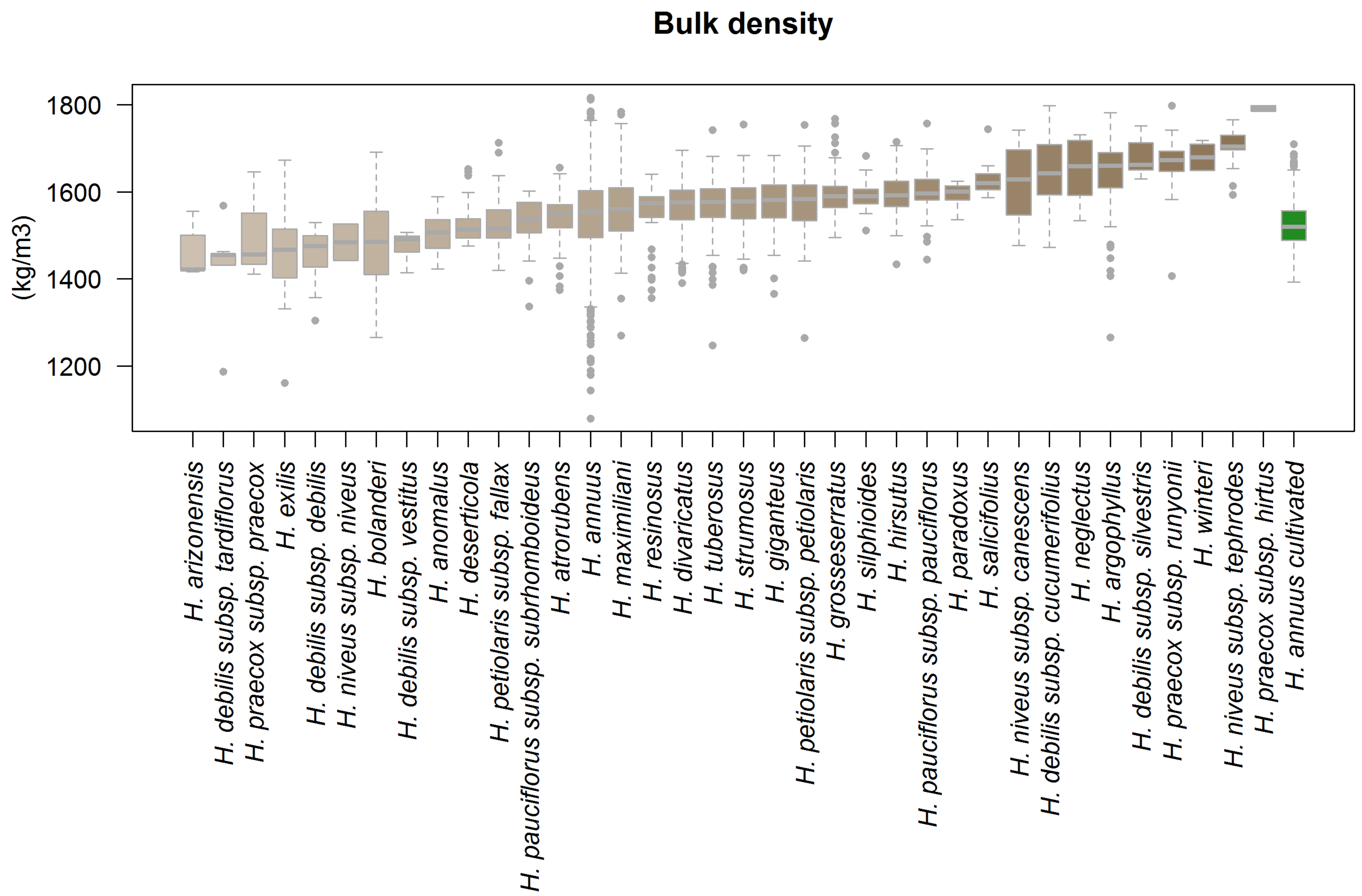
Bulk density
(kg/m3)
1800
1600
1400
1200
H. arizonensis
H. debilis subsp. tardiflorus
H. praecox subsp. praecox
H. exilis
H. debilis subsp. debilis
H. niveus subsp. niveus
H. bolanderi
H. debilis subsp. vestitus
H. anomalus
H. deserticola
H. petiolaris subsp. fallax
H. pauciflorus subsp. subrhomboideus
H. atrorubens
H. annuus
H. maximiliani
H. resinosus
H. divaricatus
H. tuberosus
H. strumosus
H. giganteus
H. petiolaris subsp. petiolaris
H. grosseserratus
H. silphioides
H. hirsutus
H. pauciflorus subsp. pauciflorus
H. paradoxus
H. salicifolius
H. niveus subsp. canescens
H. debilis subsp. cucumerifolius
H. neglectus
H. argophyllus
H. debilis subsp. silvestris
H. praecox subsp. runyonii
H. winteri
H. niveus subsp. tephrodes
H. praecox subsp. hirtus
H. annuus cultivated

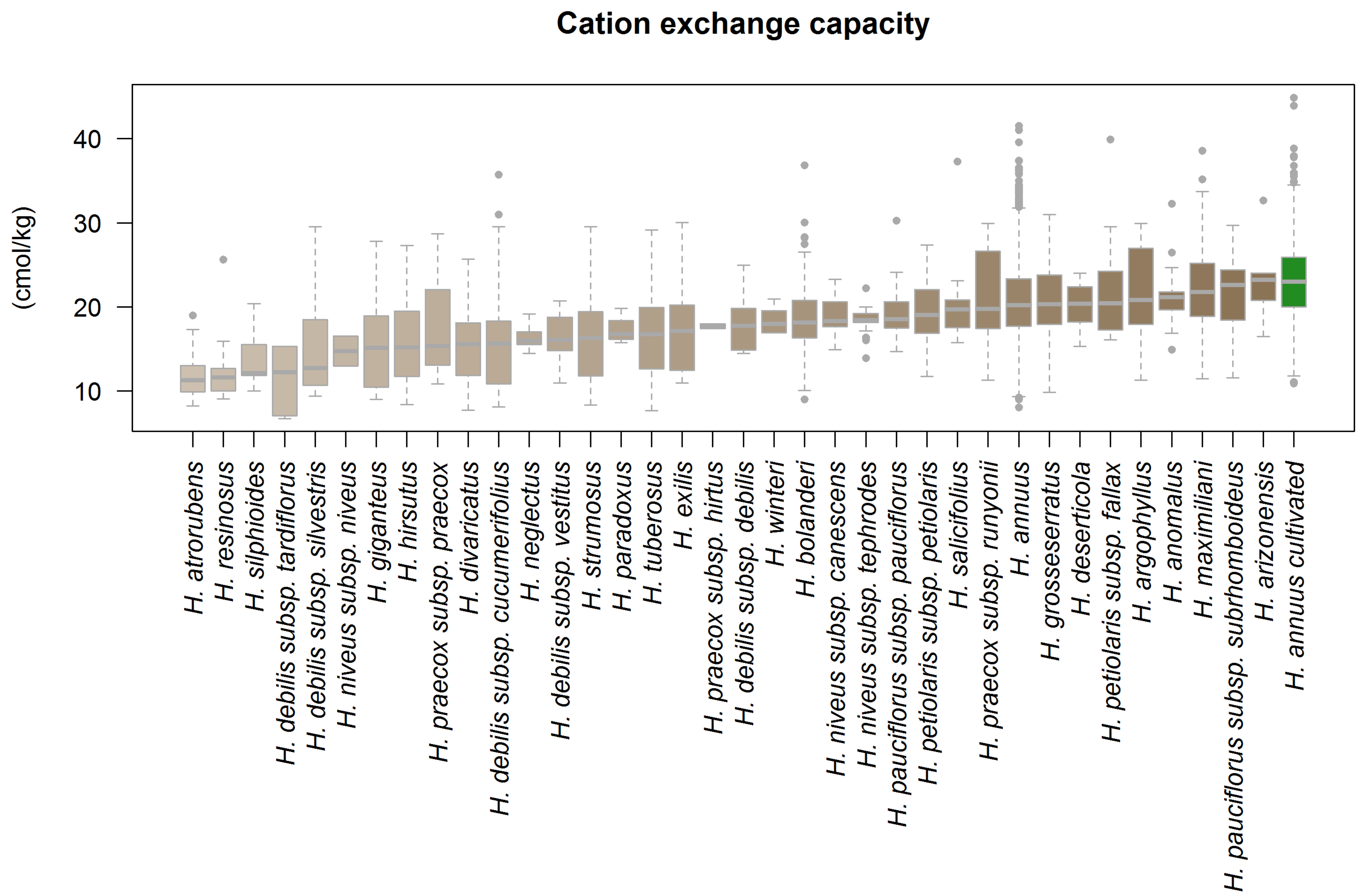

Cation exchange capacity
(cmol/kg)
10
20
30
40
H. atrorubens
H. resinosus
H. silphioides
H. debilis subsp. tardiflorus
H. debilis subsp. silvestris
H. niveus subsp. niveus
H. giganteus
H. hirsutus
H. praecox subsp. praecox
H. divaricatus
H. debilis subsp. cucumerifolius
H. neglectus
H. debilis subsp. vestitus
H. strumosus
H. paradoxus
H. tuberosus
H. exilis
H. praecox subsp. hirtus
H. debilis subsp. debilis
H. winteri
H. bolanderi
H. niveus subsp. canescens
H. niveus subsp. tephrodes
H. pauciflorus subsp. pauciflorus
H. petiolaris subsp. petiolaris
H. salicifolius
H. praecox subsp. runyonii
H. annuus
H. grosseserratus
H. deserticola
H. petiolaris subsp. fallax
H. argophyllus
H. anomalus
H. maximiliani
H. pauciflorus subsp. subrhomboideus
H. arizonensis
H. annuus cultivated

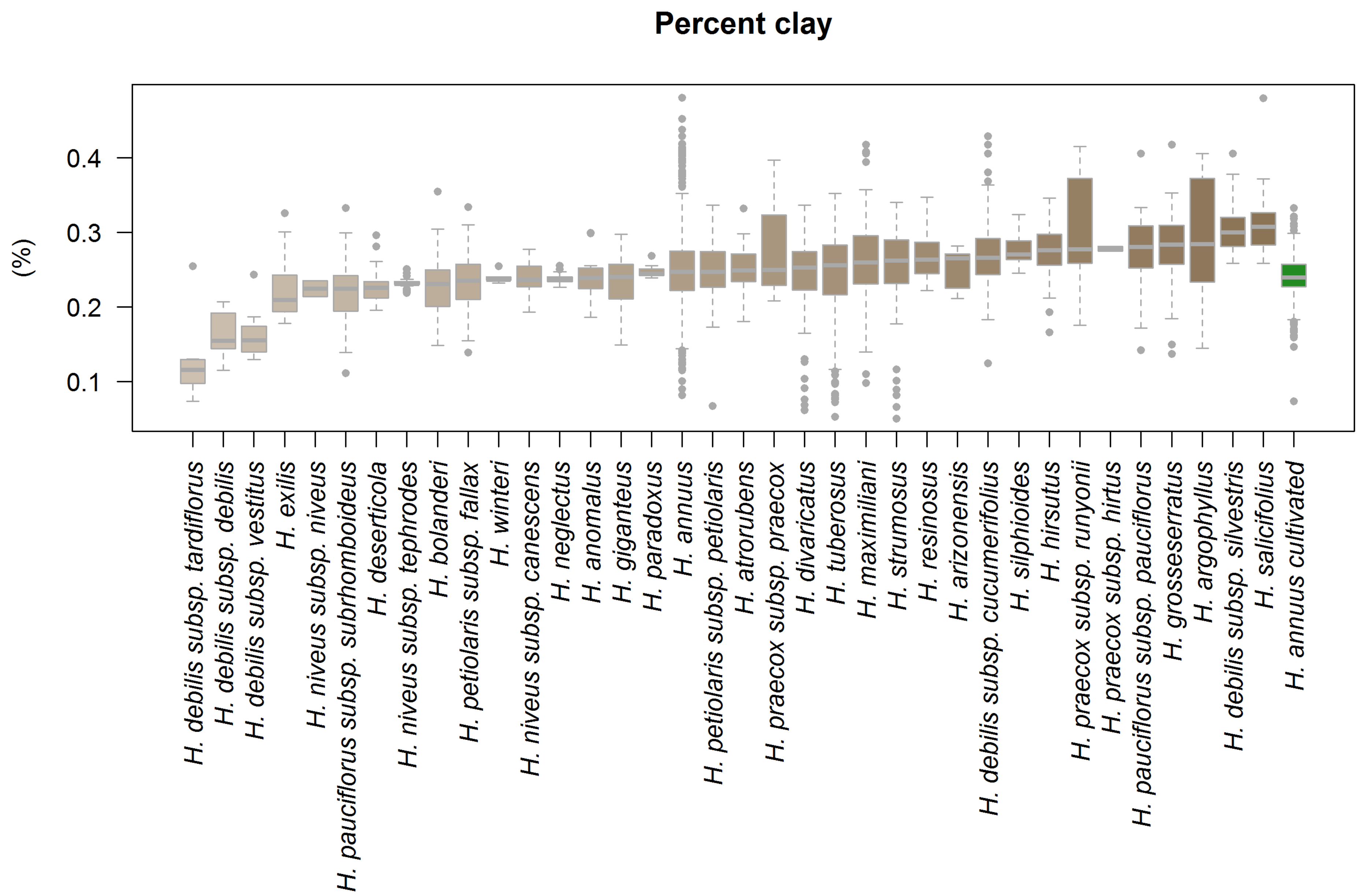

Percent clay
(%)
0.1
0.2
0.3
0.4
H. debilis subsp. tardiflorus
H. debilis subsp. debilis
H. debilis subsp. vestitus
H. exilis
H. niveus subsp. niveus
H. pauciflorus subsp. subrhomboideus
H. deserticola
H. niveus subsp. tephrodes
H. bolanderi
H. petiolaris subsp. fallax
H. winteri
H. niveus subsp. canescens
H. neglectus
H. anomalus
H. giganteus
H. paradoxus
H. annuus
H. petiolaris subsp. petiolaris
H. atrorubens
H. praecox subsp. praecox
H. divaricatus
H. tuberosus
H. maximiliani
H. strumosus
H. resinosus
H. arizonensis
H. debilis subsp. cucumerifolius
H. silphioides
H. hirsutus
H. praecox subsp. runyonii
H. praecox subsp. hirtus
H. pauciflorus subsp. pauciflorus
H. grosseserratus
H. argophyllus
H. debilis subsp. silvestris
H. salicifolius
H. annuus cultivated

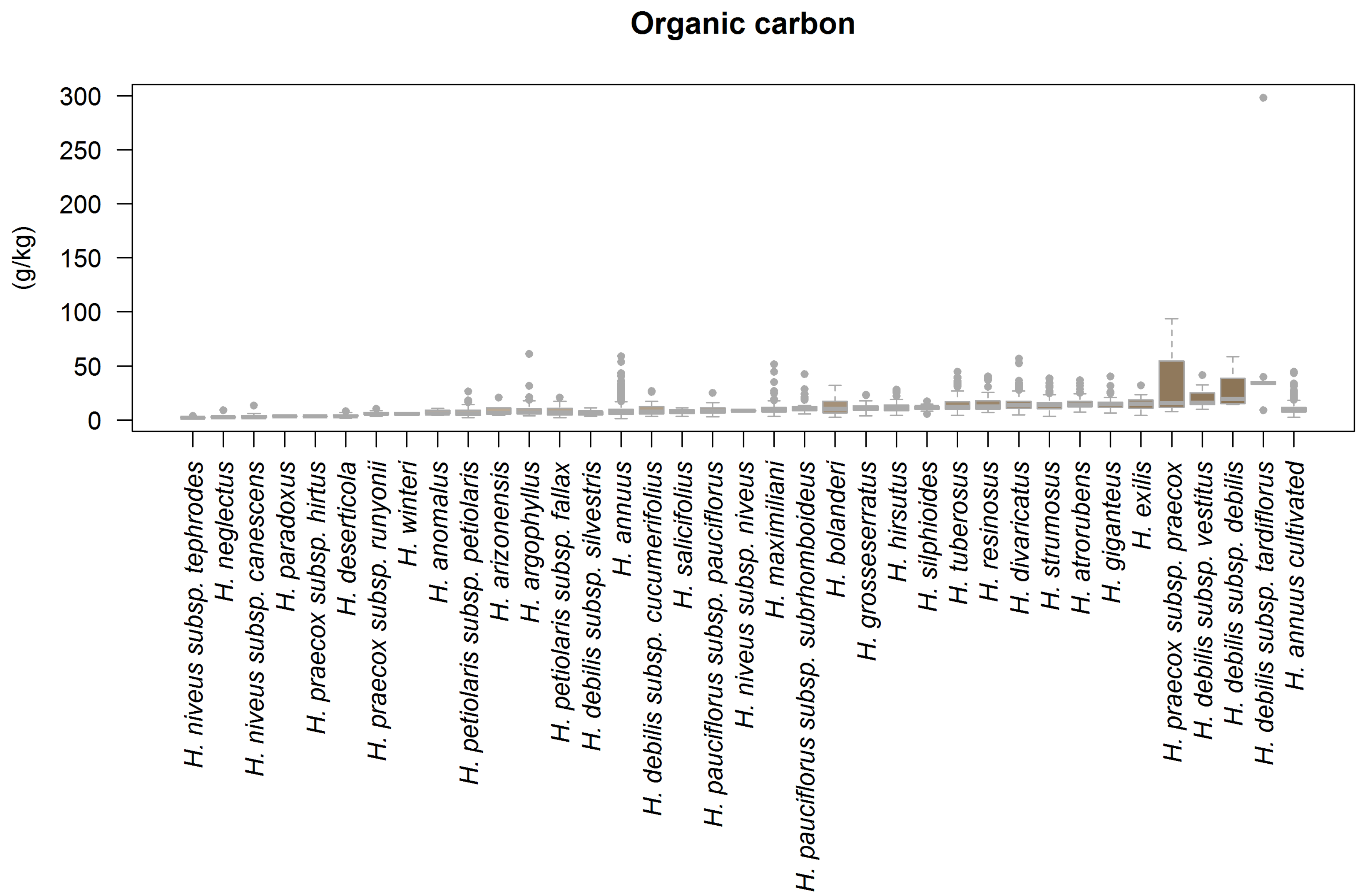

Organic carbon
(g/kg)
0
50
100
150
200
250
300
H. niveus subsp. tephrodes
H. neglectus
H. niveus subsp. canescens
H. paradoxus
H. praecox subsp. hirtus
H. deserticola
H. praecox subsp. runyonii
H. winteri
H. anomalus
H. petiolaris subsp. petiolaris
H. arizonensis
H. argophyllus
H. petiolaris subsp. fallax
H. debilis subsp. silvestris
H. annuus
H. debilis subsp. cucumerifolius
H. salicifolius
H. pauciflorus subsp. pauciflorus
H. niveus subsp. niveus
H. maximiliani
H. pauciflorus subsp. subrhomboideus
H. bolanderi
H. grosseserratus
H. hirsutus
H. silphioides
H. tuberosus
H. resinosus
H. divaricatus
H. strumosus
H. atrorubens
H. giganteus
H. exilis
H. praecox subsp. praecox
H. debilis subsp. vestitus
H. debilis subsp. debilis
H. debilis subsp. tardiflorus
H. annuus cultivated

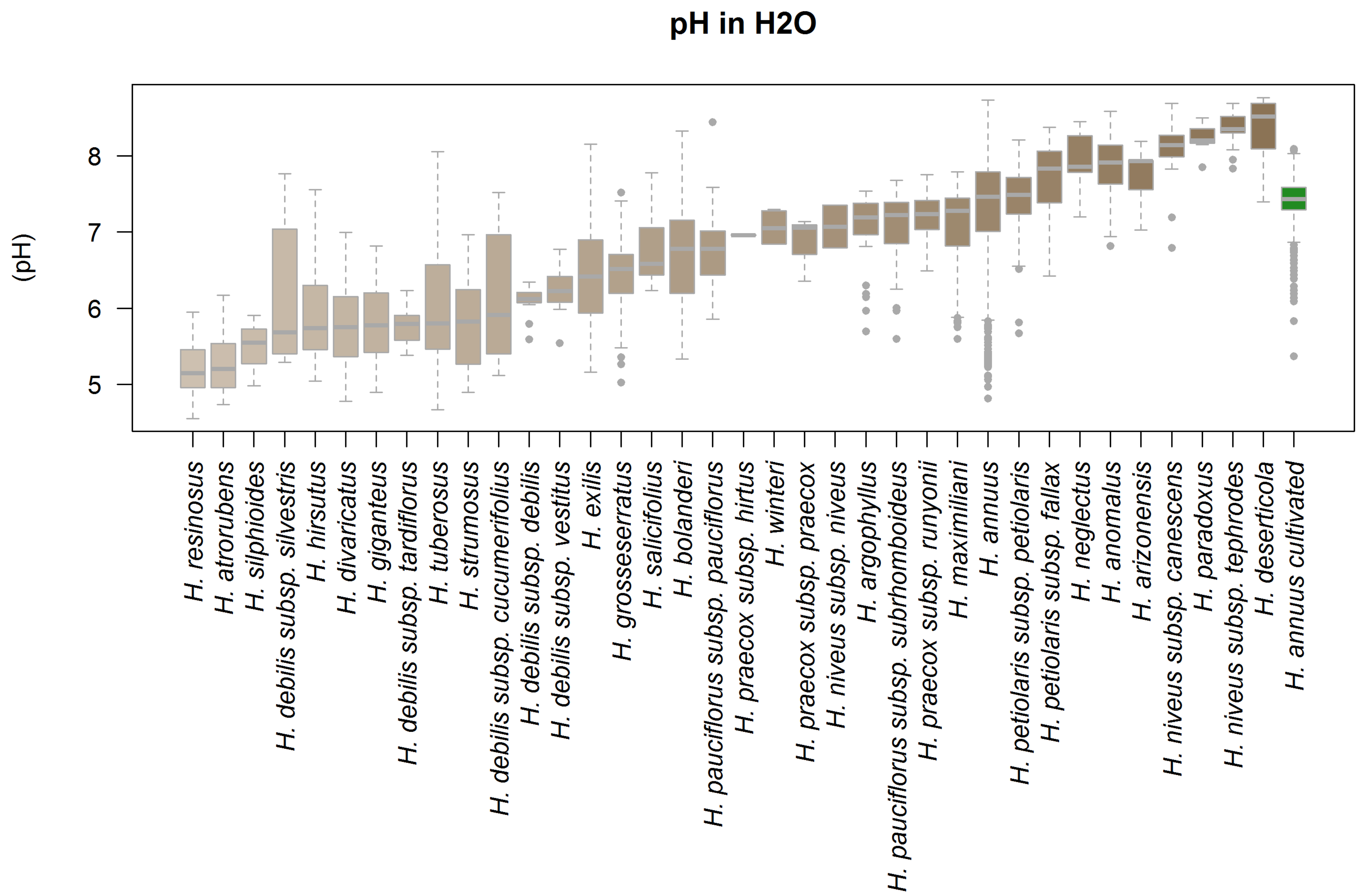

pH in H2O
(pH)
8
7
6
5
H. resinosus
H. atrorubens
H. silphioides
H. debilis subsp. silvestris
H. hirsutus
H. divaricatus
H. giganteus
H. debilis subsp. tardiflorus
H. tuberosus
H. strumosus
H. debilis subsp. cucumerifolius
H. debilis subsp. debilis
H. debilis subsp. vestitus
H. exilis
H. grosseserratus
H. salicifolius
H. bolanderi
H. pauciflorus subsp. pauciflorus
H. praecox subsp. hirtus
H. winteri
H. praecox subsp. praecox
H. niveus subsp. niveus
H. argophyllus
H. pauciflorus subsp. subrhomboideus
H. praecox subsp. runyonii
H. maximiliani
H. annuus
H. petiolaris subsp. petiolaris
H. petiolaris subsp. fallax
H. neglectus
H. anomalus
H. arizonensis
H. niveus subsp. canescens
H. paradoxus
H. niveus subsp. tephrodes
H. deserticola
H. annuus cultivated

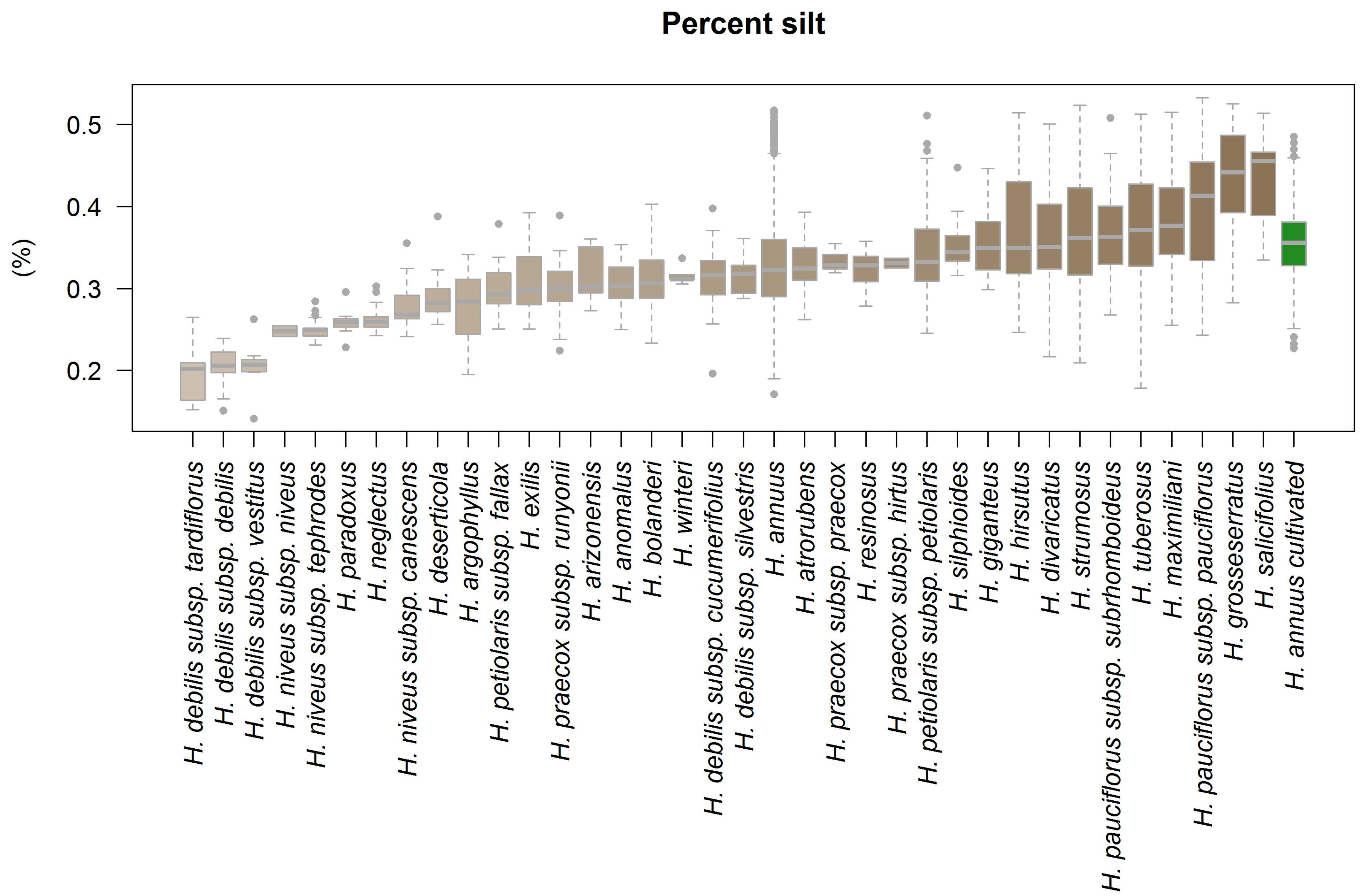

Percent silt
(%)
0.2
0.3
0.4
0.5
H. debilis subsp. tardiflorus
H. debilis subsp. debilis
H. debilis subsp. vestitus
H. niveus subsp. niveus
H. niveus subsp. tephrodes
H. paradoxus
H. neglectus
H. niveus subsp. canescens
H. deserticola
H. argophyllus
H. petiolaris subsp. fallax
H. exilis
H. praecox subsp. runyonii
H. arizonensis
H. anomalus
H. bolanderi
H. winteri
H. debilis subsp. cucumerifolius
H. debilis subsp. silvestris
H. annuus
H. atrorubens
H. praecox subsp. praecox
H. resinosus
H. praecox subsp. hirtus
H. petiolaris subsp. petiolaris
H. silphioides
H. giganteus
H. hirsutus
H. divaricatus
H. strumosus
H. pauciflorus subsp. subrhomboideus
H. tuberosus
H. maximiliani
H. pauciflorus subsp. pauciflorus
H. grosseserratus
H. salicifolius
H. annuus cultivated

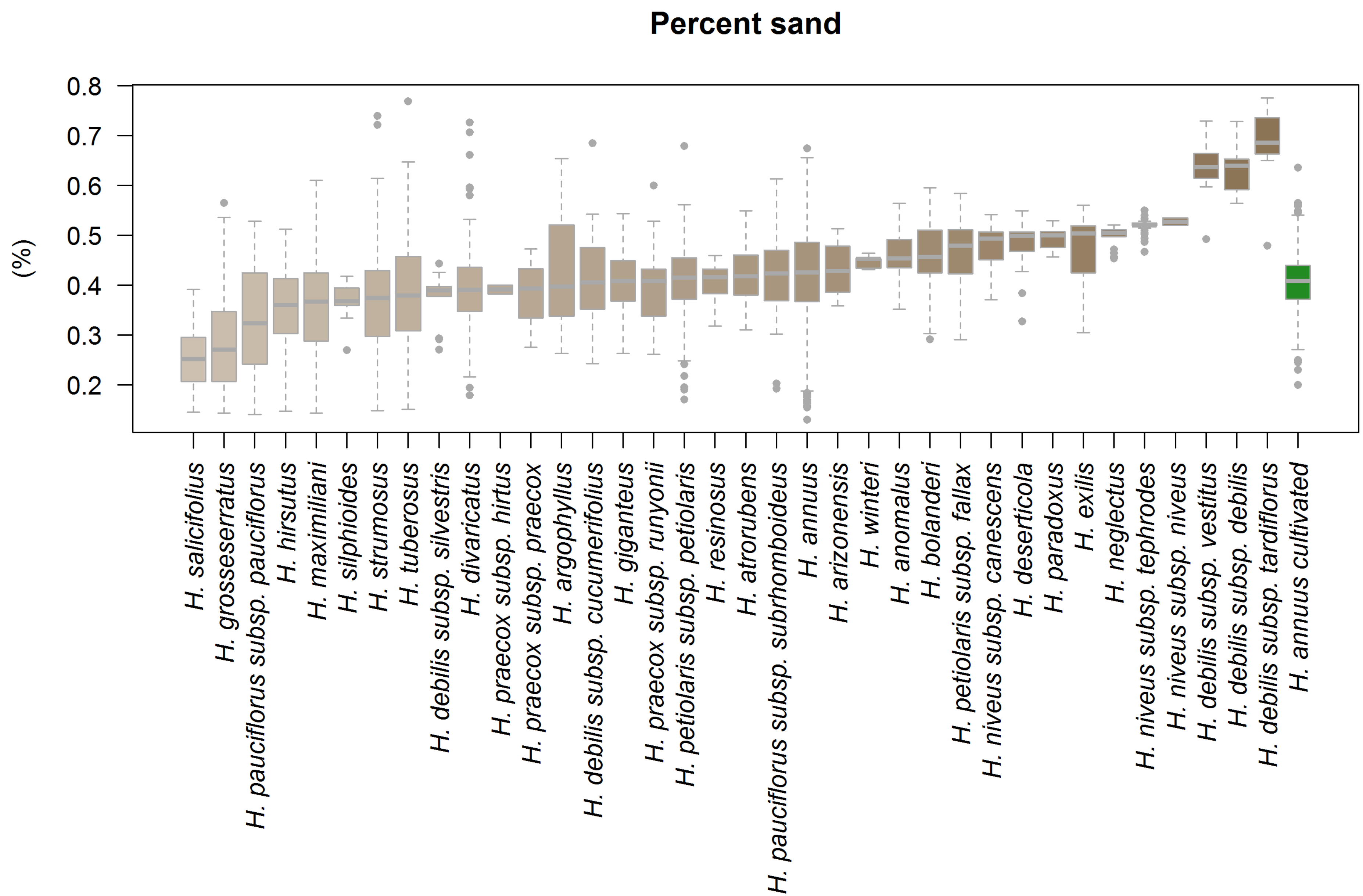

Percent sand
(%)
0.8
0.7
0.6
0.5
0.4
0.3
0.2
H. salicifolius
H. grosseserratus
H. pauciflorus subsp. pauciflorus
H. hirsutus
H. maximiliani
H. silphioides
H. strumosus
H. tuberosus
H. debilis subsp. silvestris
H. divaricatus
H. praecox subsp. hirtus
H. praecox subsp. praecox
H. argophyllus
H. debilis subsp. cucumerifolius
H. giganteus
H. praecox subsp. runyonii
H. petiolaris subsp. petiolaris
H. resinosus
H. atrorubens
H. pauciflorus subsp. subrhomboideus
H. annuus
H. arizonensis
H. winteri
H. anomalus
H. bolanderi
H. petiolaris subsp. fallax
H. niveus subsp. canescens
H. deserticola
H. paradoxus
H. exilis
H. neglectus
H. niveus subsp. tephrodes
H. niveus subsp. niveus
H. debilis subsp. vestitus
H. debilis subsp. debilis
H. debilis subsp. tardiflorus
H. annuus cultivated
