# Supplementary material for: Ecogeography and utility to plant breeding of the crop wild relatives of sunflower (Helianthus annuus L.)
Source: Front Plant Sci. 2015 Oct 8;6:841. doi: 10.3389/fpls.2015.00841 (PMC4597133; doi:10.3389/fpls.2015.00841)
Supplement: Supplementary file 9 [file Image2.PDF]

Figure S2. Species distribution maps for the 36 *Helianthus* taxa examined in this study.

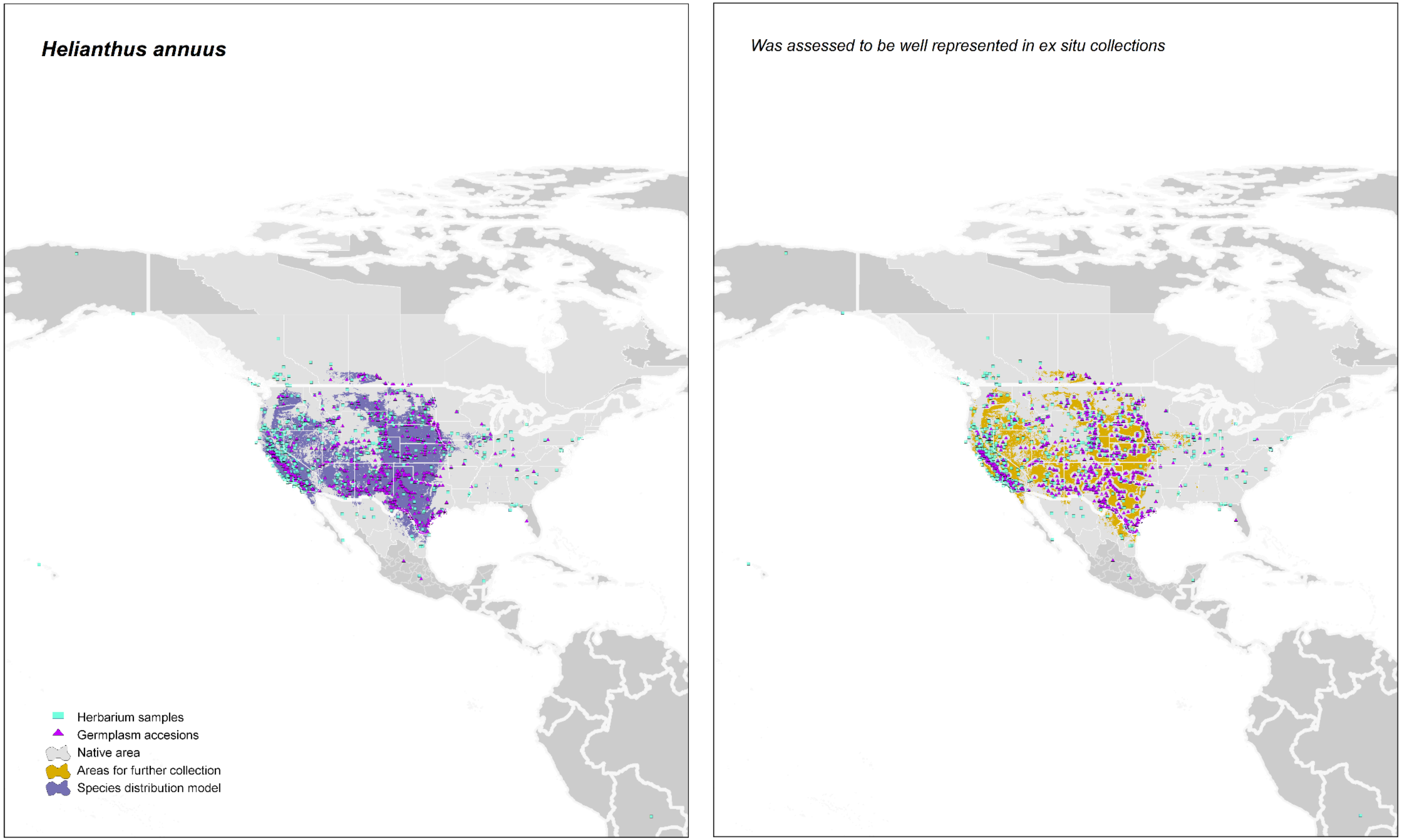

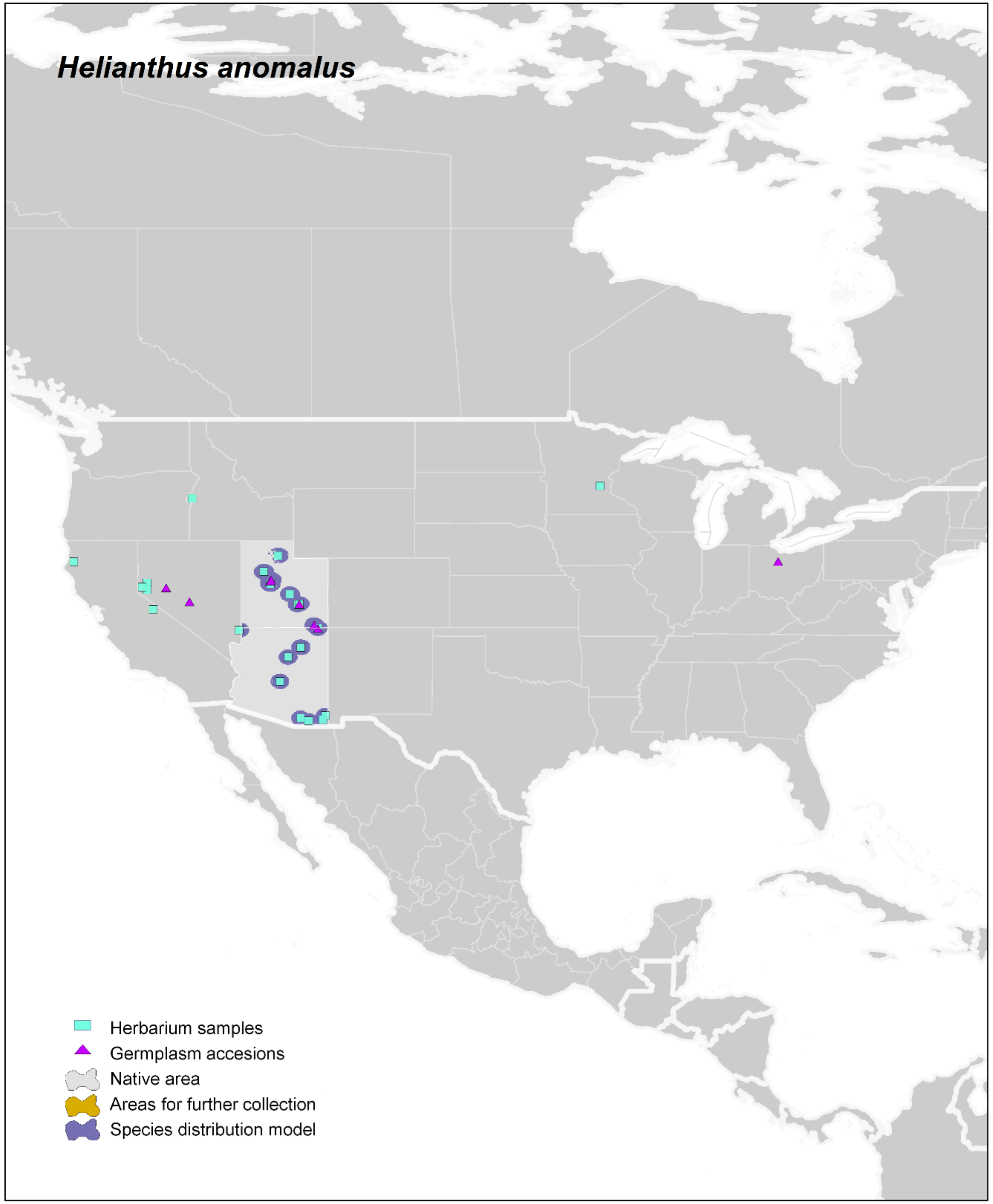
Helianthus anomalus
Herbarium samples
Germplasm accesions
Native area
Areas for further collection
Species distribution model

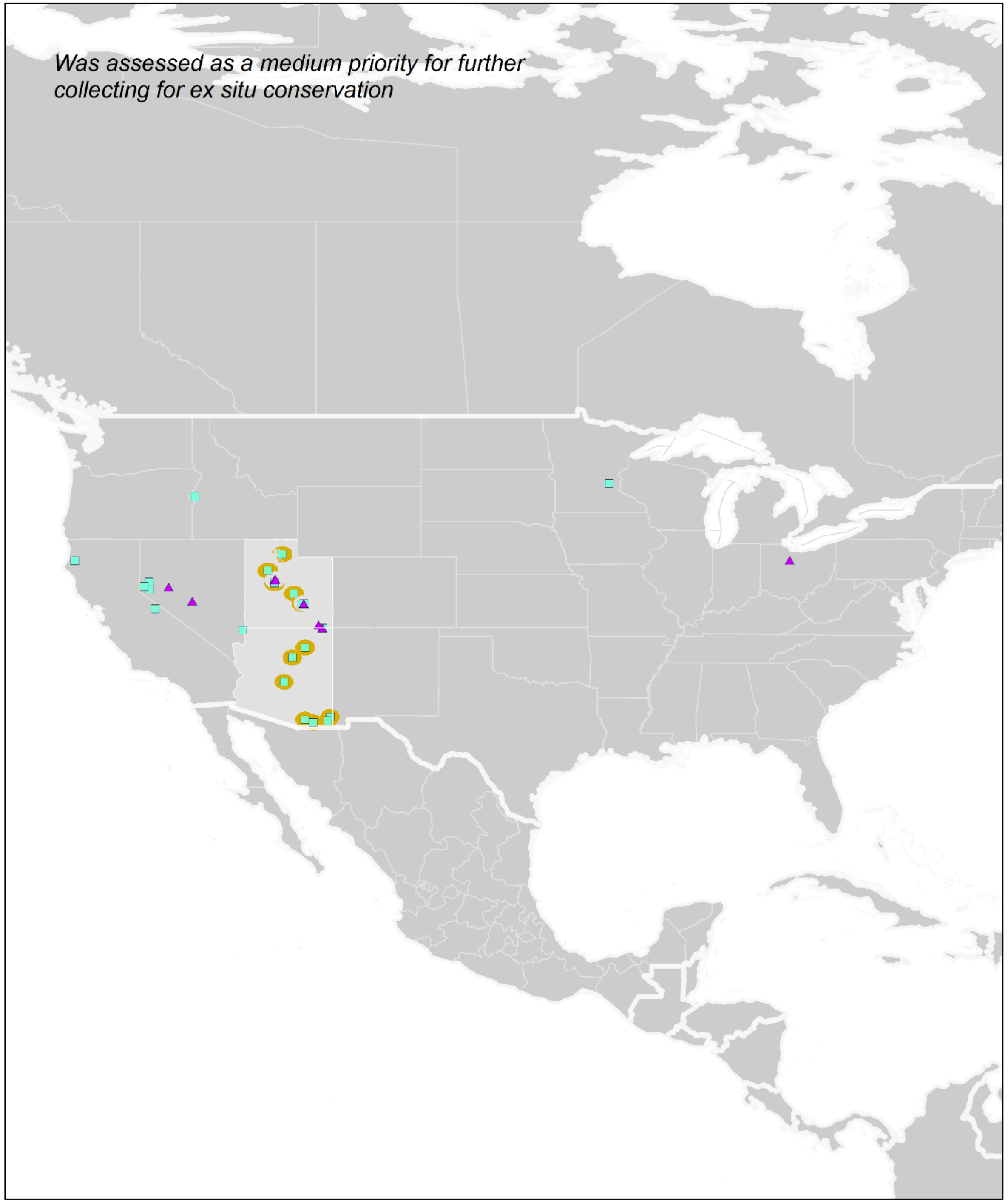
Was assessed as a medium priority for further collecting for ex situ conservation

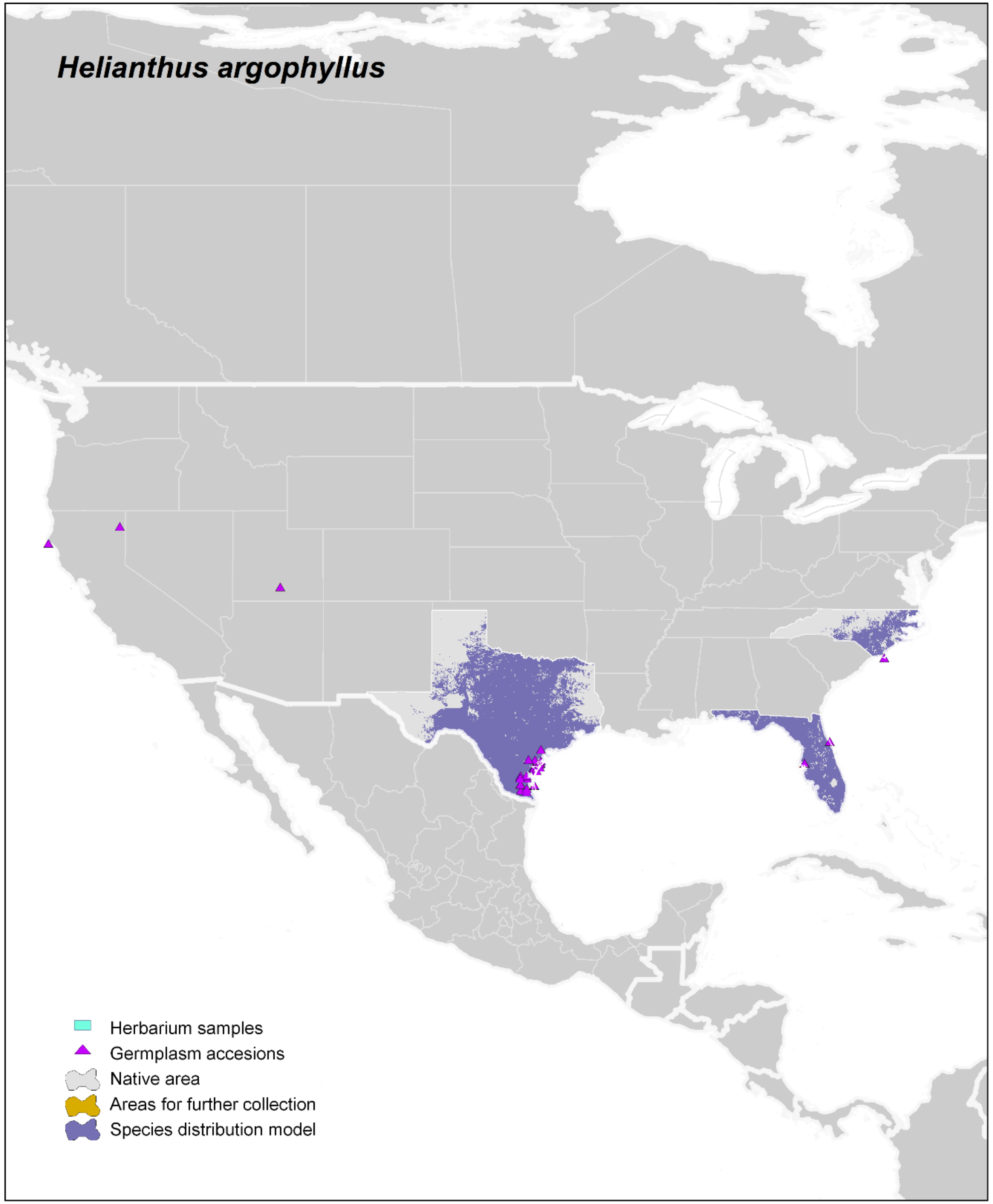

Helianthus argophyllus
Herbarium samples
Germplasm accesions
Native area
Areas for further collection
Species distribution model

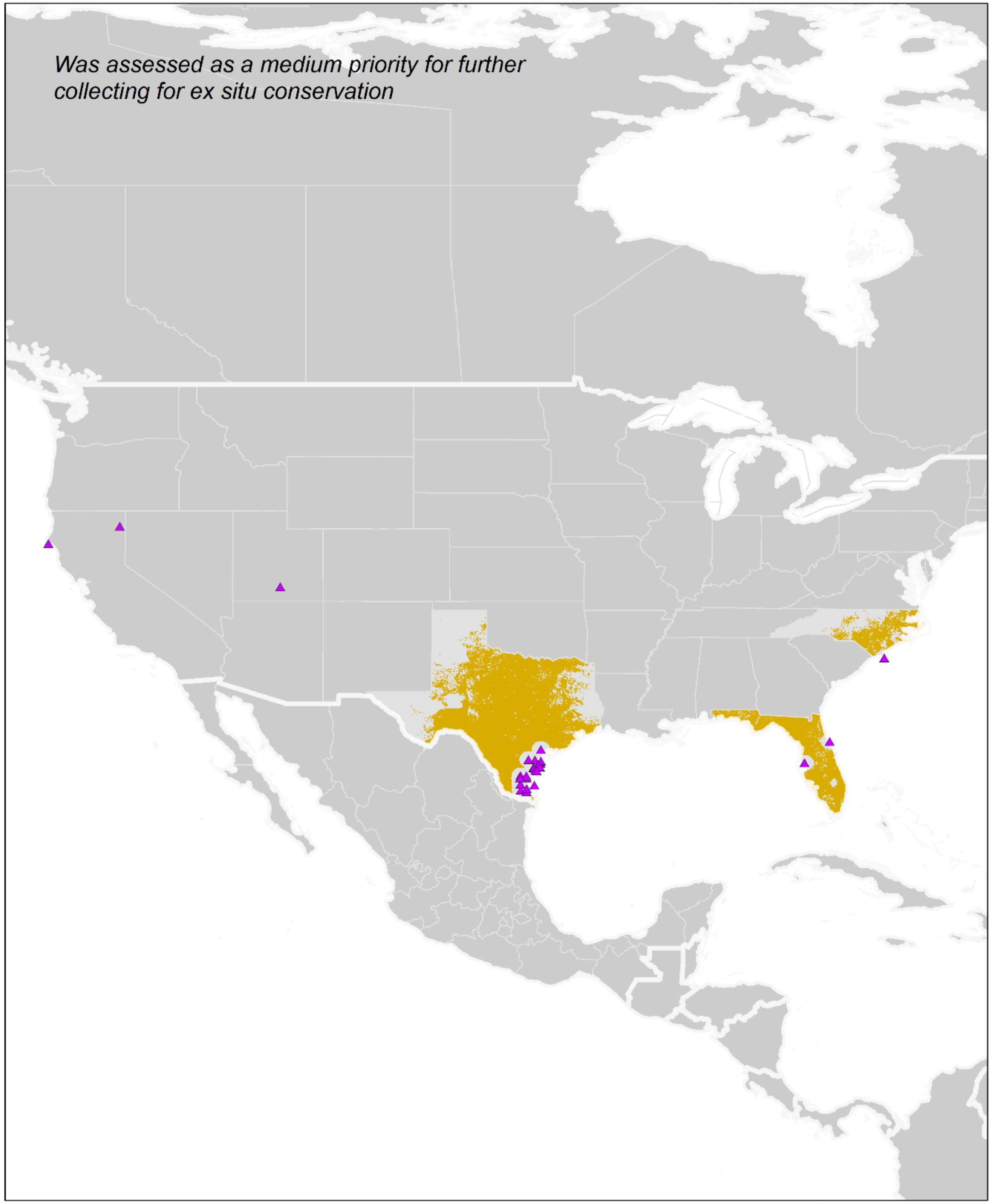

Was assessed as a medium priority for further collecting for ex situ conservation

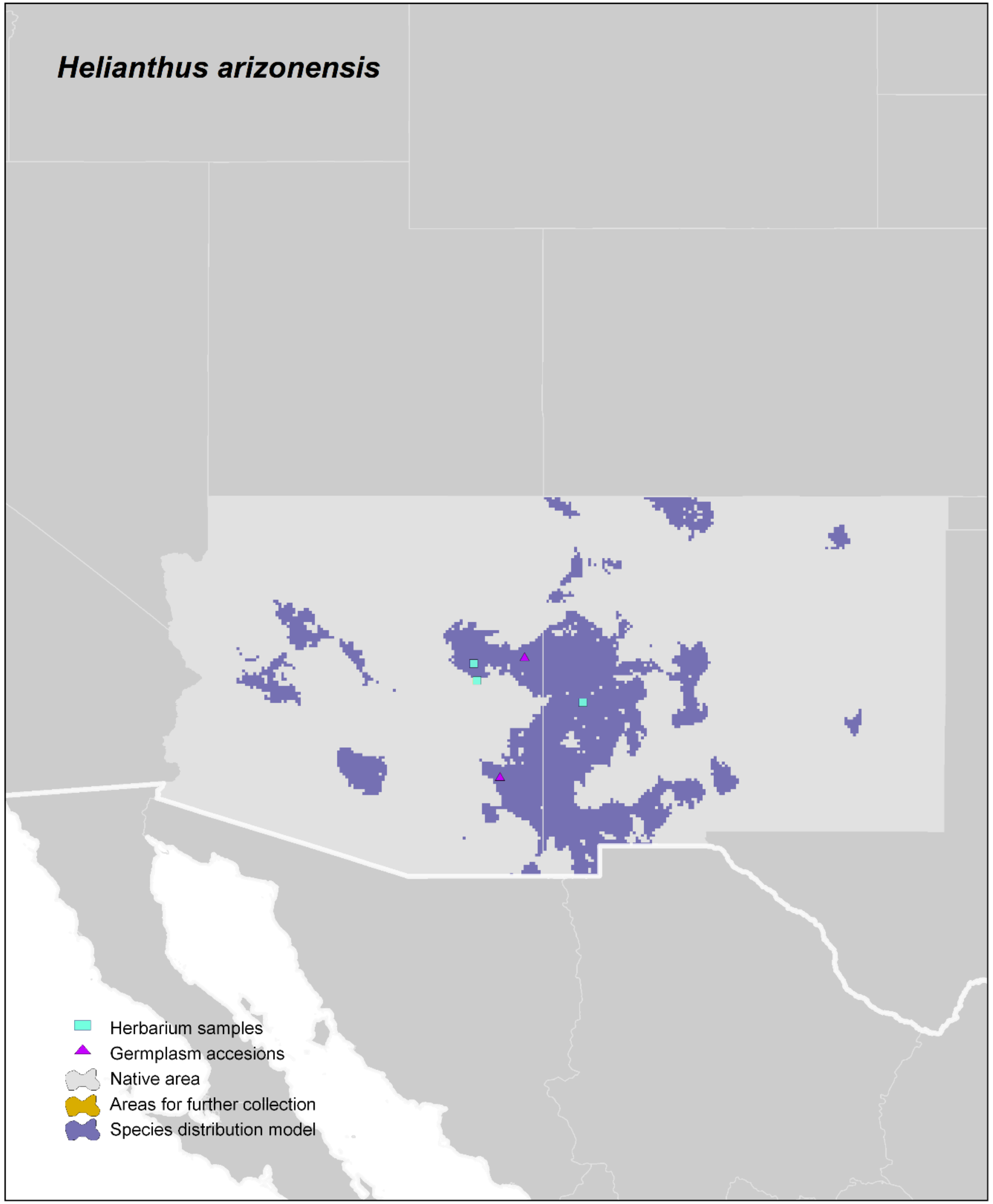
Helianthus arizonensis
Herbarium samples
Germplasm accesions
Native area
Areas for further collection
Species distribution model

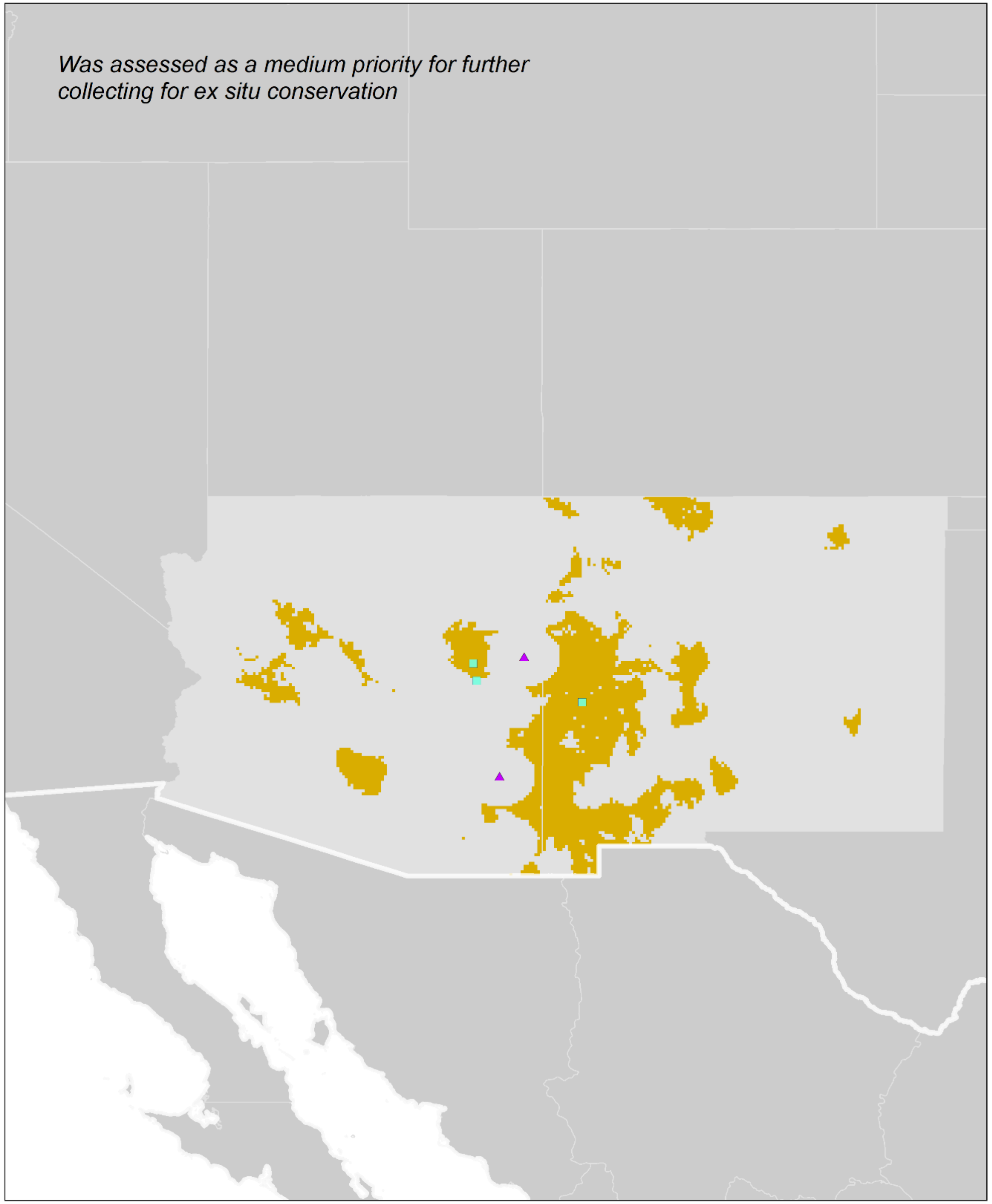
Was assessed as a medium priority for further
collecting for ex situ conservation

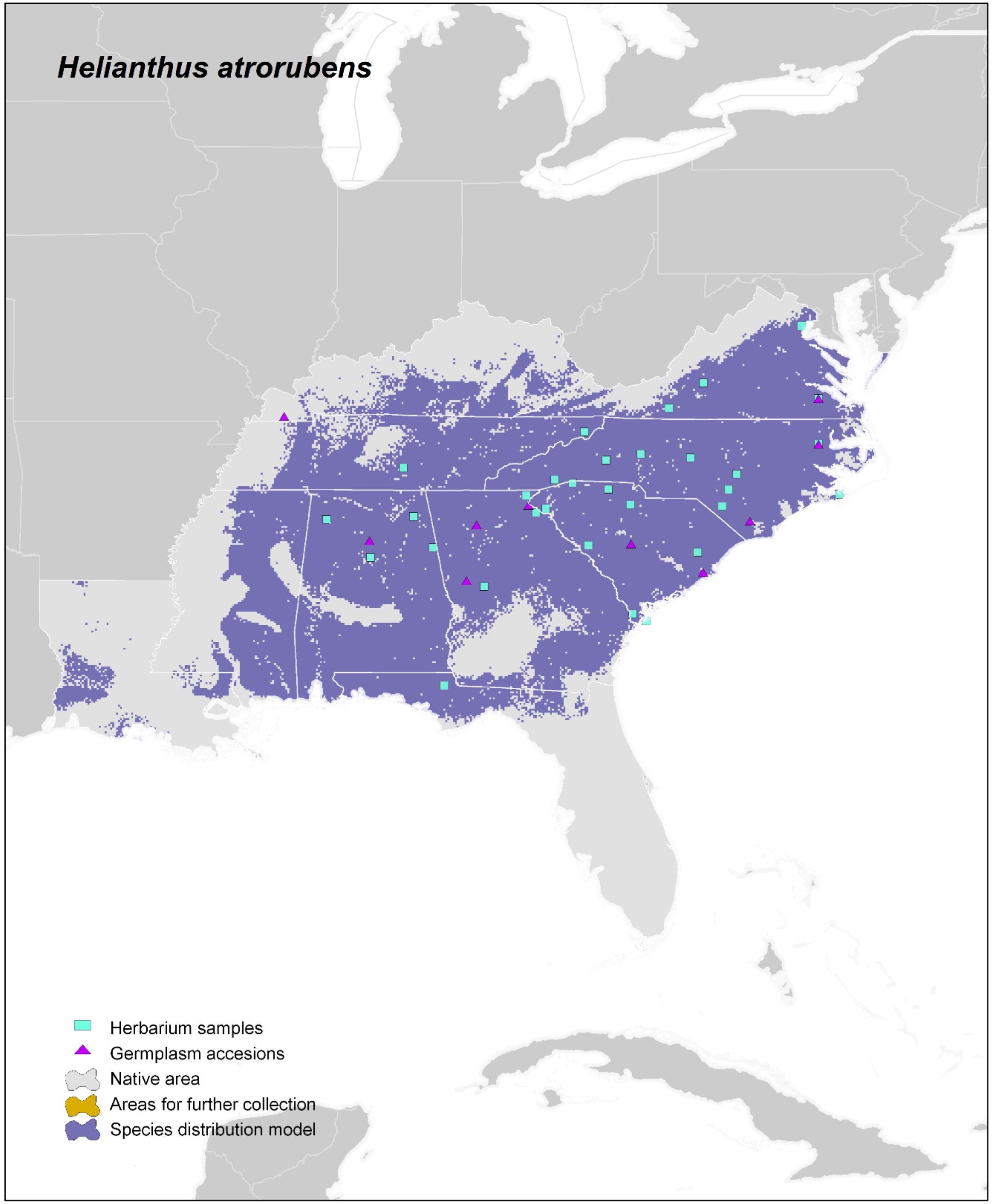
Helianthus atrorubens
Herbarium samples
Germplasm accesions
Native area
Areas for further collection
Species distribution model

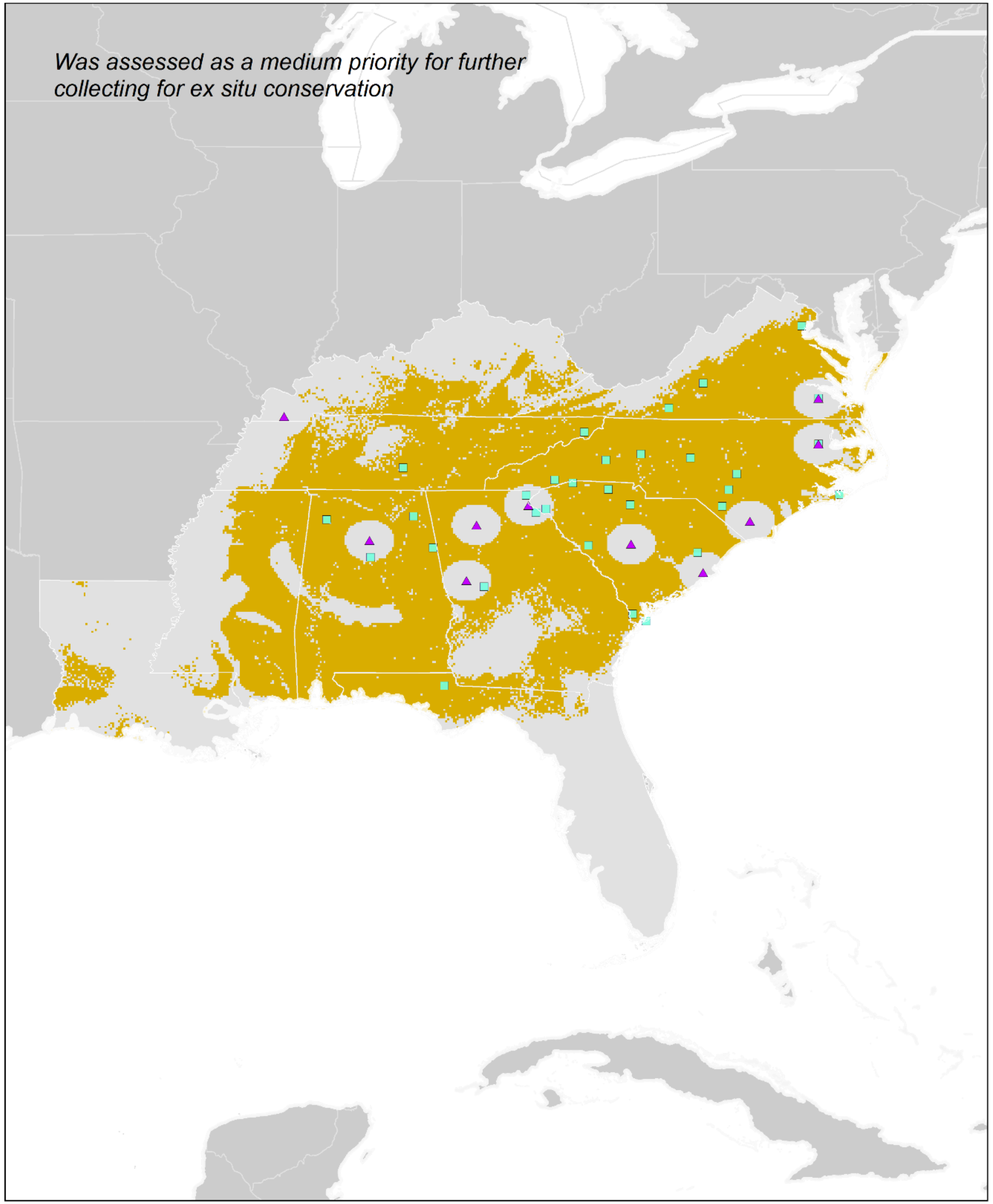
Was assessed as a medium priority for further collecting for ex situ conservation

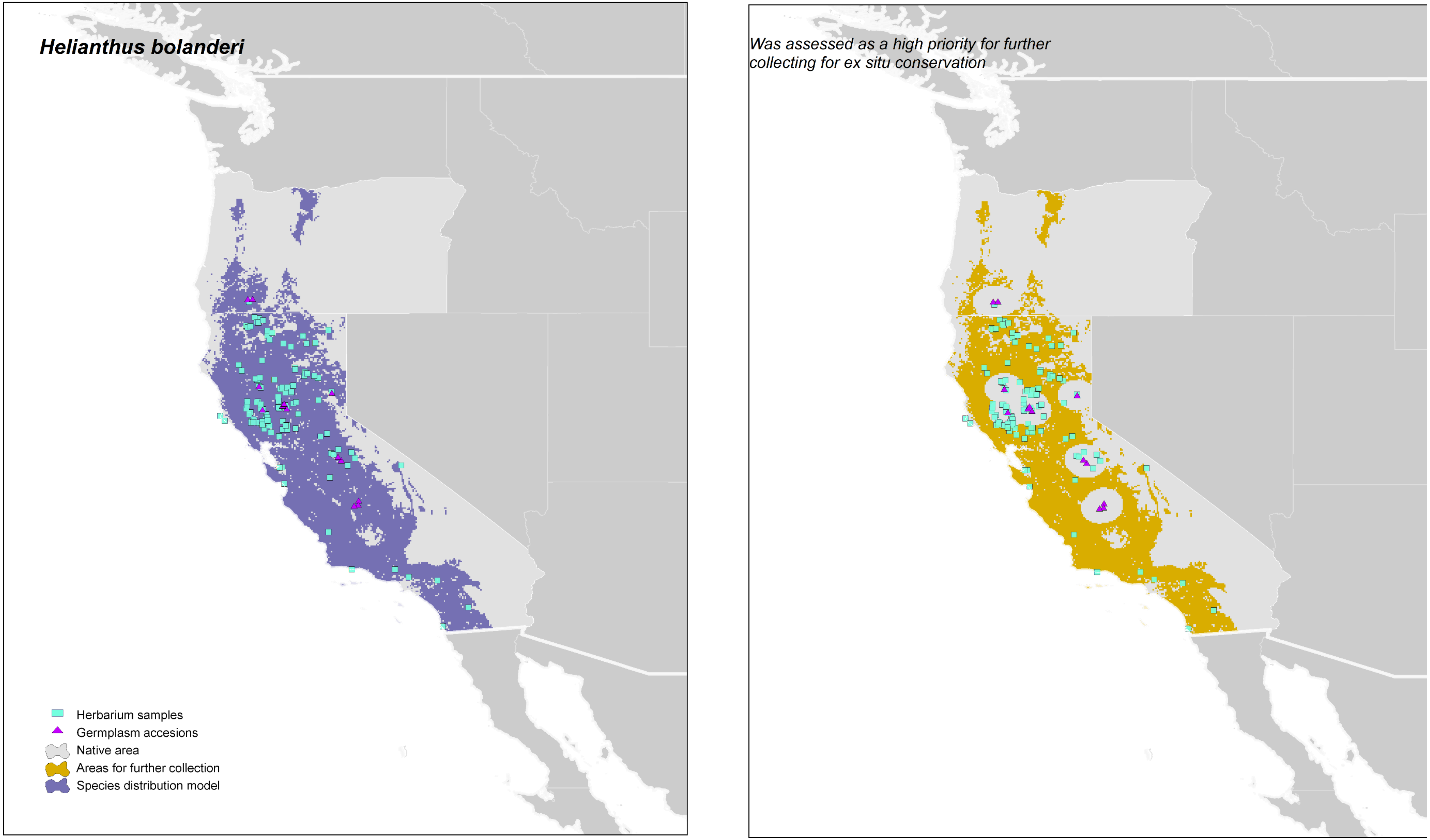
Helianthus bolanderi
Herbarium samples
Germplasm accesions
Native area
Areas for further collection
Species distribution model
Was assessed as a high priority for further
collecting for ex situ conservation

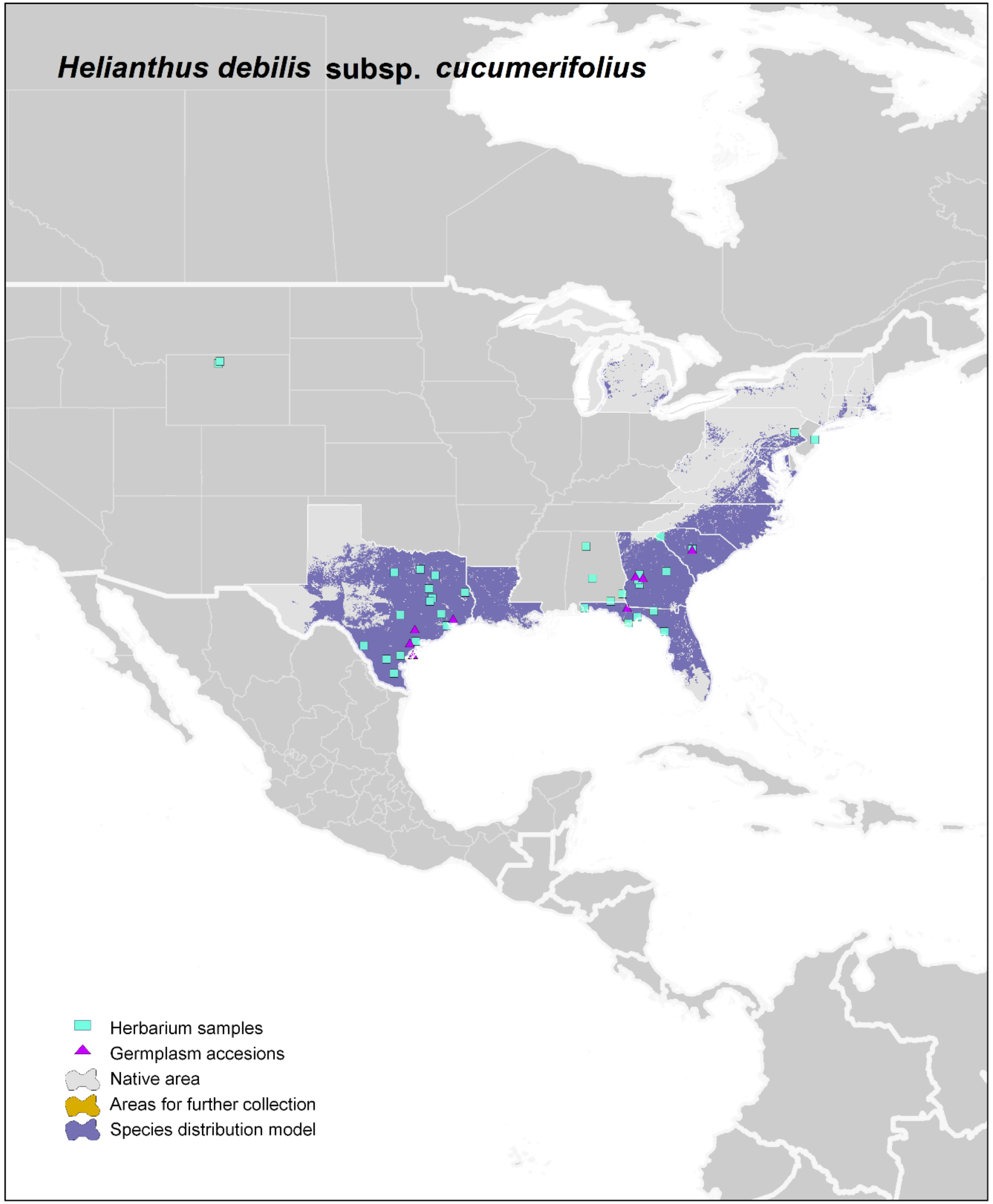
Helianthus debilis subsp. *cucumerifolius*
Herbarium samples
Germplasm accesions
Native area
Areas for further collection
Species distribution model

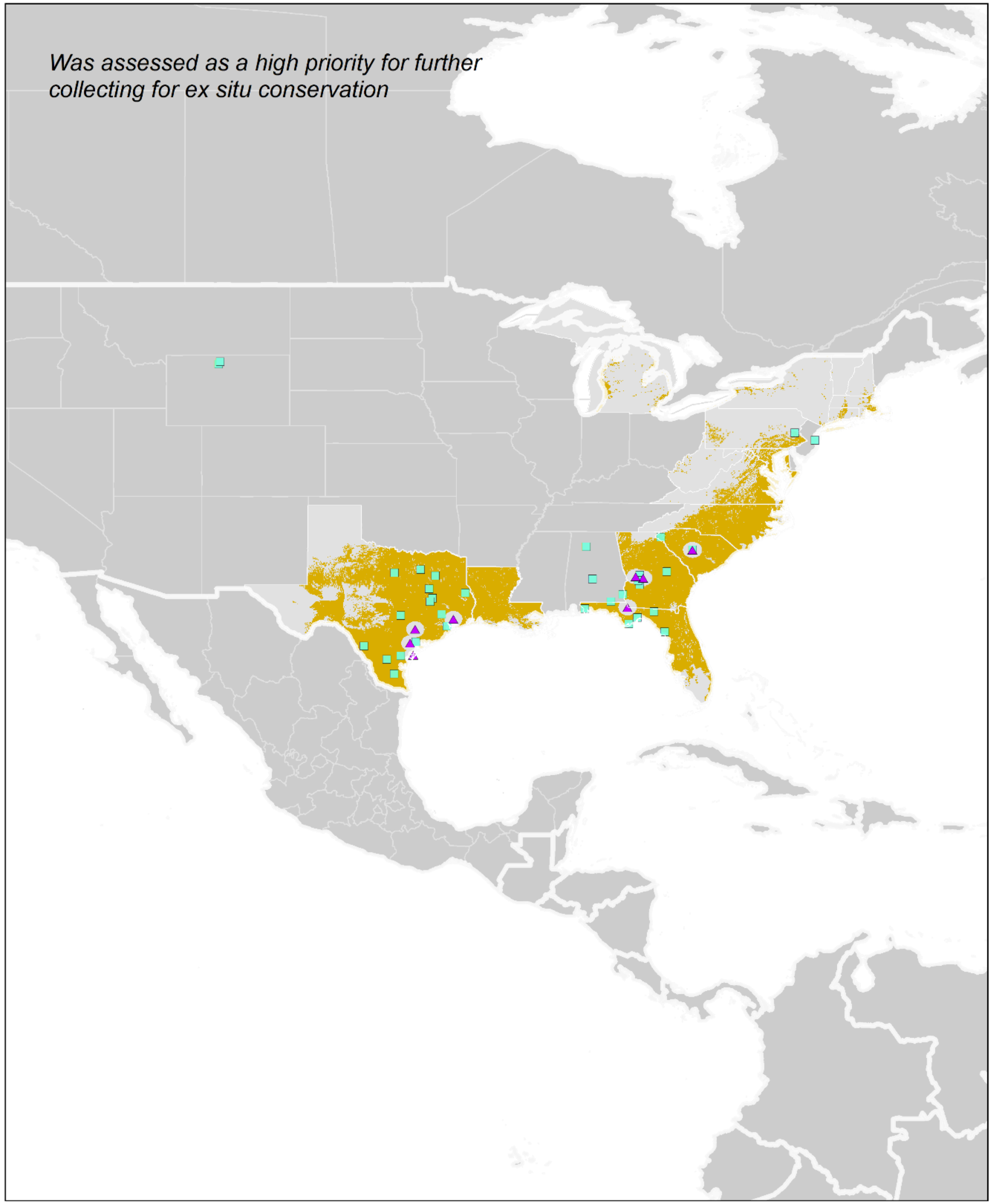
Was assessed as a high priority for further collecting for ex situ conservation

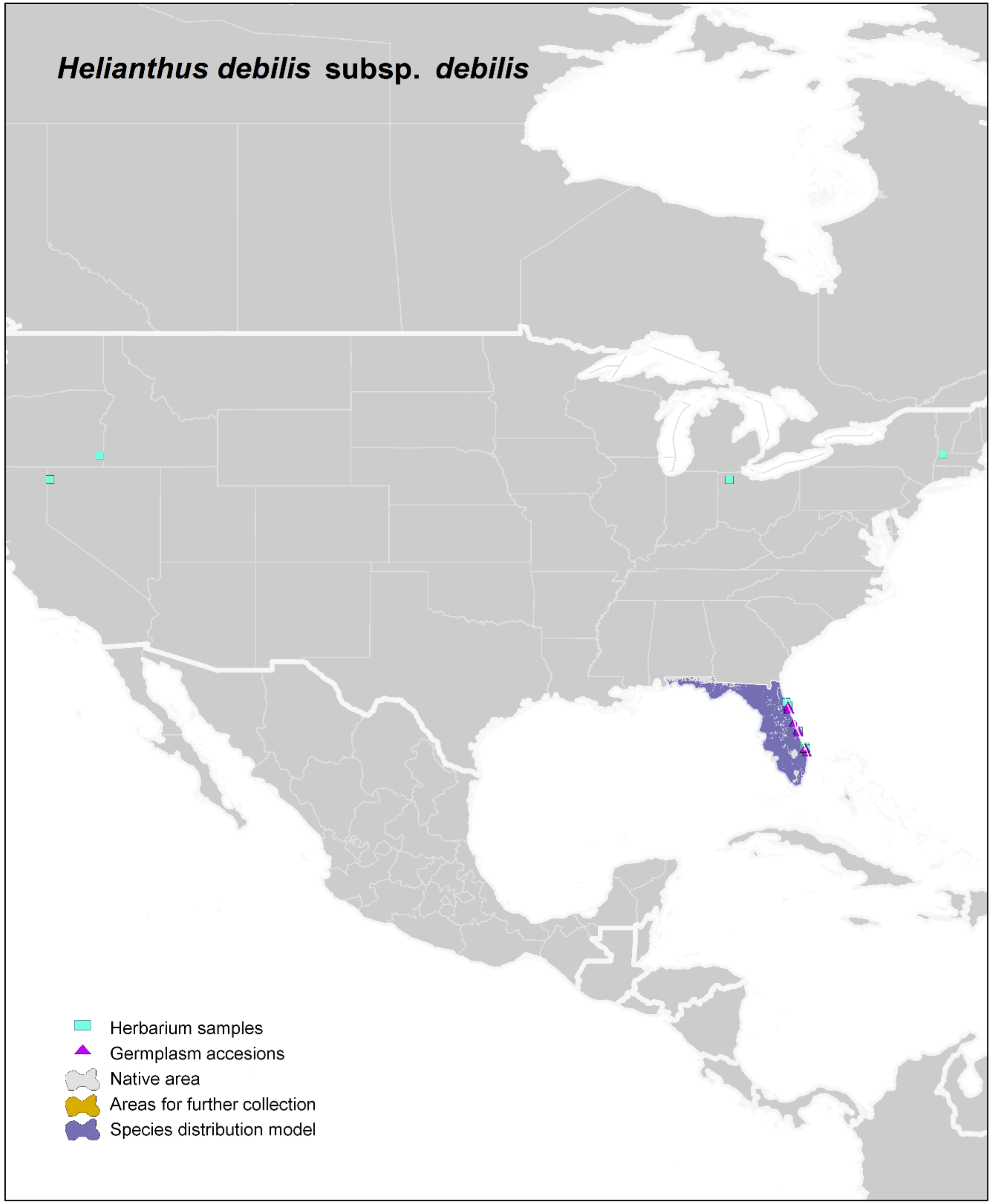
Helianthus debilis subsp. *debilis*
Herbarium samples
Germplasm accesions
Native area
Areas for further collection
Species distribution model

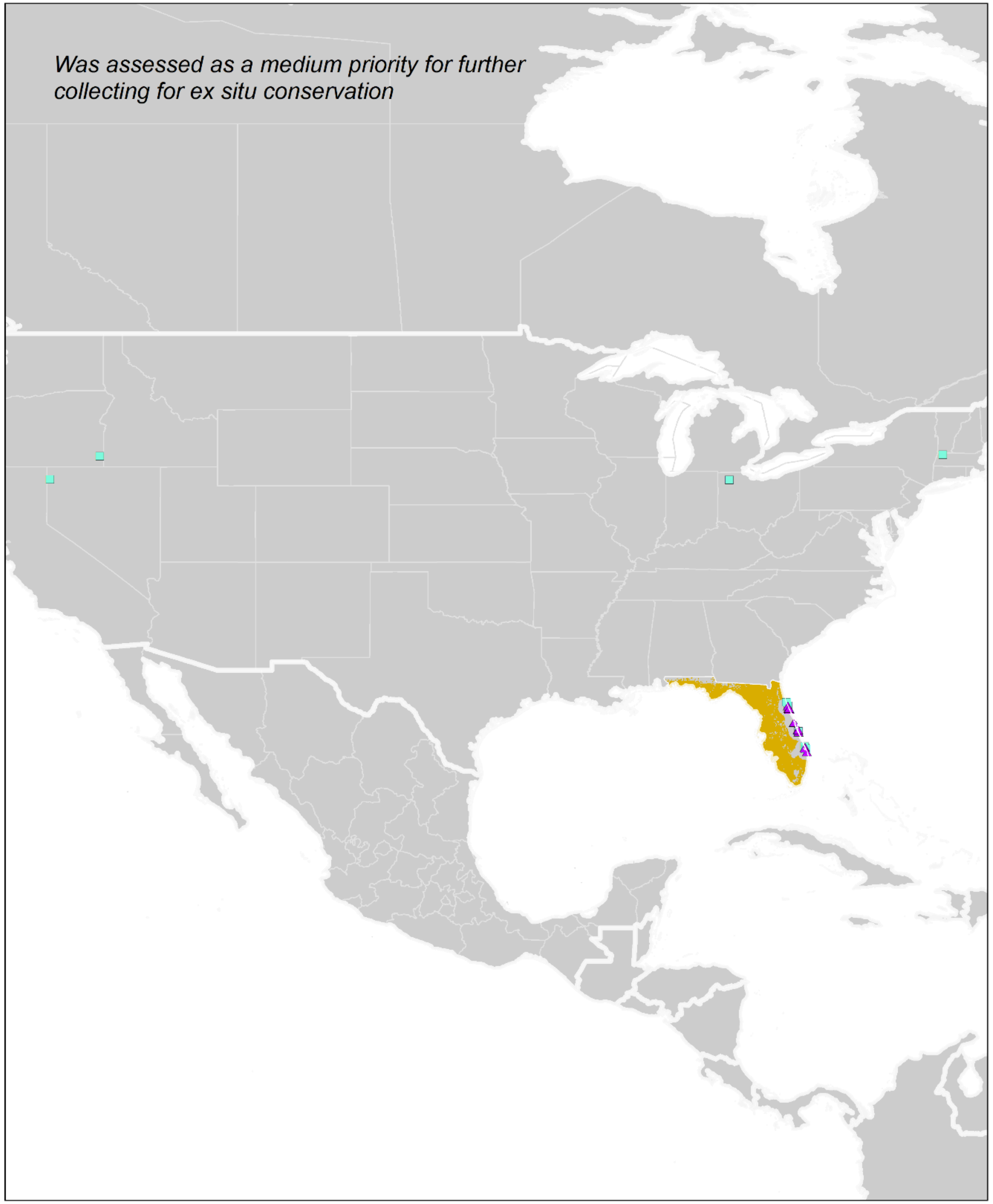
Was assessed as a medium priority for further collecting for ex situ conservation

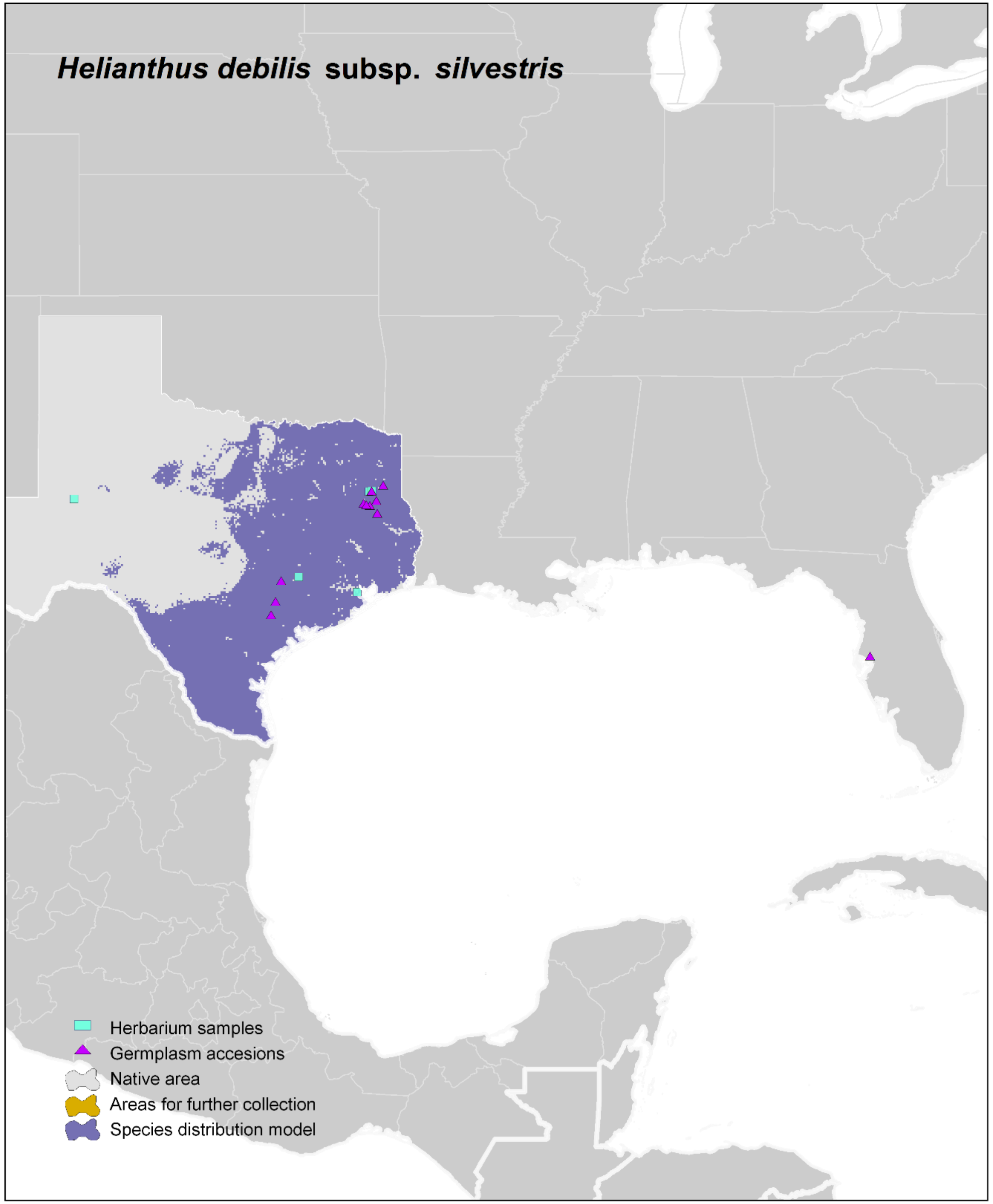
Helianthus debilis subsp. *silvestris*
Herbarium samples
Germplasm accesions
Native area
Areas for further collection
Species distribution model

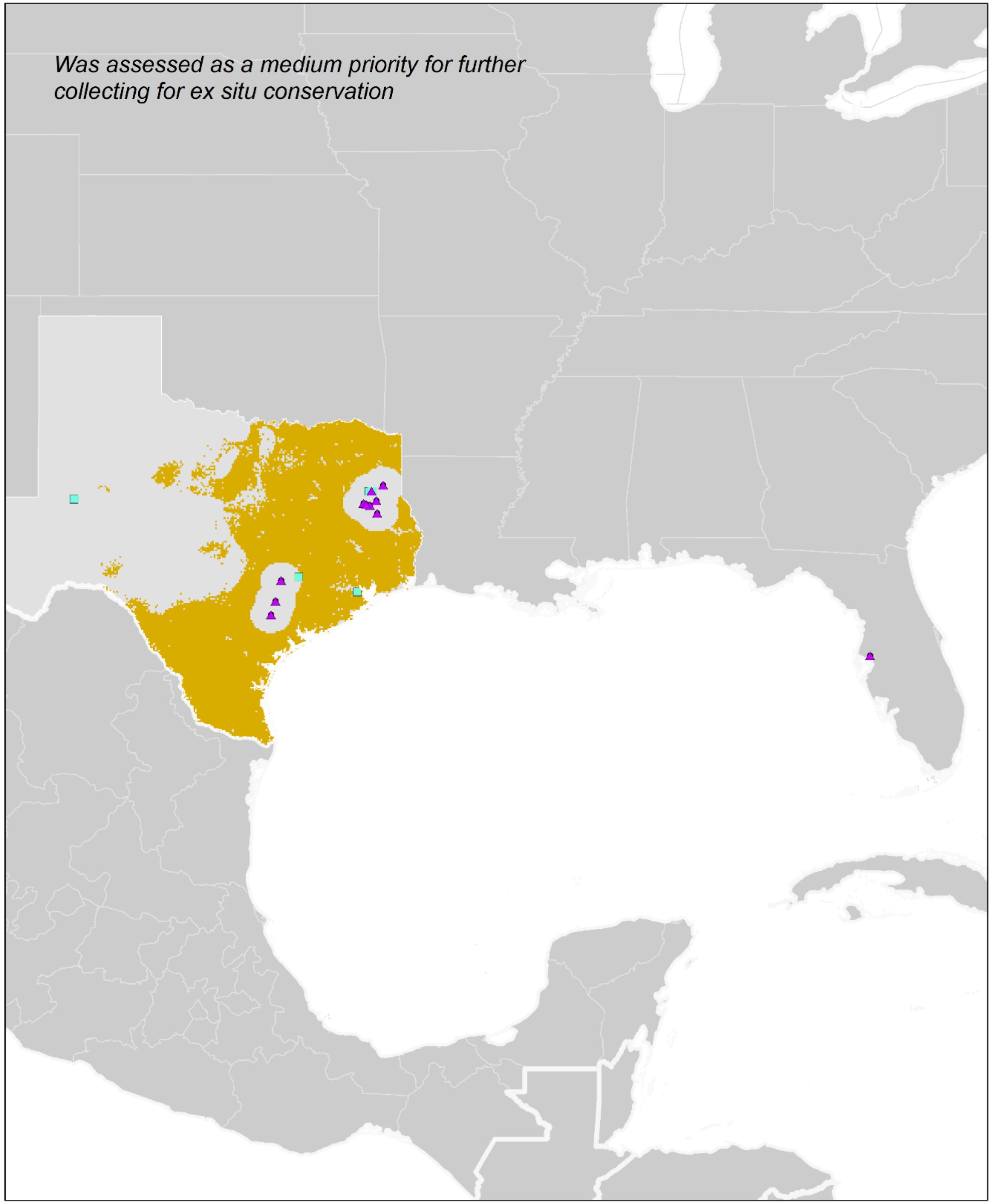
Was assessed as a medium priority for further collecting for ex situ conservation

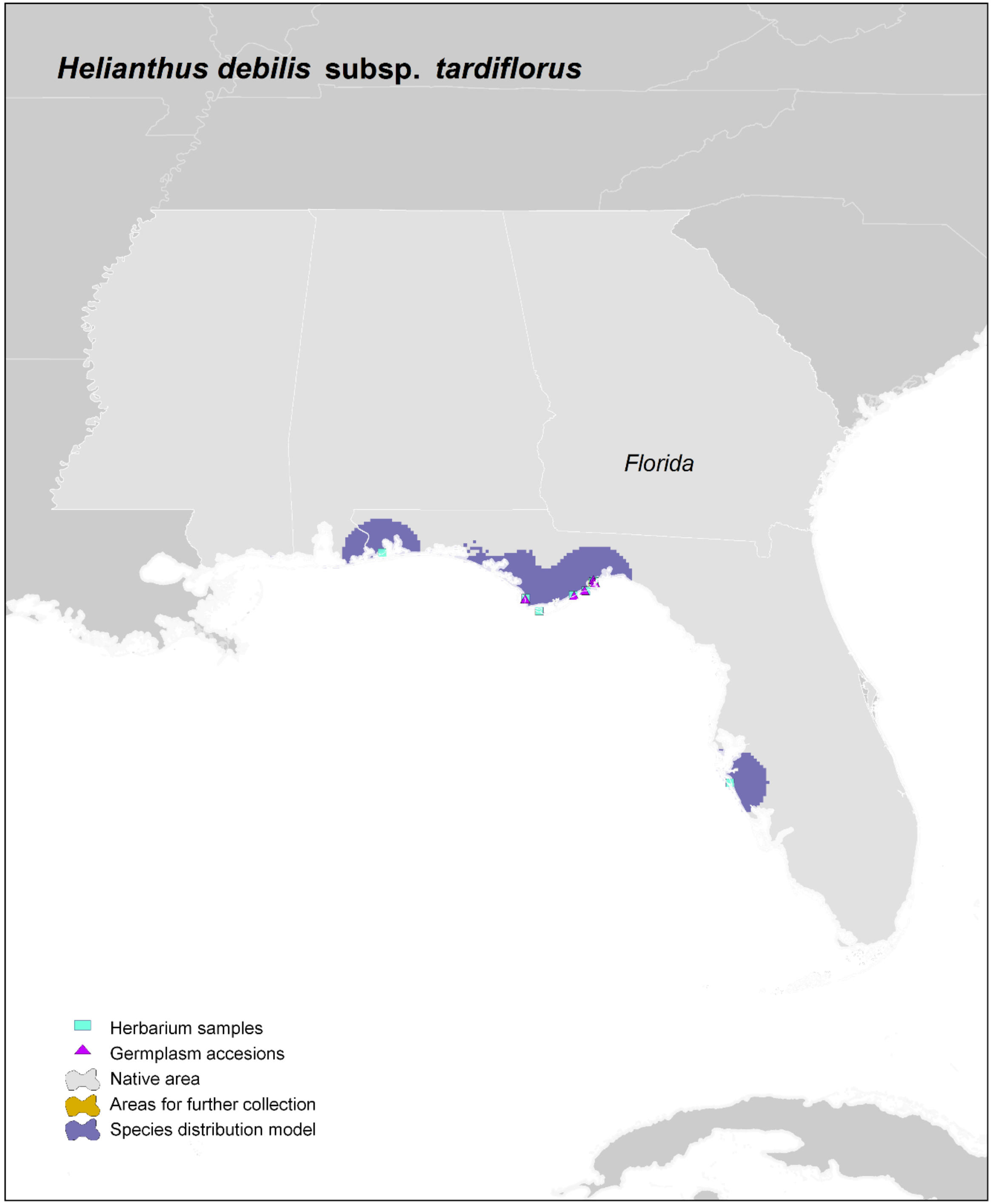
Helianthus debilis subsp. tardiflorus
Florida
Herbarium samples
Germplasm accesions
Native area
Areas for further collection
Species distribution model

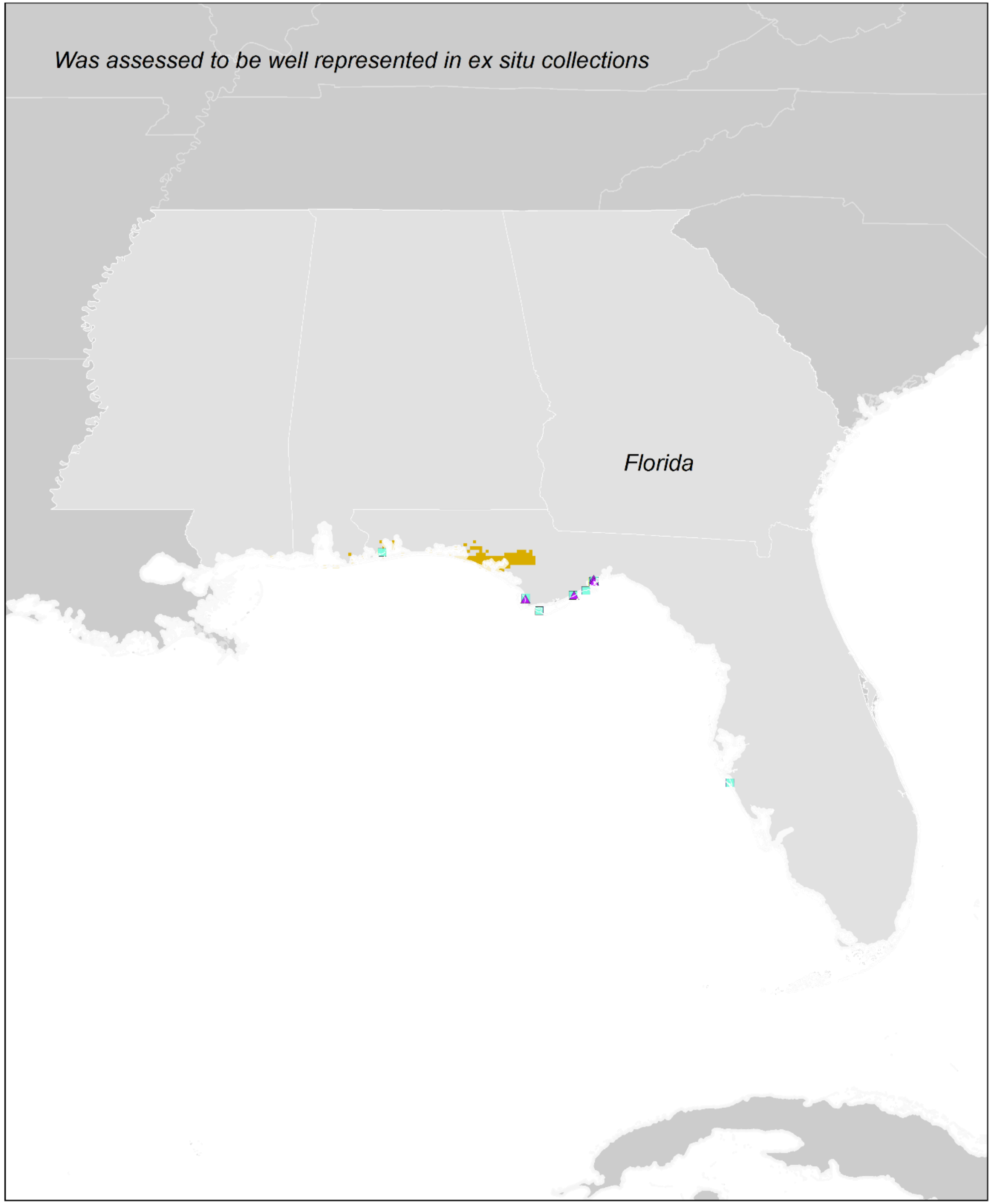
Was assessed to be well represented in ex situ collections
Florida

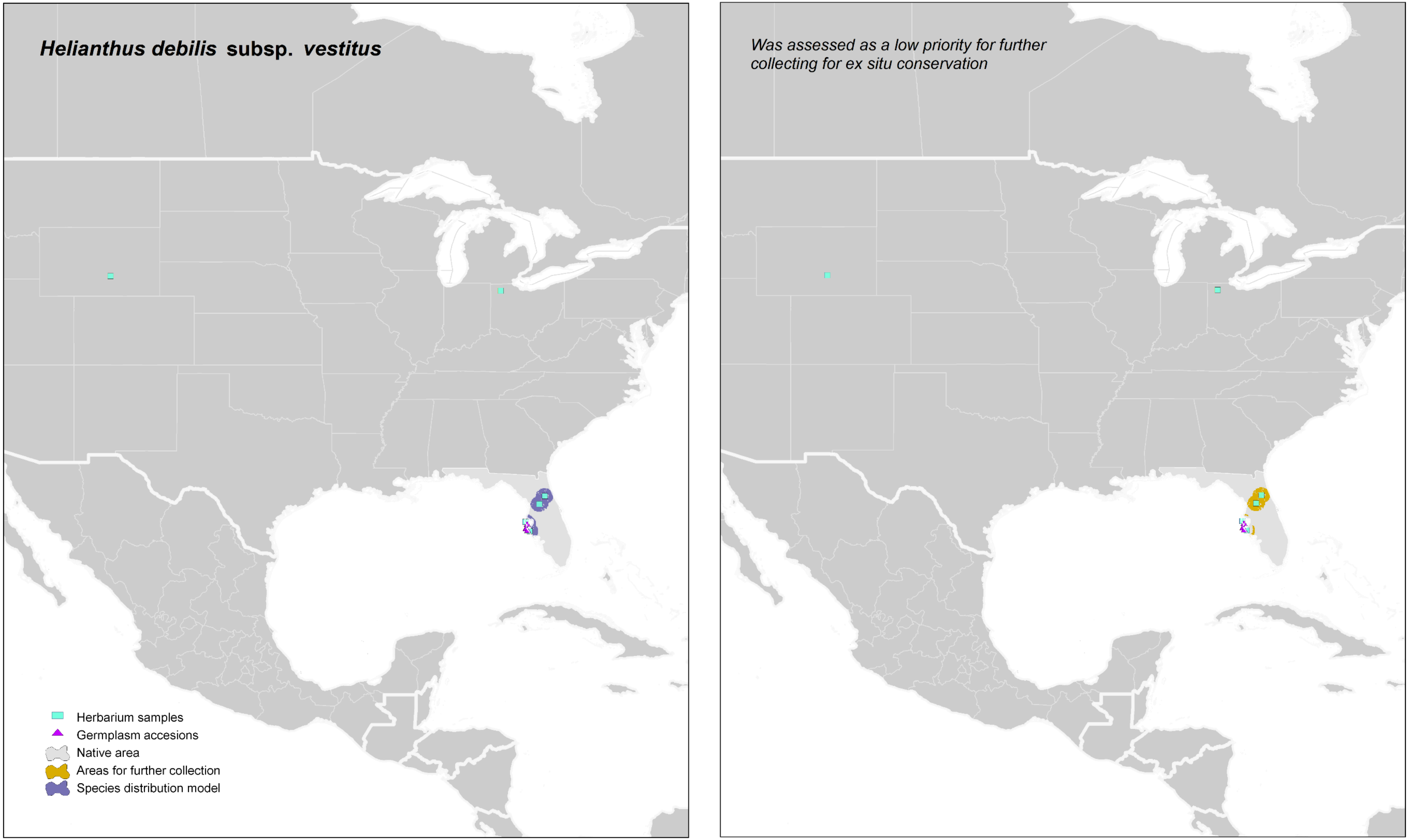
Helianthus debilis subsp. vestitus
Herbarium samples
Germplasm accesions
Native area
Areas for further collection
Species distribution model
Was assessed as a low priority for further collecting for ex situ conservation

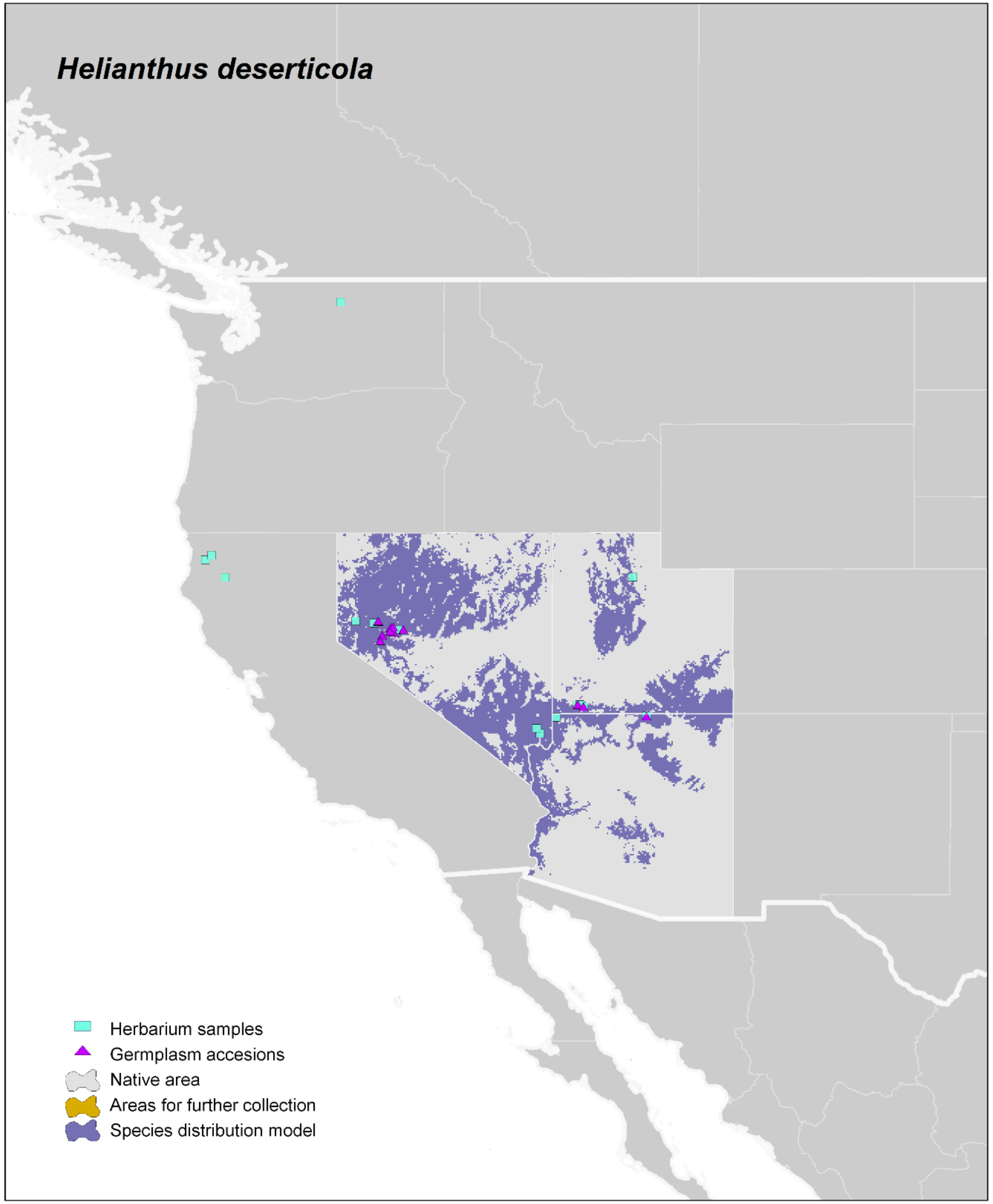
Helianthus deserticola
Herbarium samples
Germplasm accesions
Native area
Areas for further collection
Species distribution model

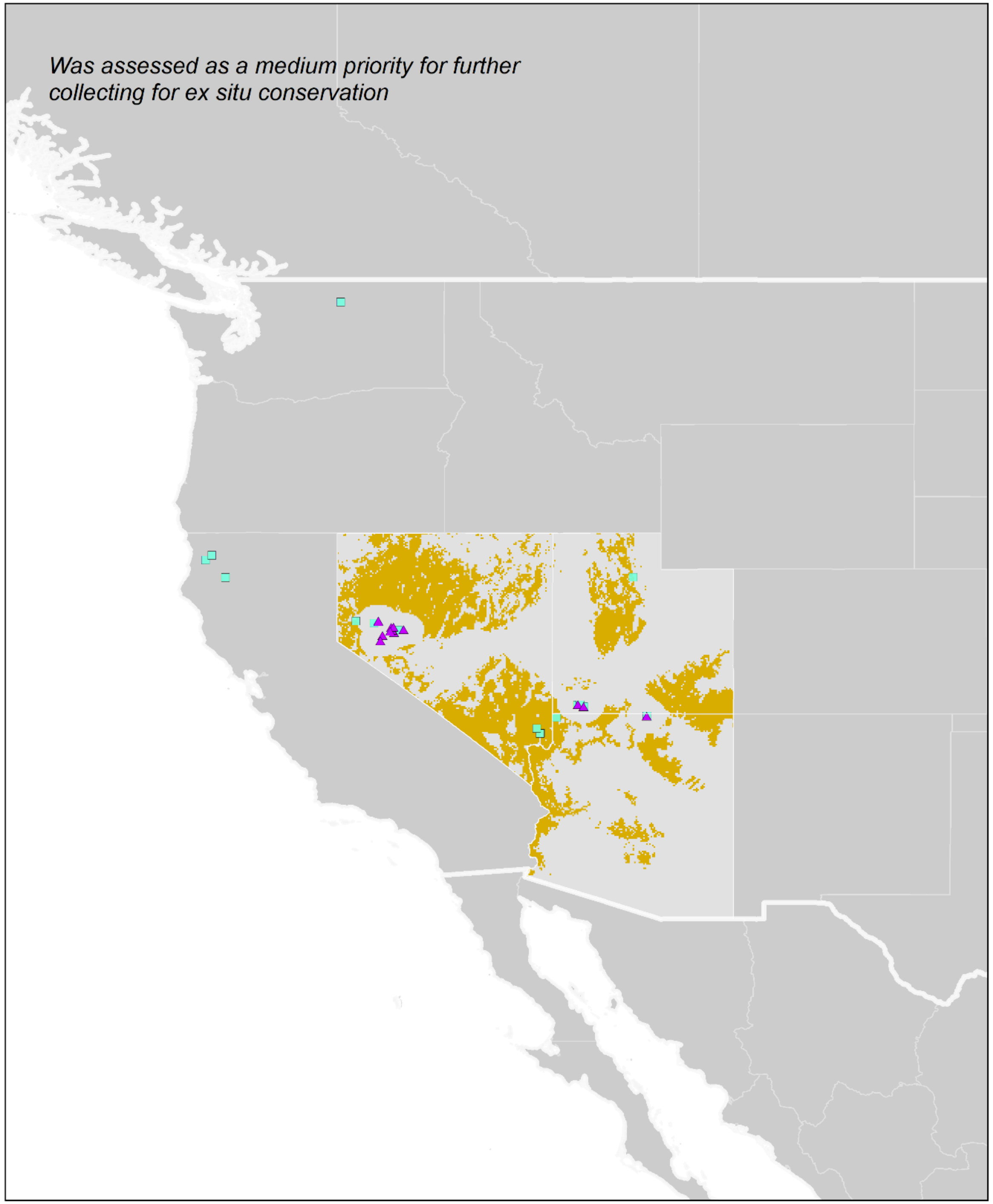
Was assessed as a medium priority for further collecting for ex situ conservation

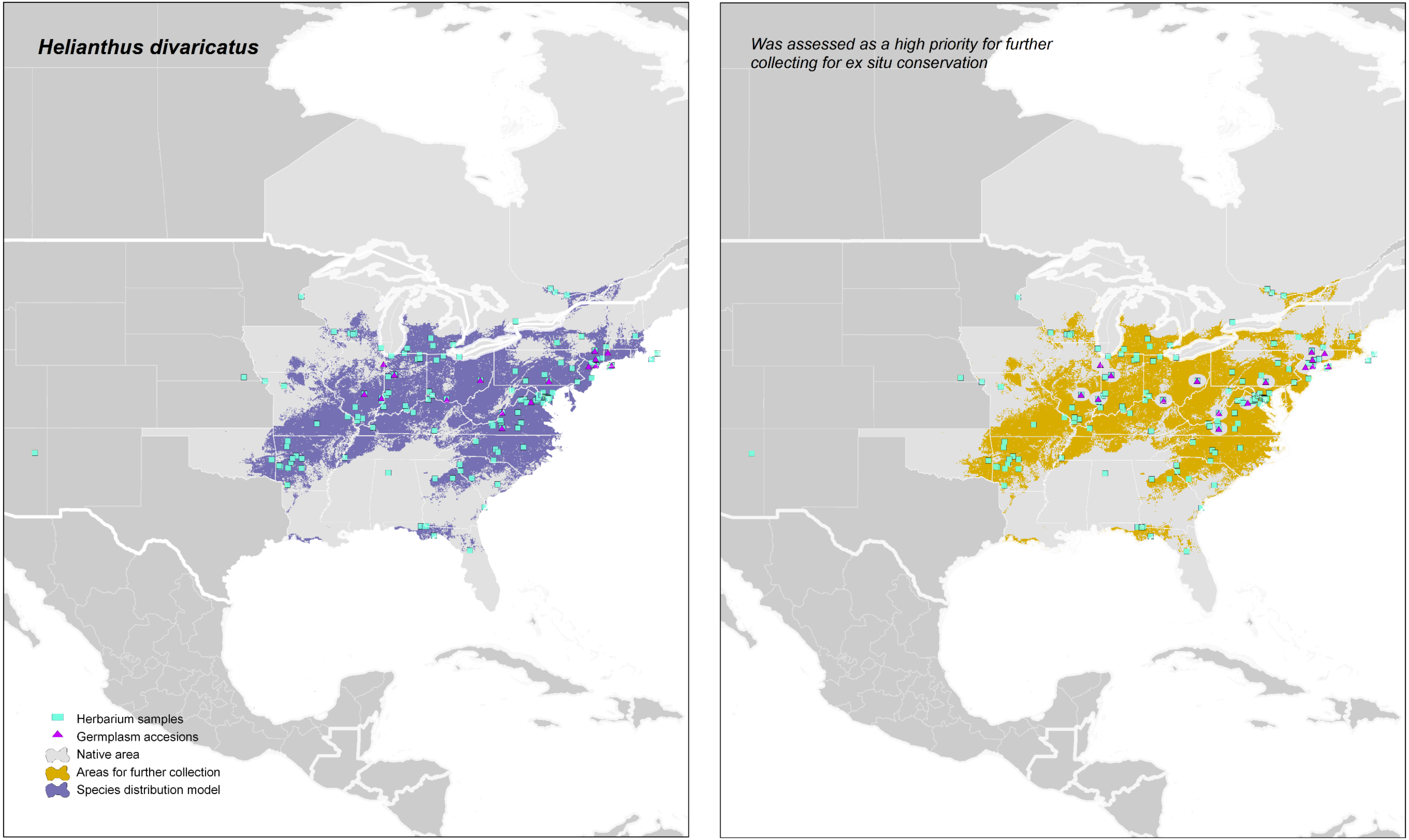
Helianthus divaricatus
Herbarium samples
Germplasm accesions
Native area
Areas for further collection
Species distribution model
Was assessed as a high priority for further collecting for ex situ conservation

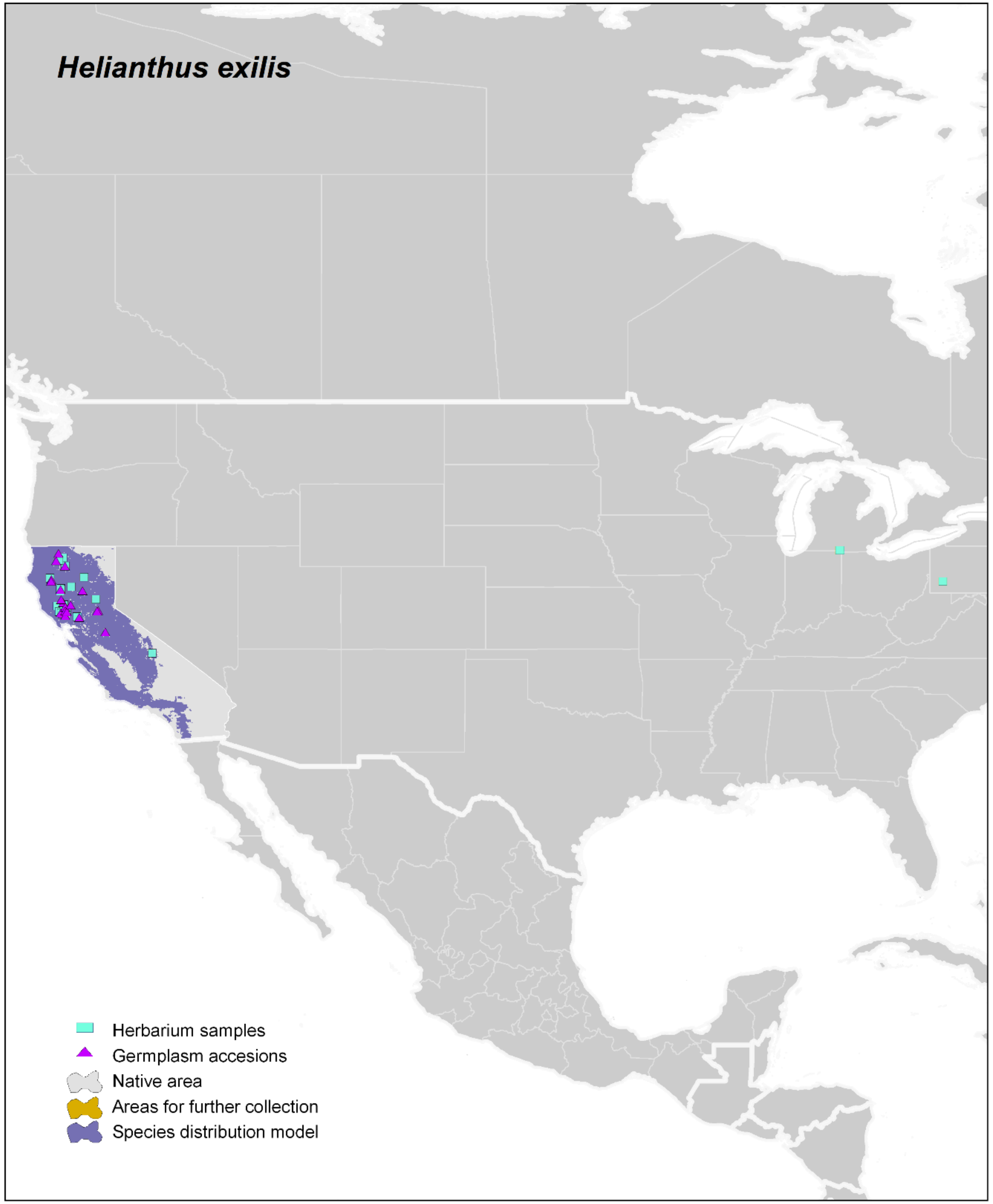
Helianthus exilis
Herbarium samples
Germplasm accesions
Native area
Areas for further collection
Species distribution model

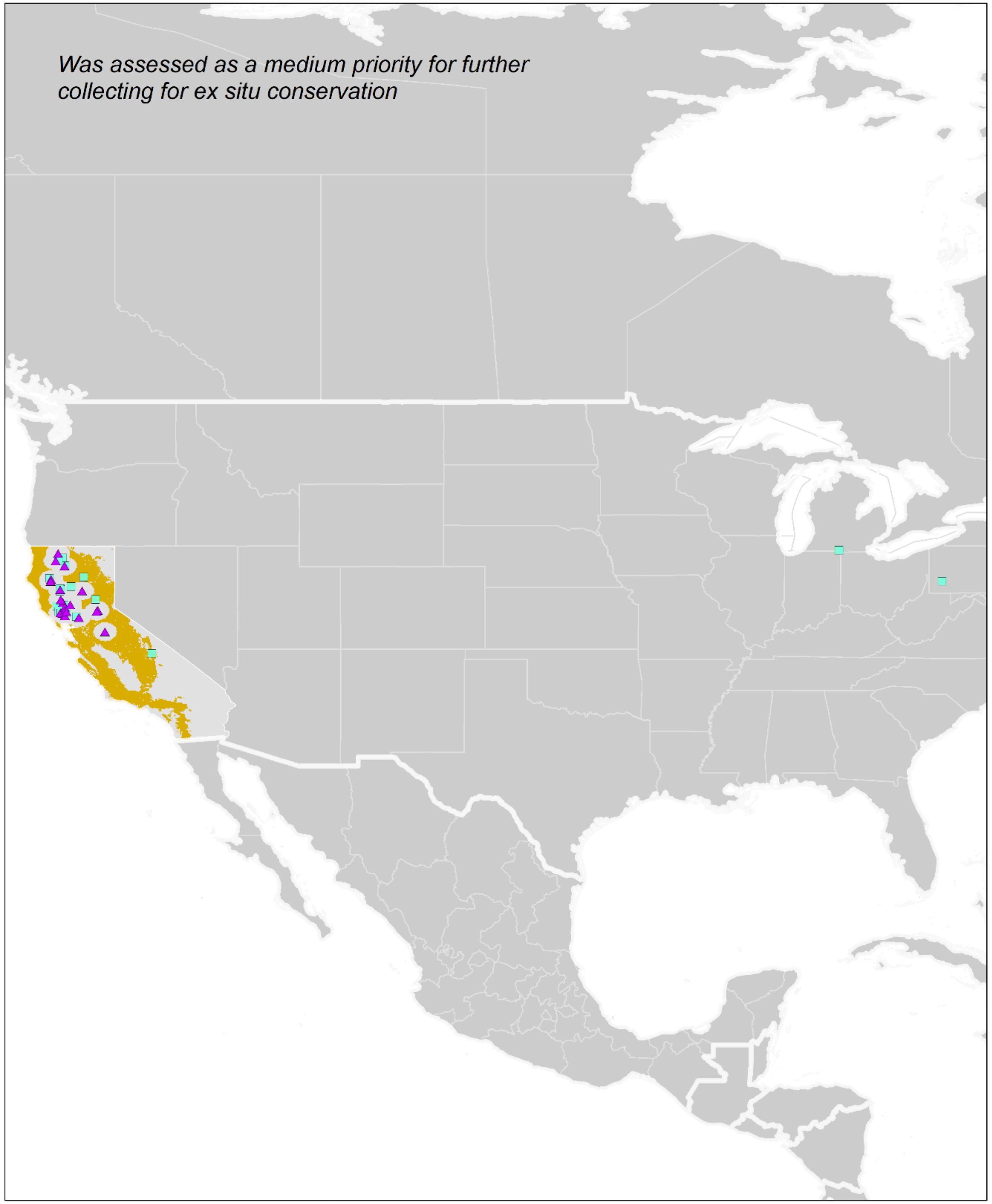
Was assessed as a medium priority for further collecting for ex situ conservation

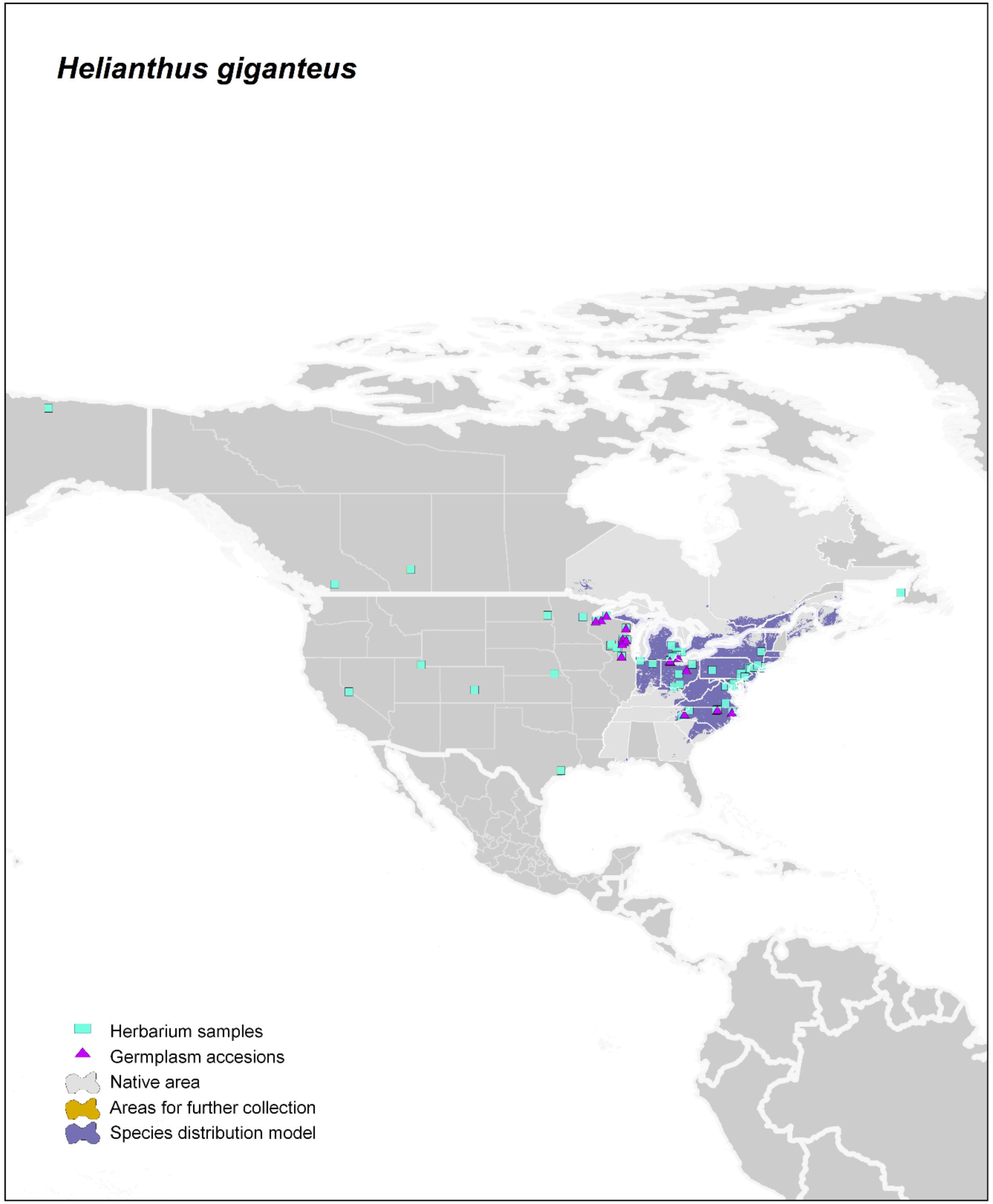
Helianthus giganteus
Herbarium samples
Germplasm accesions
Native area
Areas for further collection
Species distribution model

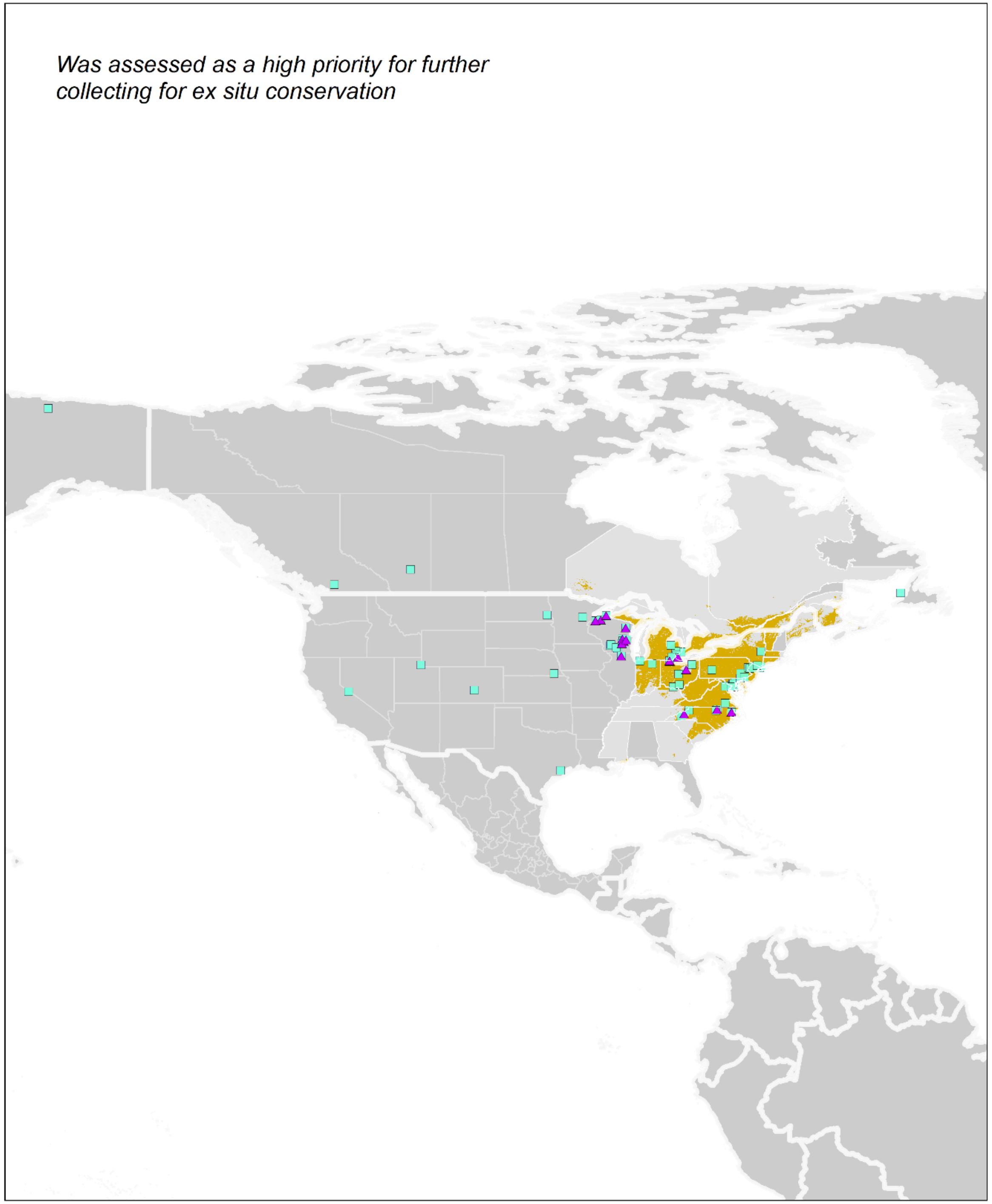
Was assessed as a high priority for further collecting for ex situ conservation

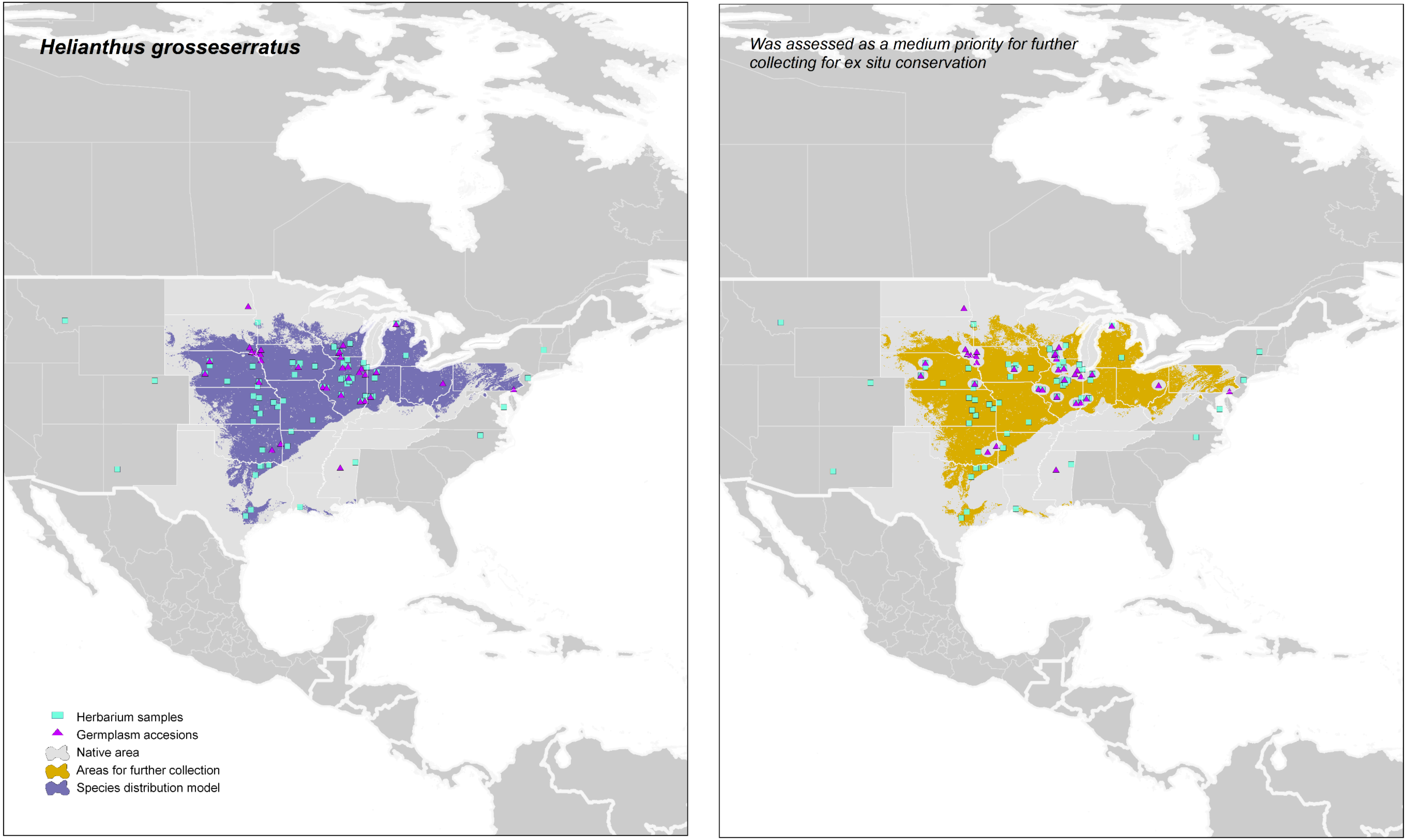
Helianthus grosseserratus
Herbarium samples
Germplasm accesions
Native area
Areas for further collection
Species distribution model
Was assessed as a medium priority for further collecting for ex situ conservation

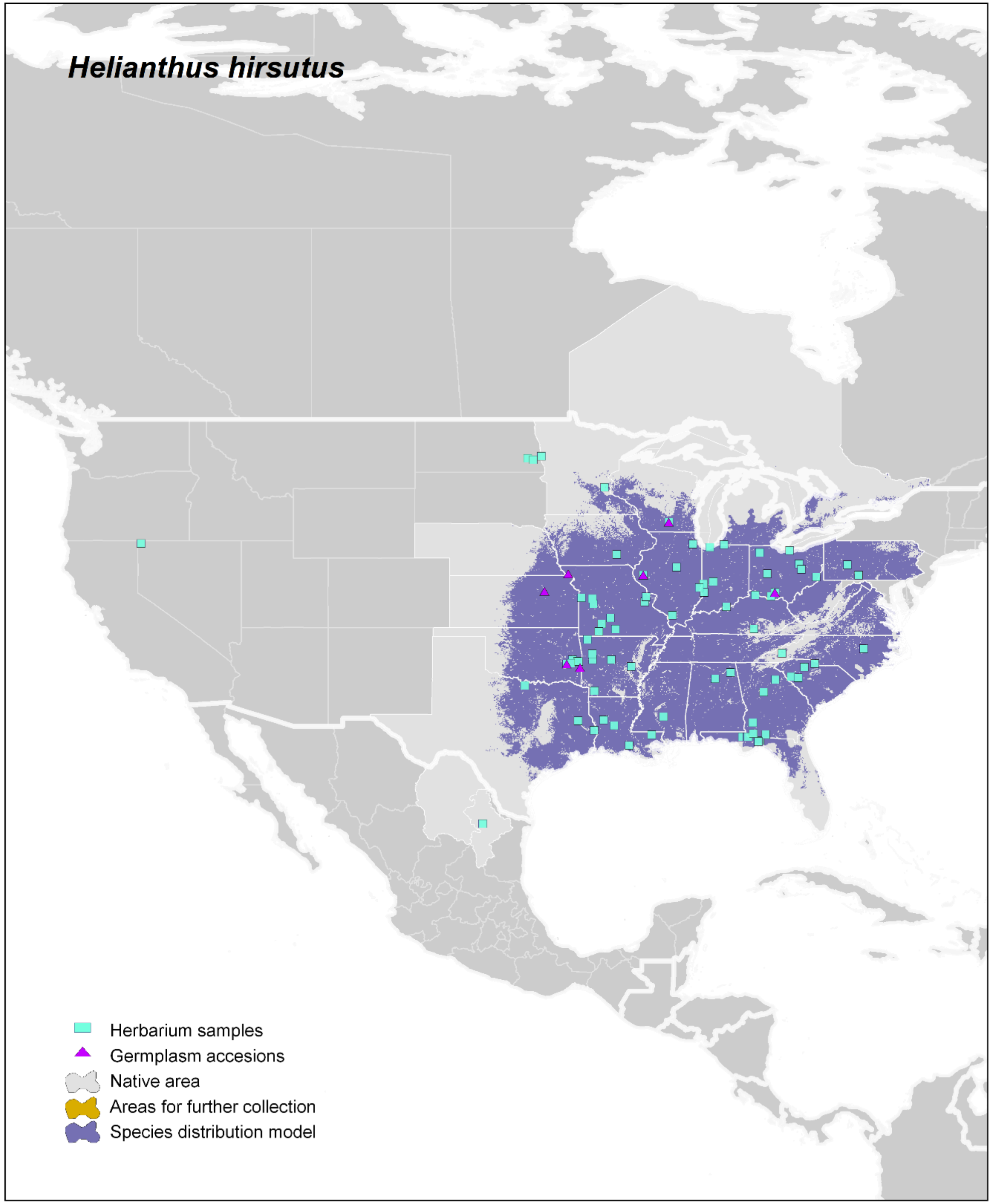
Helianthus hirsutus
Herbarium samples
Germplasm accesions
Native area
Areas for further collection
Species distribution model
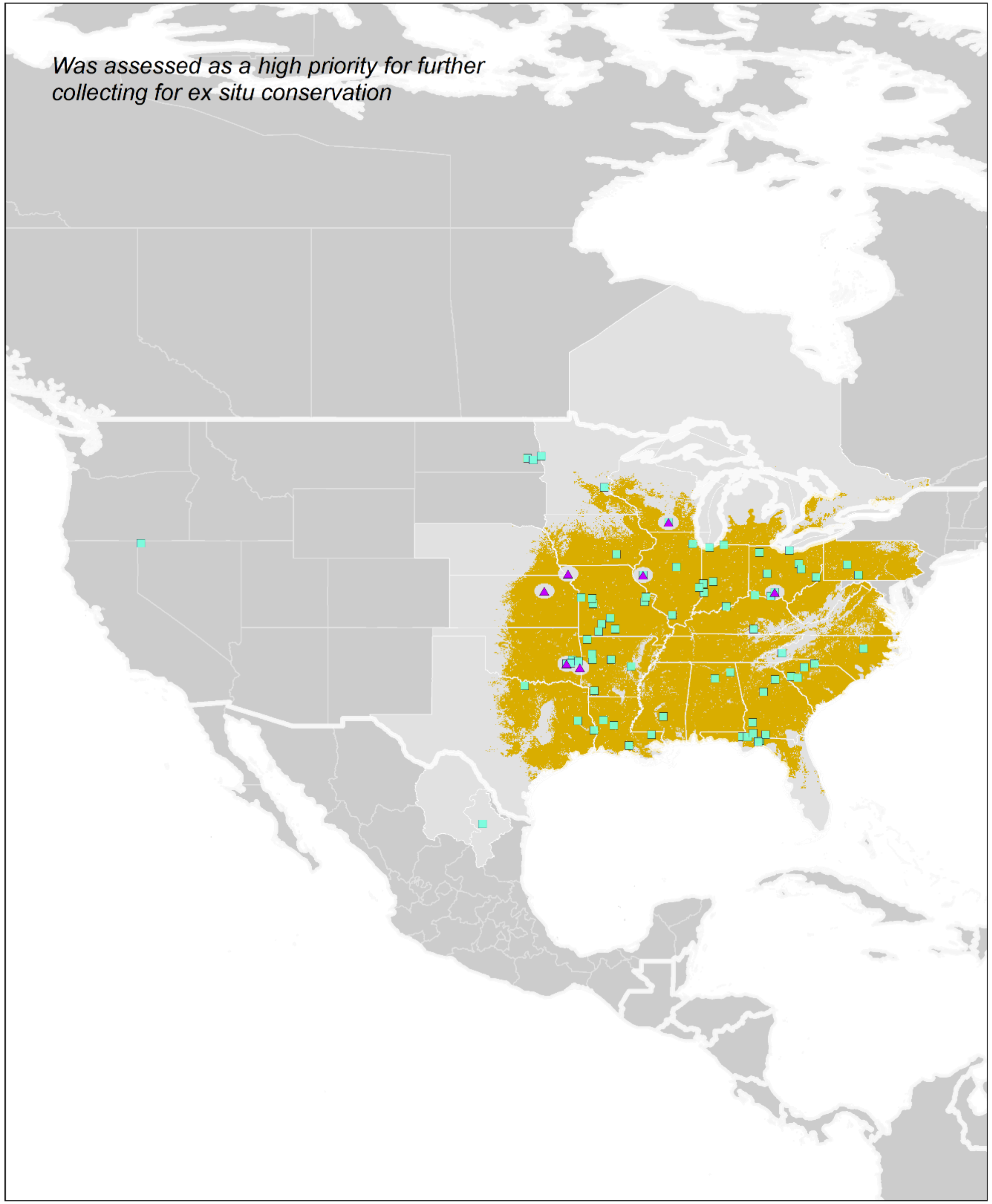
Was assessed as a high priority for further collecting for ex situ conservation

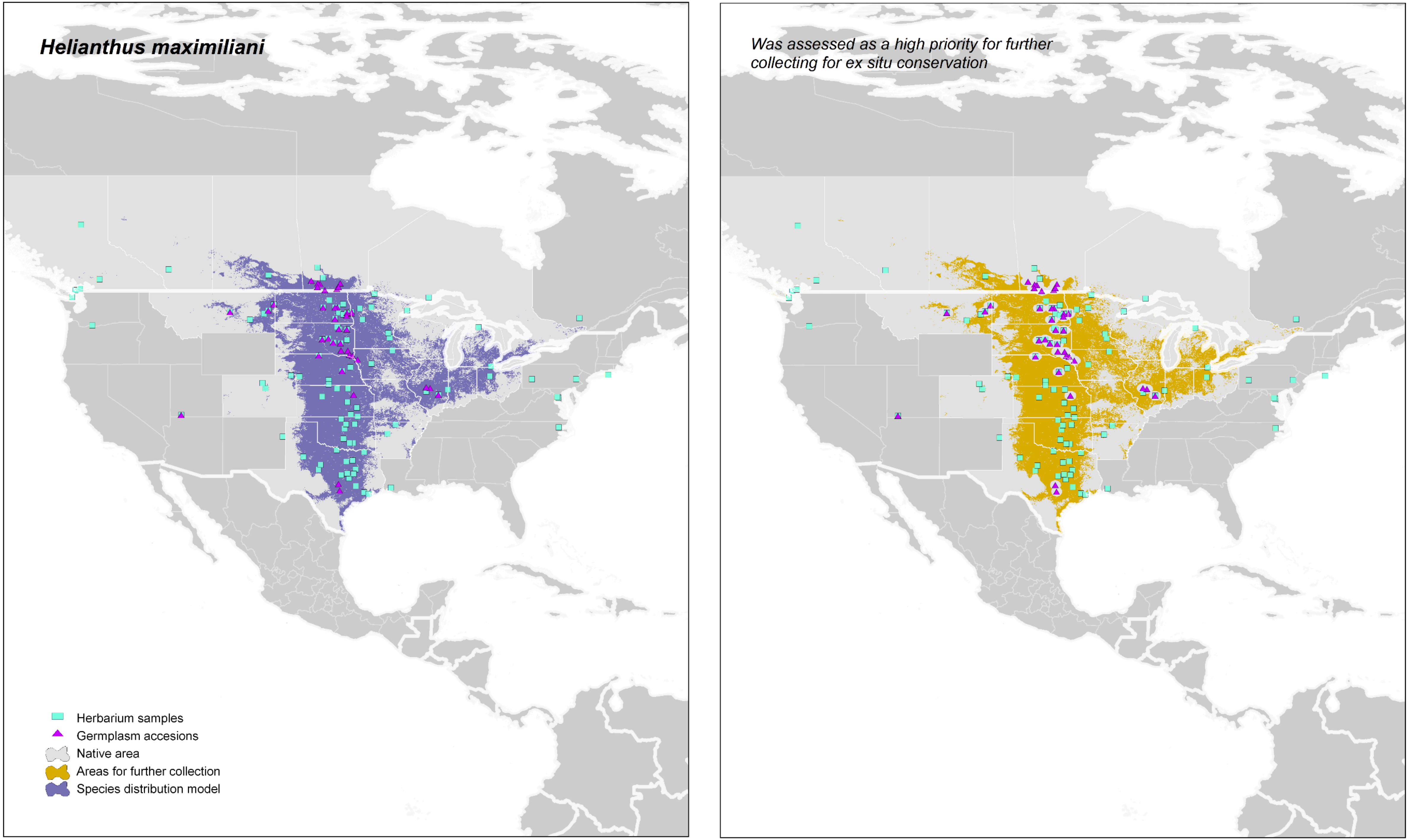
Helianthus maximiliani
Was assessed as a high priority for further collecting for ex situ conservation
Herbarium samples
Germplasm accesions
Native area
Areas for further collection
Species distribution model

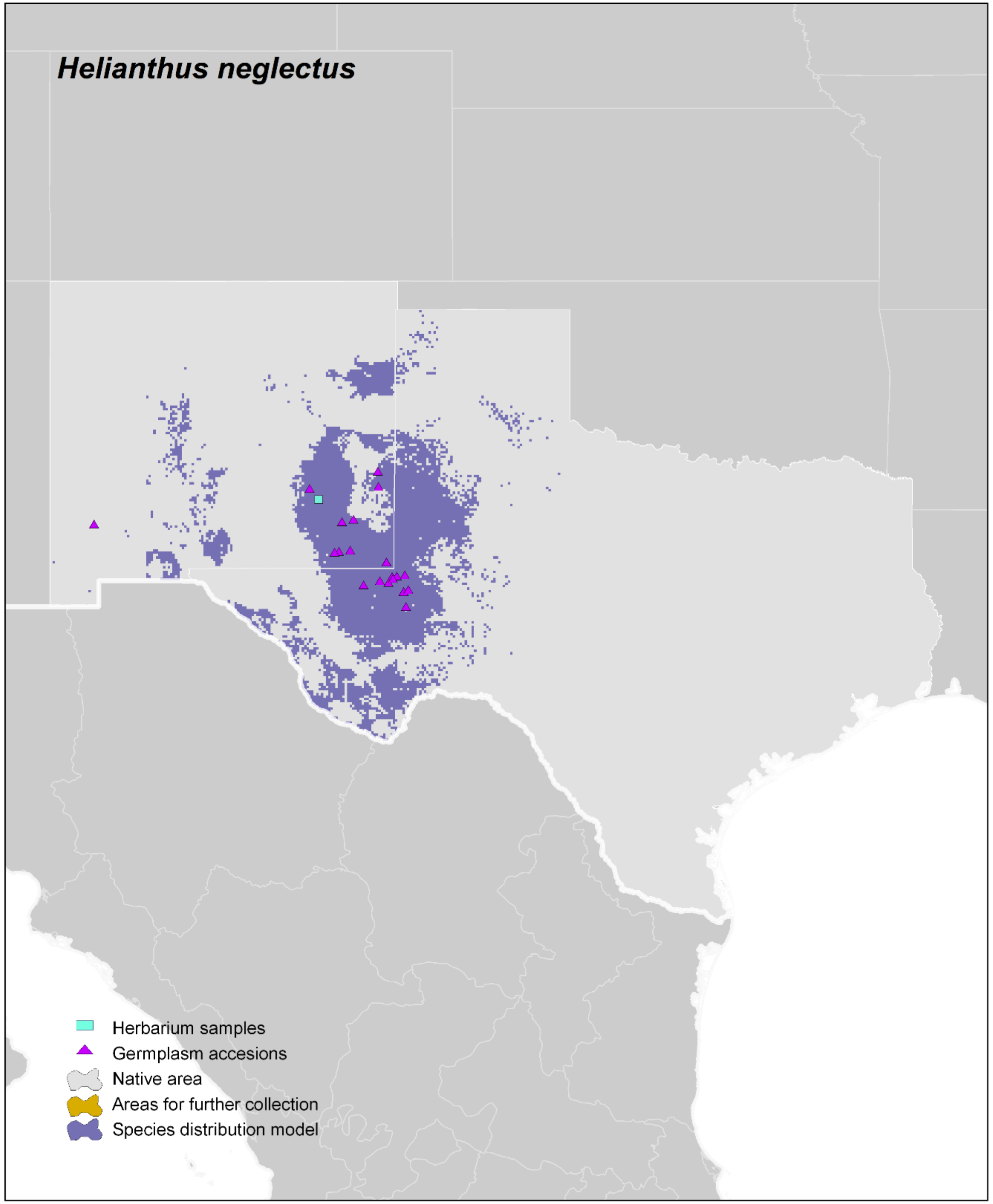
Helianthus neglectus
Herbarium samples
Germplasm accesions
Native area
Areas for further collection
Species distribution model

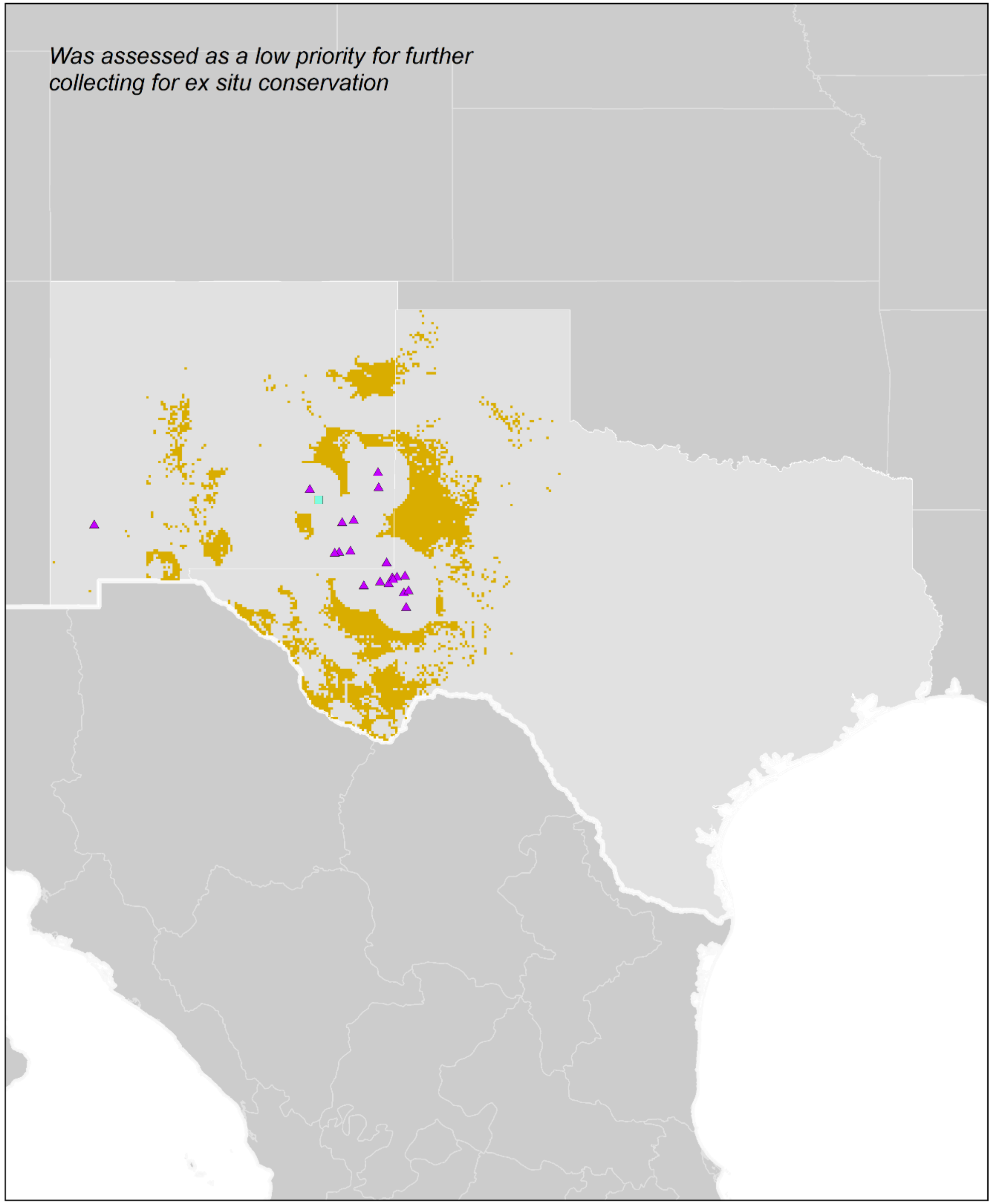
Was assessed as a low priority for further collecting for ex situ conservation

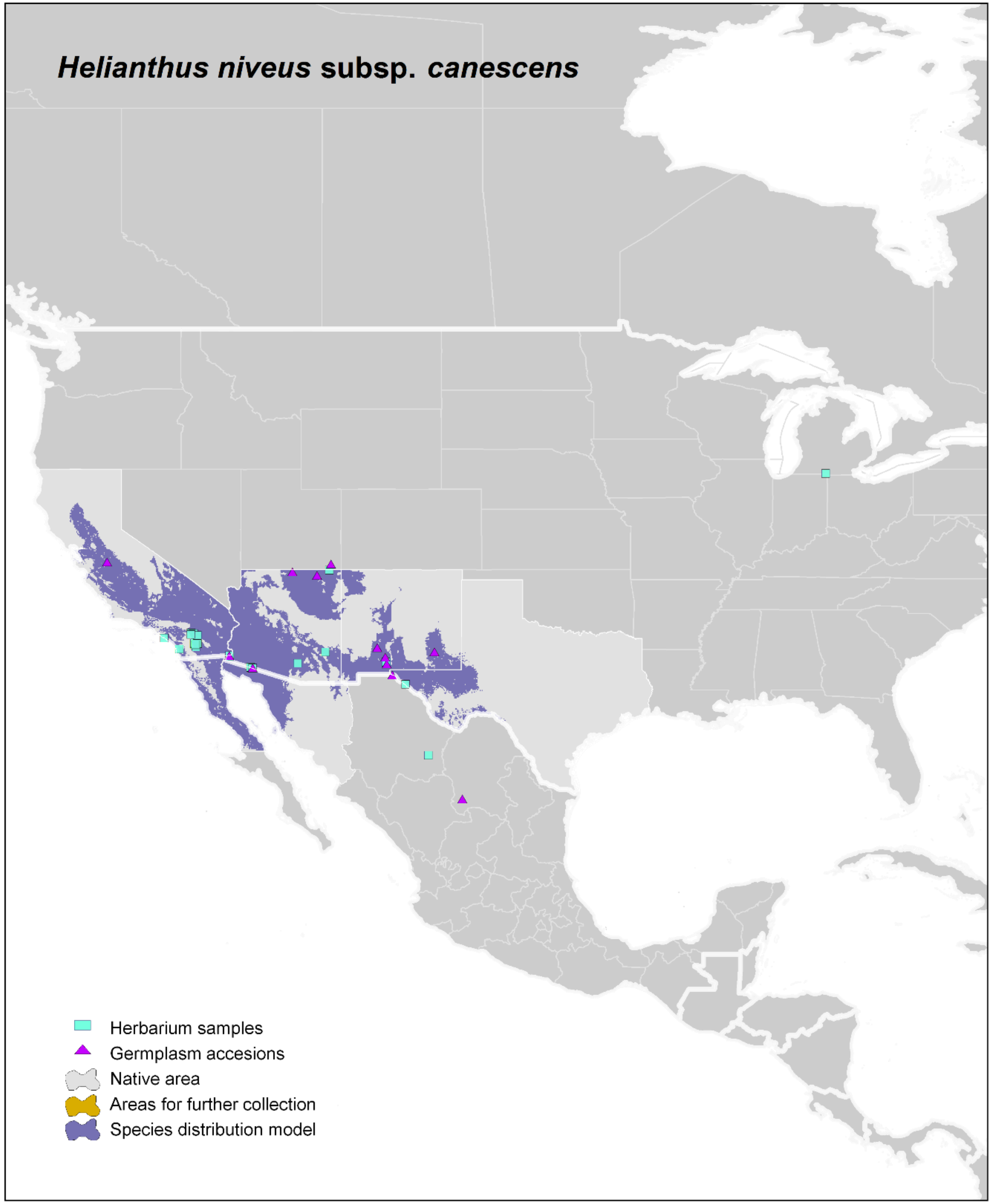
Helianthus niveus subsp. *canescens*
Herbarium samples
Germplasm accesions
Native area
Areas for further collection
Species distribution model

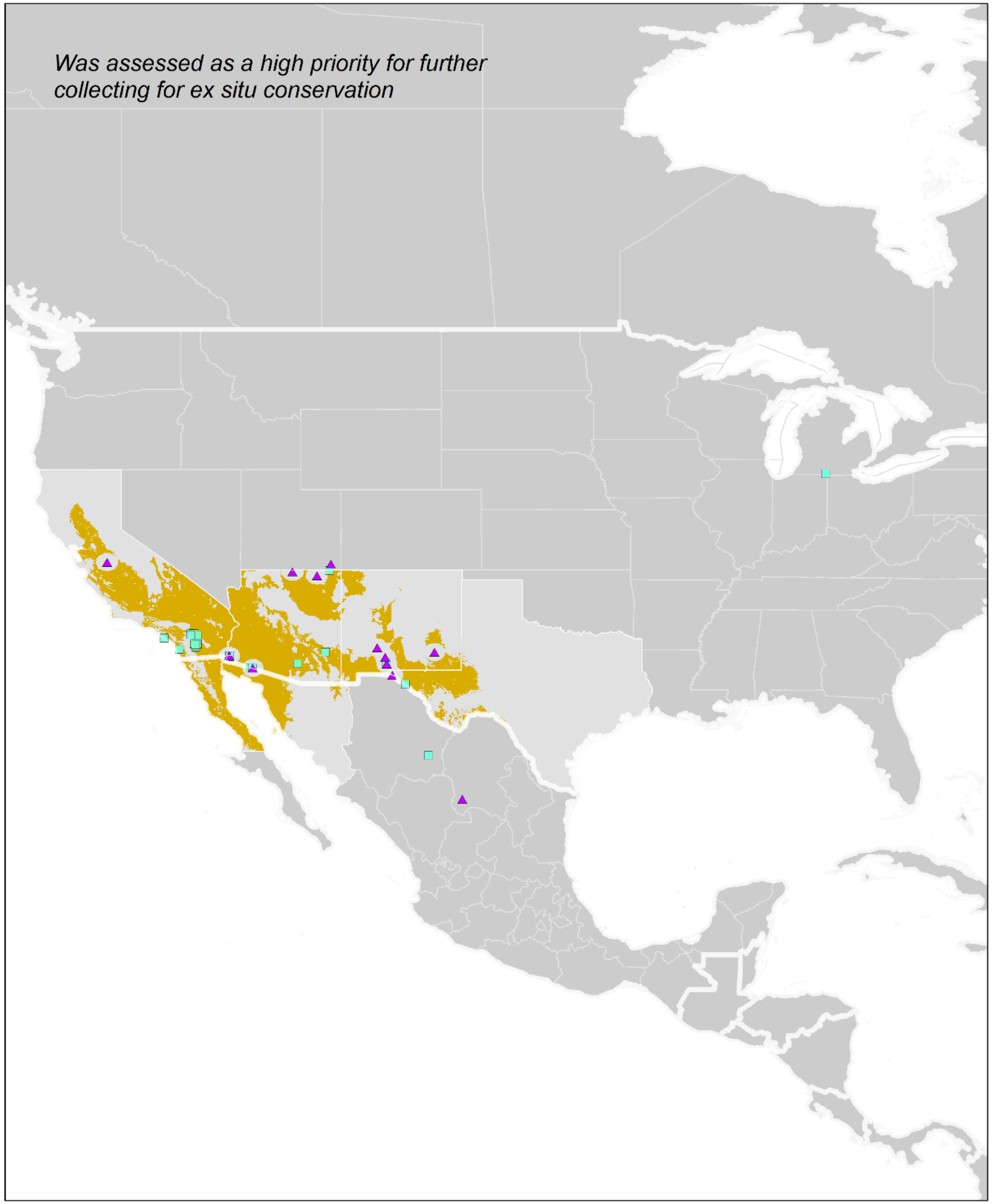
Was assessed as a high priority for further collecting for ex situ conservation

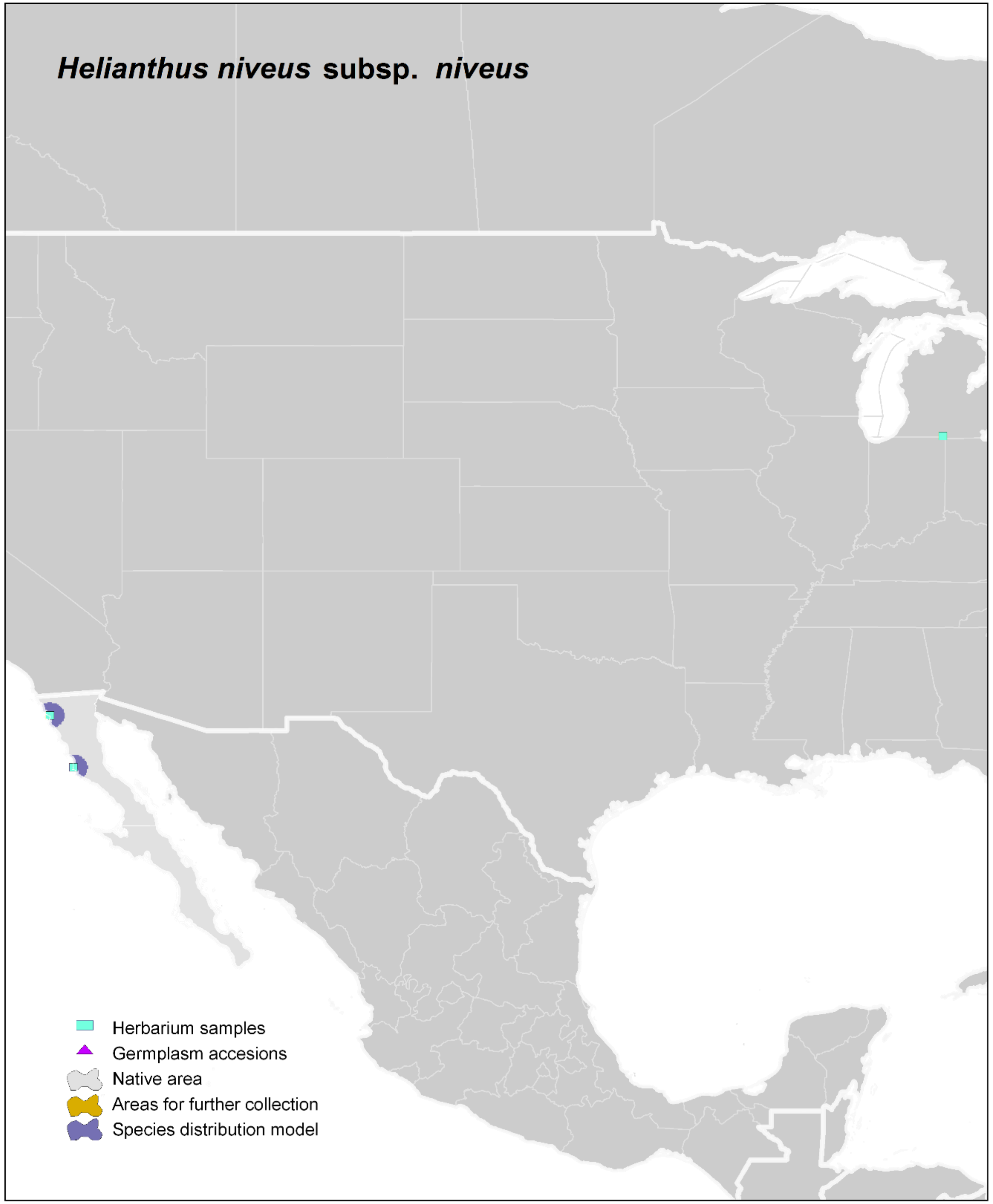
Helianthus niveus subsp. niveus
Herbarium samples
Germplasm accesions
Native area
Areas for further collection
Species distribution model
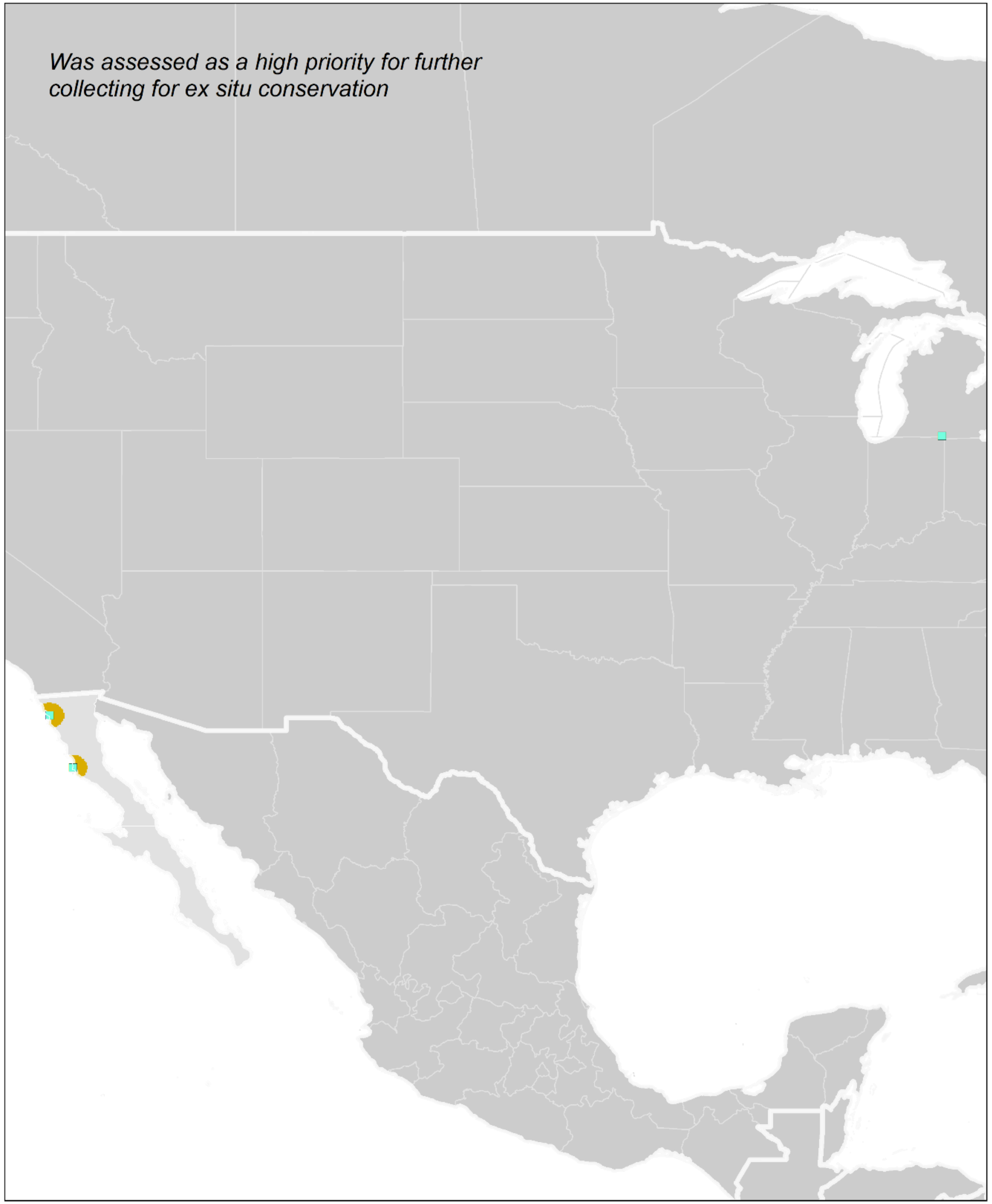
Was assessed as a high priority for further collecting for ex situ conservation

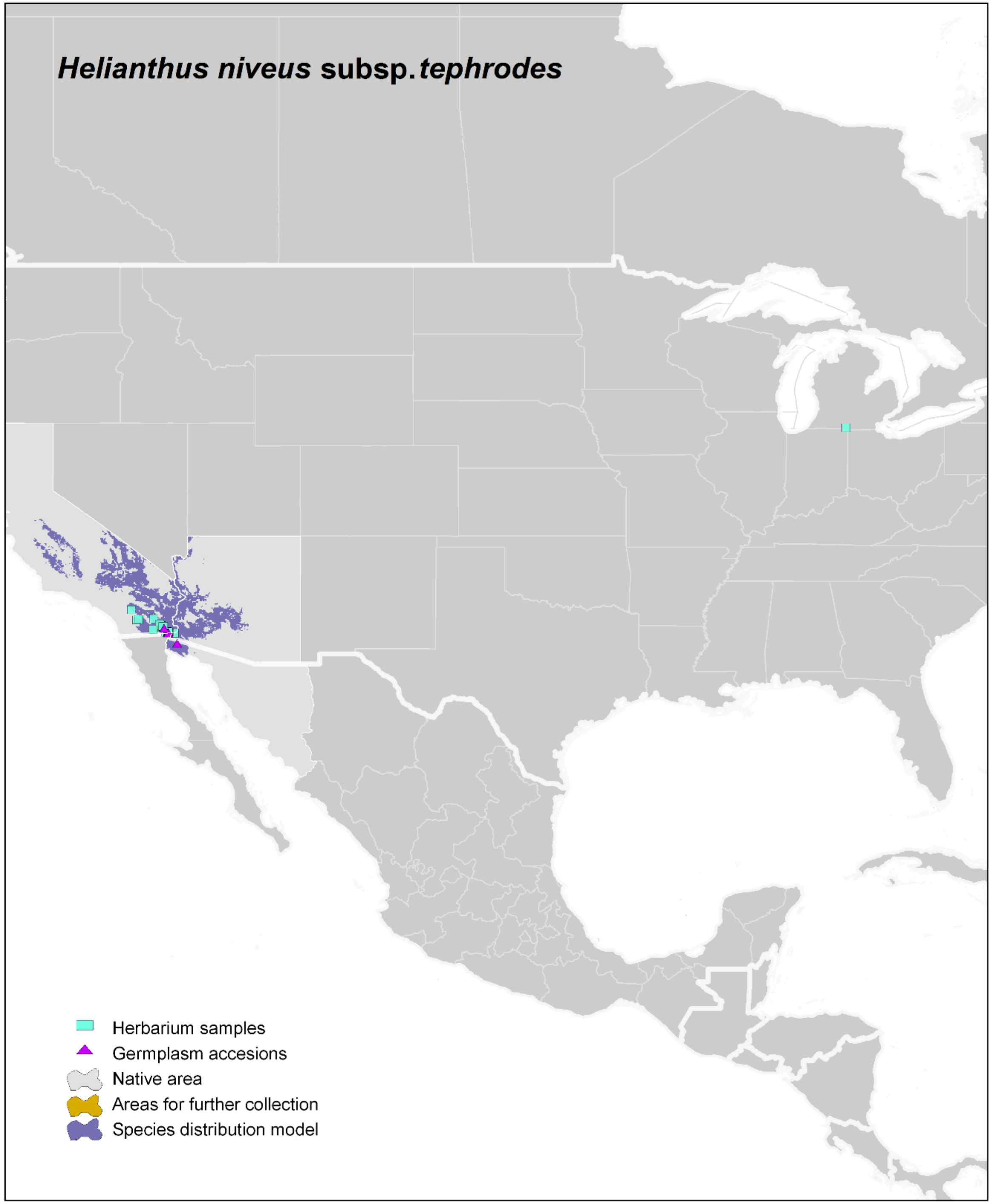
Helianthus niveus subsp.*tephrodes*
Herbarium samples
Germplasm accesions
Native area
Areas for further collection
Species distribution model

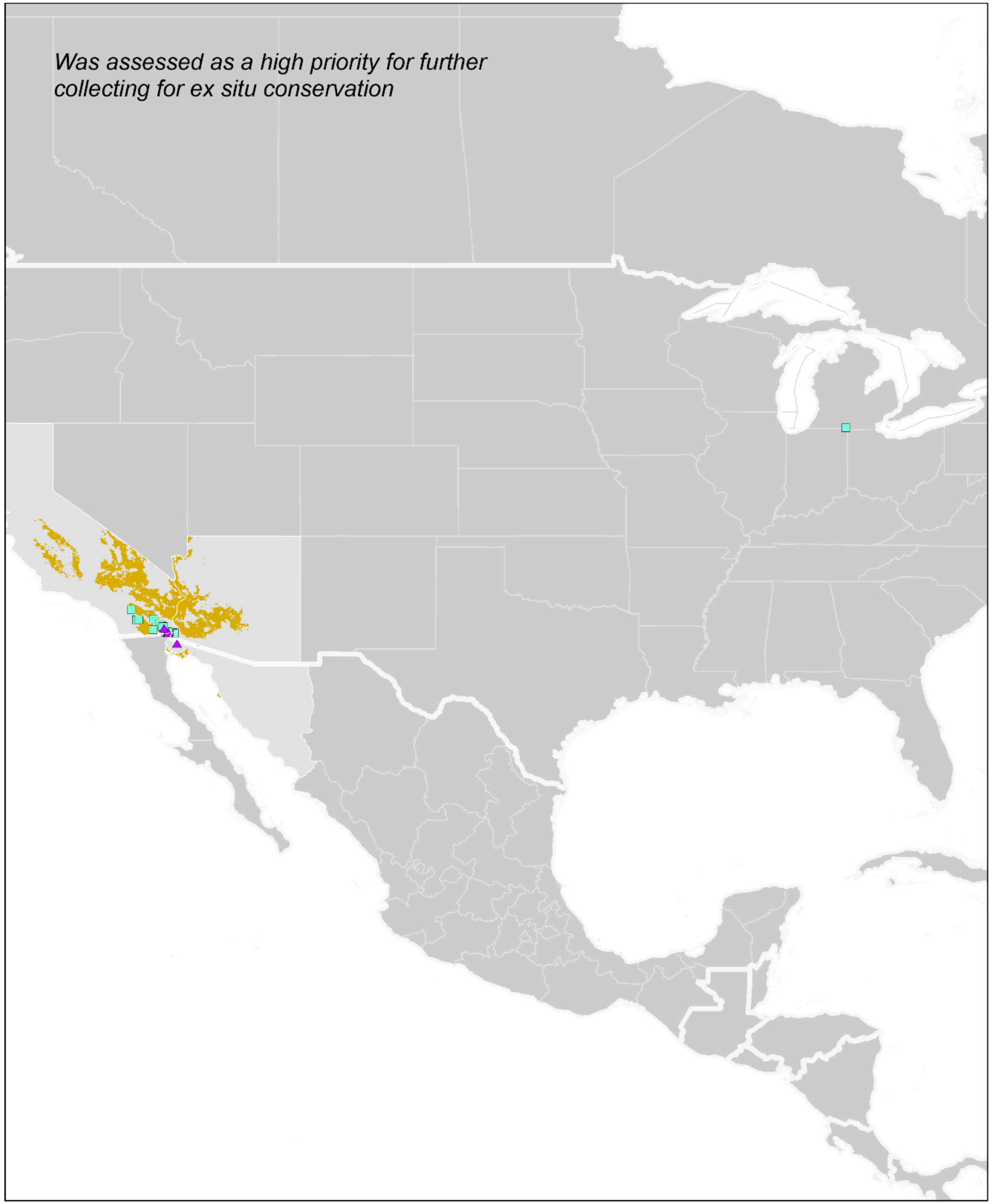
Was assessed as a high priority for further collecting for ex situ conservation

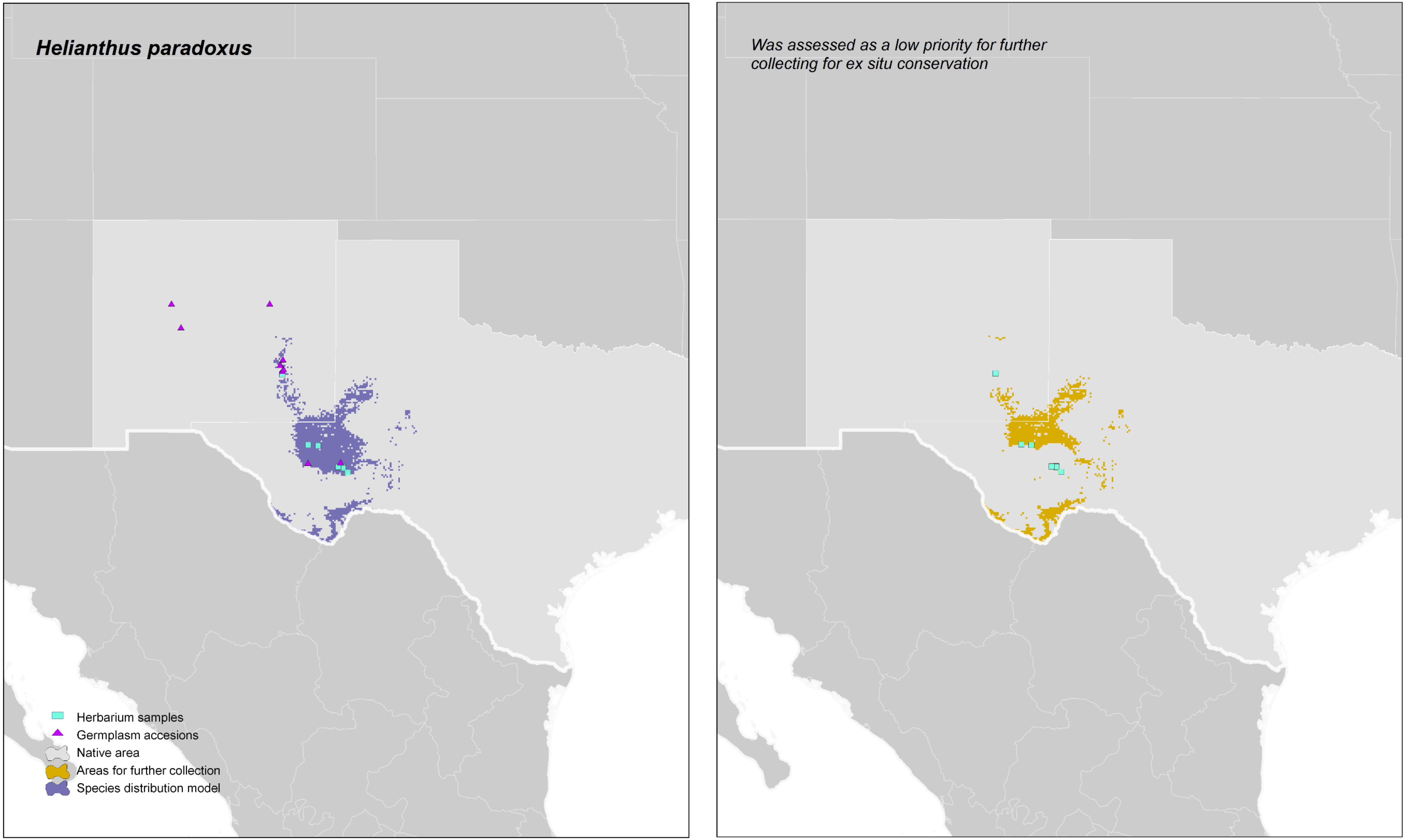
Helianthus paradoxus
Herbarium samples
Germplasm accesions
Native area
Areas for further collection
Species distribution model
Was assessed as a low priority for further
collecting for ex situ conservation

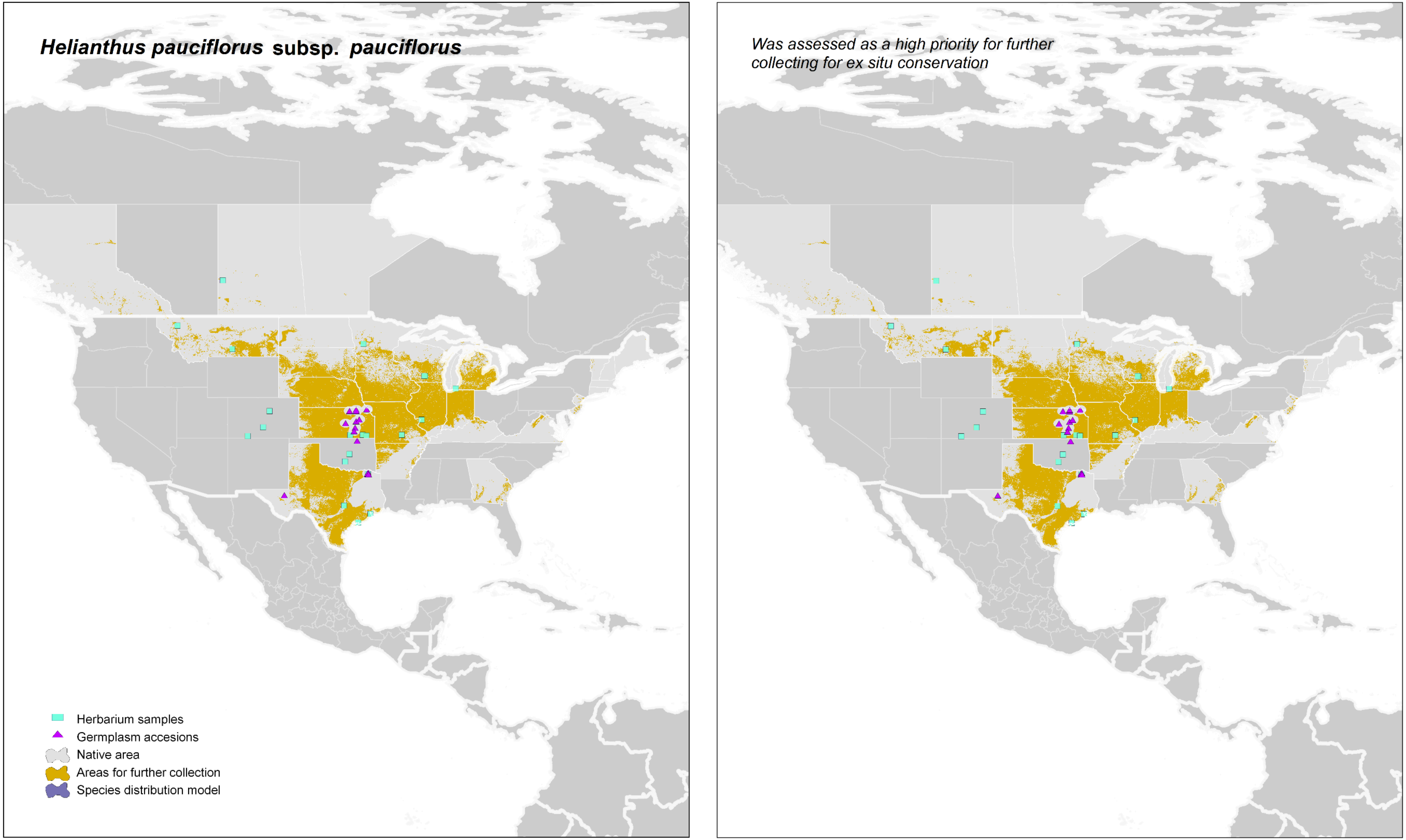
Helianthus pauciflorus subsp. *pauciflorus*
Herbarium samples
Germplasm accesions
Native area
Areas for further collection
Species distribution model
Was assessed as a high priority for further collecting for ex situ conservation

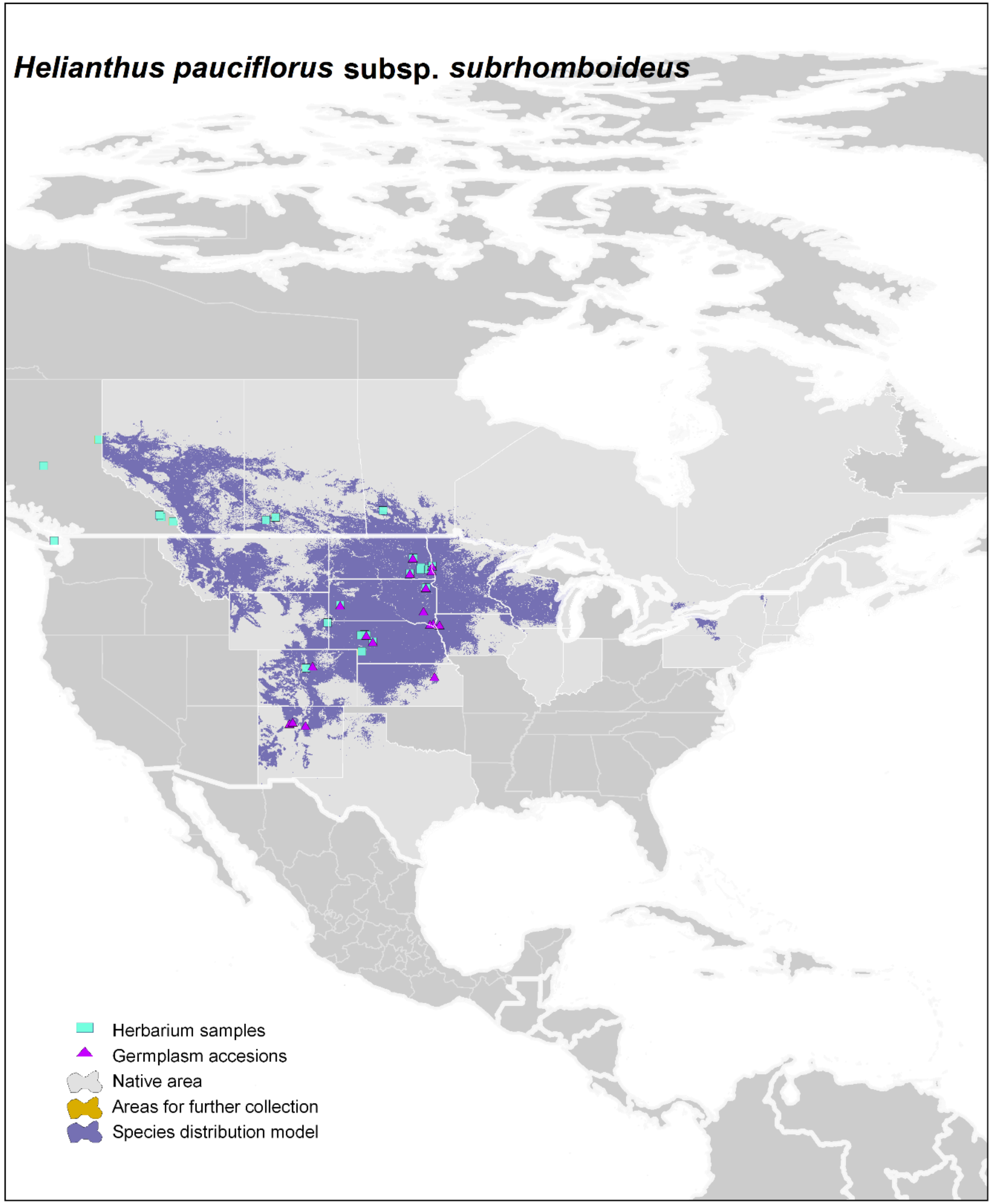
Helianthus pauciflorus subsp. *subrhomboideus*
Herbarium samples
Germplasm accesions
Native area
Areas for further collection
Species distribution model

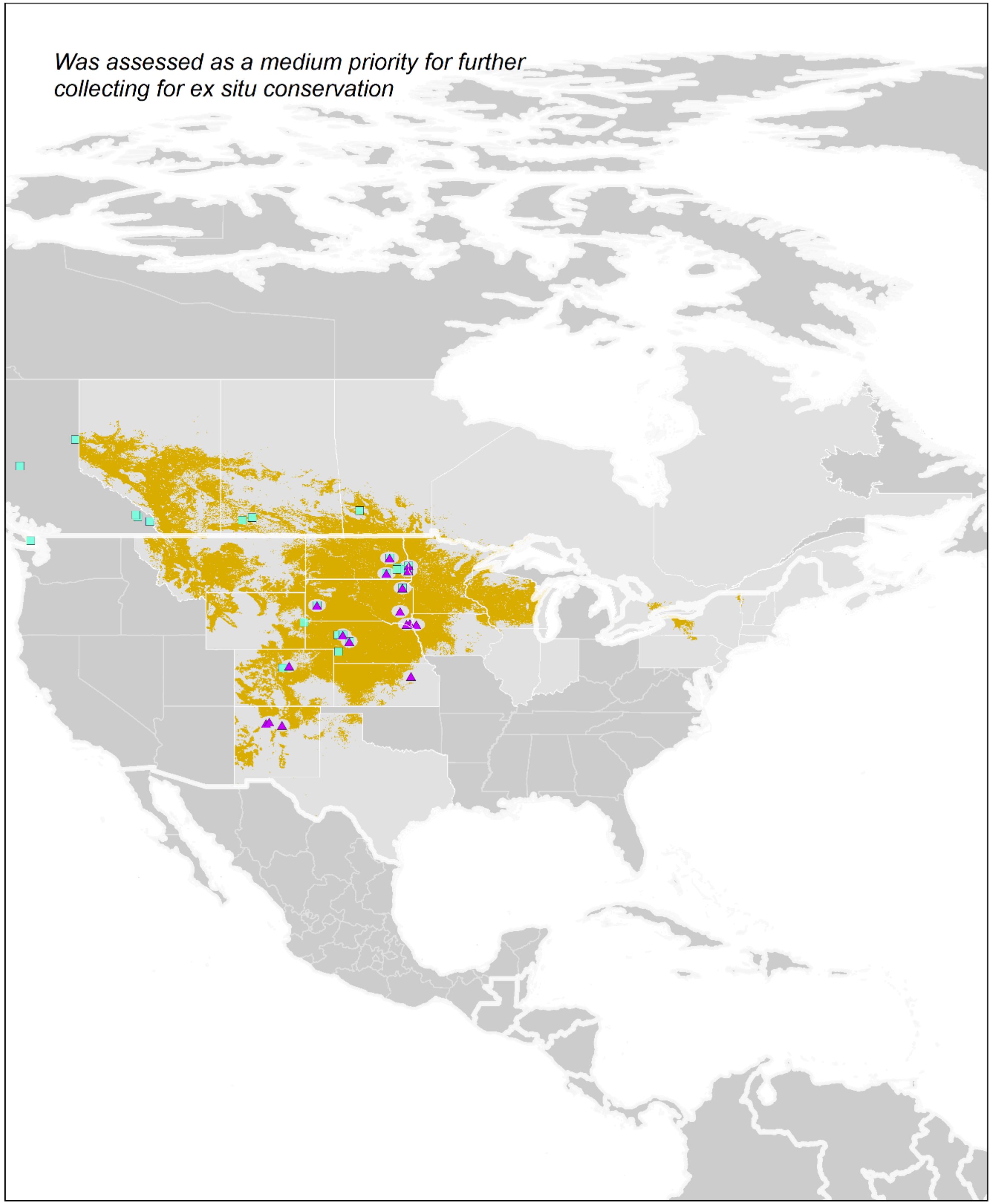
Was assessed as a medium priority for further collecting for ex situ conservation

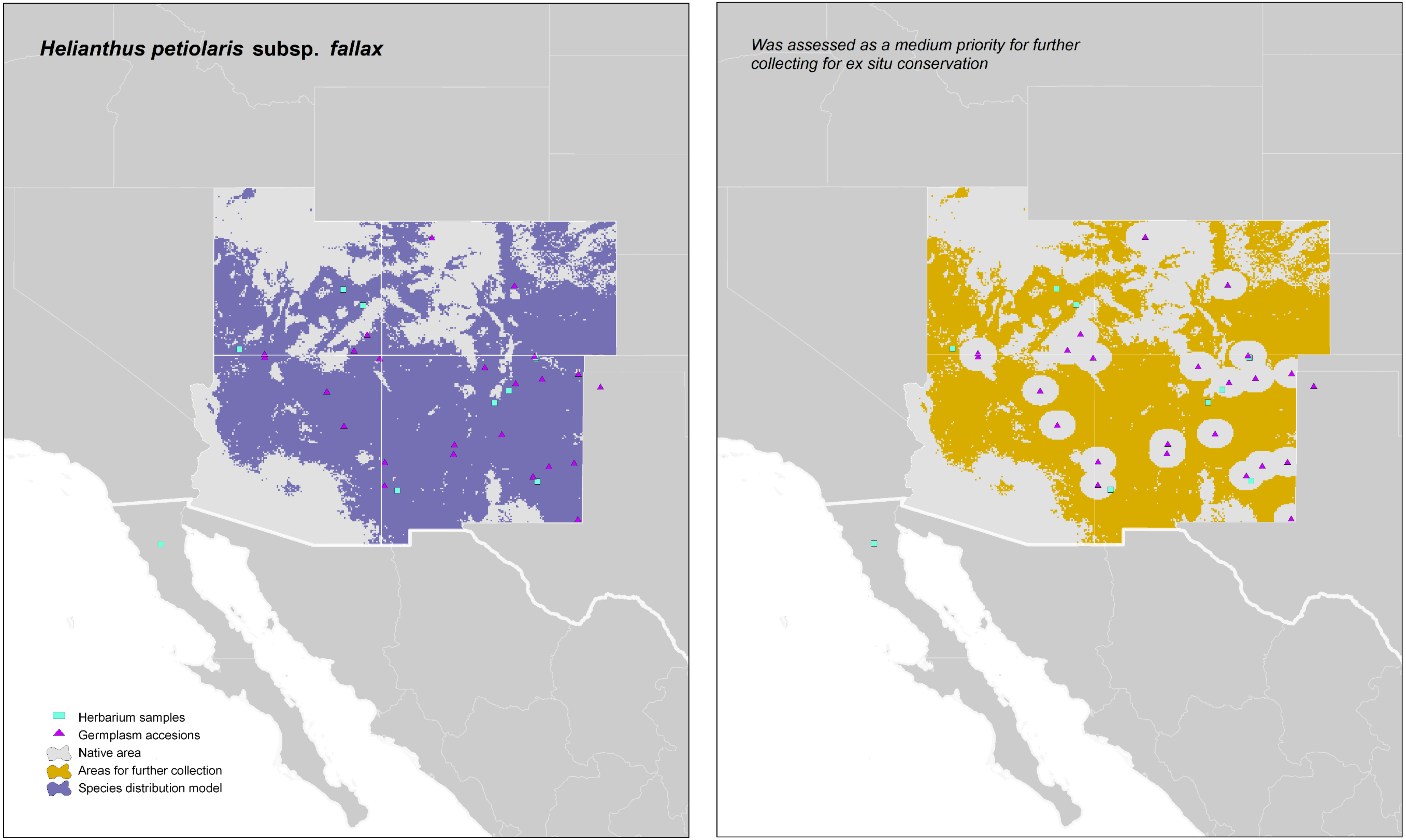
Helianthus petiolaris subsp. fallax
Was assessed as a medium priority for further collecting for ex situ conservation
Herbarium samples
Germplasm accesions
Native area
Areas for further collection
Species distribution model

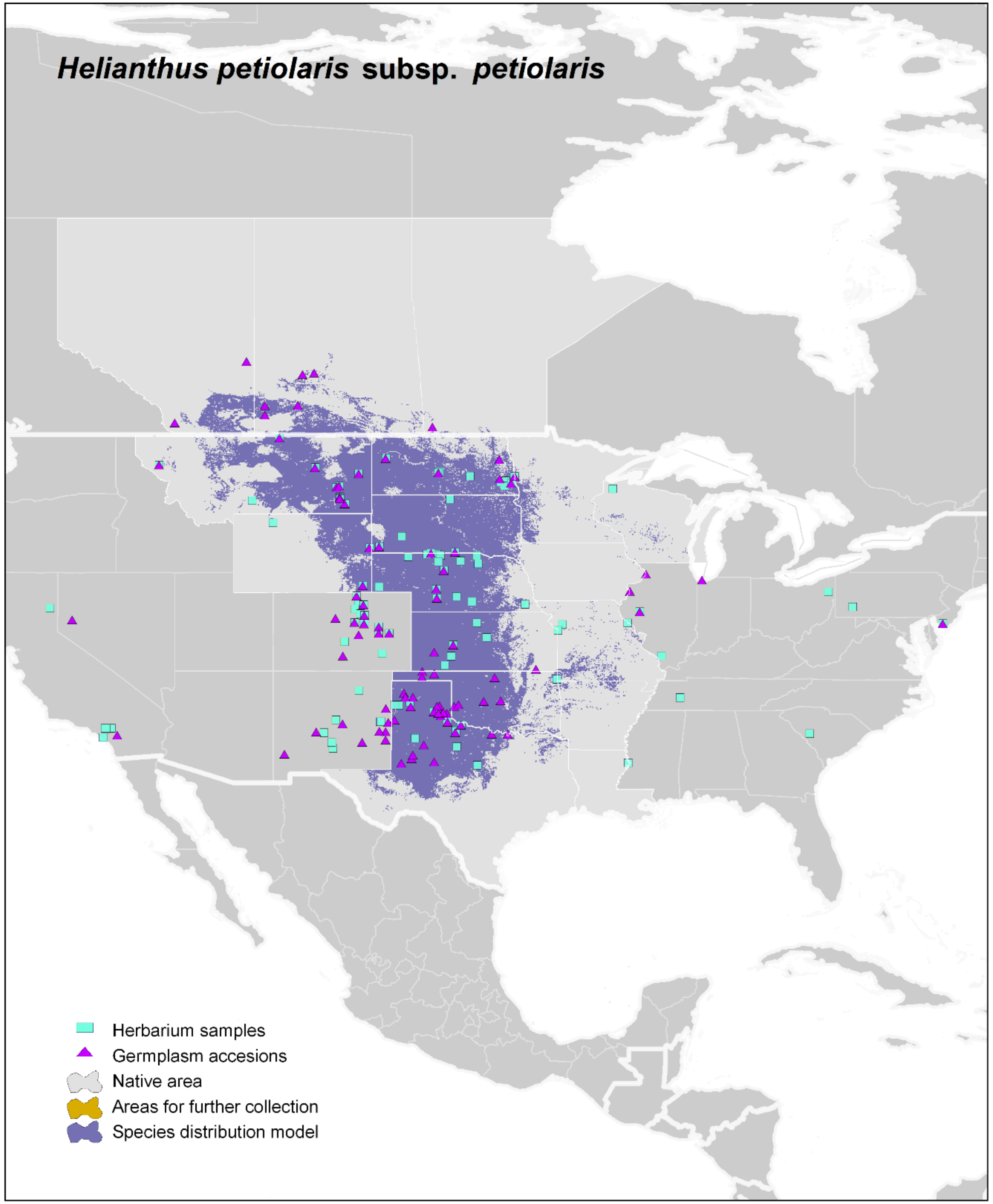

Helianthus petiolaris subsp. petiolaris
Herbarium samples
Germplasm accesions
Native area
Areas for further collection
Species distribution model

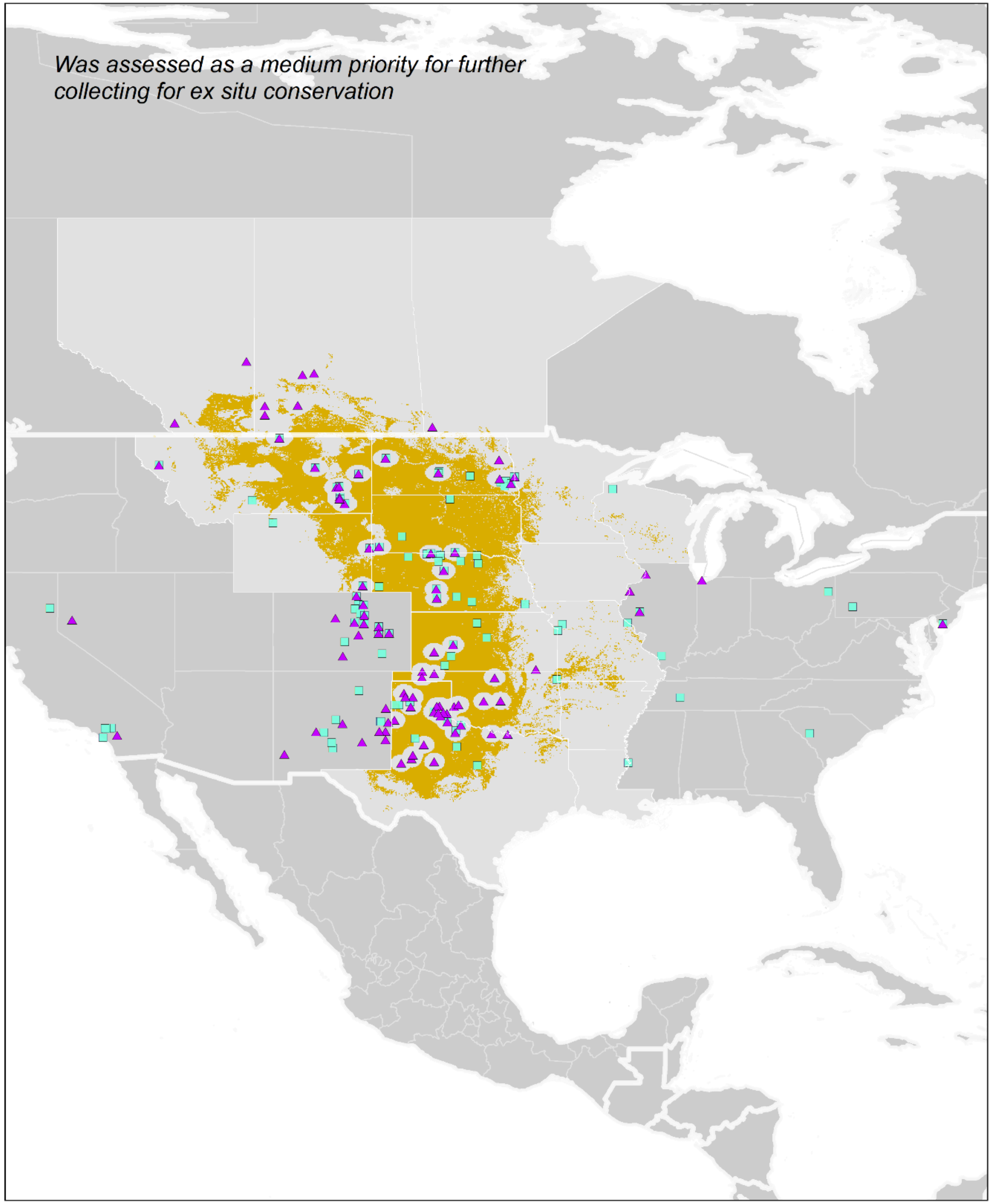

Was assessed as a medium priority for further collecting for ex situ conservation

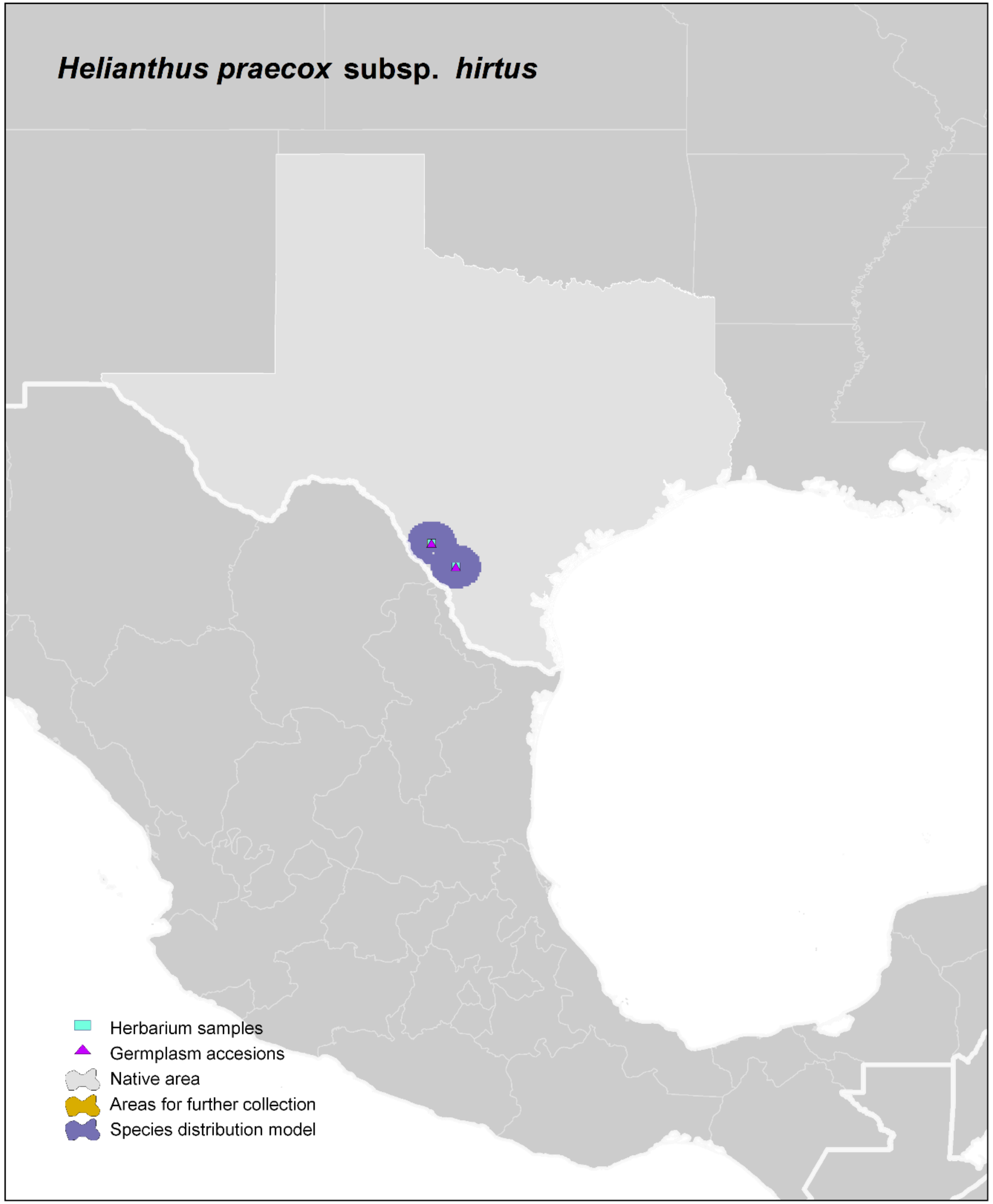
Helianthus praecox subsp. *hirtus*
Herbarium samples
Germplasm accesions
Native area
Areas for further collection
Species distribution model

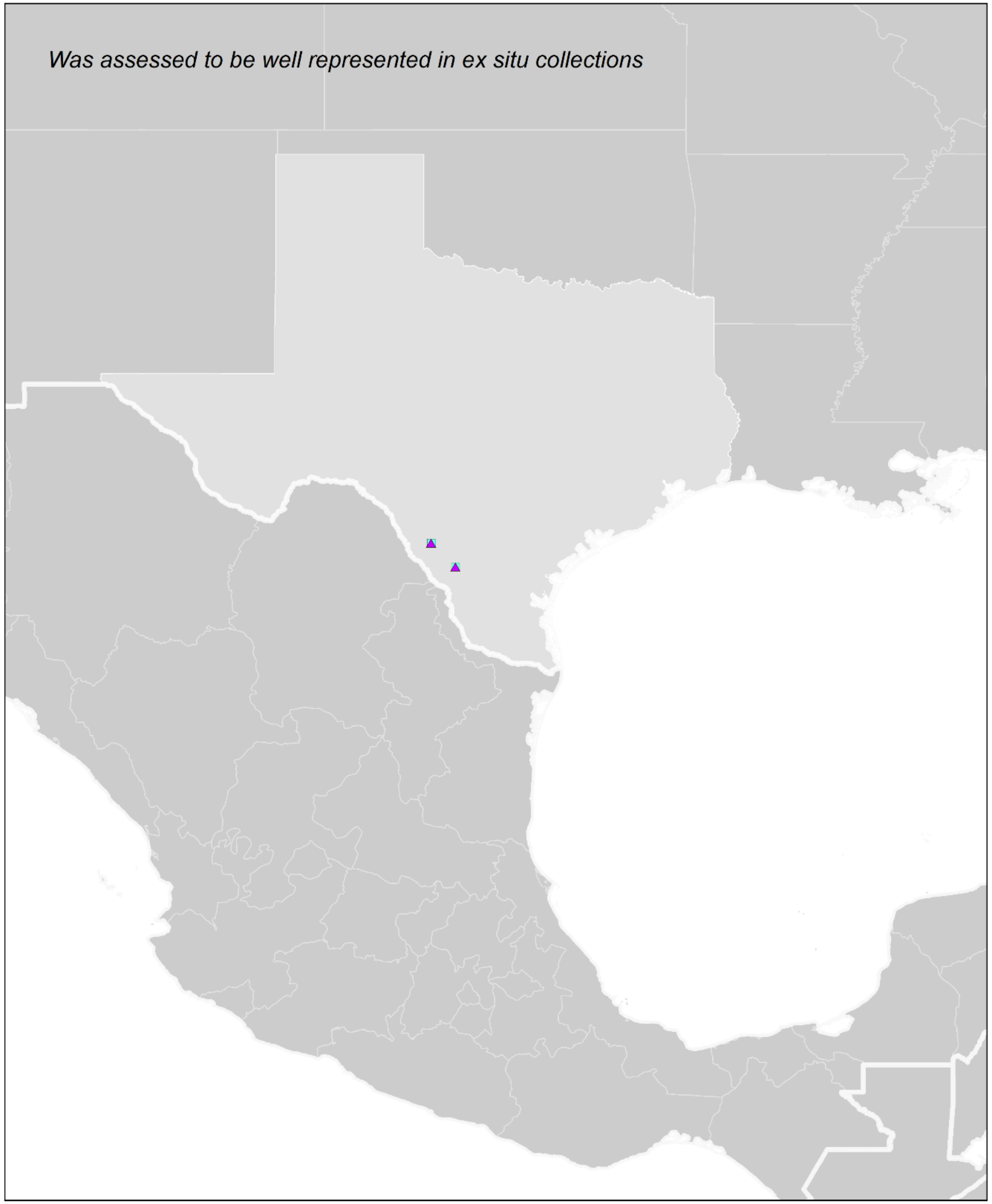
Was assessed to be well represented in ex situ collections

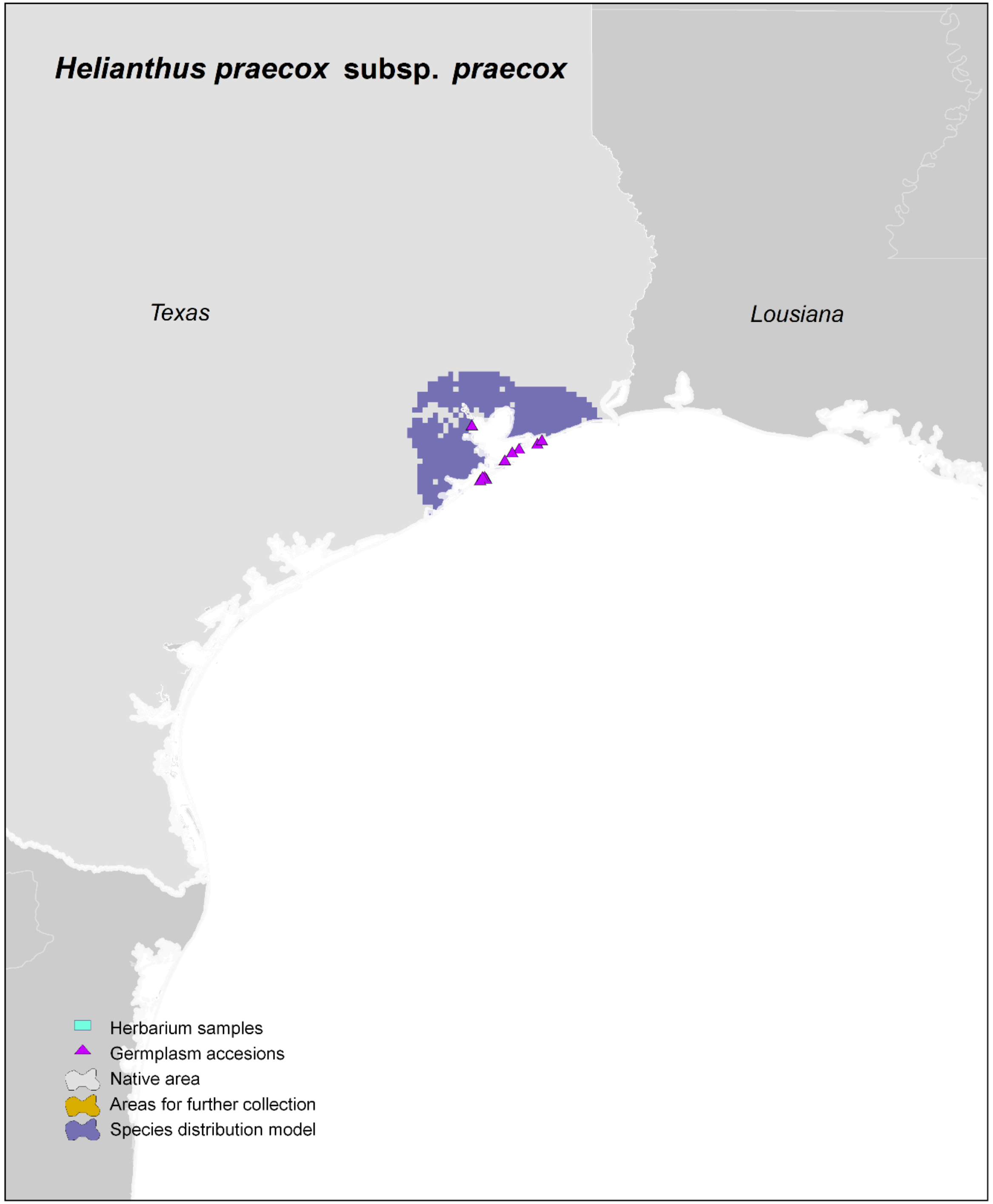
Helianthus praecox subsp. *praecox*
Texas
Lousiana
Herbarium samples
Germplasm accesions
Native area
Areas for further collection
Species distribution model

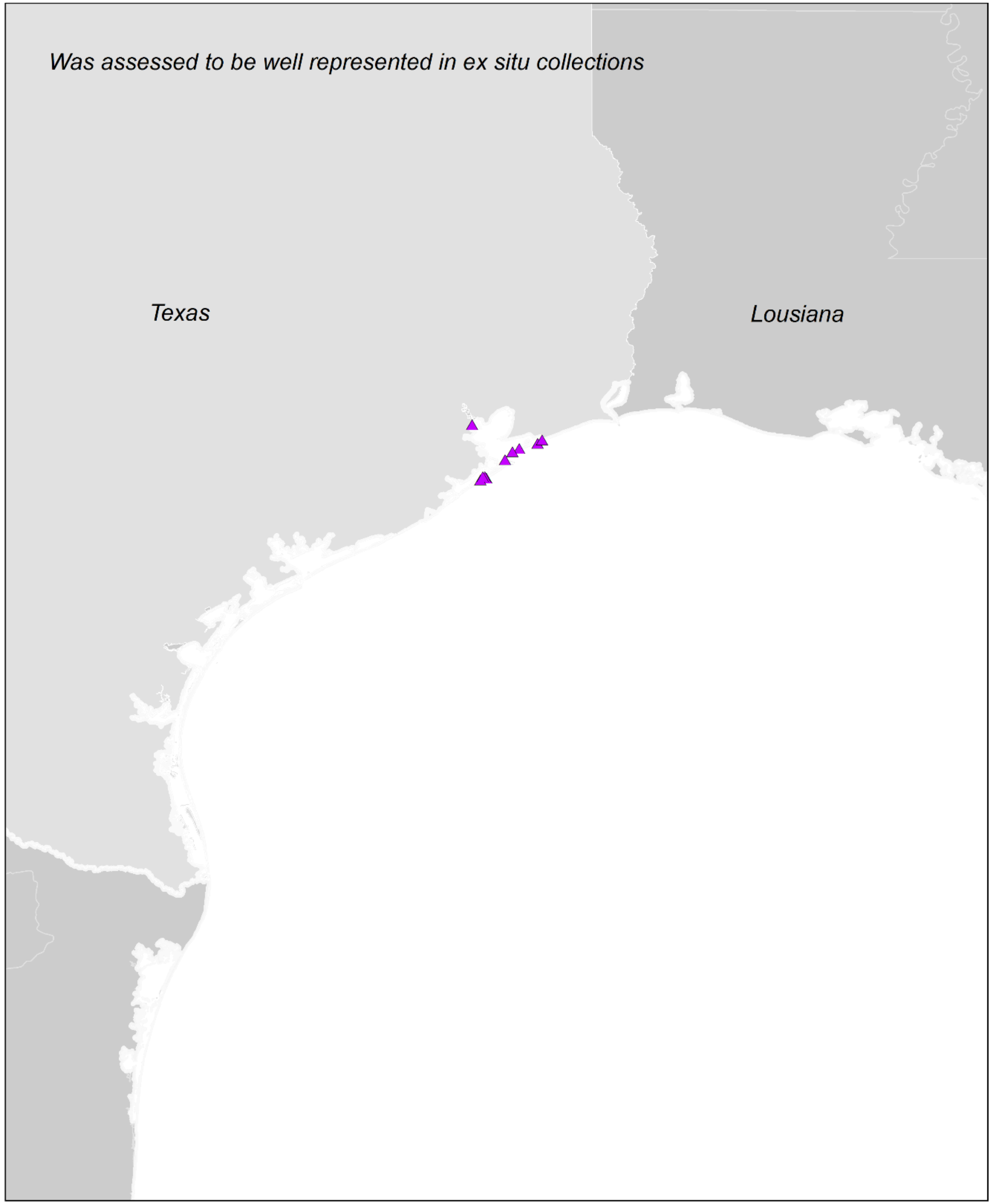
Was assessed to be well represented in ex situ collections
Texas
Lousiana

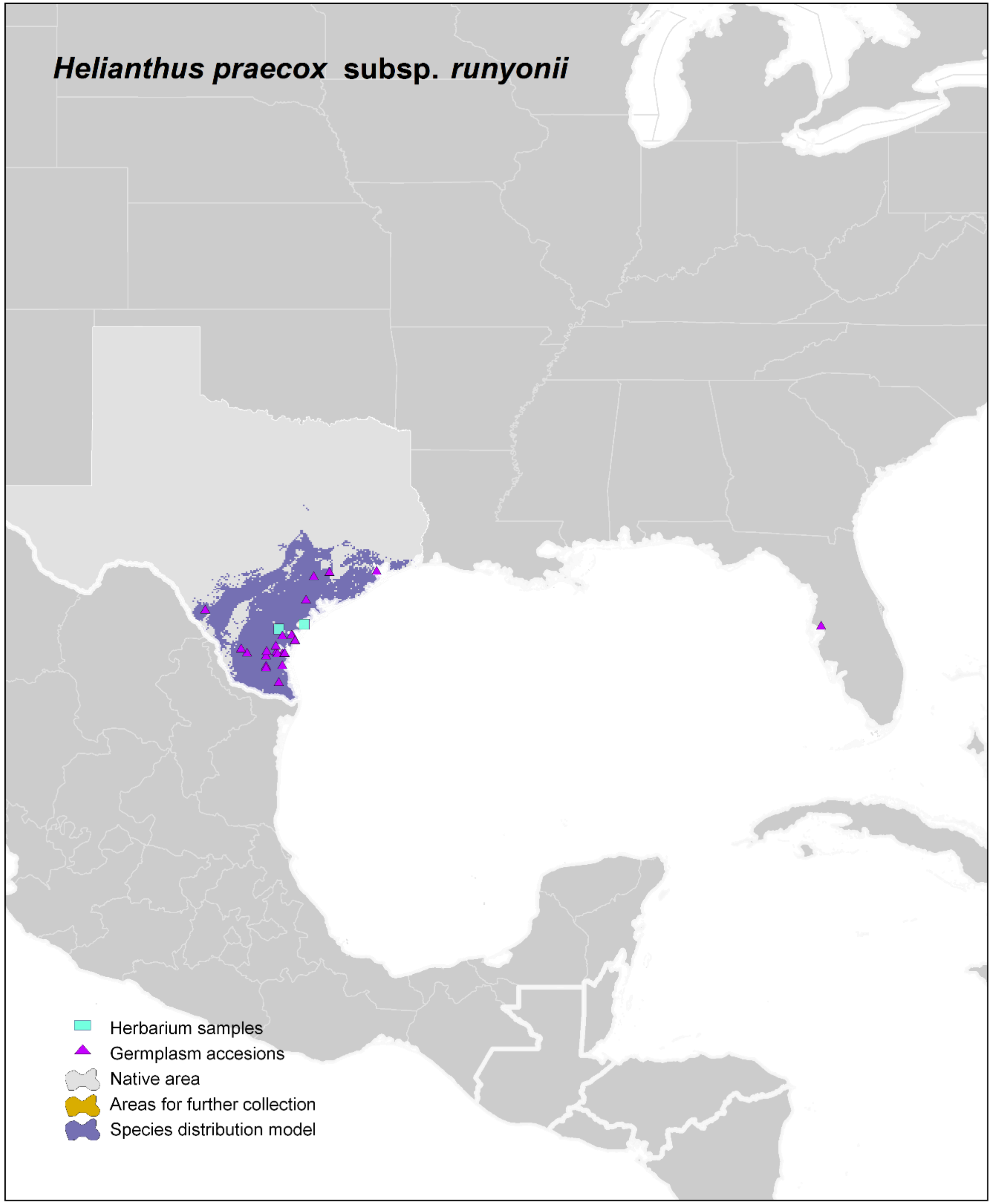

Helianthus praecox subsp. *runyonii*
Herbarium samples
Germplasm accesions
Native area
Areas for further collection
Species distribution model

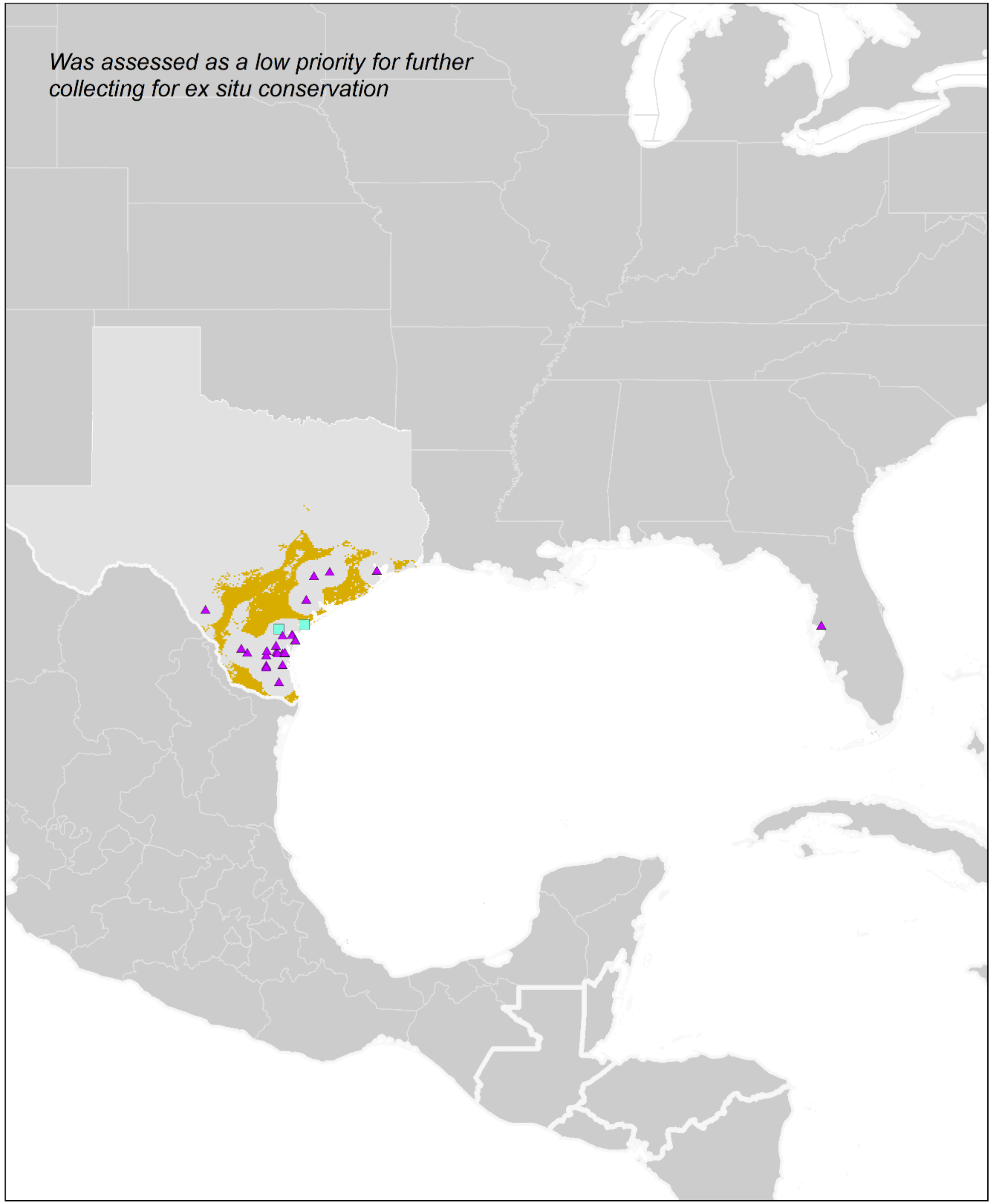

Was assessed as a low priority for further collecting for ex situ conservation

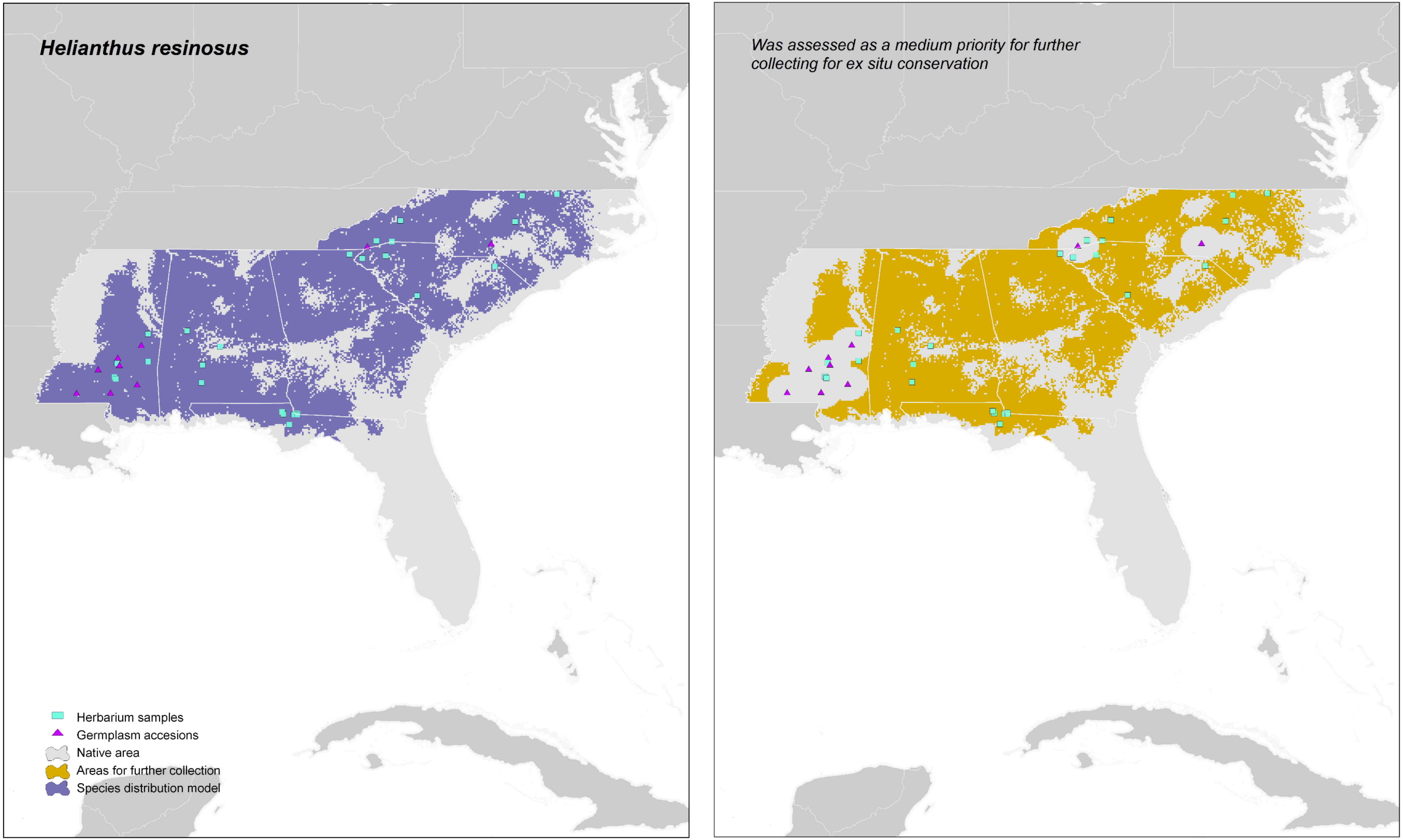

Helianthus resinosus
Herbarium samples
Germplasm accesions
Native area
Areas for further collection
Species distribution model
Was assessed as a medium priority for further collecting for ex situ conservation

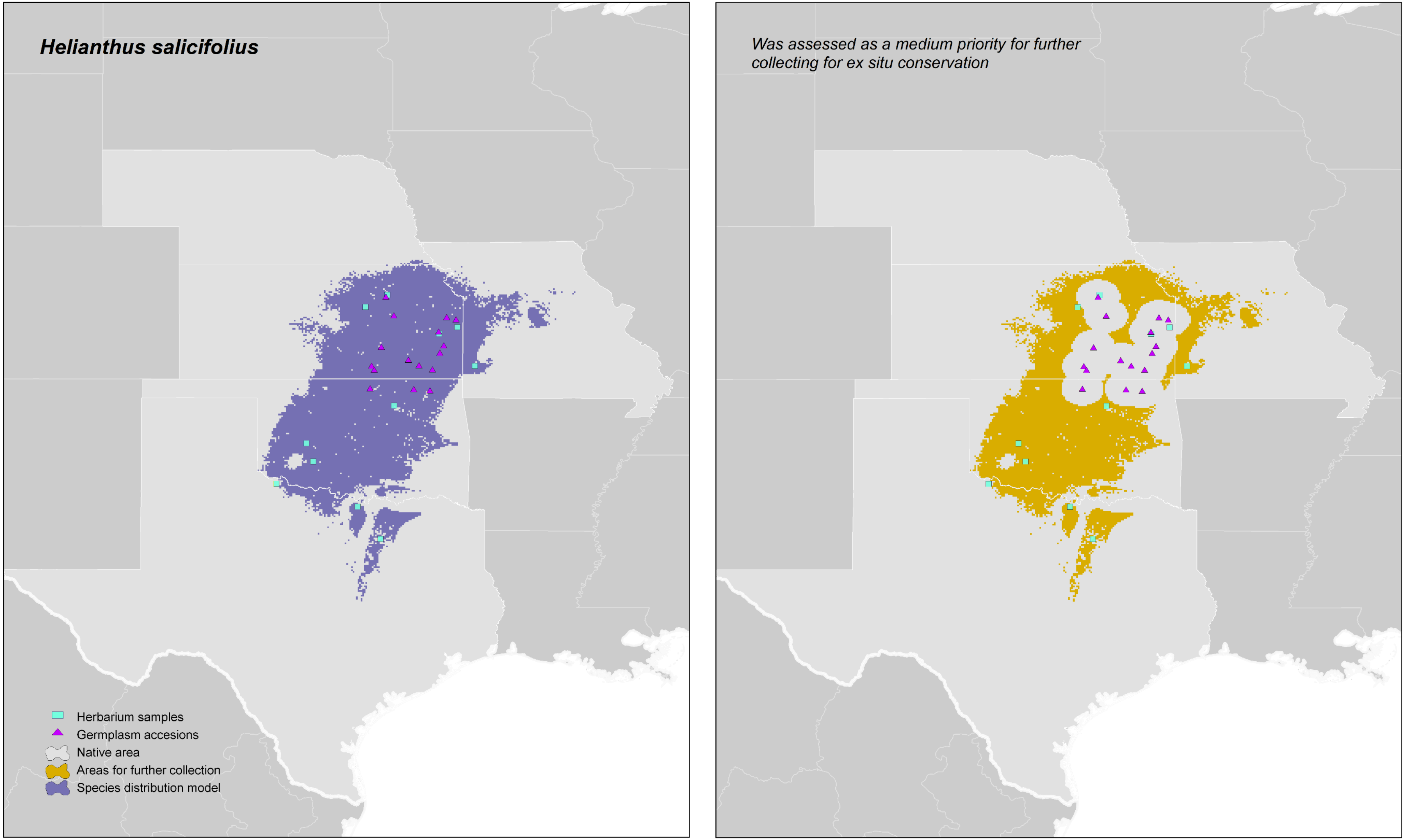
Helianthus salicifolius
Herbarium samples
Germplasm accesions
Native area
Areas for further collection
Species distribution model
Was assessed as a medium priority for further collecting for ex situ conservation

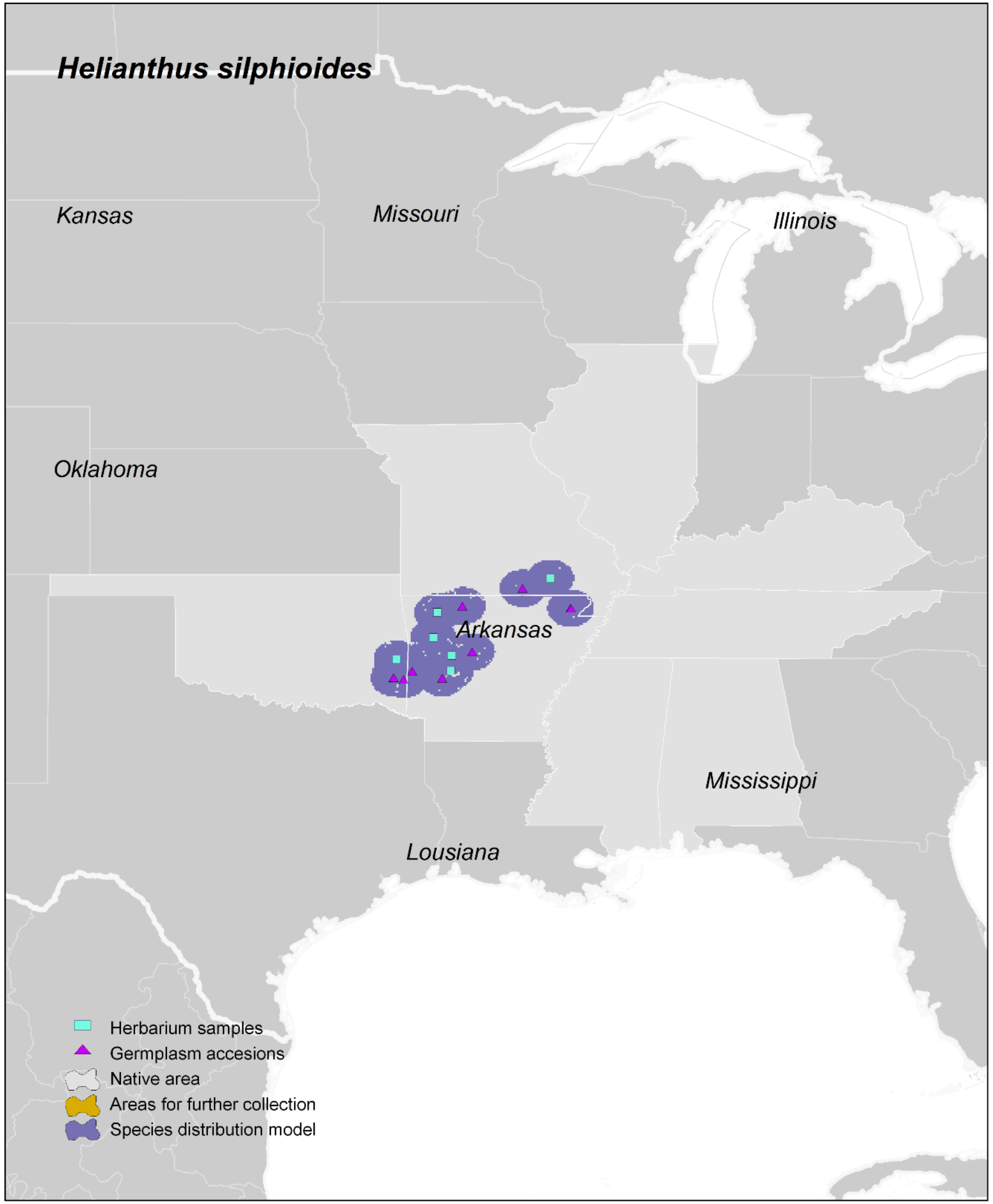
Helianthus silphioides
Kansas
Missouri
Illinois
Oklahoma
Arkansas
Mississippi
Lousiana
Herbarium samples
Germplasm accesions
Native area
Areas for further collection
Species distribution model

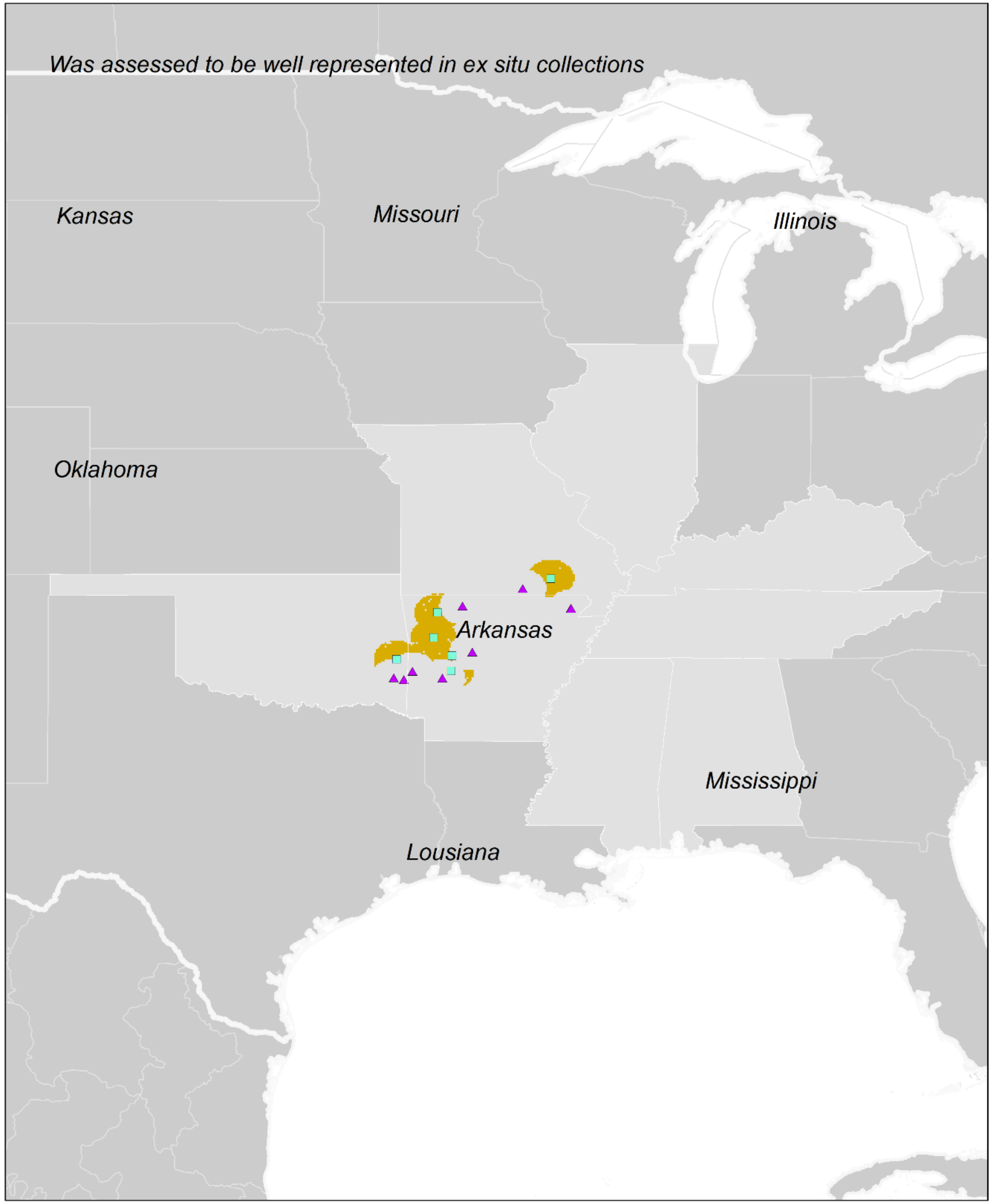
Was assessed to be well represented in ex situ collections
Kansas
Missouri
Illinois
Oklahoma
Arkansas
Mississippi
Lousiana

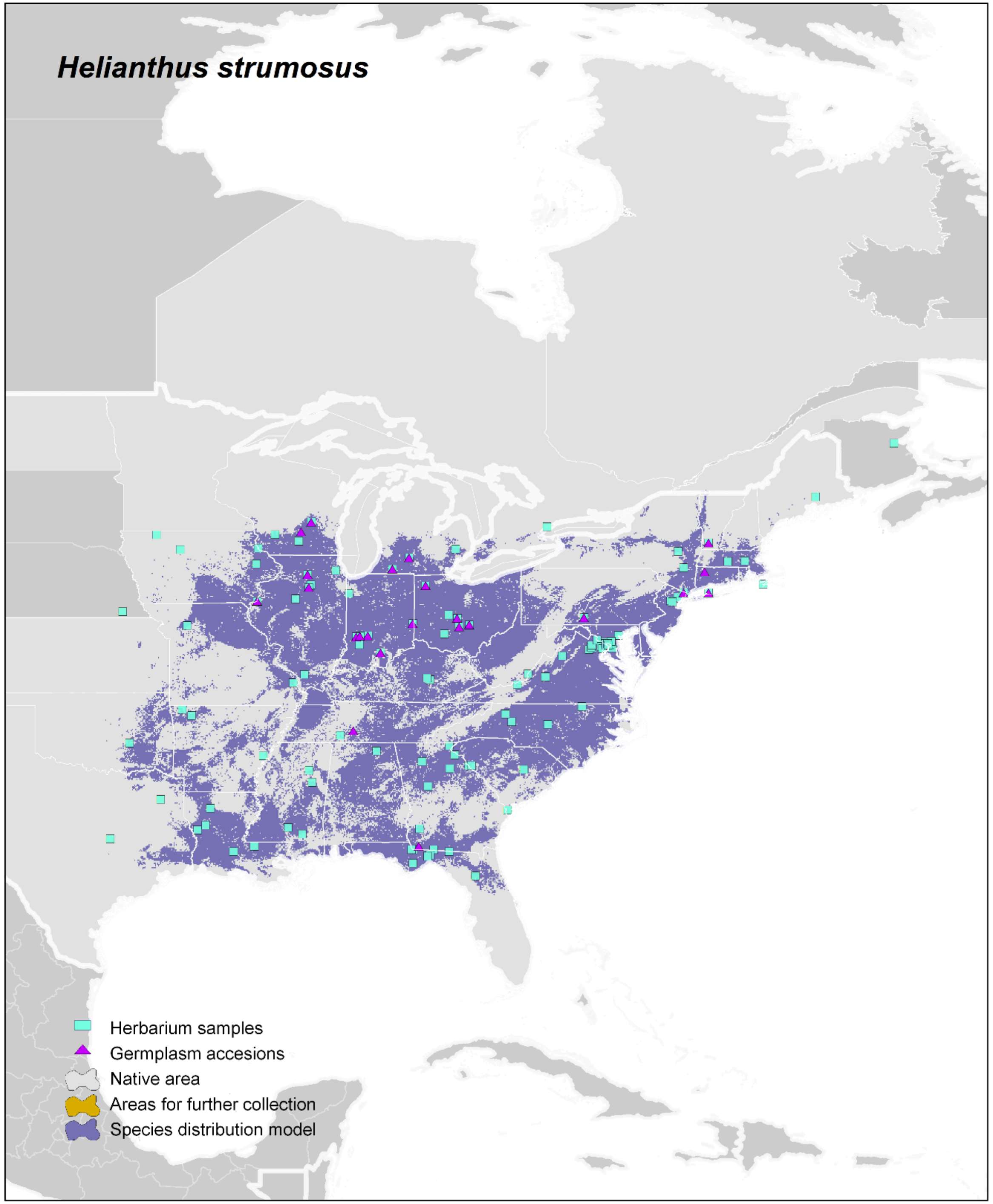
Helianthus strumosus
Herbarium samples
Germplasm accesions
Native area
Areas for further collection
Species distribution model

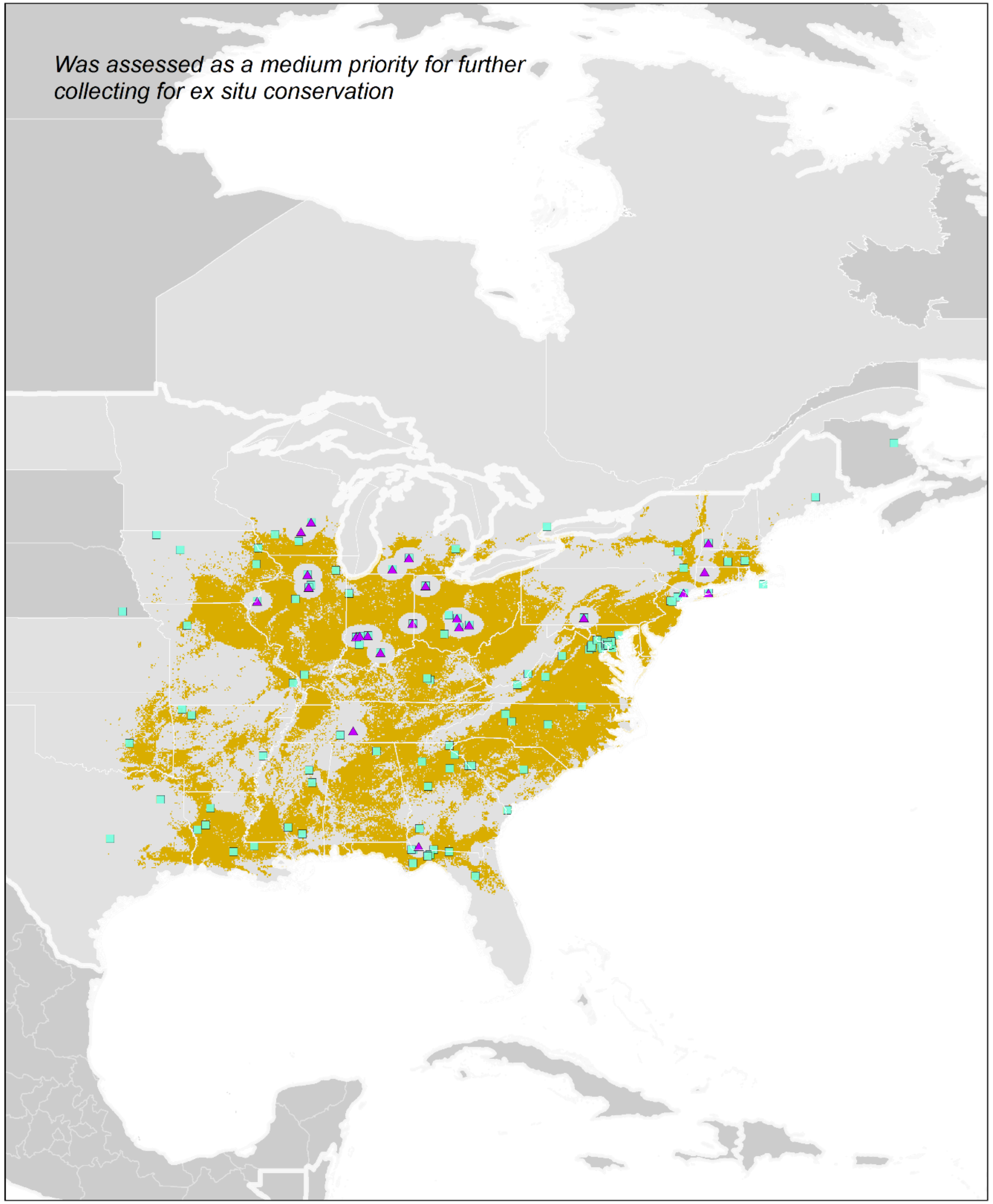
Was assessed as a medium priority for further collecting for ex situ conservation

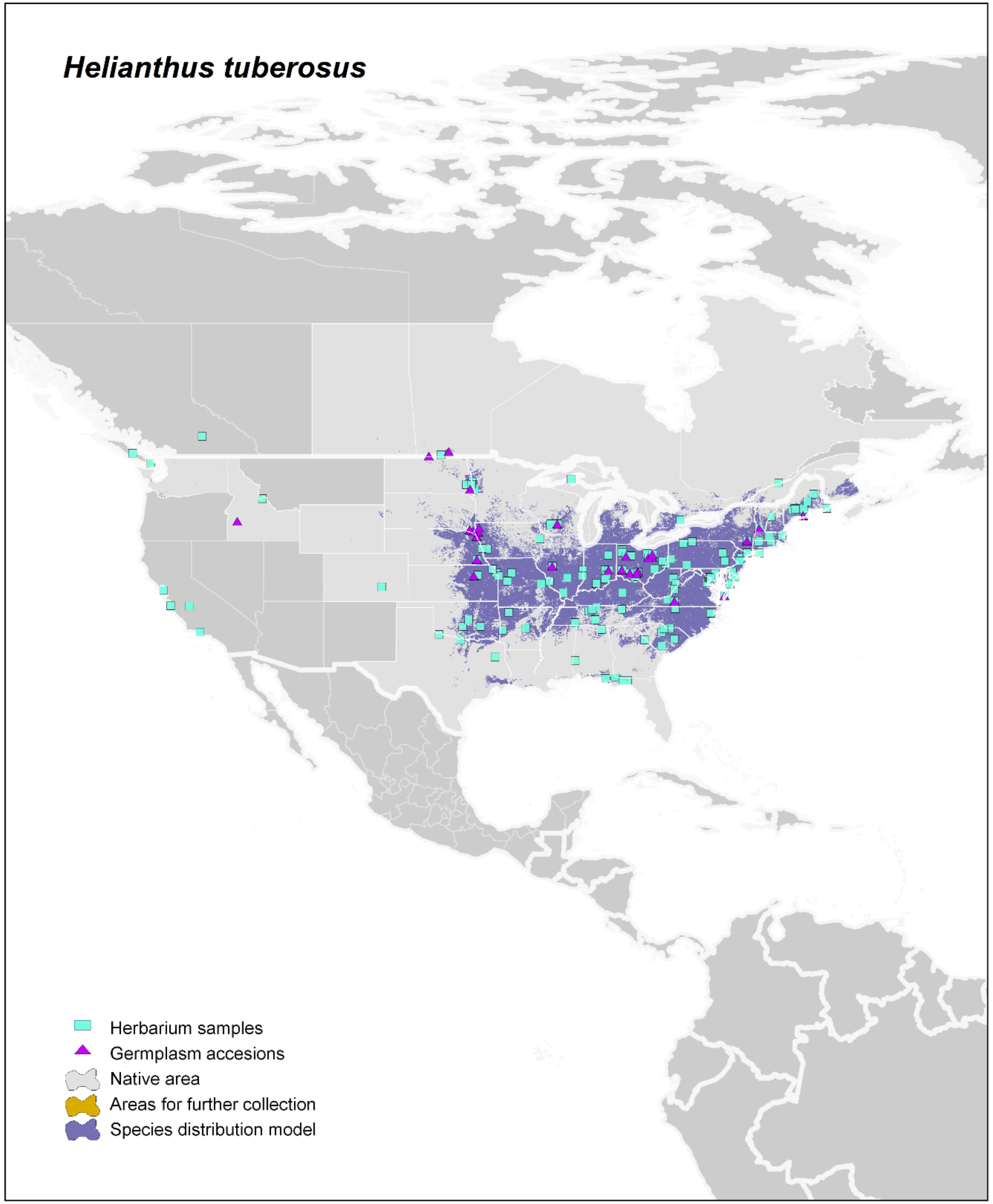
Helianthus tuberosus
Herbarium samples
Germplasm accesions
Native area
Areas for further collection
Species distribution model
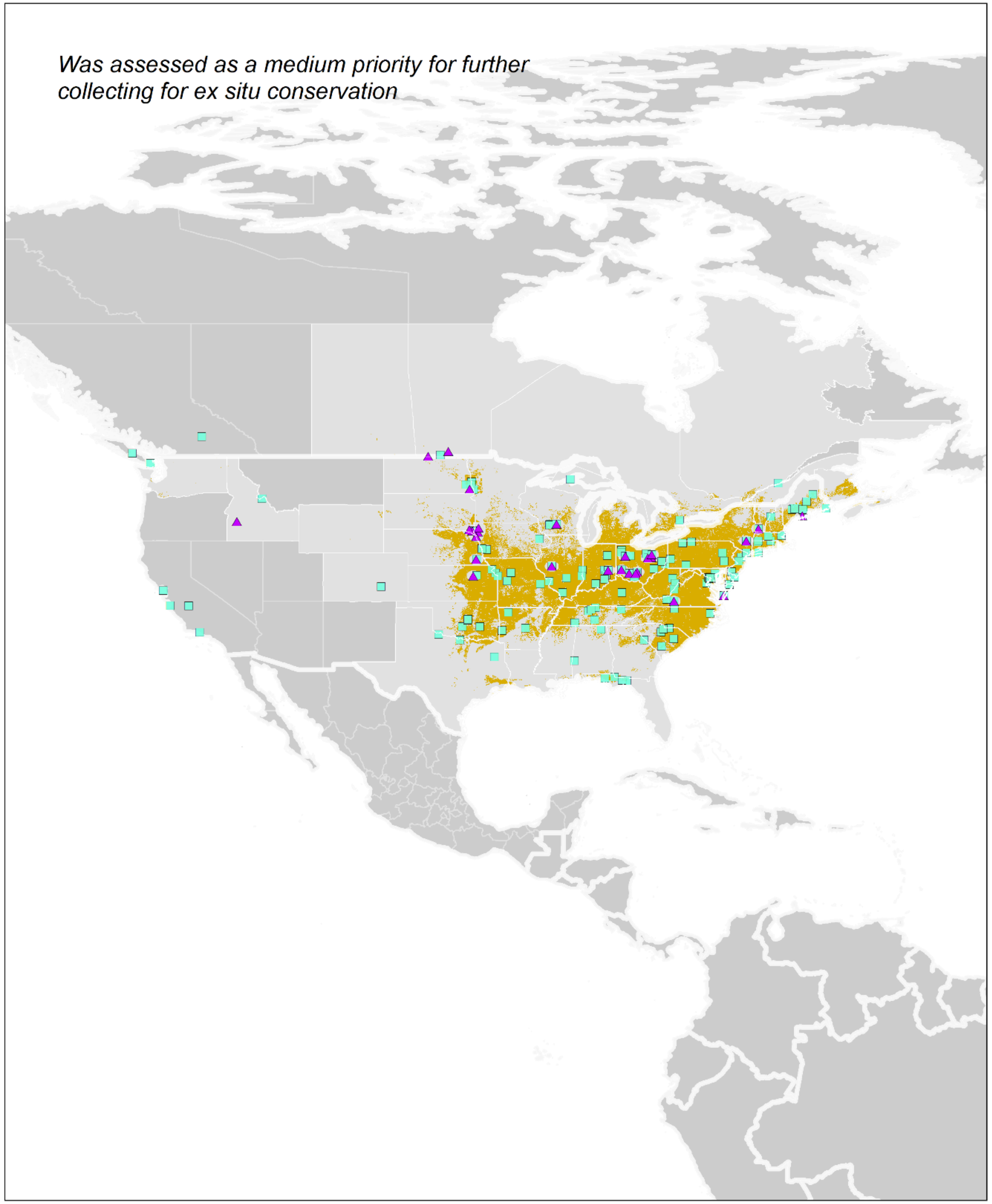
Was assessed as a medium priority for further collecting for ex situ conservation

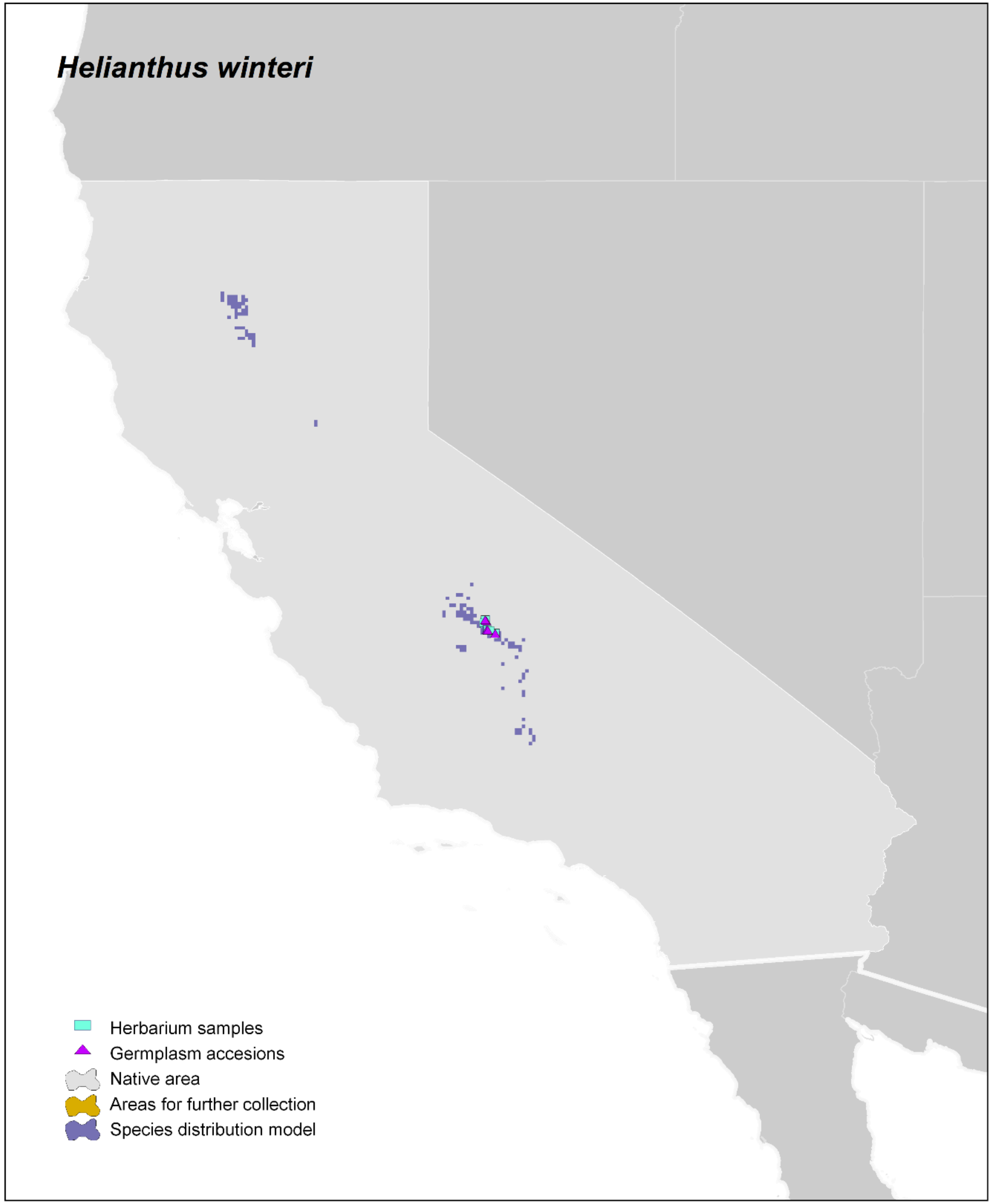
Helianthus winteri
Herbarium samples
Germplasm accesions
Native area
Areas for further collection
Species distribution model

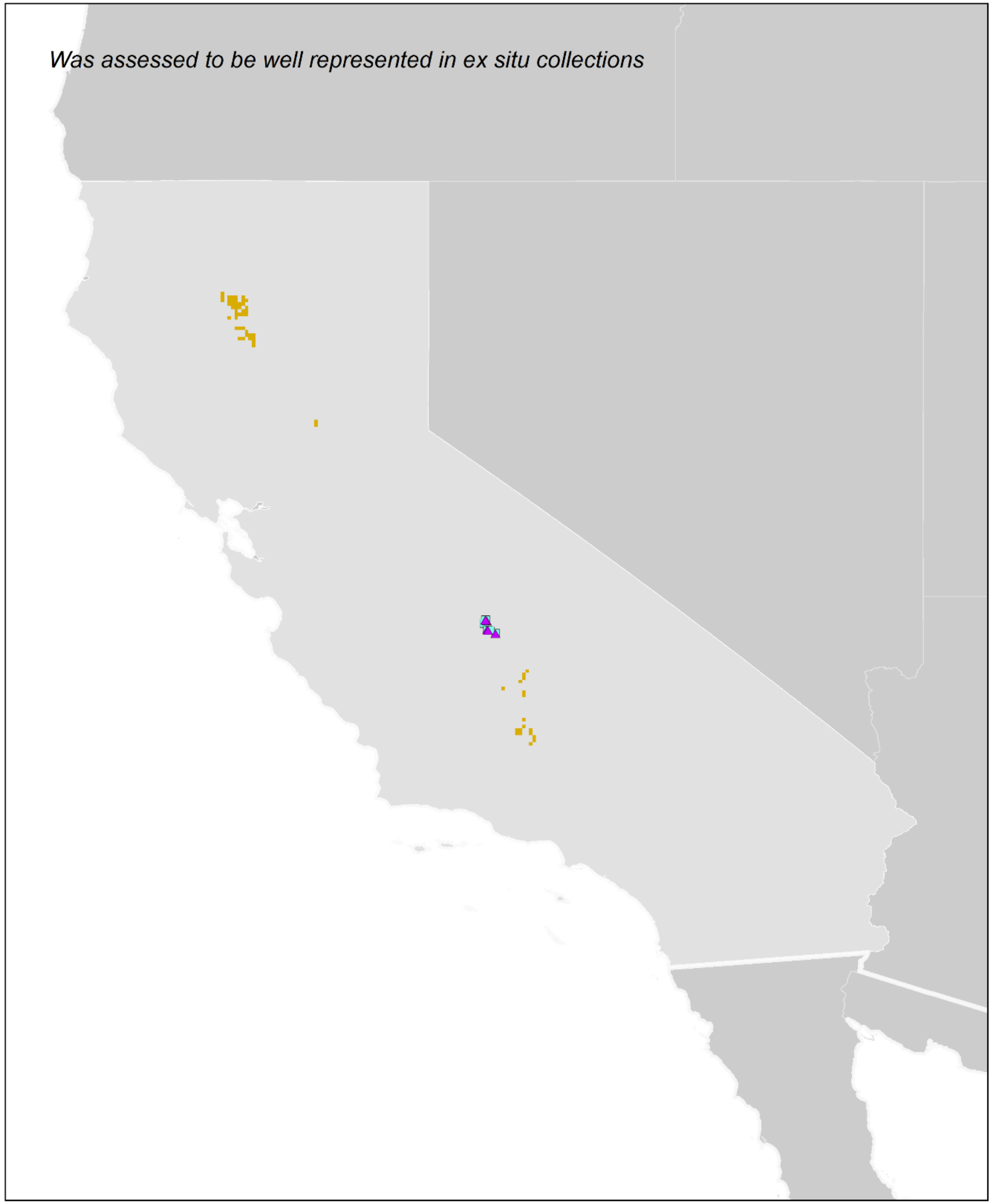
Was assessed to be well represented in ex situ collections
