# Supplementary material for: Ecogeography and utility to plant breeding of the crop wild relatives of sunflower (Helianthus annuus L.)
Source: Front Plant Sci. 2015 Oct 8;6:841. doi: 10.3389/fpls.2015.00841 (PMC4597133; doi:10.3389/fpls.2015.00841)

Figure S3. Heat map of geographic overlap as determined with respect to the smaller (minor range) in the bottom left, and larger range (major) in the top right. Red indicates no geographic overlap, white indicates a small amount of overlap and blue indicates a larger amount of overlap.

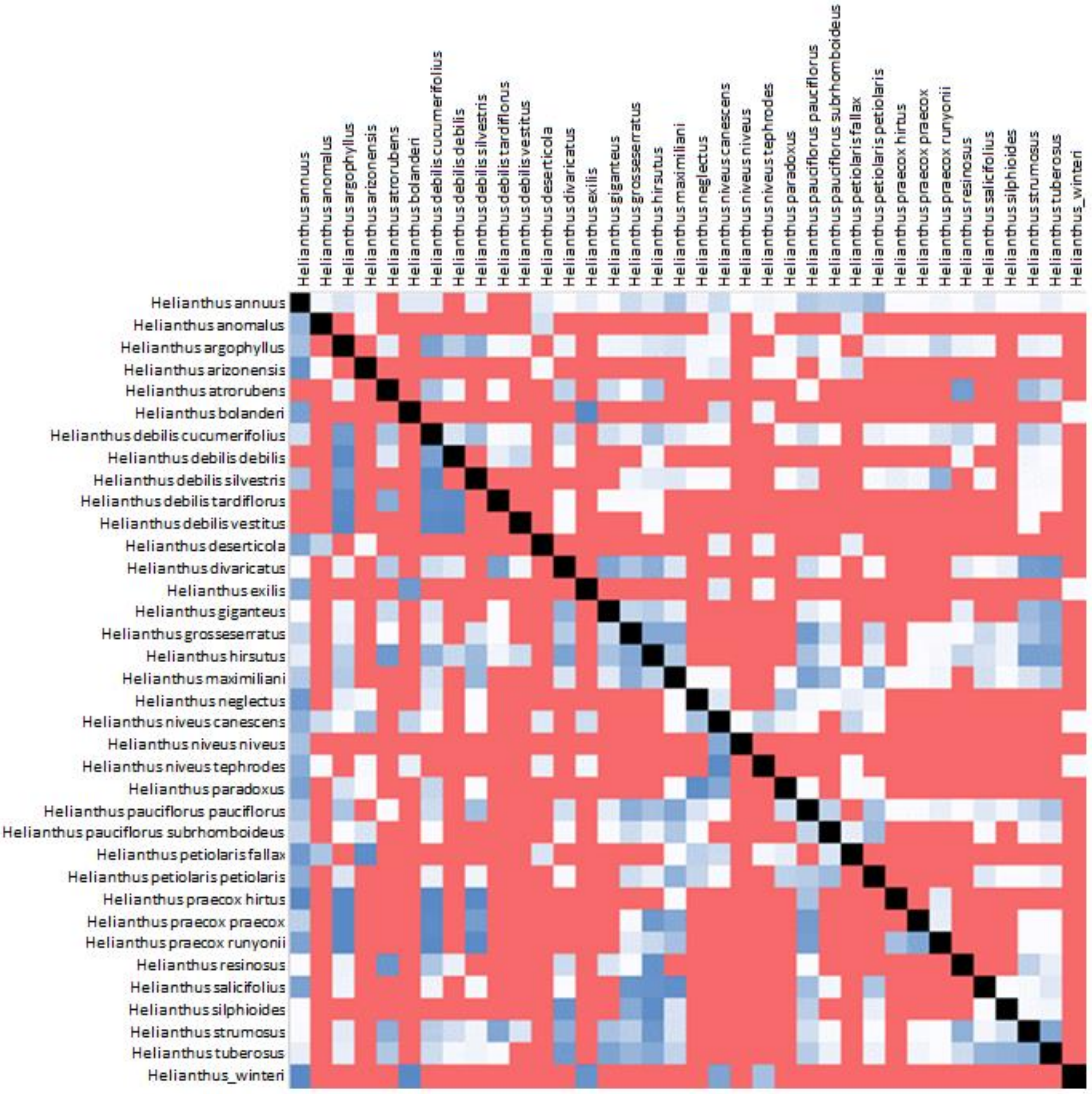

Supplement: Supplementary file 10 [file Image3.PDF]
