# Supplementary material for: Ecogeography and utility to plant breeding of the crop wild relatives of sunflower (Helianthus annuus L.)
Source: Front Plant Sci. 2015 Oct 8;6:841. doi: 10.3389/fpls.2015.00841 (PMC4597133; doi:10.3389/fpls.2015.00841)
Supplement: Supplementary file 11 [file Image4.PDF]

Figure S4. Predicted Niche Occupancy (PNO) for all 19 bioclimatic and 7 biophysical variables. Horizontal axes represent the bioclim parameter space divided into 50 equally spaced bins; vertical axes denote the total suitability of the mean annual temperature index of each species over its entire geographic range. Overlapping peaks of PNO profiles indicate similar tolerances, while the overall breadth of the profile denotes the degree of specificity in tolerance. Black profiles indicate the primary germplasm, red indicates the secondary germplasm pool, blue indicates the tertiary germplasm pool.

Predicted Niche Occupancy-Bioclim 1

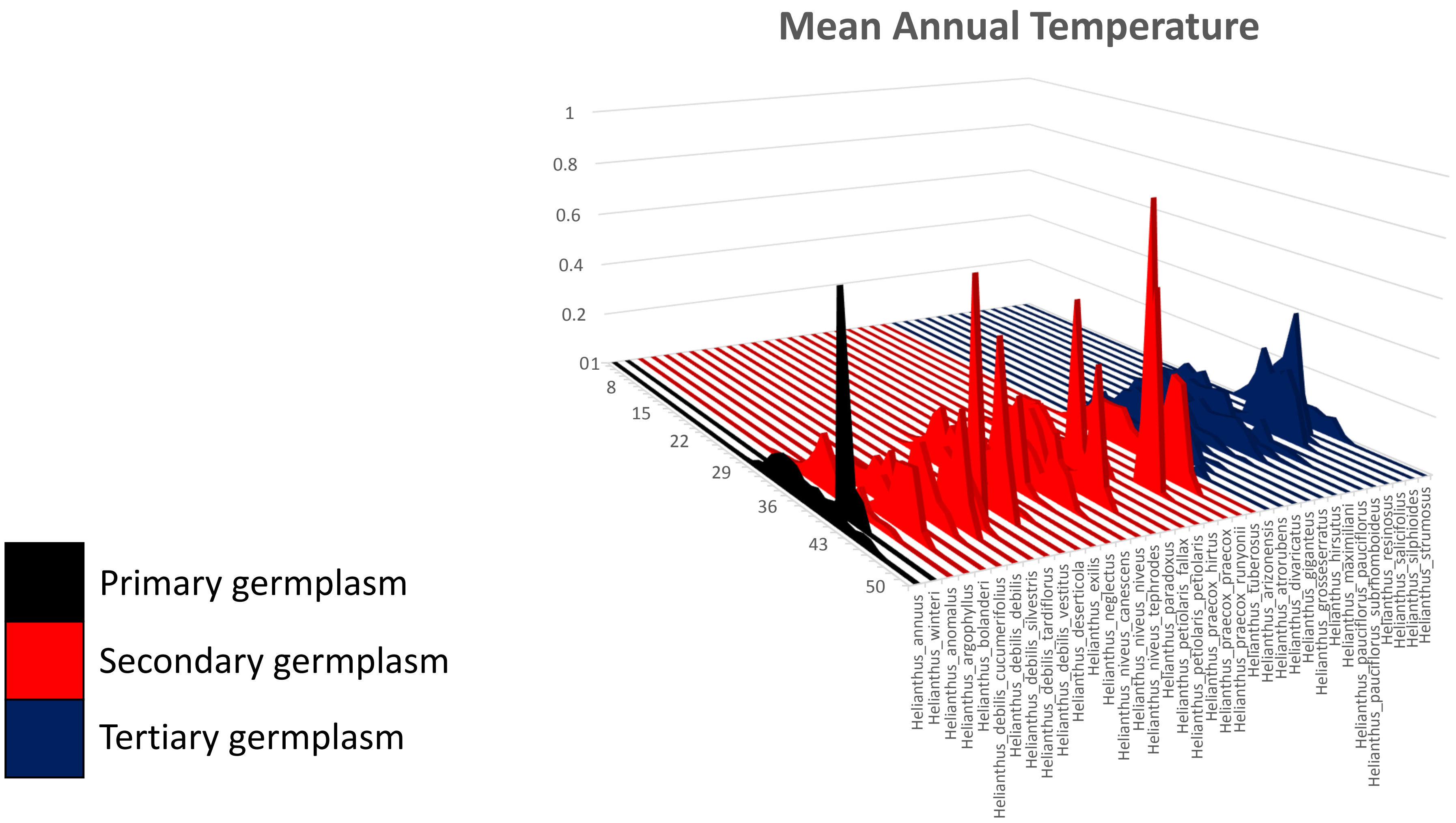

Predicted Niche Occupancy-Bioclim 2

Mean Diurnal Range

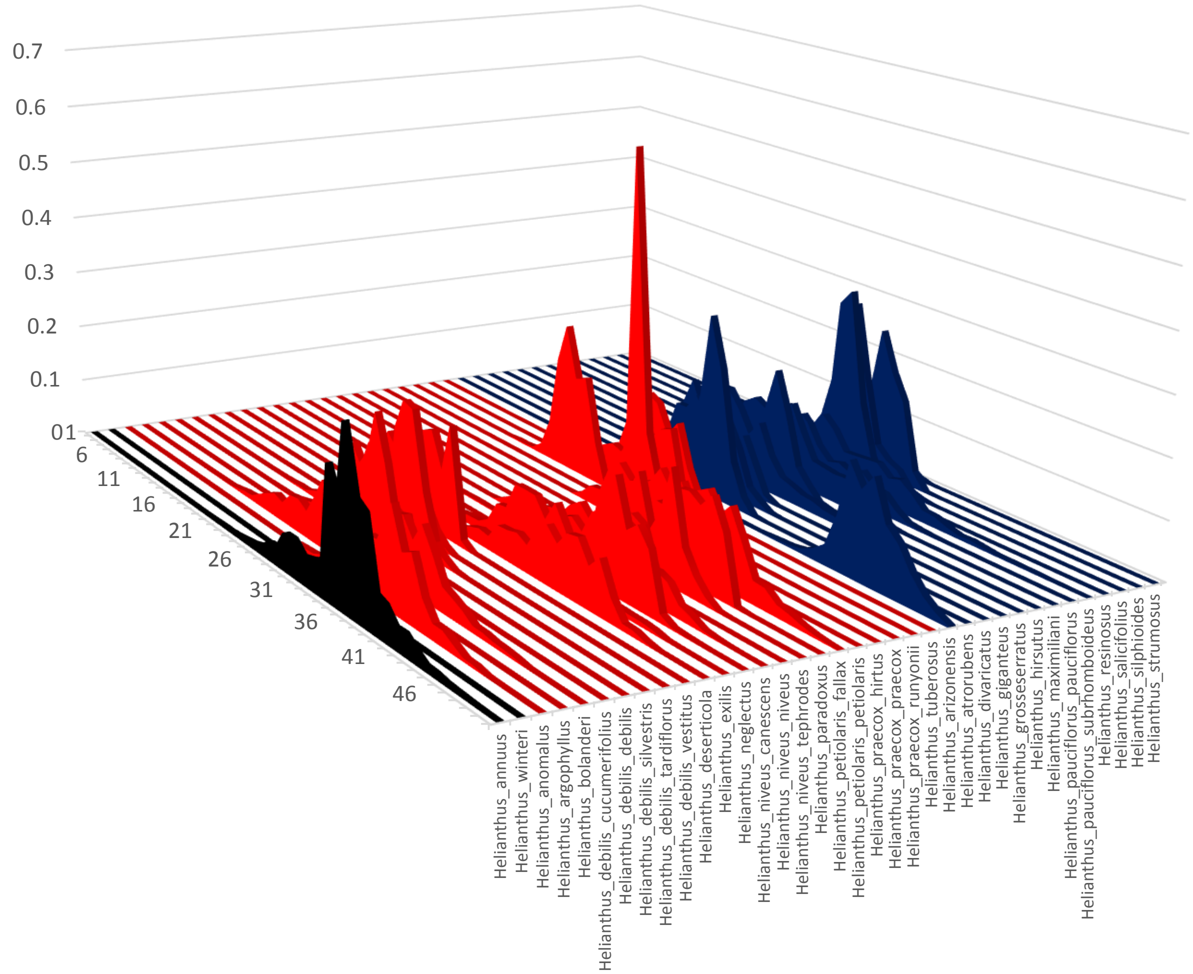

Primary germplasm

Secondary germplasm

Tertiary germplasm

Predicted Niche Occupancy-Bioclim 3

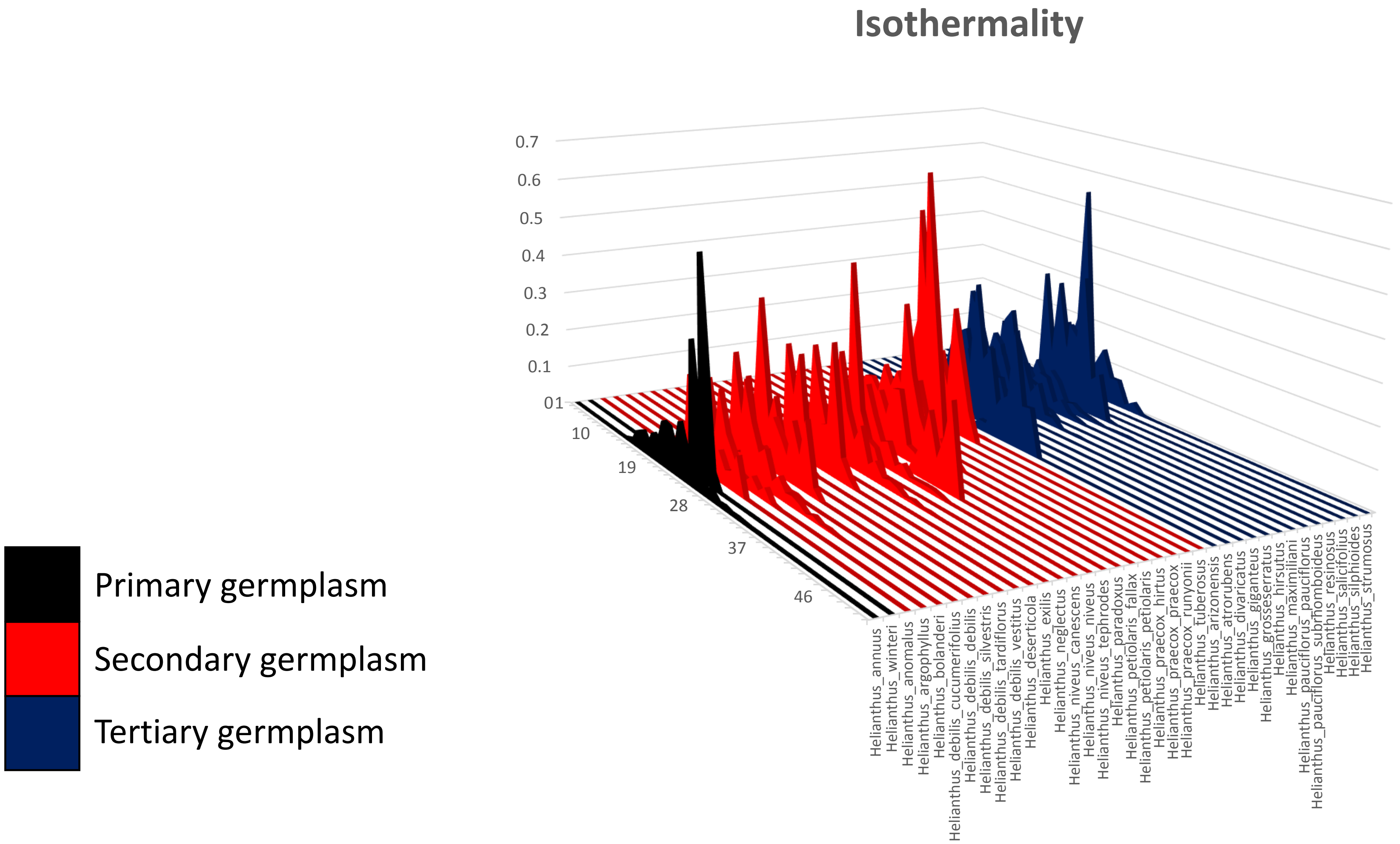

Predicted Niche Occupancy-Bioclim 4

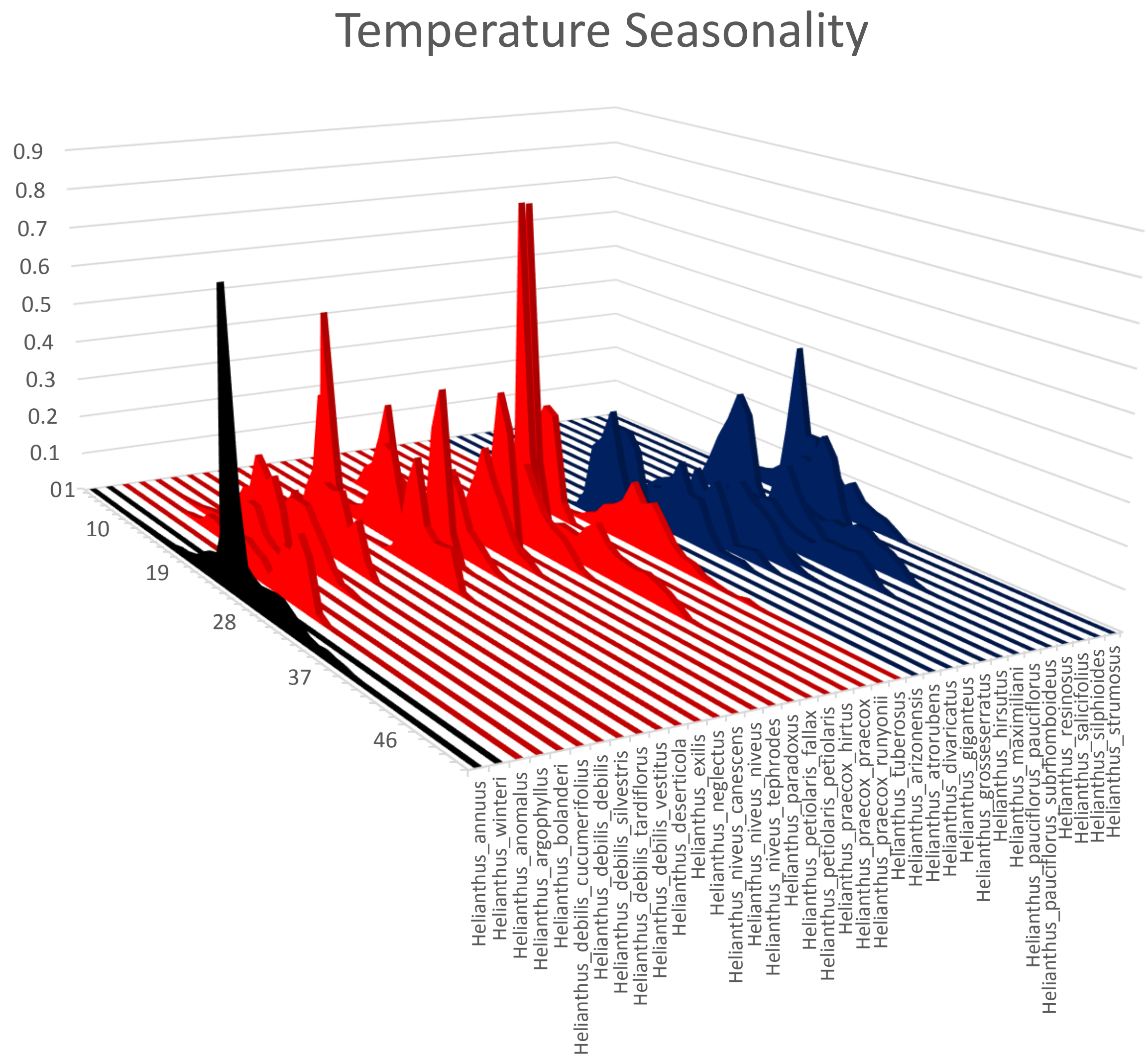

Primary germplasm

Secondary germplasm

Tertiary germplasm

Predicted Niche Occupancy-Bioclim 5

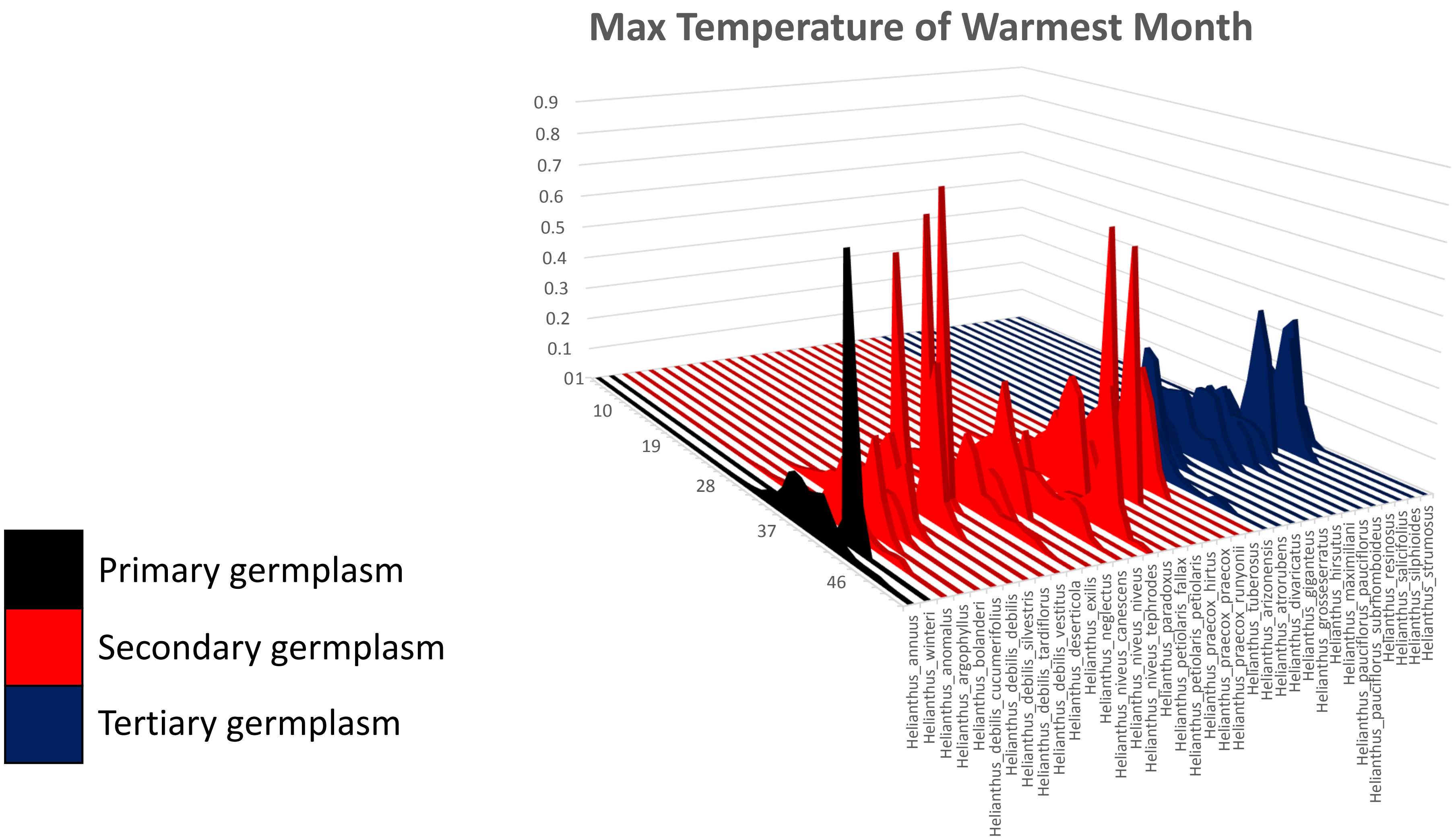

Predicted Niche Occupancy-Bioclim 6

Min Temperature of Coldest Month

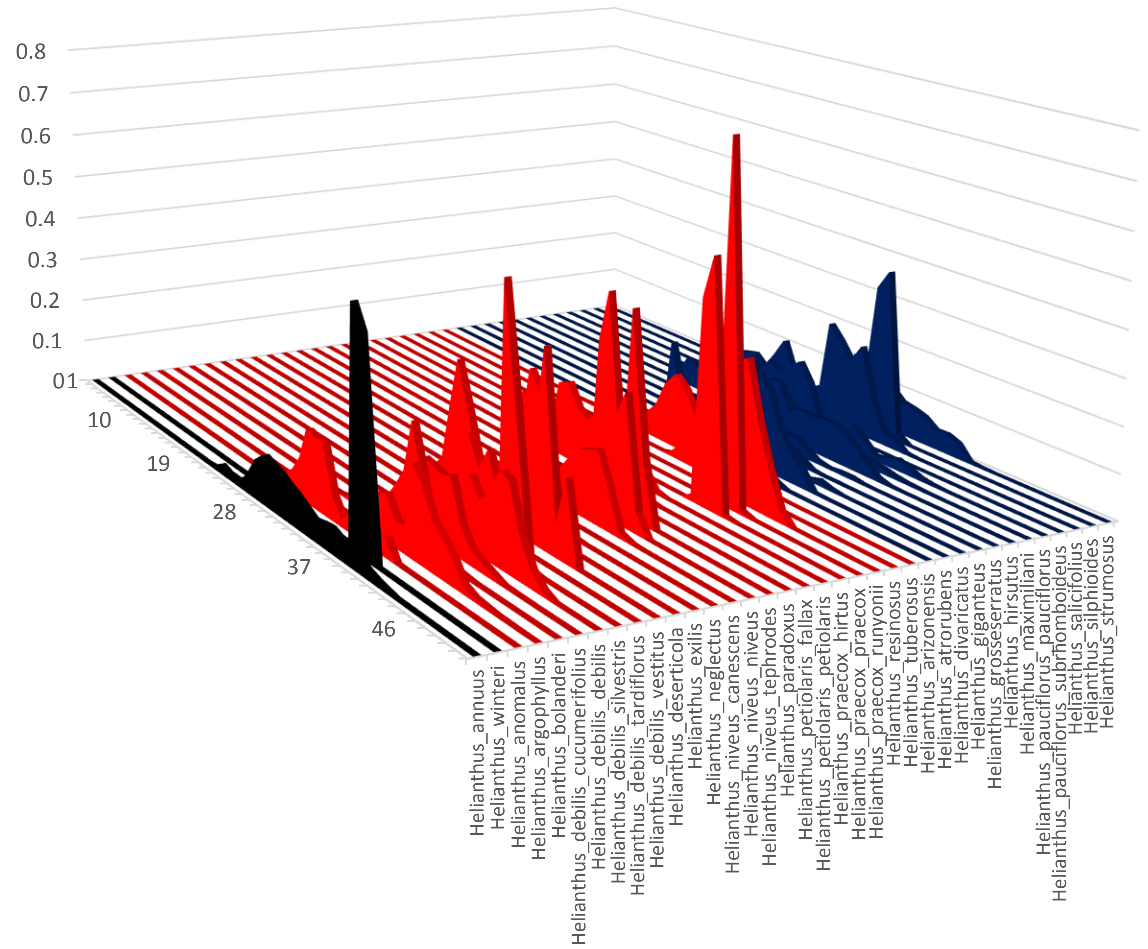

Primary germplasm

Secondary germplasm

Tertiary germplasm

Predicted Niche Occupancy-Bioclim 7

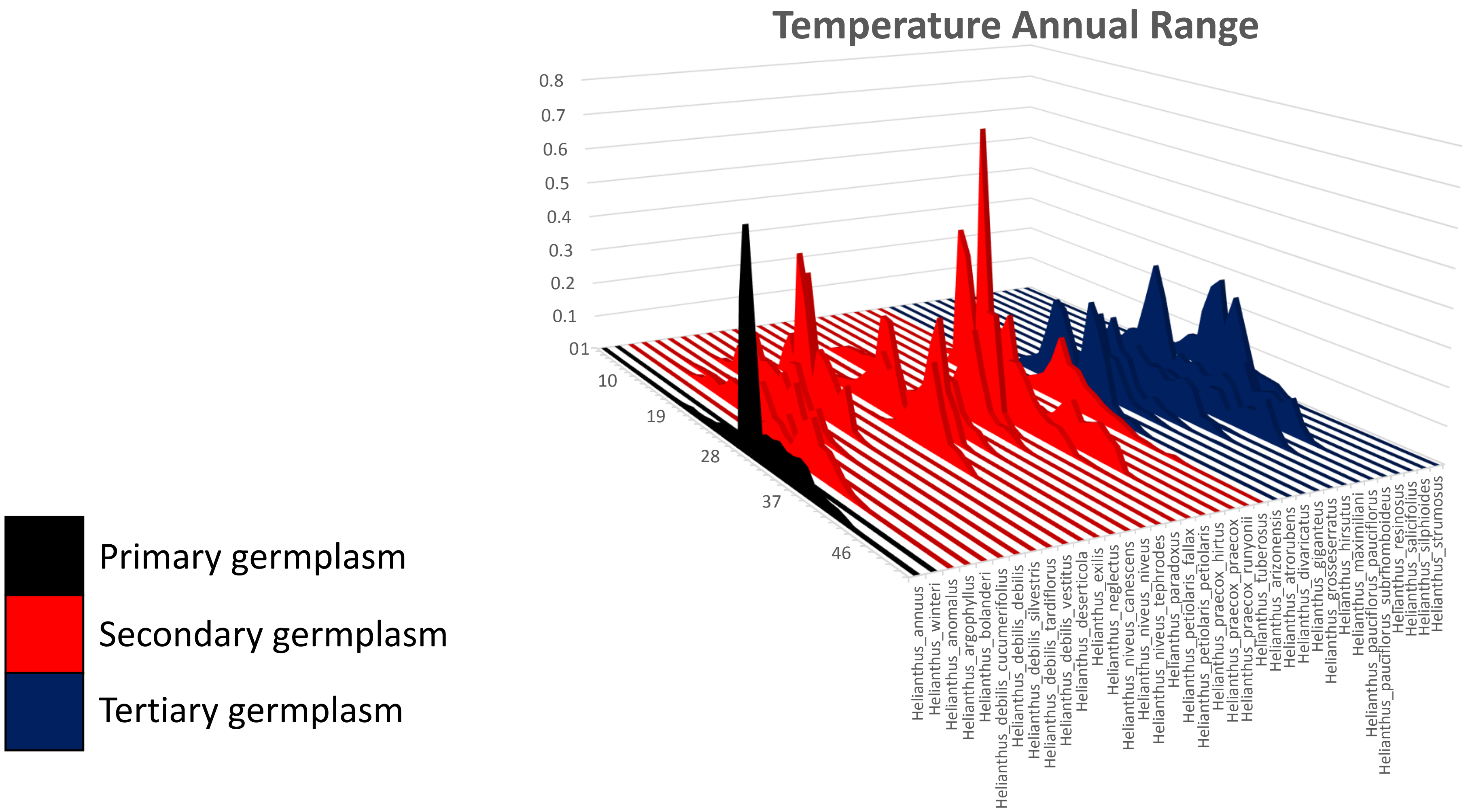

Predicted Niche Occupancy-Bioclim 8

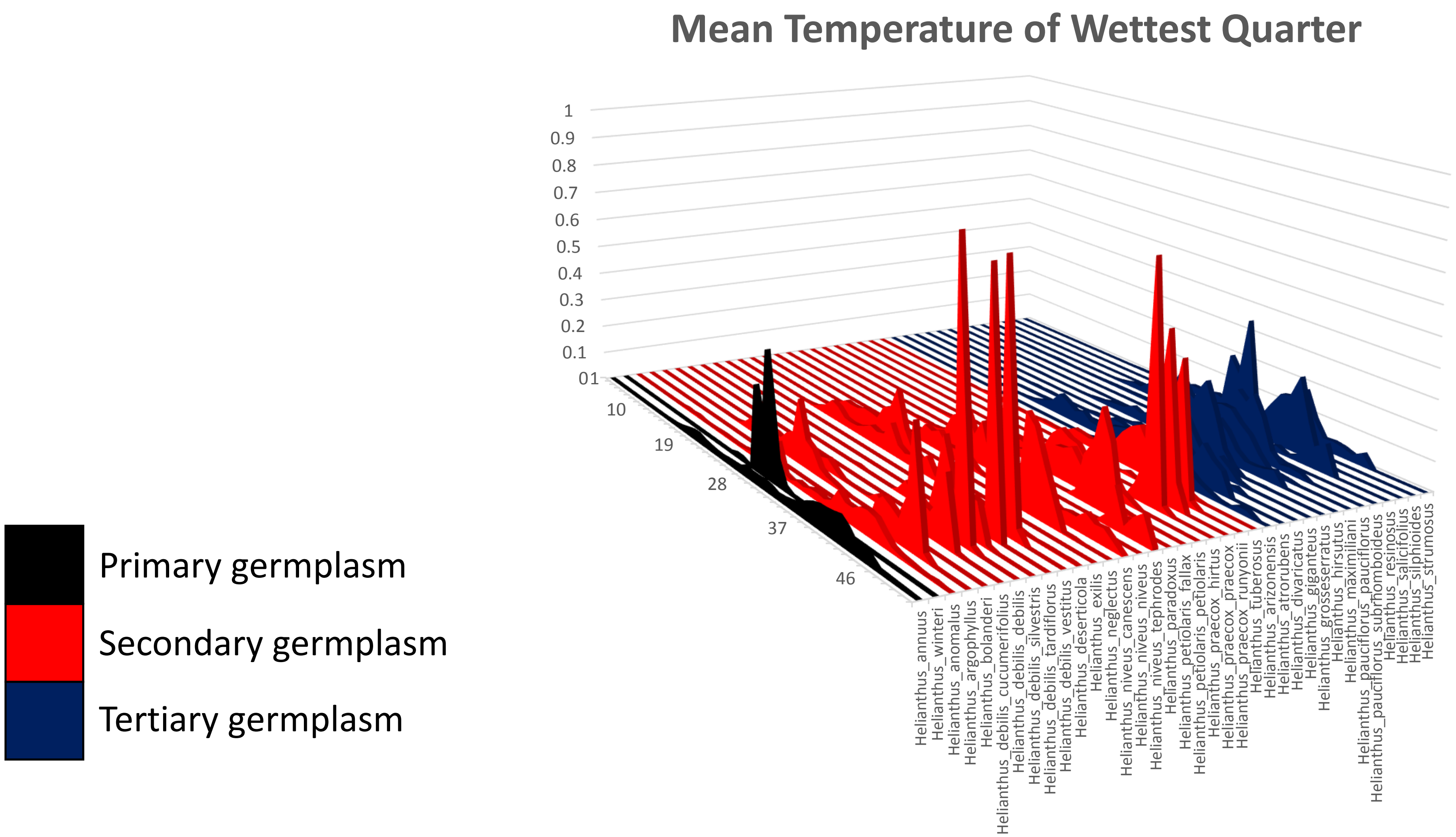

Predicted Niche Occupancy-Bioclim 9

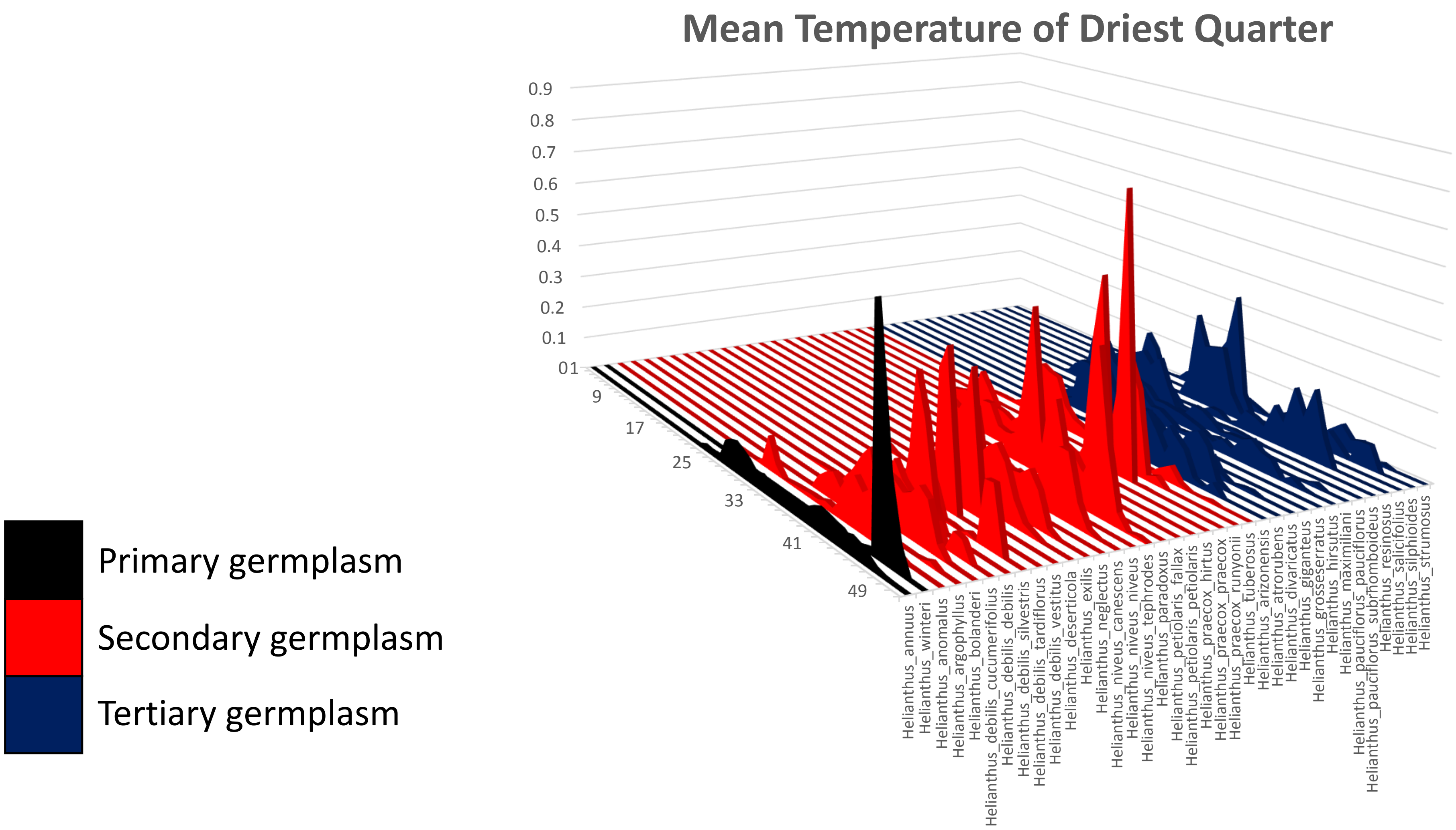

Predicted Niche Occupancy-Bioclim 10

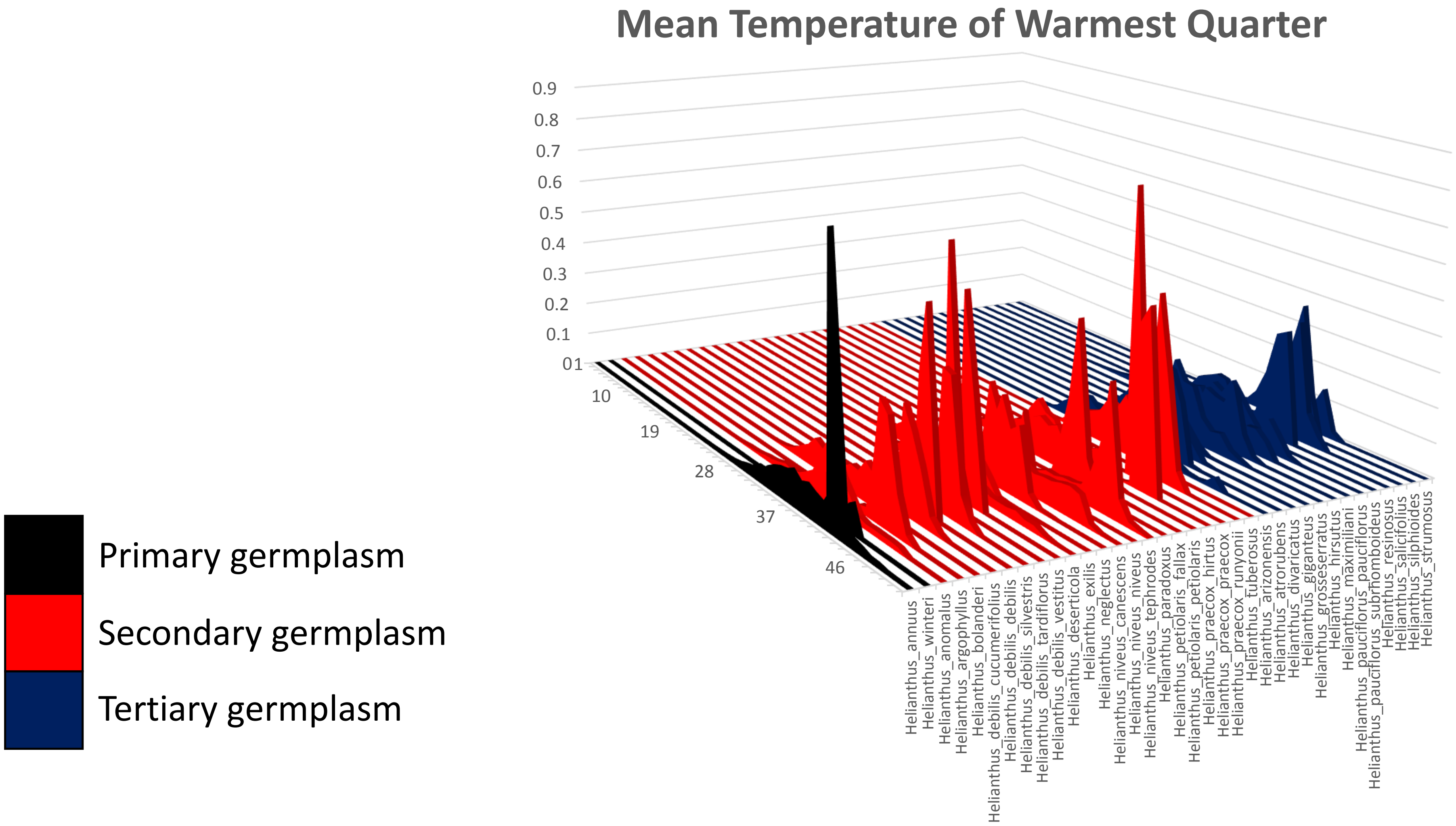

Predicted Niche Occupancy-Bioclim 11

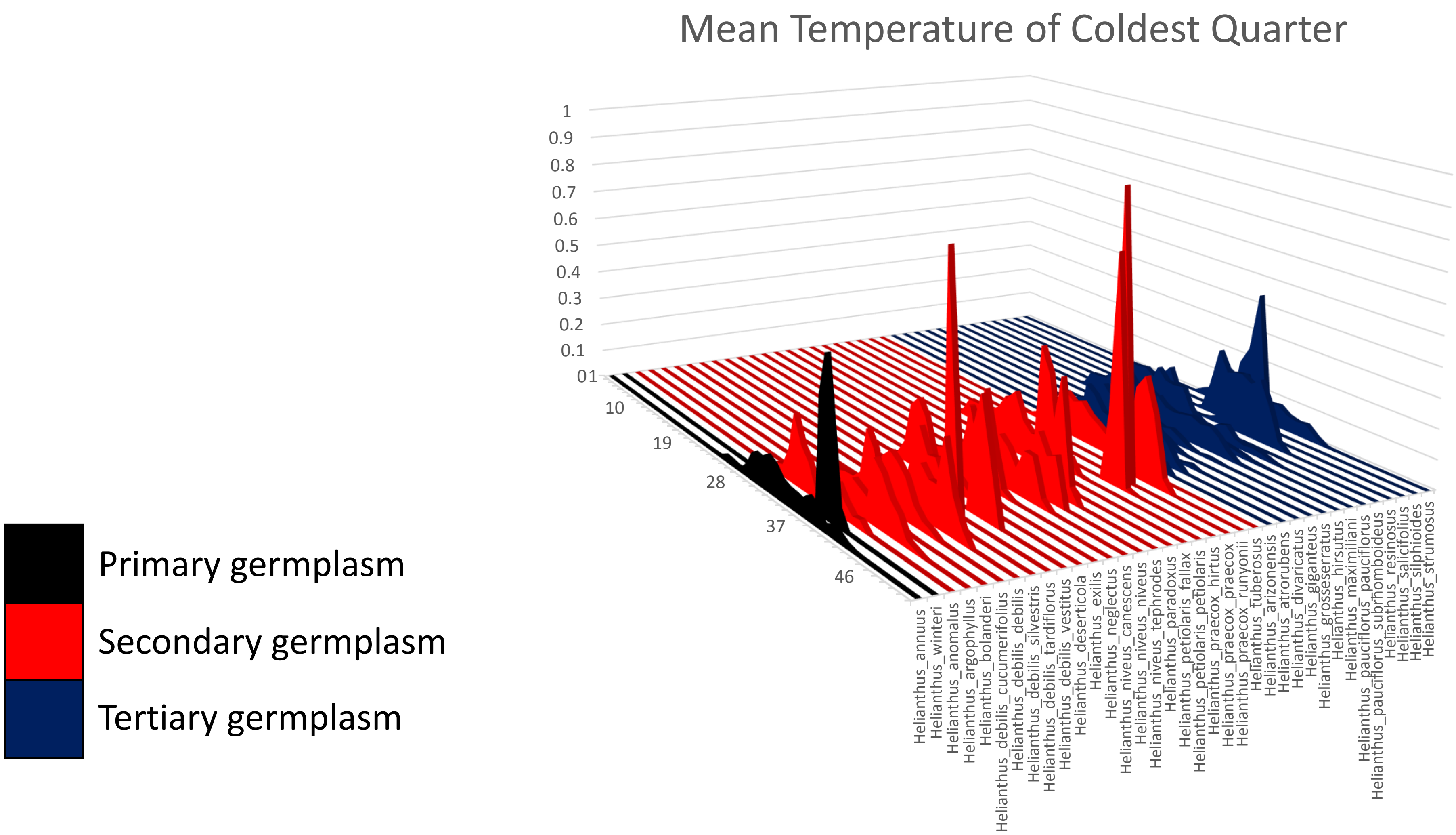

Predicted Niche Occupancy-Bioclim 12

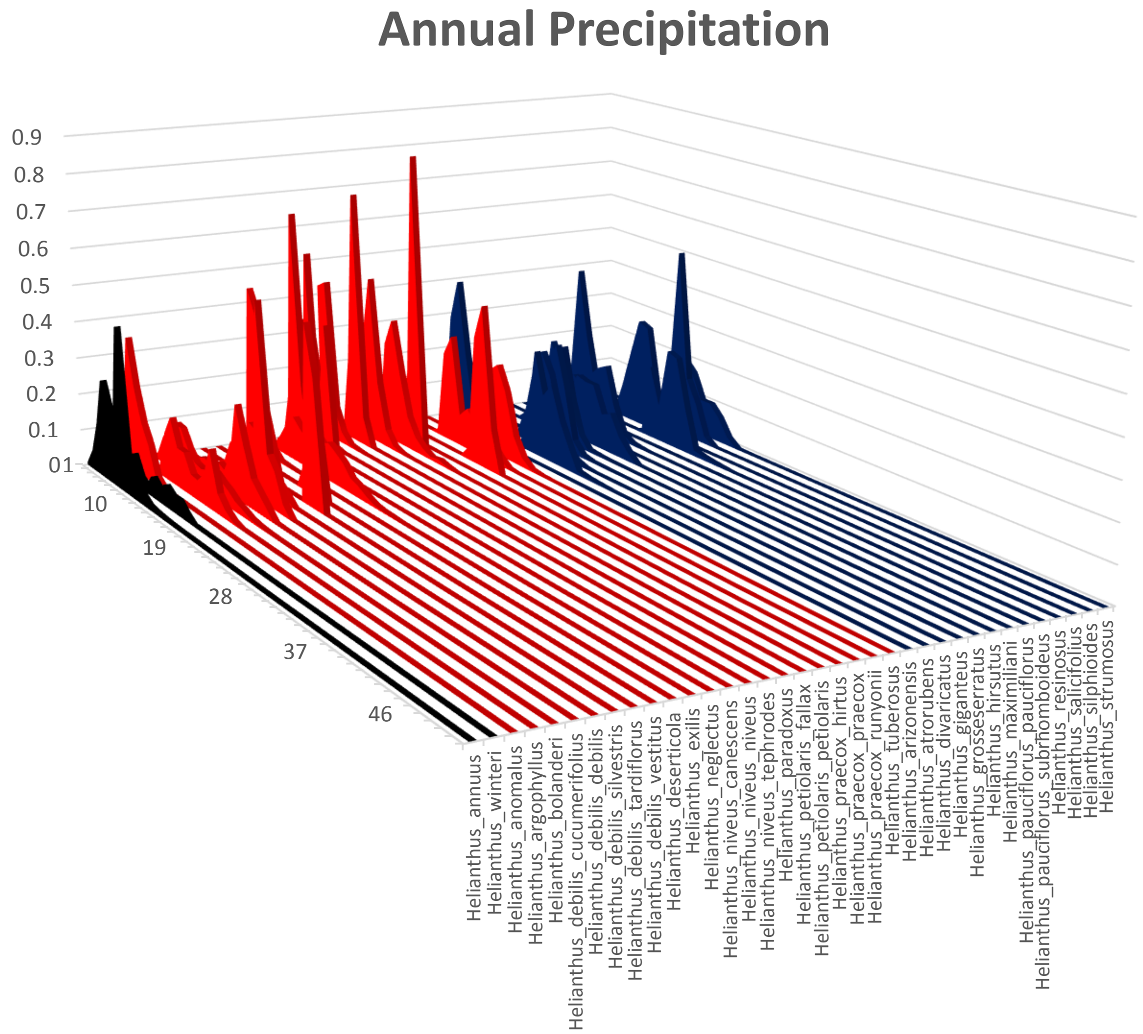

Primary germplasm

Secondary germplasm

Tertiary germplasm

Predicted Niche Occupancy-Bioclim 13

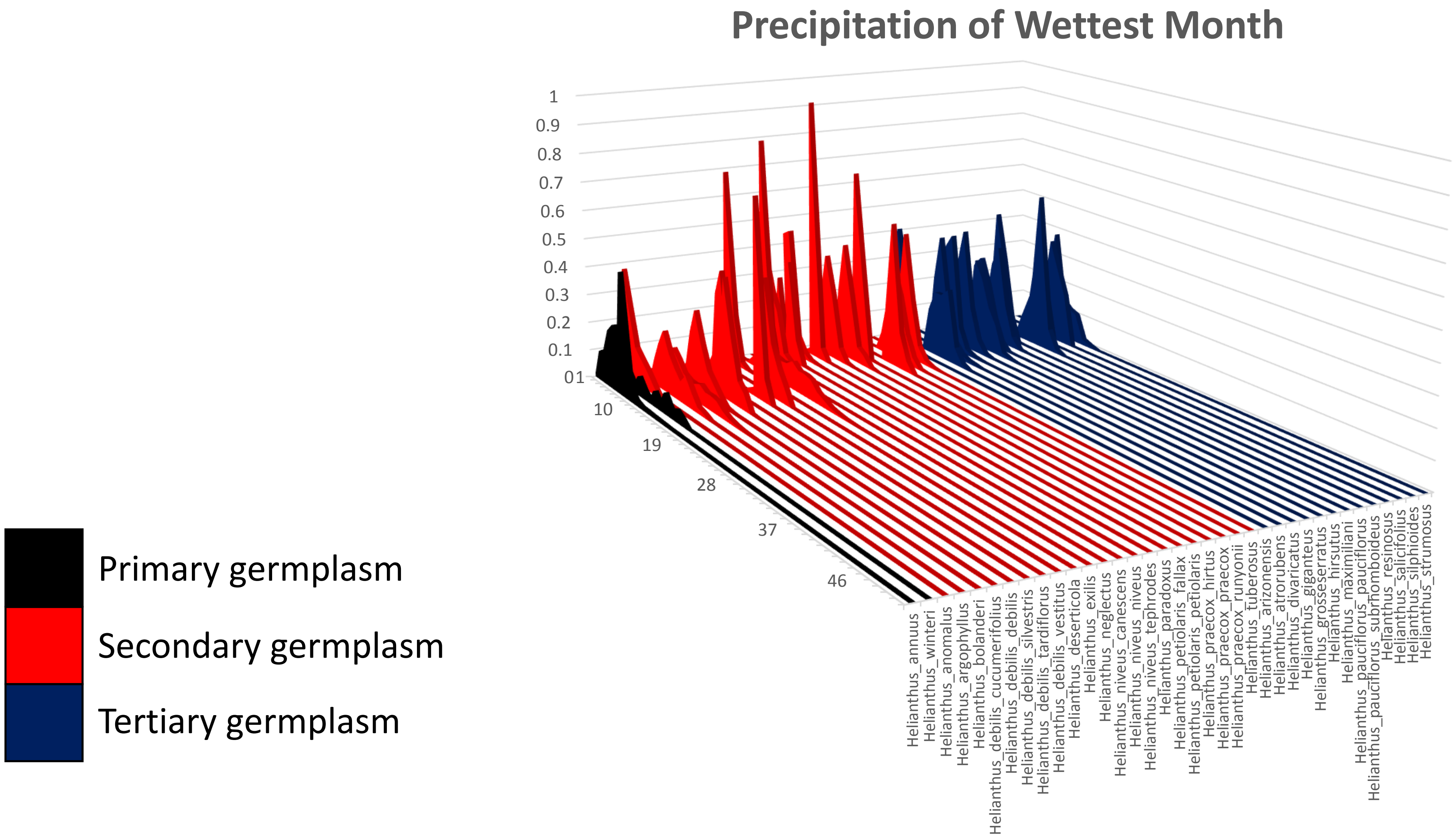

Predicted Niche Occupancy-Bioclim 14

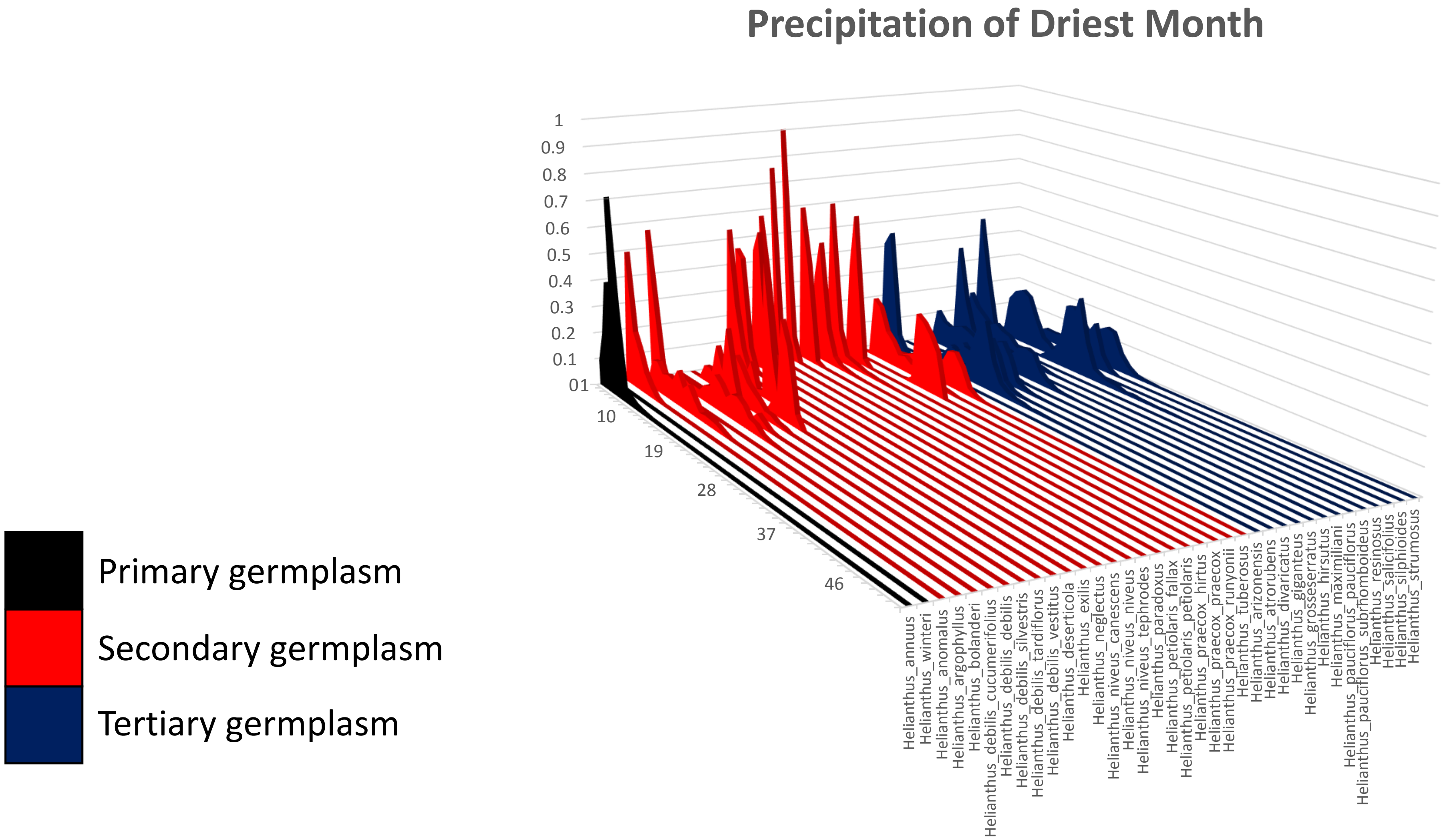

Predicted Niche Occupancy-Bioclim 15

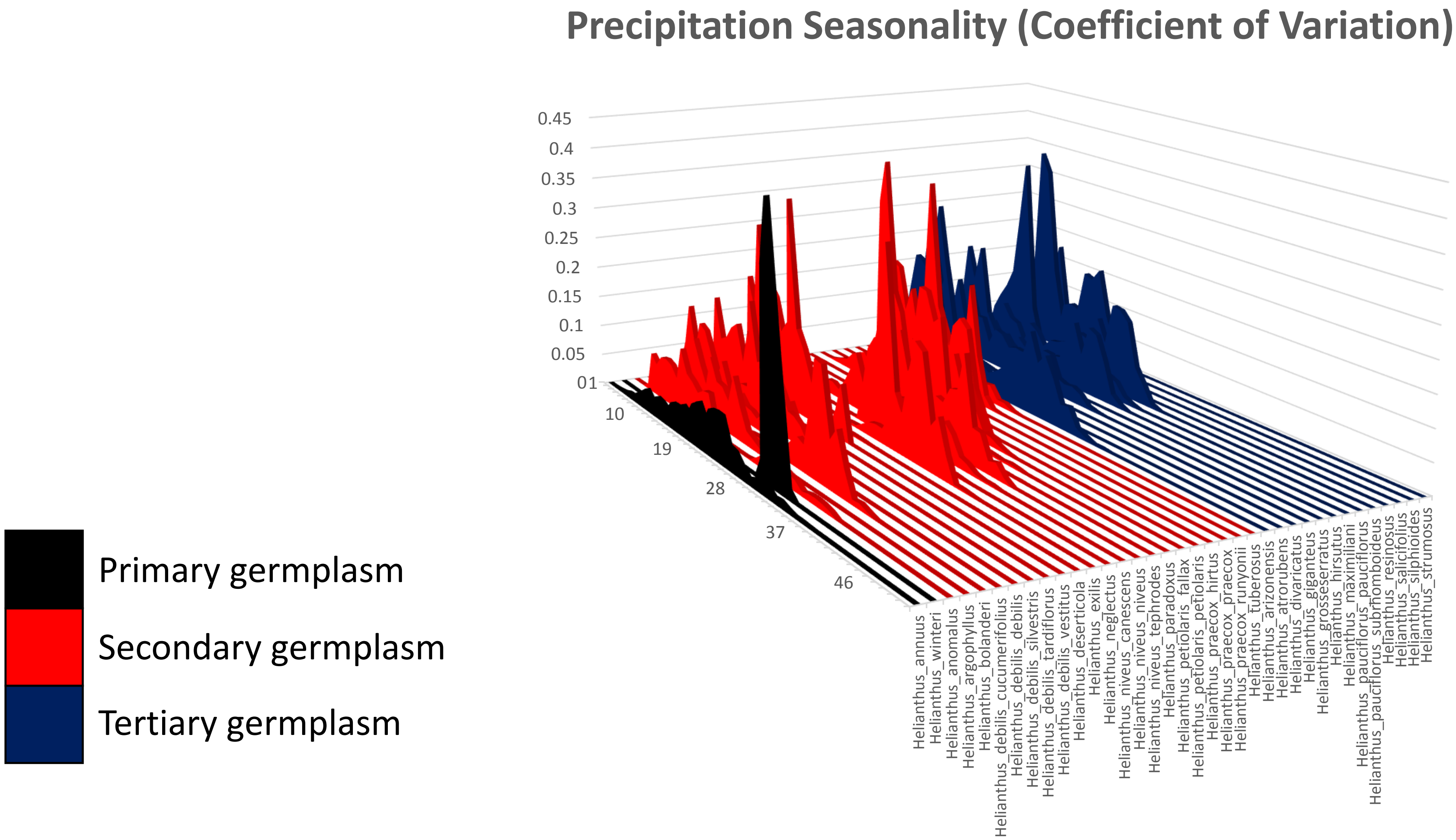

Predicted Niche Occupancy-Bioclim 16

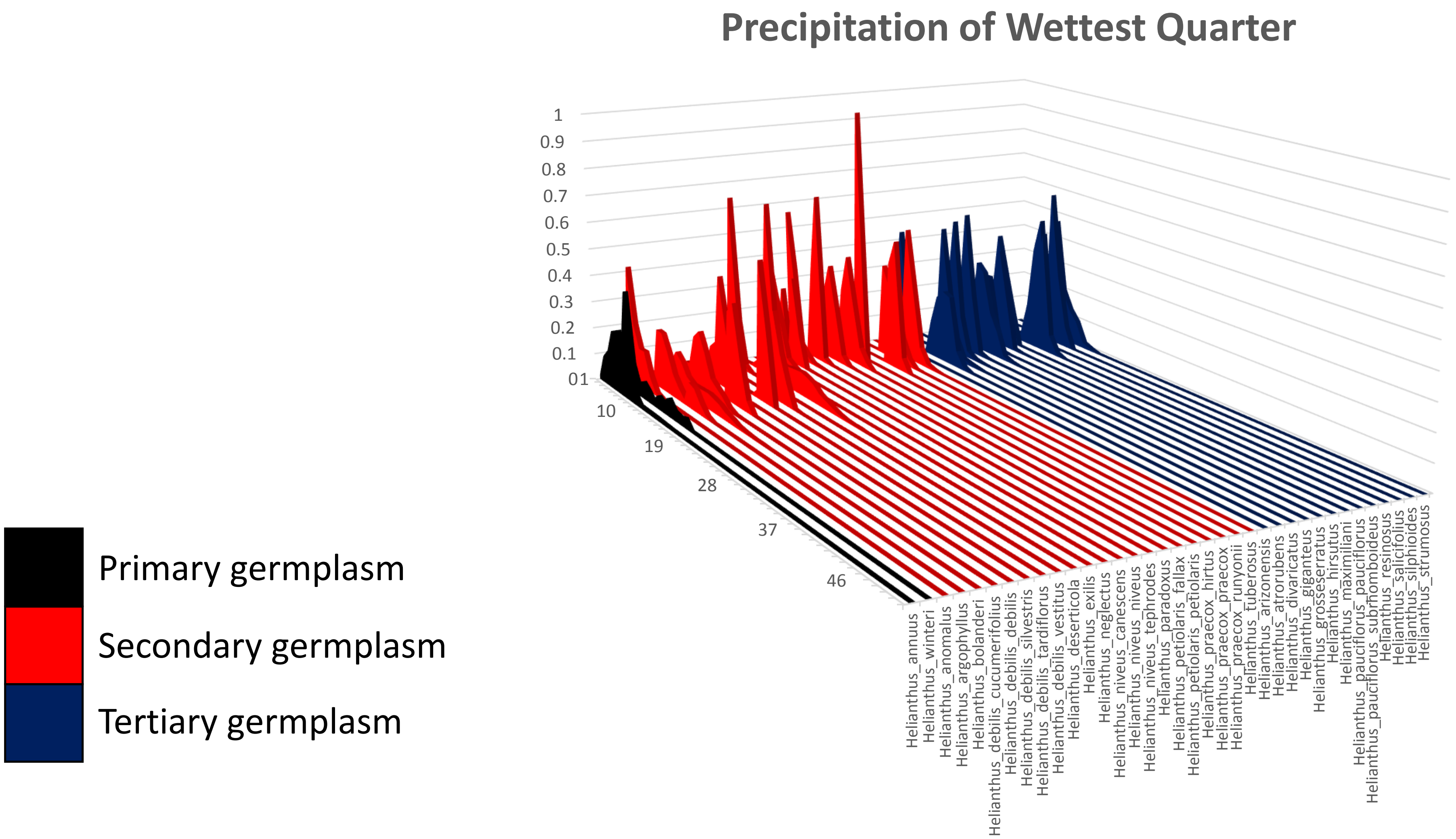

Predicted Niche Occupancy-Bioclim 17

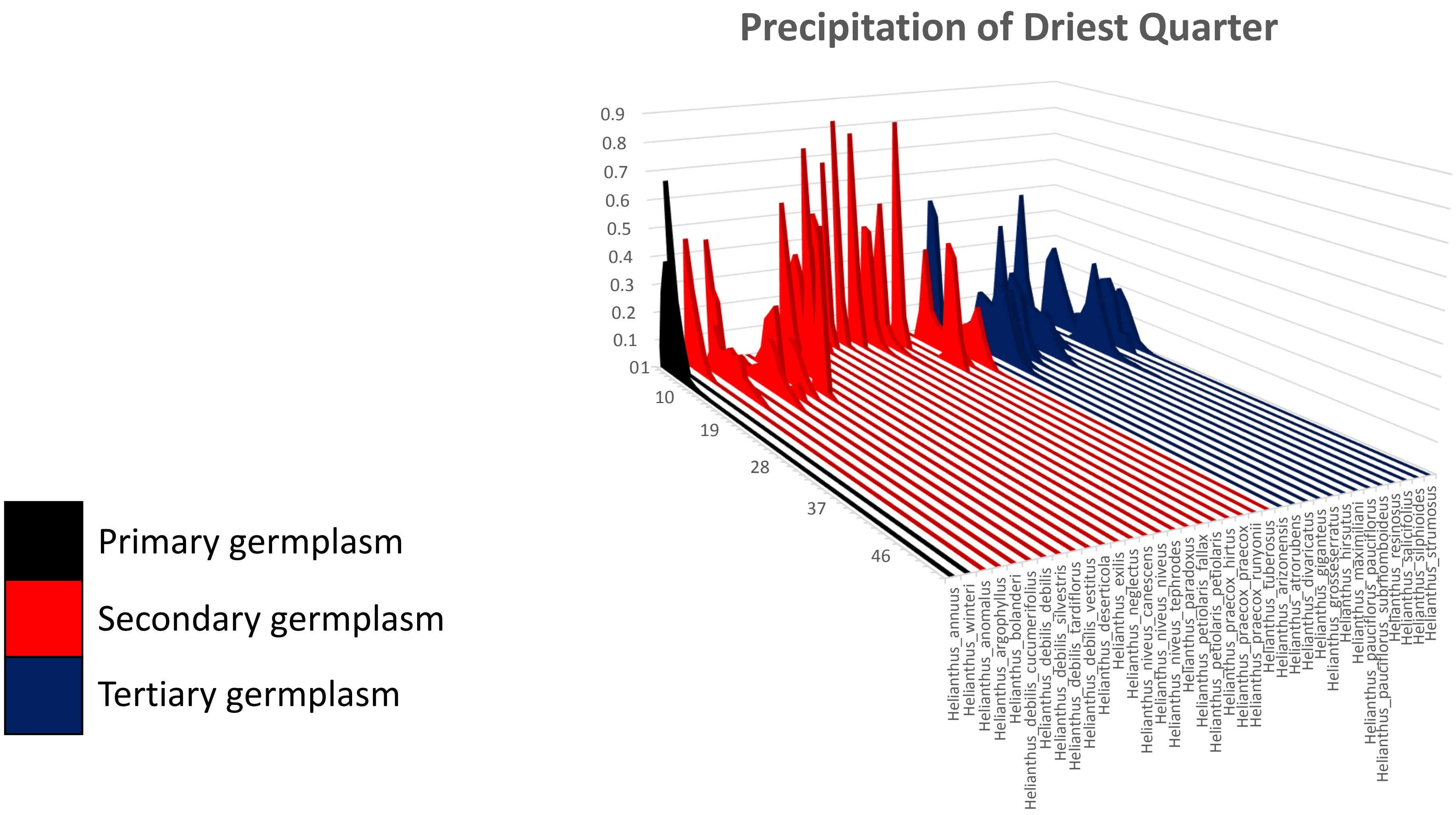

Predicted Niche Occupancy-Bioclim 18

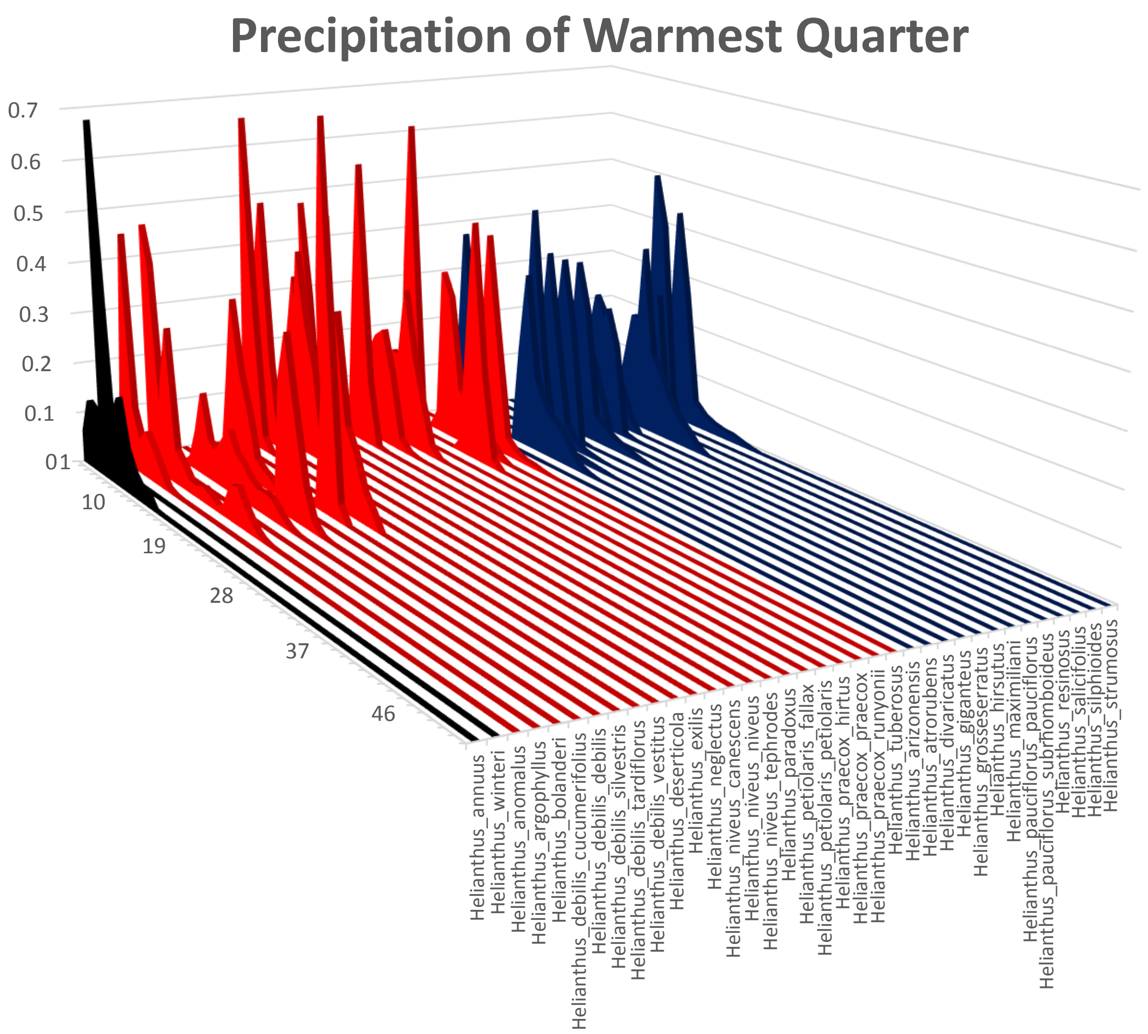

Primary germplasm

Secondary germplasm

Tertiary germplasm

Predicted Niche Occupancy-Bioclim 19

Precipitation of Coldest Quarter

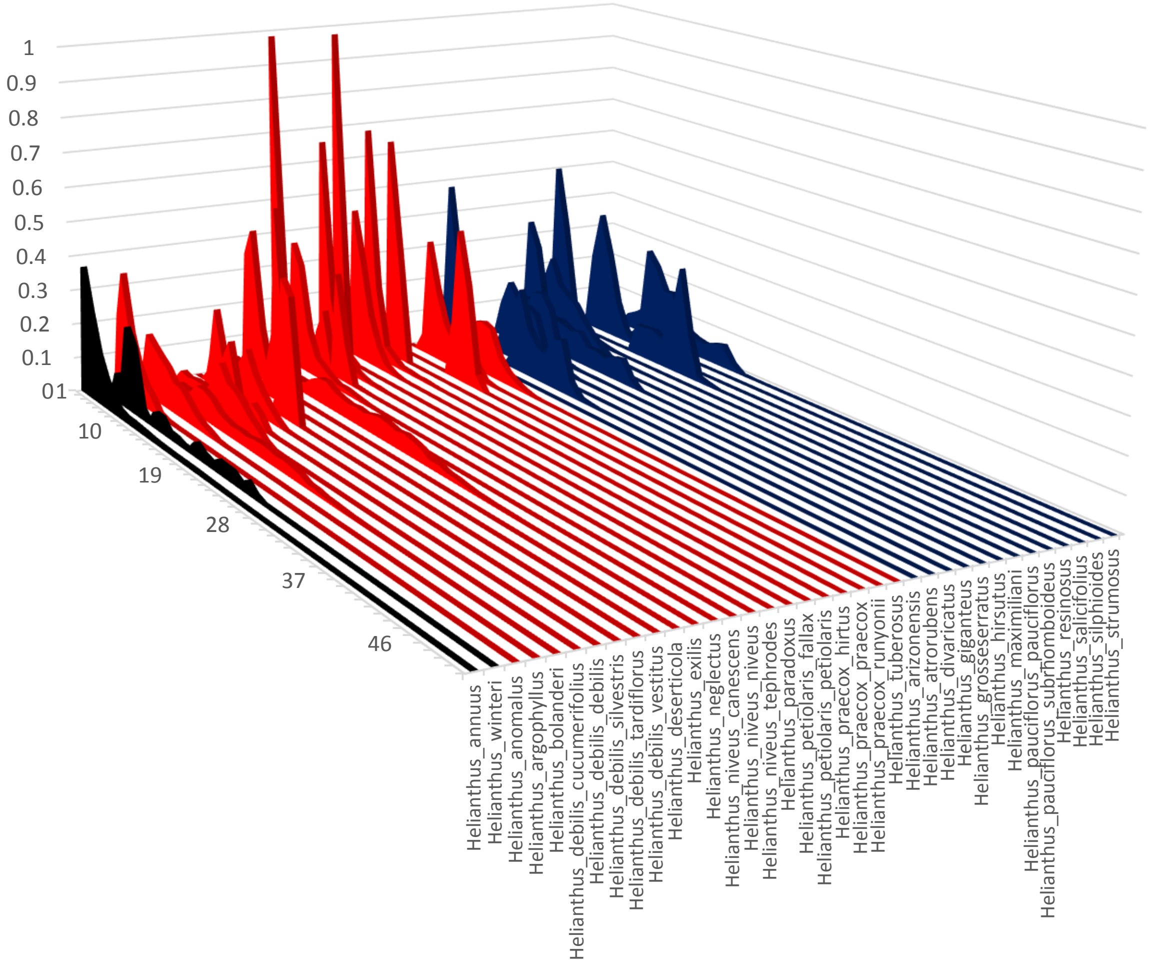

Primary germplasm

Secondary germplasm

Tertiary germplasm

Predicted Niche Occupancy-Bioclim 20

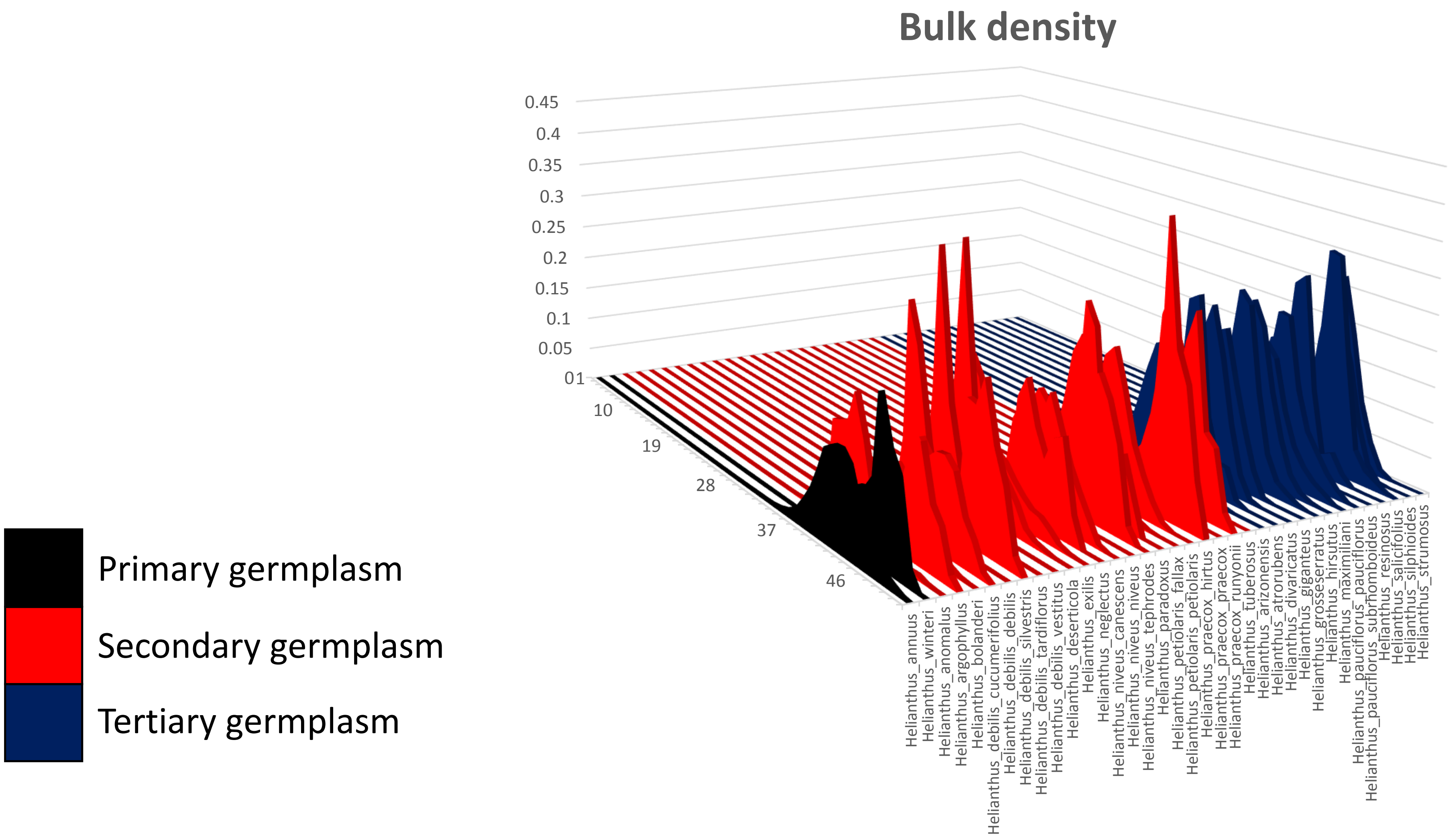

Predicted Niche Occupancy-Bioclim 21

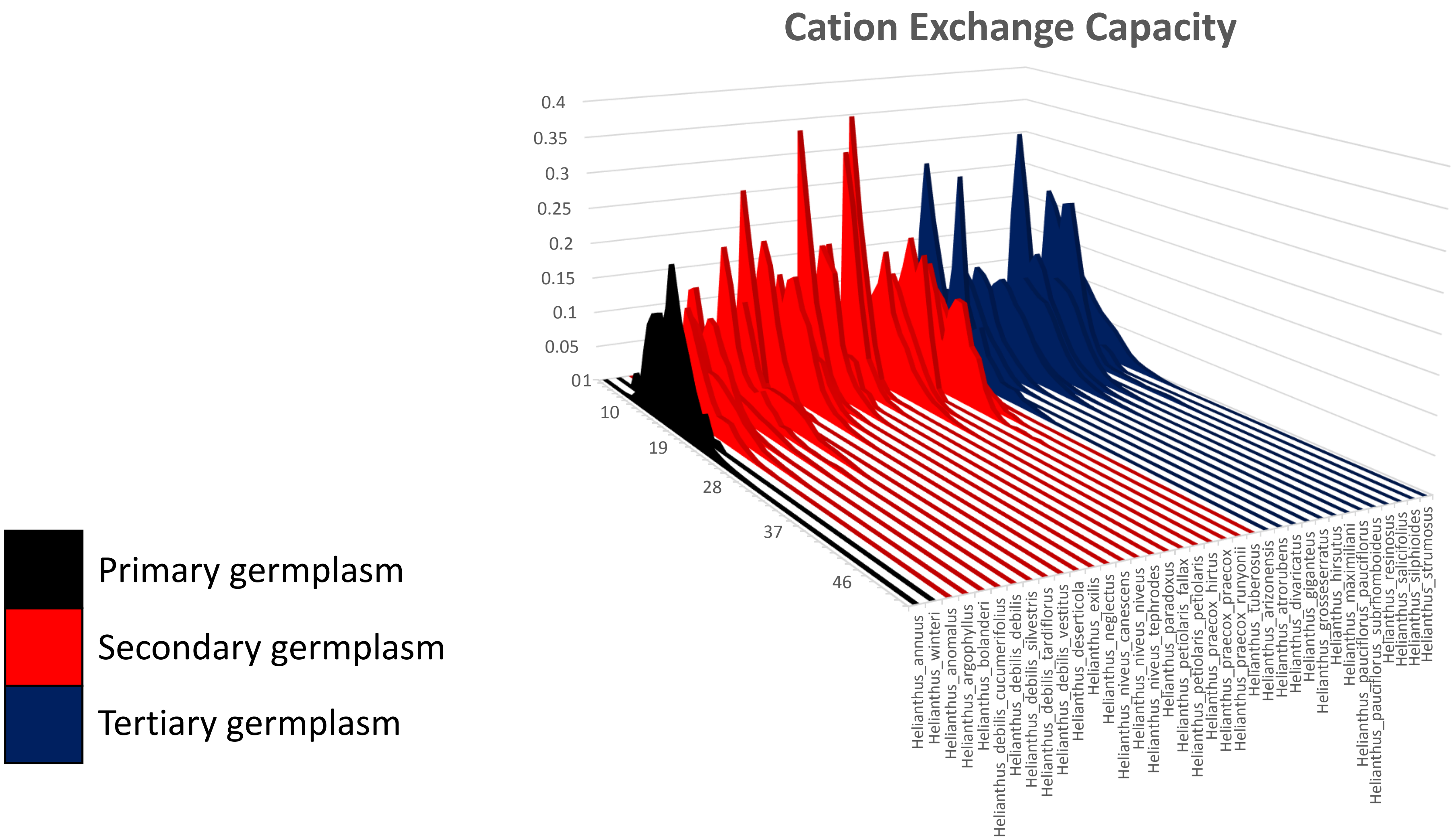

Predicted Niche Occupancy-Bioclim 22

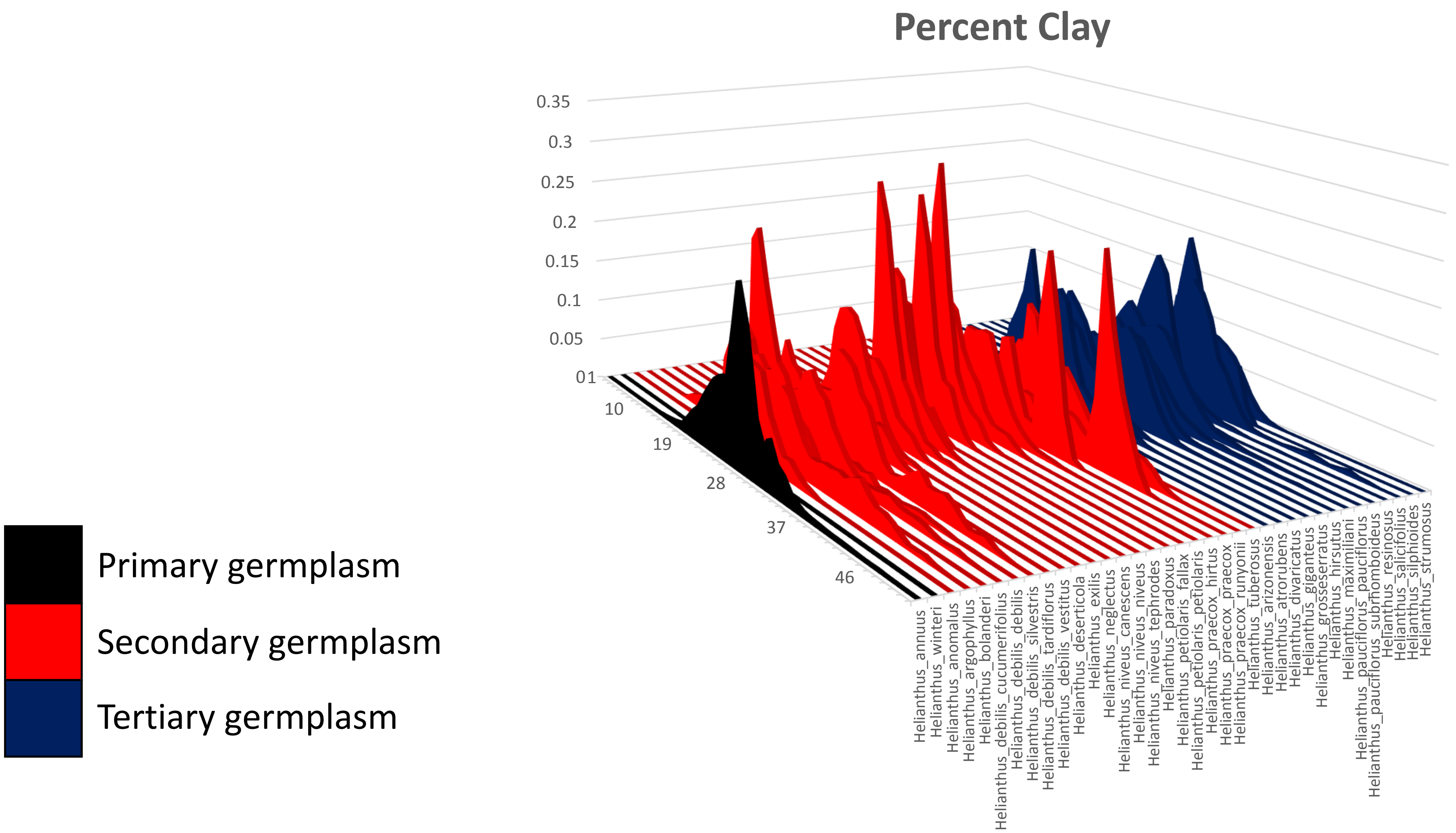

Predicted Niche Occupancy-Bioclim 23

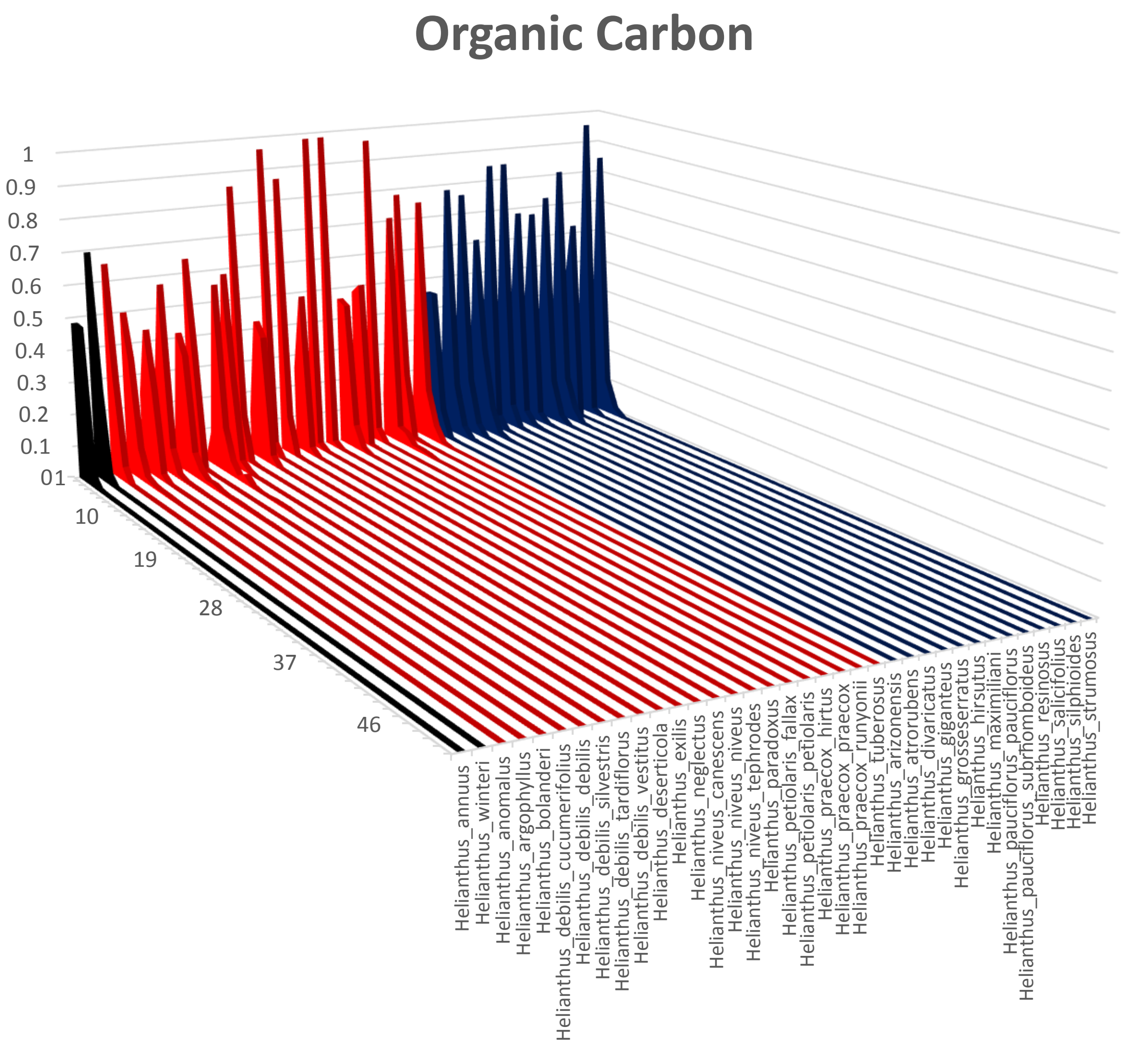

Primary germplasm

Secondary germplasm

Tertiary germplasm

Predicted Niche Occupancy-Bioclim 24

pH in H2O

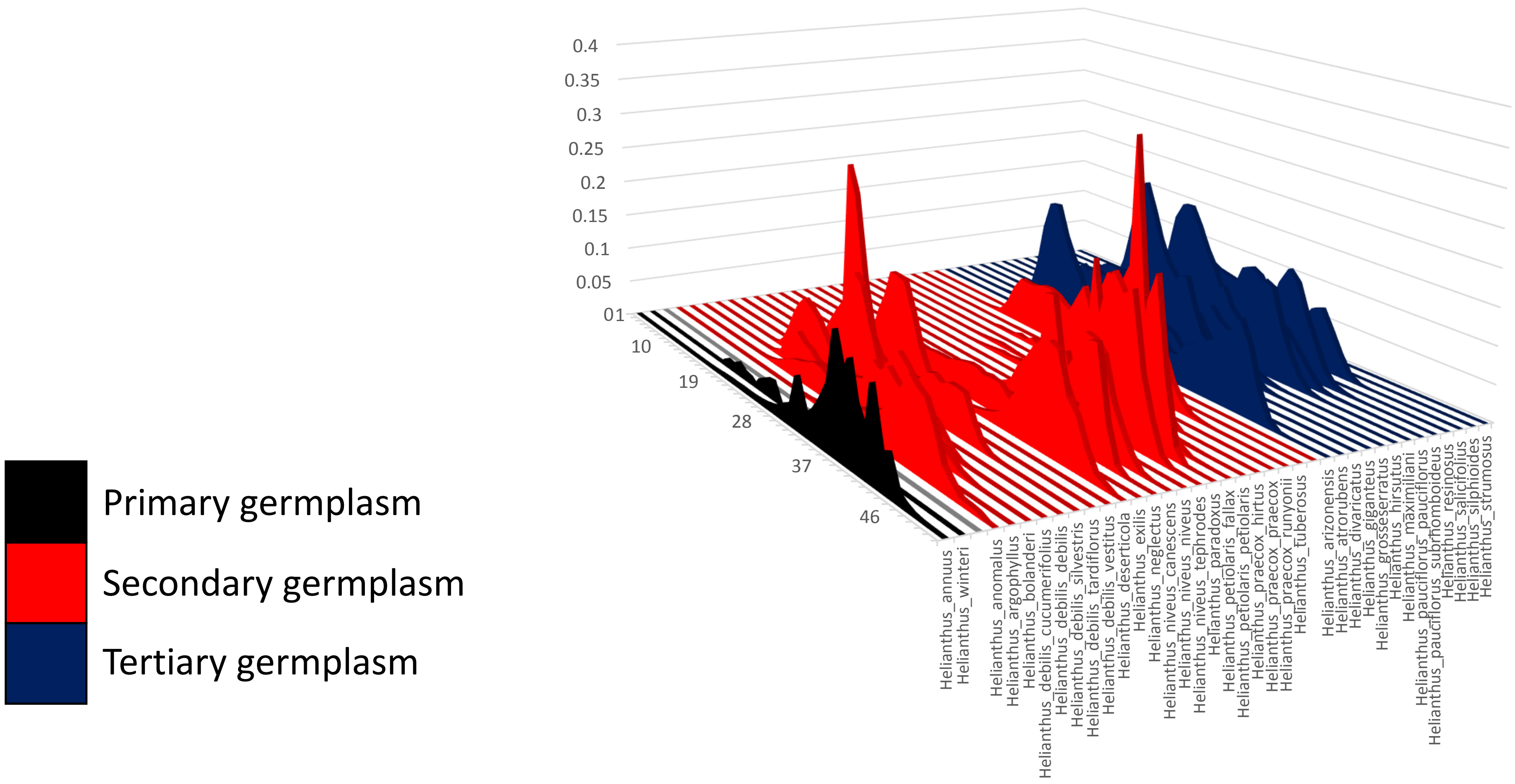

Predicted Niche Occupancy-Bioclim 25

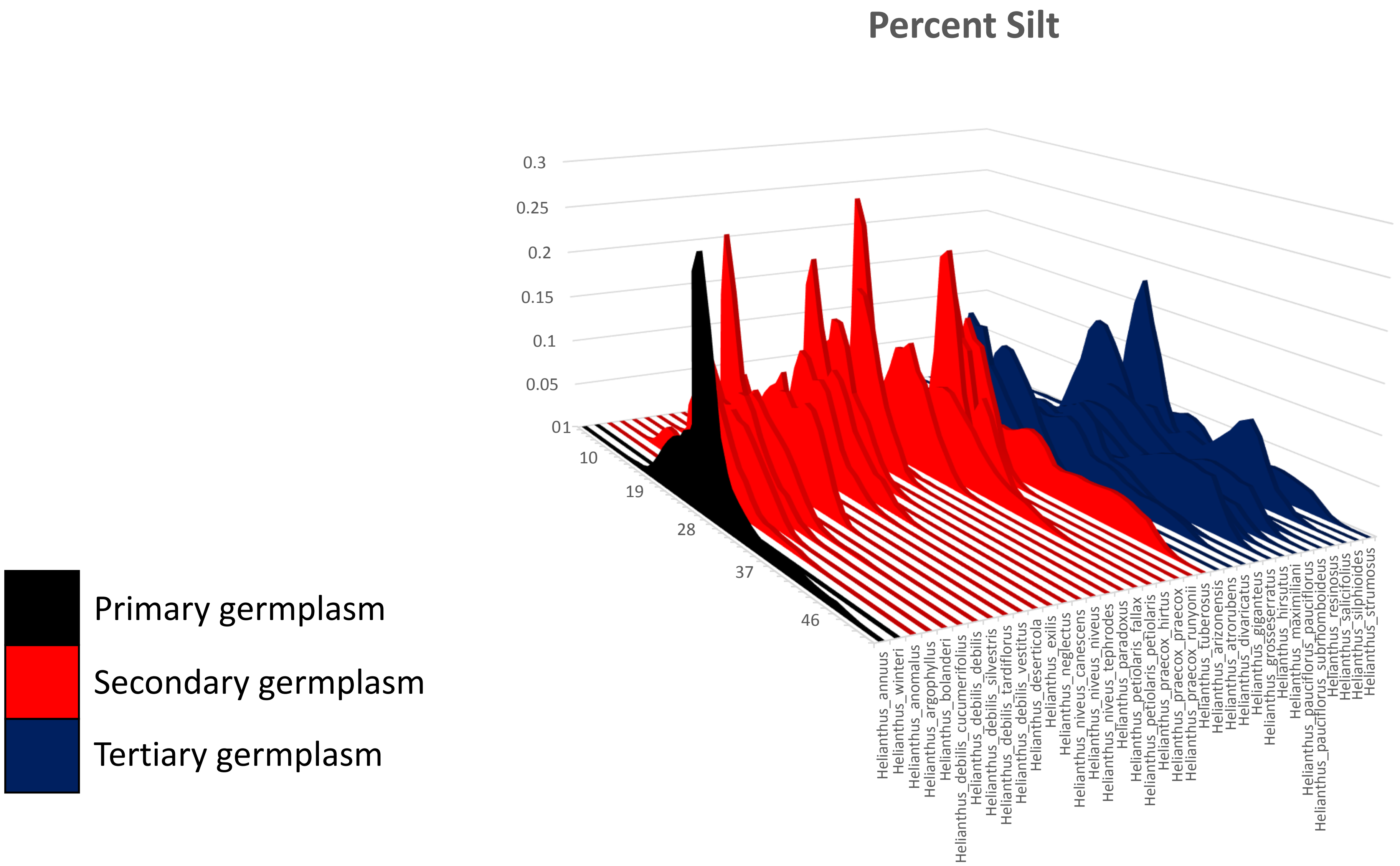

Predicted Niche Occupancy-Bioclim 26

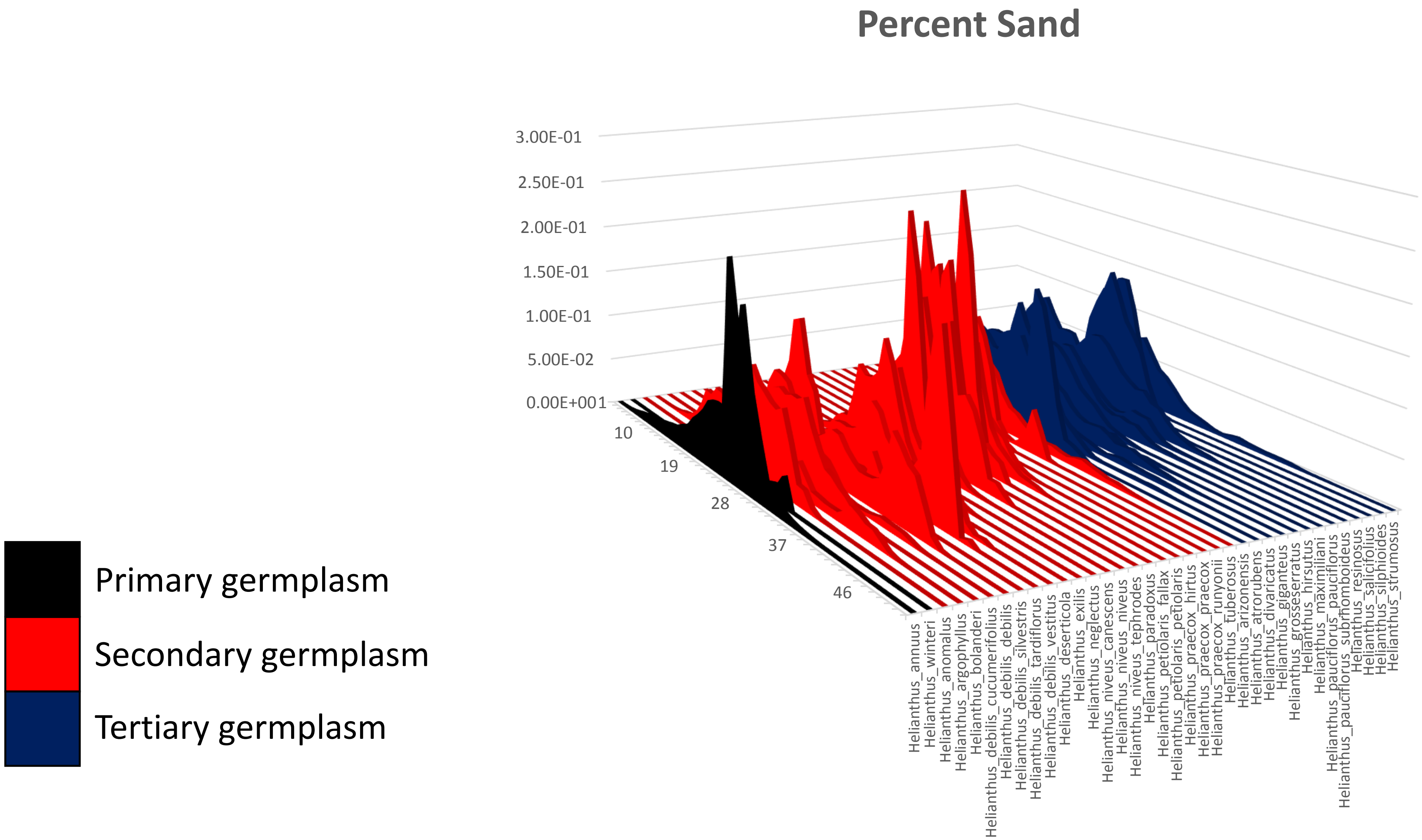

Variable	Recommendation for Collection
Bio 1	mean annual temperature
Bio 2	mean diurnal range
Bio 3	Isothermality
Bio 4	Temperature seasonality
Bio 5	Max temp warmest month
Bio 6	Min temp coldest month
Bio 7	Temperature annual range
Bio 8	Mean temperature wettest quarter
Bio 9	Mean temp of driest quarter
Bio 10	Mean temp warmest quarter
Bio 11	Mean temp coldest quarter
Bio 12	Annual precipitation
Bio 13	Precipitation in wettest month
Bio 14	Precipitation in driest month
Bio 15	Precipitation Seasonality
Bio 16	Precipitation in wettest quarter
Bio 17	Precipitation in driest quarter
Bio 18	Precipitation in warmest quarter
Bio 19	Precipitation in coldest quarter
Bio 20	Bulk density
Bio 21	Cation Exchange Capacity
Bio 22	Percent Clay
Bio 23	Organic Carbon
Bio 24	pH in H2O
Bio 25	Percent Silt
Bio 26	Percent Sand
